# Novel Functionalized Pyrrolopyridines to Target Brk

**DOI:** 10.3390/molecules31152730

**Published:** 2026-08-06

**Authors:** Erik Schmidt, Jannis von Veh, Anne-Christin Sarnow, Wolfgang Sippl, Julian Kowalski, Niels Heise, Frank Totzke, Andreas Hilgeroth

**Affiliations:** 1 Institute of Pharmacy, Martin-Luther-University Halle-Wittenberg, Wolfgang-Langenbeck-Str. 4, 06120 Halle, Germany; serik2@hotmail.de (E.S.); jannis-vonveh@web.de (J.v.V.); anne-christin.sarnow@pharmazie.uni-halle.de (A.-C.S.); wolfgang.sippl@pharmazie.uni-halle.de (W.S.); julian.kowalski@pharmazie.uni-halle.de (J.K.); 2 Institute of Chemistry, Martin-Luther-University Halle-Wittenberg, Kurt-Mothes-Str. 3, 06120 Halle, Germany; niels.heise@chemie.uni-halle.de; 3 Reaction Biology Europe GmbH, Engesserstr. 4, 79108 Freiburg, Germany; f.totzke@reactionbiology.de

**Keywords:** drug development, small-molecule inhibitors, substituent effects, protein kinase inhibitor

## Abstract

Background: Increasing resistance against protein kinase inhibitors used in cancer therapies enforces the search for novel target structures to be addressed with favourable small-molecule inhibitors. One of these novel target structures is the tyrosine kinase Brk that is known to play a prominent role in breast cancer progression. Moreover, Brk overexpression in various kinds of cancer is associated with poor outcomes, making Brk an interesting target structure for potential treatment. So far, no class of promising Brk inhibitors has been identified. Methods: We synthesized novel functionalized pyrrolopyridines in one- and two-step reactions under substitution of the molecular scaffold and the 4-aniline residue, respectively. They were evaluated as inhibitors of Brk and HER2 in a radiolabelled enzyme assay. Results: The most favourable substituents for Brk inhibitory activity at the aniline residues were 3-hydroxy functions combined with either bromo or nitro substituents at the molecular scaffold. Those compounds, as well as bromo- and nitro-substituted compounds, also showed the best HER2 inhibitor activities. Conclusions: Novel pyrrolopyridines were discovered to be a promising class of nanomolar Brk inhibitor with additional HER2 activities to further strengthen Brk inhibitory activity in prospective anticancer therapies. Thus, the first class of Brk inhibitors could be identified.

## 1. Introduction

With the understanding of cellular signalling pathways, protein kinases have been found to be attractive target structures for cancer therapy because they may be dysregulated in cancer cells [1,2]. Normally, they activate proteins through phosphorylation under ATP hydrolysis [3]. Activated proteins undergo different folding and following interactions to determine the activation of such cellular pathways [4]. In cancer cells, protein kinases are found to be overexpressed or overactive, causing aberrant functions such as cellular dysregulation that result in cancer cell proliferation [5,6].

Protein kinase inhibitors that bind to the active site of the kinases to prevent their activity have been developed [1,7]. However, their efficacy is limited by the sensitivity of the respective tumour cells [1,8]. In the case where resistance occurs towards such a kinase inhibitor, they sustain their proliferative signalling [8]. The reasons for such resistance may be mutations of the active site of the kinases that prevent inhibitor binding or alternative so-called bypass signalling pathways [9].

As resistance against these established protein kinase inhibitors in clinical use emerges, novel target structures have to be found that may be successfully addressed by developed inhibitors [1]. Preferably, such overexpressed or overactive target structures would be present in several kinds of cancer so that developed inhibitors may be widely used.

Brk, as a breast tumour kinase, is a nonreceptor tyrosine kinase and has been known since its origin, namely breast cancer cells, from which it was first cloned [10,11]. It functions via autophosphorylation and following activation at two different tyrosine residues, one being in the catalytic kinase domain [12].

Several transcription factors are phosphorylated by Brk, which activate the proliferation of cancer cells [13,14,15]. Brk is found to be overexpressed in all types of breast cancer, namely oestrogen receptor-positive, HER2-positive, and triple-negative breast cancer [16,17]. It contributes to cell migration, invasion, metastases and cell survival [18,19,20]. In addition to its prominent role in breast cancer, Brk is found to be overexpressed in prostate cancer associated with a poor prognosis [21,22]. In prostate cancer, Brk phosphorylates substrates involved in cancer cell proliferation, survival and migration [23]. In colon cancer, Brk activates STAT3, which is known to promote survival and proliferation of cancer cells [24]. Also, in non-small cell lung cancer, Brk overexpression is associated with decreased survival [25,26]. Moreover, Brk overexpression is found to be induced by used chemotherapeutics, as well as in pancreatic cancer and bladder cancer [27,28]. Thus, Brk is a promising target structure for various kinds of cancer.

Presently, no specific Brk inhibitors are used in cancer therapies because the few discovered inhibitors have different target structures, like vemurafenib as a BRAF inhibitor and dasatinib as a BCR-ABL inhibitor, and only address Brk as an off-target [29,30,31]. Other published inhibitors proved to be inactive in cancer cell proliferation studies.

We discovered functionalized pyrrolopyridines as a novel class of promising Brk inhibitors and evaluated protein kinase inhibitor potential depending on various substitution patterns, both at the molecular scaffold and at the 4-position of the pyridine core. Thus, we identified nanomolar Brk inhibitors within a novel class of promising protein kinase inhibitors with relevance to various kinds of cancer.

## 2. Results and Discussion

Protein kinase inhibitors that bind to the active site cavity of the ATP-binding pocket generally undergo hydrogen bonding to the amino acid hinge region via one *N*H and one nitrogen function separated by a carbon atom of a heterocyclic system, as in the BRAF inhibitor vemurafenib with a pyrrolopyridine core. Specific protein kinase binding results from functional residues at the respective core with a 3-(4-chlorphenyl) substituent and a substituted carbonylphenyl residue at the 5-position of the annelated pyrrolo ring in vemurafenib [32]. The pyrrolopyridine nucleus is also found in JAK inhibitors with a specific JAK binding of a 3-pyrazolo residue and a 4-amino residue [33].

We used the unsubstituted pyrrolopyridine core and derivatized it in the 4-position with variously substituted aniline residues. Thus, a known specific kinase binding of vemurafenib could be excluded. By using the established pyrrolopyridine core, we, thus, evaluated the off-target Brk properties by enhancing potential binding abilities with residues placed in the 4-position of the central heterocyclic compound.

**Scheme 1:**
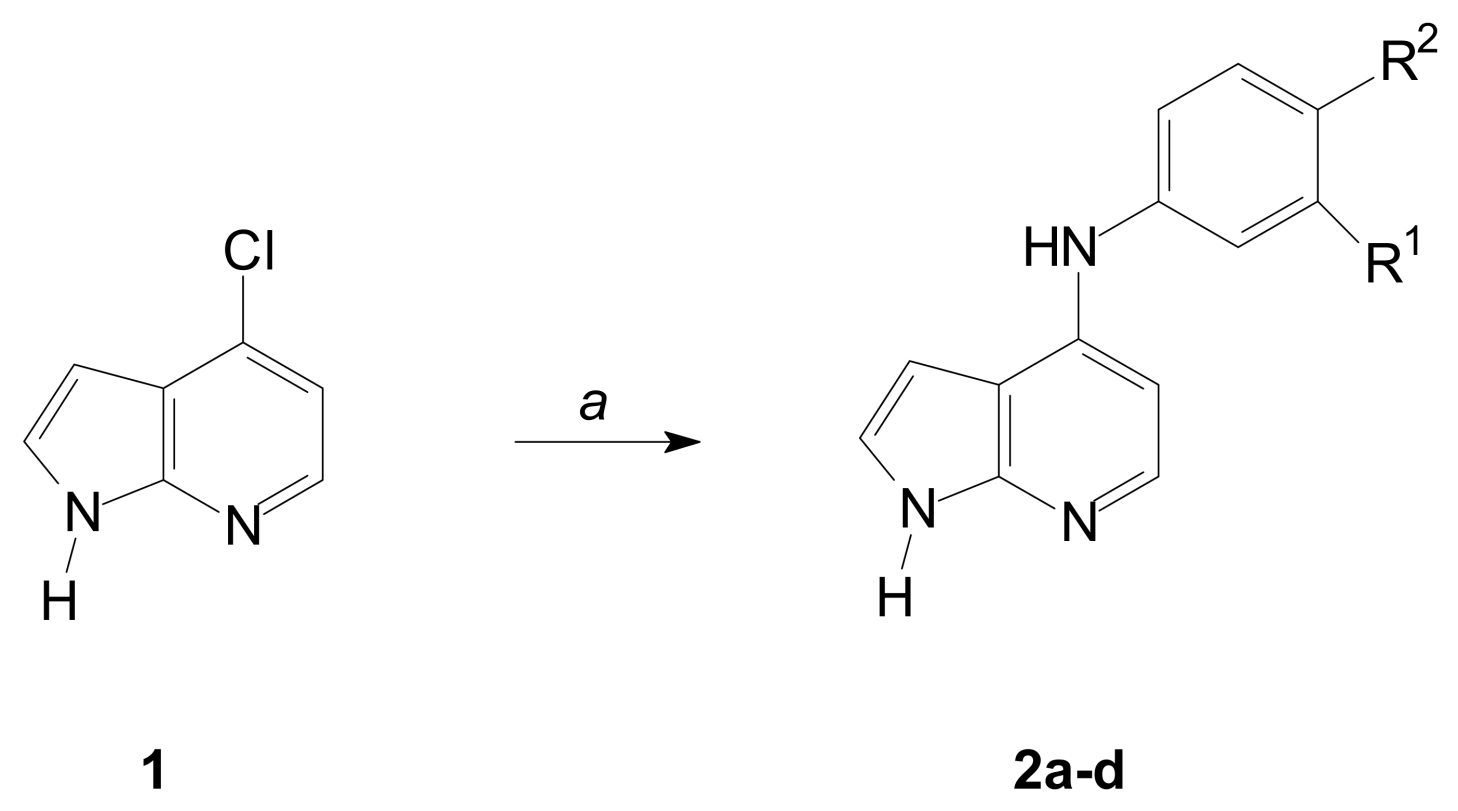
Reagents and conditions: (*a*) aniline derivative, reflux, NMP.

So, 4-chloropyrrolopyridine compound **1** was heated under reflux with various anilines in *N*-methylpyrrolidone (NMP) to give compounds **2a**–**d** after workup of the mixtures and final purification using column chromatography over silica gel (Scheme 1).

**Scheme 2:**
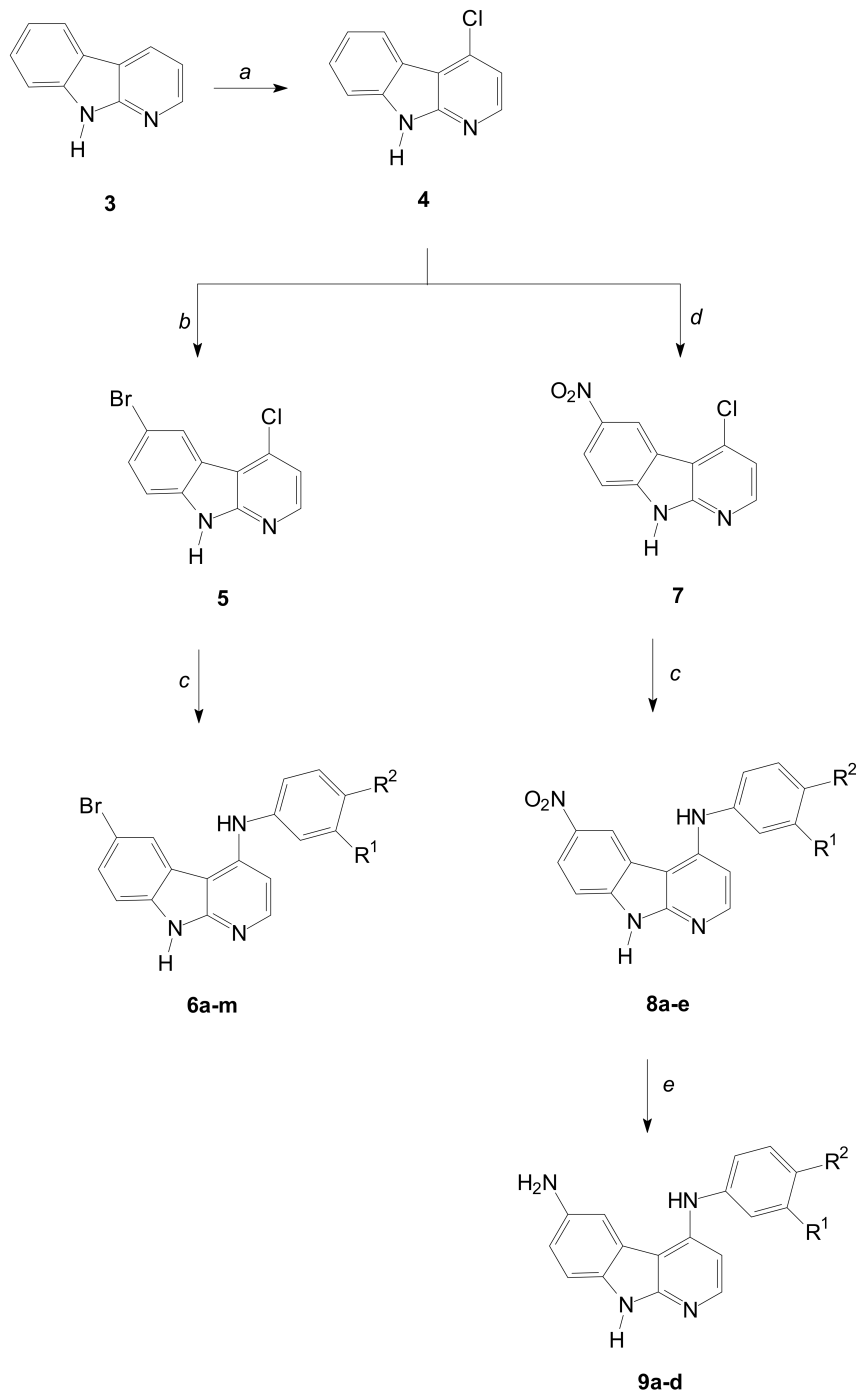
Reagents and conditions: (*a*) hydrogen peroxide, reflux, acetic acid; phosphorus oxychloride, room temperature, DMF; (*b*) bromine, room temperature, acetic acid; (*c*) aniline derivative, reflux, NMP; (*d*) nitric acid, 5 °C to room temperature; (*e*) tin(II) chloride, reflux, hydrochloric acid.

In order to increase the molecular scaffold for further functionalization, the benzo-annelated pyrrolopyridine **3** was first 4-chlorinated to compound **4** after formation of a pyridine *N*-oxide intermediate by the use of hydrogen peroxide following treatment with phosphorus oxychloride (Scheme 2).

Then, various substitutions of the anellated phenyl residue were performed via electrophilic substitution at the *para* position to the *N*H pyrrolo function.

Following this, bromination was achieved via the use of bromine in acetic acid to produce compound **5**, which was consequently treated with various anilines in NMP to yield compound series **6a**–**m**.

Then, compound **4** was treated with nitric acid to produce compound **7**, which was treated with various anilines in NMP under reflux to yield compound series **8a**–**e**. Lastly, the nitro functions in compounds **8** were reduced to amine functions with tin(II) chloride in acetic acid to yield compound series **9a**–**d**.

The purity of the target compounds was checked via HPLC analysis, using a gradient eluent mixture of water and methanol with increasing methanol portions. Nearly all of the compounds had a purity > 95%, although the final yields of some target compounds were poor due to the need for repetition of the final purification procedure via column chromatography.

The inhibitory activity of the compounds was determined at increasing concentrations by measuring the degree of phosphorylation of a Brk substrate as kinase activity via the use of radiolabelled ATP and the scintillation counting technique. Each concentration-dependent kinase activity value was subtracted from 100 to yield the corresponding inhibition value. Lastly, the IC_50_ value of each compound was calculated from all the resulting concentration-dependent inhibition values. The values are shown in Table 1.

The 4-anilino pyrrolopyridine **2a** with 3-chloro substitution was not active as a Brk inhibitor and had an IC_50_ value of 92.9 µM. When the 3-chloro function was replaced with a nitro function, the IC_50_ value in compound **2b** mainly increased to 1.45 µM. We introduced an additional methyl function in the 4-position of the 4-anilino substituent of compound **2a**. The disubstitution of derivative **2c** also increased the inhibitory activity to a value of 9.97 µM. Then, we replaced the 3-chloro function of compound **2c** with a hydroxy function that could undergo potential hydrogen acceptor or donator bonding. We achieved nanomolar activity for compound **2d** with an IC_50_ value of 0.83 µM. Thus, compound **2d** was more active than the staurosporine control used, with an IC_50_ value of 0.89 µM.

We then attached a substituted phenyl residue to the pyrrolo function to exclude side effects in the inhibition of protein kinases affected by known pyrrolopyridines. The 6-bromo-substituted compound **6a** with the 3-chloro function in the 4-anilino residue resulted in largely improved Brk inhibition, with an IC_50_ value of 2.76 µM compared to that of compound **2a**. In derivative **6b**, with an additional 4-methyl function at the aniline substituent, we achieved further improved activity, with an IC_50_ value of 1.26, similar to the improvement observed for compound **2c**. When the 4-methyl function was replaced with a chloro function in compound **6c**, the activity decreased, with an IC_50_ value of 2.04. However, this activity was better than that of the only 3-chloroaniline-substituted derivative **6a**.

When the 3-chloro function was replaced with a 3-nitro function in derivative **6d**, inhibitory activity decreased. The addition of a 4-methyl substituent again increased the activity of compound **6e**, similarly to compound **6b**. When the 4-methyl group was replaced with a fluoro function in derivative **6f**, activity further increased. We then investigated the influence that a 4-substituent with a potential hydrogen bond donator or acceptor function could have on inhibitory activity. Derivative **6g** with a 4-amino function increased the activity to nanomolar ranges, with an IC_50_ value of 0.43 µM. A 4-hydroxy substituent in the 3-anilino residue of derivative **6h** further improved that nanomolar activity.

When the 4-favourable fluoro substituent was placed in the 3-position of the aniline residue in compound **6i**, nanomolar inhibitory activity resulted. A trifluoro methyl function, however, instead of the 3-fluoro function in derivative **6j**, was not successful, with largely decreased inhibitory activity measuring 27.1 µM. When the trifluoro function with its large space demand was replaced with a smaller methoxy function in compound **6k**, inhibitory activity mainly increased to an IC_50_ value of 0.04 µM. A 3-hydroxy function with both hydrogen bond acceptor and donator activity in the 3-aniline residue was most effective in compound **6l**, with a picomolar activity of <0.003 µM, exhibiting the best inhibitory activity so far. When, in that compound, a methoxy function was added in position 4 of the aniline residue in derivative **6m**, activity decreased to 0.02 µM. However, compound **6m** exhibited the second-best inhibitory activity of all the compounds. Concerning the structure–activity relationships, it can be concluded that hydrogen bond acceptor functions in the 3-anilino residues mainly increase the inhibitory activity of benzo-annelated compounds **6**, preferably if they are placed in the 3-position.

Next, we investigated the effect of a 6-nitro function in compound series **8a**–**e** with various 4-anilino substituents. Derivative **8a** with 3-chloroaniline substitution exhibited slightly decreased Brk inhibitory activity compared to compound **6a**. Compound **8b** with the additional 4-methyl function, however, was more active, with an IC_50_ value of 1.01 µM compared to compound **6b**. A 3-ethoxy function at the 4-aniline residue of compound **8c** resulted in decreased Brk inhibitory activity. When that 3-ethoxy function was replaced with a hydroxy function, the activity of compound **8d** largely increased, similar to that of derivative **6l**, with an IC_50_ value in the picomolar range <0.003 µM. Derivative **8e** without a 3-aniline but with 4-trifluoromethyl substitution was not promising, with an IC_50_ value of 7.85 µM. Generally, it can be stated that for the different 6-substitutions at the molecular scaffold, the resulting Brk inhibitory activities largely depend on favourable substitution at the 3-aniline substituent, with the 3-hydroxy function being the best so far.

We then reduced the 6-nitro function at the molecular scaffold to an amino function that could both serve as a hydrogen bond acceptor and fulfil a donator function similar to that of the 3-hydroxy group at the 3-aniline residue.

Compound **9a** with the 3-chloroaniline substituent resulted in a nearly tenfold increase in activity, with an IC_50_ value of 0.365 µM, compared to compound **8a** with the 6-nitro function. Derivative **9b** with the additional 4-methyl substituent in the 3-aniline residue, however, was less active than compound **8b**. Compound **9c** with the 3-ethoxy function again resulted in increased Brk inhibitory activity, with an IC_50_ value of 3.41 µM compared to derivative **8c**. Lastly, compound **9d** with the 3-hydroxyaniline substitution again resulted in the best activity, similarly to that in compound series **6** and **8**, with an IC_50_ value in the lower nanomolar range of 0.01 µM. Thus, it is possible to conclude that the 6-anilino function is more favourable for potential hydrogen bonding to the protein backbone, compared to bromo and nitro substitutions, when it is combined with exclusive 3-anilino substitution.

In breast cancer, Brk is activated by the HER2 receptor ligand [34]. In HER2-positive breast cancer, studies have described increasing resistance to the kinase inhibitors used in clinical settings, lapatinib and neratinib [7,35]. Given this, we aimed to investigate whether our novel Brk inhibitor series **2**, **6**, **8** and **9** possessed additional HER2-inhibiting properties. Such inhibition would increase the potential of Brk inhibition by reducing Brk activation via HER2 and would, additionally, be useful in the treatment of resistant HER2-positive breast cancer, as an alternative to the small-molecule inhibitors used with the resistances described. Moreover, compared to the monoclonal antibodies used in HER2-positive breast cancer treatment, such as trastuzumab, our small-molecule inhibitors would be an attractive alternative owing to their lower production costs [36].

Compound **2a** was not active as a HER2 inhibitor, whereas derivative **2b** exhibited residual activity that further increased with the introduction of a methyl group at the aniline residue in derivative **2c**. However, compound **2d** with a 3-hydroxy function at the aniline residue was not more active as a HER2 inhibitor.

Additionally, compound **6a** with the 3-chloroaniline function did not exhibit HER2 activity. In derivative **6b**, with an additional 4-methyl function at the aniline residue, activity increased. When that methyl function was replaced with a chloro substituent in derivative **6c**, activity decreased.

The 3-nitro group at the aniline residue of compound **6d** was more favourable than the 3-chloro function of compound **6a**. Again, an additional 4-methyl group in compound **6e** increased activity, and replacement of the 4-methyl function with a fluoro function in derivative **6f** was more favourable. A hydrophilic amino function at the 4-position of the aniline residue in compound **6g** was not favourable for HER2 inhibition; however, a hydroxy group at the 4-position of compound **6h** demonstrated similar results to that of compound **6f**.

The 3-fluoro group in derivative **6i** was more favourable than the 3-nitro function of compound **6d**. However, a trifluoromethyl function instead of the fluoro group in derivative **6j** led to a loss of activity. The 3-methoxy group of compound **6k** resulted in similar activity to the 3-fluoro function of compound **6i**. When that group was replaced with the 3-hydroxy group, activity against HER2 increased in compound **6l**, whereas an additional 4-methoxy group at the aniline residue in derivative **6m** decreased the activity. Thus, compound **6l** was considered to exhibit the best dual-activity action against Brk and HER2.

Derivative **8a** demonstrated residual HER2 activity. Again, the additional 4-methyl function in compound **8b** increased this activity. The more space-demanding 3-ethoxy function in derivative **8c** was less favourable, whereas replacement with the hydroxy group in derivative **8d** resulted in the best activity within that series, similarly to compound **6l**. The trifluoromethyl function in the 4-position of the aniline residue of compound **8e** led to a loss of activity. Comparing the 6-nitro with the 6-bromo substitution at the molecular scaffold, it can be stated that the nitro function was more favourable, with comparably increased activities in those derivatives with the same aniline substitutions.

A reduction of 6-nitro to the 6-amino function in series **9** led to largely increased activity against HER2 in compound **9a** compared to derivative **8a**. A similar result of increased activity was found for derivative **9b** compared to **8b**; however, the 3-ethoxy aniline derivative **9c** was less active than derivative **8c** with just residual activity. Moreover, compound **9d** exhibited a slight decrease in activity compared to derivative **8d**. However, the 6-amino function at the molecular scaffold tended to be more favourable than the 6-nitro function, as shown for compounds **9a** and **9b**. With compounds **8d** and **9d**, we identified two more dual inhibitors of both kinases Brk and HER2, respectively.

**Figure 1:**
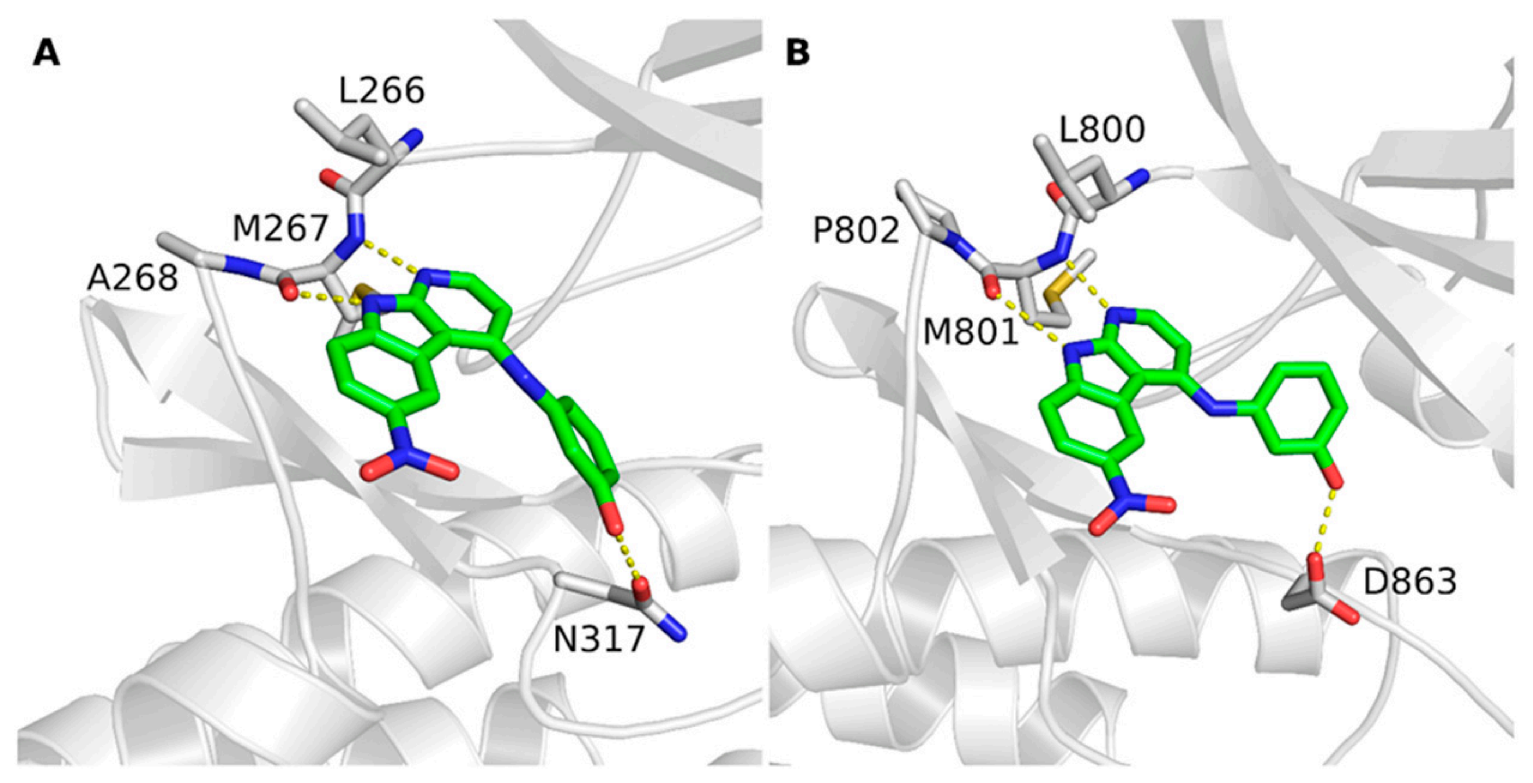
Top-ranked docking pose of compound **8d** in Brk (**A**) and HER2 (**B**). The protein backbone is represented as white ribbon, while important binding site residues are shown as white sticks. The ligand is presented as green sticks. Hydrogen bonds between Brk or HER2 and the ligand are displayed as yellow dashed lines.

Docking studies were performed for the synthesized pyrrolopyridine derivatives **6j**, **6l**, **8d**, and **8e** in the ATP-binding pocket of Brk and HER2. The docking results obtained revealed a common binding mode for all four inhibitors in both kinases. The pyrrolopyridine core formed two hydrogen bonds with the kinase hinge region, involving the backbone atoms of Met267 in Brk and Met801 in HER2. Furthermore, the 3-hydroxy substituents of compounds **6l** and **8d** formed a hydrogen bond with Asn317 (Brk) and Asp863 (HER2) in the kinase back pocket. This observation supports the enhanced inhibitory activity of **6l** and **8d** against Brk and HER2. Docking poses for the highly active inhibitor **8d** in Brk and HER2 are shown in Figure 1. Co-folding with Boltz-2 predicted the same binding mode for all the inhibitors investigated.

The predicted ADME properties obtained using QikProp in Schrödinger further highlighted the favourable drug-like characteristics of compounds **6l** and **8d**. Both compounds satisfied Lipinski’s Rule of Five without violations. Moreover, compound **6l** exhibited high predicted intestinal permeability values (Caco-2 = 757 nm/s) and 100% predicted human oral absorption. Compound **8d** exhibited the lowest molecular weight (320 Da), favourable lipophilicity (QPlogPo/w = 2.42), and the highest predicted aqueous solubility (QPlogS = −4.28).

Lastly, we evaluated the effect of the selected compounds on breast cancer cell growth as a proof of principle. We selected compound **6a**, with moderate inhibition of Brk and no HER2 activity; compound **6k**, with largely improved activity against Brk and residual HER2 activity; and derivative **6l**, with the best Brk inhibitory activity and moderate HER2 inhibitory activity. The inhibition of breast cancer cell growth was determined by the NCI (National Cancer Institute) via a fluorescence assay determining the fluorescent sulforhodamine dye bound to cellular proteins in growing cells.

The various breast cancer cells lines are shown in Table 2, with values of the compound concentrations determined for reducing cellular growth inhibition by 50% (GI_50_).

Compound **6a** resulted in GI_50_ values from 3.73 to 35.9 µM in the breast cancer cell lines, with a mean of 17 µM. Thus, compound **6a** exhibits similar activity to the lapatinib control [37]. For compound **6k**, with largely improved Brk inhibitory activity, GI_50_ values were reduced, with a range of 0.05 to 2.98 µM and a mean value of 1.66 µM, reflecting largely improved Brk inhibitory activity. Finally, the best compound, **6l**, as a dual inhibitor with improved activity against Brk and moderate activity against HER2, resulted in GI_50_ values of 0.44 µM to 1.03 µM, with an improved mean value of 0.67 µM. With the correlation of increased enzyme inhibition activity and increased inhibition of cancer cell growth, evidence indicates that the reduced cancer cell growth resulted from reduced enzyme activity. Moreover, we determined the effect of compound **6l** without the 6-bromo function at the molecular scaffold and an IC_50_ value of 0.003 µM for Brk inhibition on the phosphorylation of STAT3, which is involved in the proliferation of breast cancer cells [13,14]. At a concentration > 0.1 µM, we observed a block of STAT3 phosphorylation, revealing corresponding cellular effects in breast cancer cells in relation to the nanomolar inhibition of Brk in vitro. Additionally, the NCI determined the cellular toxicity for compounds **6a**, **6k** and **6l** as LC_50_. Only compound **6l** exhibited cytotoxic effects in the MDA-MB-231 cells, with an LC_50_ value of 7.67 µM. In the other breast cancer cell lines, compound **6l** was not cytotoxic, with LC_50_ values > 100 µM. Additionally, compounds **6a** and **6k** resulted in LC_50_ values > 100 µM in all breast cancer cell lines and, thus, were not cytotoxic.

## 3. Materials and Methods

General: Commercial reagents were used without further purification. The ^1^H-NMR spectra (500 MHz resp. 400 MHz) were measured using tetramethylsilane as internal standard, using an Agilent Technologies VNMRS (Agilent Technologies, Santa Clara, CA, USA). ^13^C-NMR spectra of sufficiently soluble compounds were conducted at 151 MHz. Due to the low solubility of the compounds, only APT spectra could be measured. Thin-layer chromatography (TLC) was performed on E. Merck 5554 silica gel plates (Merck, Darmstadt, Germany). High-resolution mass spectra were recorded using a Bruker Apex III mass spectrometer (Bruker, Billerica, MA, USA).

**Formation of the 4-aniline-substituted 1*H*-pyrrolo[2**,**3-*b*]pyridines (2a**–**d)**

One equivalent of 4-chloro-1*H*-pyrrolo[2,3-*b*]pyridine **1** and five equivalents of the respective aniline were dissolved in NMP (5 mL) under inert atmosphere and heated under reflux at 170 °C for 6 h. The reaction mixture was cooled to room temperature, diluted with ethyl acetate (20 mL) and subsequently extracted with water (20 mL, pH 10). The aqueous phase was then extracted with ethyl acetate (20 mL) twice. The unified organic layers were washed with water (20 mL) and dried over water-free sodium sulfate. The residue was filtered off and the solvent removed under reduced pressure. The remaining oil was purified via column chromatography using silica gel and a mixture of cyclohexane, acetonitrile and ethyl acetate (2:1:1) as eluent.

***Data for N-(3-chlorophenyl)-1H-pyrrolo[2*** *,**3-b]pyridine-4-amine*** **(2a)**

Yield 31%; brownish solid; mp 55–60 °C; ^1^H NMR (DMSO-*d*_6_) *δ* 11.41 (s, 1H, *N*H-1), 8.77 (s, 1H, *N*H), 7.96 (d, ^3^*J*_6/5_ = 5.4 Hz, 1H, H-6), 7.35 (t, ^3^*J*_5‘/6‘_ = 7.9, ^3^*J*_5‘/4‘_ = 7.9 Hz, 1H, H-5‘), 7.27 (t, ^4^*J*_2‘/6‘_ = 2.1, ^4^*J*_2‘/4‘_ = 2.1 Hz, 1H, H-2‘), 7.25 (dd, ^3^*J*_6‘/5‘_ = 7.9, ^4^*J*_6‘/2‘_ = 2.0 Hz, 1H, H-6‘), 7.24 (dd, ^3^*J*_2/3_ = 3.5, ^3^*J*_2/*N*H-1_ = 2.4 Hz, 1H, H-2), 7.02 (dd, ^3^*J*_4‘/5‘_ = 7.9, ^4^*J*_4‘/2‘_ = 2.0 Hz, 1H, H-4‘), 6.75 (d, ^3^*J*_5/6_ = 5.4 Hz, 1H, H-5), 6.55 (dd, ^3^*J*_3/2_ = 3.5, ^4^*J*_3/*N*H-1_ = 1.9 Hz, 1H, H-3); ^13^C NMR (DMSO-*d*_6_) *δ* 152.78, 147.62, 137.61, 136.99, 129.88, 124.42, 120.72, 116.00, 113.68, 111.90, 111.68; HRMS (ESI) *m*/*z* (%) = calculated for C_13_H_11_ClN_3_ [M+H]^+^: 244.0636; found: 244.0635.

***Data for N-(3-nitrophenyl)-1H-pyrrolo[2*** *,**3-b]pyridine-4-amine*** **(2b)**

Yield 23%; brown-redish solid; mp 232–234 °C; ^1^H NMR (DMSO-*d*_6_) *δ* 11.49 (s, 1H, *N*H**-**1), 9.10 (s, 1H, *N*H), 8.05 (t, ^3^*J*_2‘/6‘_ = 2.3 Hz, ^3^*J*_2‘/4‘_ = 2,3Hz, 1H, H-2‘), 8.02 (d, ^3^*J*_6/5_ = 5.4 Hz, 1H, H-6), 7.79 (ddd, ^3^*J*_6‘/5‘_ = 8.1, ^4^*J*_6‘/2‘_ = 2.3, ^4^*J*_6‘/4‘_ = 0.9 Hz, 1H, H-6‘), 7.71 (ddd, ^3^*J*_4‘/5‘_ = 8.1, ^4^*J*_4‘/2‘_ = 2.3, ^4^*J*_4‘/6‘_ = 0.9 Hz, 1H, H-4‘), 7.59 (t, ^3^*J*_5‘/6‘_ = 8.1 Hz, ^3^*J*_5‘4‘_ = 8.1 Hz, 1H, H-5‘), 7.28 (dd, ^3^*J*_2/3_ = 3.5, ^3^*J*_2/*N*H-1_ = 2.3 Hz, 1H, H-2), 6.85 (d, ^3^*J*_5/6_ = 5.4 Hz, 1H, H-5), 6.55 (dd, ^3^*J*_3/2_ = 3.5, ^3^*J*_3/*N*H-1_ = 1.8 Hz, 1H, H-3); ^13^C NMR (DMSO-*d*_6_) δ 150.38, 148.93, 144.26, 143.57, 142.09, 130.87, 125.15, 123.53, 115.92, 113.12, 110.12, 100.63, 98.56; HRMS (ESI) *m*/*z* (%) = calculated for C_13_H_11_N_4_O_2_ [M+H]^+^: 255.0877; found: 255.0874.

***Data for N-(3-chloro-4-methylphenyl)-1H-pyrrolo[2*** *,**3-b]pyridine-4-amine*** **(2c)**

Yield 18%; red-brownish solid; mp 189–191 °C; ^1^H NMR (DMSO-*d*_6_) *δ* 11.36 (s, 1H, *N*H-1), 8.64 (s, 1H, *N*H), 7.92 (d, ^3^*J*_6/5_ = 5.5 Hz, 1H, H-6), 7.30 (d, ^3^*J*_5‘/6‘_ = 8.2 Hz, 1H, H-5‘), 7.29 (d, ^4^*J*_2‘/6‘_ = 2.3 Hz, 1H, H-2‘), 7.21 (dd, ^3^*J*_2/3_ = 3.5, ^3^*J*_2/*N*H-1_ = 2.1 Hz, 1H, H-2), 7.18 (dd, ^3^*J*_6‘/5‘_ = 8.2, ^4^*J*_6‘/2‘_ = 2.3 Hz, 1H, H-6‘), 6.67 (d, ^3^*J*_5/6_ = 5.5 Hz, 1H, H-5), 6.55 (dd, ^3^*J*_3/2_ = 3.5, ^4^*J*_3/*N*H-1_ = 1.7 Hz, 1H, H-3), 2.30 (s, 3H, CH_3_-4‘); ^13^C NMR (DMSO-*d*_6_) *δ* 149.76, 143.86, 142.93, 140.64, 133.35, 131.51, 128.47, 122.37, 120.20, 118.90, 108.95, 98.85, 98.11, 18.84; HRMS (ESI) *m*/*z* (%) = calculated for C_14_H_13_ClN_3_ [M+H]^+^: 258.0793; found: 258.0791.

***Data for 5-((1H-pyrrolo[2*** *,**3-b]pyridine-4-yl)amino)-2-methylphenolate*** **(2d)**

Yield 15%; brownish solid; mp 214–216 °C; ^1^H NMR (DMSO-*d*_6_) *δ* 11.25 (s, 1H, *N*H-1), 9.28 (s, 1H, OH-1‘), 8.38 (s, 1H, *N*H), 7.85 (d, ^3^*J*_6/5_ = 5.3 Hz, 1H, H-6), 7.15 (dd, ^3^*J*_2/3_ = 3.6, ^3^*J*_2/*N*H-1_ = 1.6 Hz, 1H, H-2), 7.01 (d, ^3^*J*_3‘/4‘_ = 8.0 Hz, 1H, H-3‘), 6.78 (d, ^4^*J*_6‘/4‘_ = 2.2 Hz, 1H, H-6‘), 6.63 (dd, ^3^*J*_4‘/3‘_ = 8.0, ^4^*J*_4‘/6‘_ = 2.2 Hz, 1H, H-4‘), 6.62–6.58 (m, 1H, H-3), 6.60 (d, ^3^*J*_5/6_ = 5.3 Hz, 1H, H-5), 2.09 (s, 3H, CH_3_-2‘); ^13^C NMR (DMSO-*d*_6_) *δ* 155.69, 149.59, 144.10, 143.74, 139.60, 130.69, 121.73, 118.18, 111.97, 108.52, 107.79, 98.28, 98.18, 15.49; HRMS (ESI) *m*/*z* (%) = calculated for C_14_H_14_N_3_O [M+H]^+^: 240.1131; found: 240.1128.

**Formation of the 4-chloro-9*H*-pyrido[2**,**3-*b*]indole (4)**

One equivalent of 9*H*-pyrido[2,3-*b*]indole **3** was dissolved in acetic acid, followed by the dropwise addition of 1.2 equivalents of aqueous hydrogen peroxide solution (35%). The mixture was stirred at 115 °C under reflux. After 4 h, 0.3 equivalents of hydrogen peroxide (35%) were added and continuously heated under reflux for 2 h. The solution was then concentrated, followed by the addition of a saturated potassium carbonate solution, and stirred overnight at room temperature. The solid was filtered off, washed with water, dried and subsequently dissolved in water-free DMF (100 mL) under inert atmosphere. The solution was cooled down to 0 °C, before 2.4 equivalents of phosphorus oxychloride were added and stirred at room temperature for 24 h. The mixture was poured into 250 mL of water under cooling, alkalized with a potassium hydroxide solution (10%) to pH 9 and stirred at 0 °C for 15 min. The residual solid was filtered off, washed with water, dried, and then purified over silica gel with an eluent mixture of cyclohexane and ethyl acetate (80:20).

***Data for 4-chloro-9H-pyrido[2*** *,**3-b]indole*** **(4)**

Yield 76%; colourless solid; mp 232–234 °C; ^1^H NMR (DMSO-*d*_6_) *δ* 12.18 (s, 1H, *N*H-9), 8.38 (d, ^3^*J*_2/3_ = 5.3 Hz, 1H, H-2), 8.34 (dt, ^3^*J*_5/6_ = 7.8, ^4^*J*_5/7_ = 1.1 Hz, 1H, H-5), 7.57 (dt, ^3^*J*_8/7_ = 8.2, ^4^*J*_8/6_ = 1.1 Hz, 1H, H-8), 7.53 (ddd, ^3^*J*_6/5_ = 7.8, ^3^*J*_6/7_ = 6.5, ^4^*J*_6/8_ = 1.1 Hz, 1H, H-6), 7.32 (d, ^3^*J*_3/2_ = 5.3 Hz, 1H, H-3), 7.31 (ddd, ^3^*J*_7/8_ = 8.2, ^3^*J*_7/6_ = 6.5, ^4^*J*_7/5_ = 1.1 Hz, 1H, H-7); MS (ESI) *m*/*z* (%) = found: 204,3 (100, [M+H]^+^).

**Formation of the 6-bromo-4-chloro-9*H*-pyrido[2**,**3-*b*]indole (5)**

One equivalent of compound **4** was dissolved in acetic acid and cooled down to −5 °C. Thus, 1.6 equivalents of bromine were added, and the resulting solution was stirred at room temperature for 24 h. A 1 M sodium thiosulfate solution (10 mL) was added and stirred continuously until the solution turned clear, followed by the addition of an aqueous ammonia solution to achieve pH 10. The mixture was extracted with a mixture of ethyl acetate and chloroform (1:1) (20 mL) four times. The unified organic layers were dried over sodium sulfate and filtered. The solvent was removed under reduced pressure, and the crude product of compound **5** was purified over silica gel using an eluent mixture of ethyl acetate and cyclohexane (1:1).

***Data for 6-bromo-4-chloro-9H-pyrido[2*** *,**3-b]indole*** **(5)**

Yield 67%; colourless solid; mp 202–205 °C; ^1^H NMR (DMSO-*d*_6_) *δ* 12.39 (s, 1H, *N*H-9), 8.43 (d, ^3^*J*_2/3_ = 5.2 Hz, 1H, H-2), 8.43 (d, ^4^*J*_5/7_ = 2.0 Hz, 1H, H-5), 7.68 (dd, ^3^*J*_7/8_ = 8.6, ^4^*J*_7/5_ = 2.0 Hz, 1H, H-7), 7.54 (d, ^3^*J*_8/7_ = 8.6 Hz, 1H, H-8), 7.37 (d, ^3^*J*_3/2_ = 5.2 Hz, 1H, H-3); HRMS (ESI) *m*/*z* (%) = calculated for C_11_H_7_BrClN_2_ [M+H]^+^: 280.9476; found: 280.9478.

**Formation of the 4-aniline-substituted 6-bromo-9*H*-pyrido[2**,**3-*b*]indoles (6a**–**m)**

The procedure was similar to compounds **2a**–**d**, except the volumes of ethyl acetate used for dilution after the reactions and the final extraction volumes were 30 mL each. The unified organic layers were washed with 30 mL of water, dried over sodium sulfate and filtered. The solvent was removed under reduced pressure, and the crude product of compound **6** was purified over silica gel using ethyl acetate and cyclohexane as eluent mixture (1:1).

***Data for 6-bromo-N-(3-chlorophenyl)-9H-pyrido[2*** *,**3-b]indol-4-amine*** **(6a)**

Yield 44%; colourless solid; mp 248–252 °C; ^1^H NMR (DMSO-*d*_6_) *δ* 11.87 (s, 1H, *N*H-9), 8.76 (s, 1H, *N*H), 8.31 (d, ^4^*J*_5/7_ = 1.9 Hz, 1H, H-5), 8.16 (d, ^3^*J*_2/3_ = 5.6 Hz, 1H, H-2), 7.51 (dd, ^3^*J*_7/8_ = 8.6, ^4^*J*_7/5_ = 1.9 Hz, 1H, H-7), 7.41 (d, ^3^*J*_8/7_ = 8.6 Hz, 1H, H-8), 7.38 (t, ^3^*J*_5‘/6‘_ = 8.0 Hz, ^3^*J*_5‘/4‘_ = 8.0 Hz, 1H, H-5‘), 7.34 (t, ^4^*J*_2‘/6‘_ = 2.1 Hz, ^4^*J*_2‘/4‘_ = 2.1 Hz, 1H, H-2‘), 7.27 (ddd, ^3^*J*_6‘/5‘_ = 8.0, ^4^*J*_6‘/2‘_ = 2.1, ^4^*J*_6‘/4‘_ = 1.0 Hz, 1H, H-6‘jh), 7.12 (ddd, ^3^*J*_4‘/5‘_ = 8.0, ^4^*J*_4‘/2‘_ = 2.1, ^4^*J*_4‘/6‘_ = 1.0 Hz, 1H, H-4‘), 6.86 (d, ^3^*J*_3/2_ = 5.6 Hz, 1H, H-3); ^13^C NMR (DMSO-*d*_6_) *δ* 153.95, 147.71, 145.72, 142.90, 136.47, 133.56, 130.77, 127.48, 124.77, 122.34, 121.62, 120.50, 119.29, 112.44, 111.14, 103.01, 102.38; HRMS (ESI) *m/z* (%) = calculated for C_17_H_12_BrClN_3_ [M+H]^+^: 371.9898; found: 371.9902.

***Data for 6-bromo-N-(3-chloro-4-methylphenyl)-9H-pyrido[2*** *,**3-b]indol-4-amine*** **(6b)**

Yield 27%; brownish solid; mp 269–270 °C; ^1^H NMR (DMSO-*d*_6_) *δ* 11.85 (s, 1H, *N*H-9), 8.63 (s, 1H, *N*H), 8.39 (d, ^4^*J*_5/7_ = 1.9 Hz, 1H, H-5), 8.12 (d, ^3^*J*_2/3_ = 5.7 Hz, 1H, H-2), 7.51 (dd, ^3^*J*_7/8_ = 8.6, ^4^*J*_7/5_ = 1.9 Hz, 1H, H-7), 7.41 (d, ^3^*J*_8/7_ = 8.6 Hz, 1H, H-8), 7.37 (d, ^4^*J*_2‘/6‘_ = 2.3 Hz, 1H, H-2‘), 7.35 (d, ^3^*J*_5‘/6‘_ = 8.2 Hz, 1H, H-5‘), 7.22 (dd, ^3^*J*_6‘/5‘_ = 8.2, ^4^*J*_6‘/2‘_ = 2.3 Hz, 1H, H-6‘), 6.75 (d, ^3^*J*_3/2_ = 5.7 Hz, 1H, H-3), 2.33 (s, 3H, CH_3_-4‘); ^13^C NMR (DMSO-*d*_6_) δ 153.90, 147.65, 146.40, 140.17, 136.35, 133.47, 131.56, 129.86, 127.29, 124.57, 122.07, 121.73, 120.62, 112.35, 111.15, 102.29, 101.46, 18.95; HRMS (ESI) *m*/*z* (%) = calculated for C_18_H_14_BrClN_3_ [M+H]^+^: 386.0054; found: 386.0055.

***Data for 6-bromo-N-(3*** *,**4-dichlorophenyl)-9H-pyrido[2**,**3-b]indol-4-amine*** **(6c)**

Yield 37%; greyish solid; mp 254–257 °C; ^1^H NMR (DMSO-*d*_6_) *δ* 11.84 (br, 1H, *N*H-9), 8.62 (br, 1H, *N*H), 8.15 (d, ^4^*J*_5/7_ = 1.9 Hz, 1H, H-5), 8.12 (d, ^3^*J*_2/3_ = 5.8 Hz, 1H, H-2h-2), 7.52 (dd, ^3^*J*_7/8_ = 8.6, ^4^*J*_7/5_ = 1.9 Hz, 1H, H-7), 7.50 (d, ^3^*J*_5‘/6‘_ = 8.7 Hz, 1H, H-5‘), 7.46 (d, ^4^*J*_2‘/6‘_ = 2.6 Hz, 1H, H-2‘), 7.43 (d, ^3^*J*_8/7_ = 8.6 Hz, 1H, H-8), 7.24 (dd, ^3^*J*_6‘/5‘_ = 8.7, ^4^*J*_6‘/2‘_ = 2.6 Hz, 1H, H-6‘), 6.89 (d, ^3^*J*_3/2_ = 5.8 Hz, 1H, H-3); ^13^C NMR (DMSO-*d*_6_) *δ* 153.95, 147.78, 145.28, 141.69, 136.53, 131.45, 130.93, 127.59, 124.75, 123.79, 122.01, 121.50, 120.61, 112.50, 111.16, 103.22, 102.60; HRMS (ESI) *m/z* (%) = calculated for C_17_H_11_BrCl_2_N_3_ [M+H]^+^: 405.9508; found: 405.9513.

***Data for 6-bromo-N-(3-nitrophenyl)-9H-pyrido[2*** *,**3-b]indol-4-amine*** **(6d)**

Yield 73%; green-brownish solid; mp 289–293 °C; ^1^H NMR (DMSO-*d*_6_) *δ* 11.95 (s, 1H, *N*H-9), 9.09 (s, 1H, *N*H), 8.33 (d, ^4^*J*_5/7_ = 1.9 Hz, 1H, H-5), 8.22 (d, ^3^*J*_2/3_ = 5.6 Hz, 1H, H-2), 8.12 (dd, ^4^*J*_2‘/6‘_ = 2.2, ^4^*J*_2‘/4‘_ = 2.2 Hz, 1H, H-2‘), 7.88 (ddd, ^3^*J*_6‘/5‘_ = 8.2, ^4^*J*_2‘/6‘_ = 2.2, ^4^*J*_6‘/4‘_ = 1.0 Hz, 1H, H-6‘), 7.75 (ddd, ^3^*J*_4‘/5‘_ = 8.2, ^4^*J*_4‘/2‘_ = 2.2, ^4^*J*_4‘/6‘_ = 1.0 Hz, 1H, H-4‘), 7.63 (dd, ^3^*J*_5‘/6‘_ = 8.2, ^3^*J*_5‘/4‘_ = 8.2 Hz, 1H, H-5‘), 7.54 (dd, ^3^*J*_7/8_ = 8.6, ^4^*J*_7/5_ = 1.9 Hz, 1H, H-7), 7.43 (d, ^3^*J*_8/7_ = 8.6 Hz, 1H, H-8), 6.96 (d, ^3^*J*_3/2_ = 5.6 Hz, 1H, H-3); ^13^C NMR (DMSO-*d*_6_) *δ* 153.88, 148.44, 147.69, 144.89, 142.77, 136.47, 130.37, 127.56, 125.93, 124.71, 121.33, 116.41, 114.09, 112.42, 111.03, 103.41, 102.59; HRMS (ESI) *m*/*z* (%) = calculated for C_17_H_12_BrN_4_O_2_ [M+H]^+^: 383.0138; found: 383.0140.

***Data for 6-bromo-N-(4-methyl-3-nitrophenyl)-9H-pyrido[2*** *,**3-b]indol-4-amine*** **(6e)**

Yield 67%; yellow-brownish solid; mp 288–293 °C; ^1^H NMR (DMSO-*d*_6_) *δ* 11.89 (s, 1H, *N*H-9), 8.89 (s, 1H, *N*H), 8.36 (d, ^4^*J*_5/7_ = 1.9 Hz, 1H, H-5), 8.16 (d, ^3^*J*_2/3_ = 5.7 Hz, 1H, H-2), 7.91 (d, ^4^*J*_2‘/6‘_ = 2.4 Hz, 1H, H-2‘), 7.58 (dd, *J* = 8.3, 2.4 Hz, 1H, H-6‘), 7.52 (dd, *J* = 8.6, 1.9 Hz, 1H, H-7), 7.48 (d, *J* = 8.3 Hz, 1H, H-5‘), 7.42 (d, *J* = 8.6 Hz, 1H, H-8), 6.84 (d, *J* = 5.7 Hz, 1H, H-3), 2.48 (s, 3H, CH_3_-4‘); ^13^C NMR (DMSO-*d*_6_) *δ* 153.88, 149.17, 147.69, 145.66, 140.27, 136.48, 133.45, 127.54, 126.49, 125.77, 124.67, 121.57, 116.37, 112.49, 111.19, 102.87, 101.86, 19.09; HRMS (ESI) *m*/*z* (%) = calculated for C_18_H_14_BrN_4_O_2_ [M+H]^+^: 397.0295; found: 397.0299.

***Data for 6-bromo-N-(4-methyl-3-nitrophenyl)-9H-pyrido[2*** *,**3-b]indol-4-amine*** **(6f)**

Yield 15%; orange-red solid; mp 256–263 °C; ^1^H NMR (DMSO-*d*_6_) *δ* 11.93 (s, 1H, *N*H-9), 8.92 (s, 1H, *N*H), 8.40 (d, ^4^*J*_5/7_ = 1.9 Hz, 1H, H-5), 8.19 (d, ^3^*J*_2/3_ = 5.6 Hz, 1H, H-2), 8.04 (dd, *J* = 6.5, 2.8 Hz, 1H, H-2‘), 7.75 (dt, *J* = 8.9, 4.1 Hz, 1H, H-6‘), 7.59 (dd, *J* = 11.1, 9.0 Hz, 1H, H-5‘), 7.53 (dd, ^3^*J*_7/8_ = 8.6, ^4^*J*_7/5_ = 1.9 Hz, 1H, H-7), 7.44 (d, ^3^*J*_8/7_ = 8.6 Hz, 1H, H-8), 6.86 (d, ^3^*J*_3/2_ = 5.6 Hz, 1H, H-3); ^13^C NMR (DMSO-*d*_6_) *δ* 153.82, 151.02, 149.31, 147.71, 145.62, 138.15, 136.51, 127.63, 124.64, 121.50, 119.23, 119.09, 117.66, 112.56, 111.23, 102.90, 101.73; HRMS (ESI) *m*/*z* (%) = calculated for C_17_H_11_BrFN_4_O_2_ [M+H]^+^: 401.0044; found: 401.0049.

***Data for N*** **^1^** ***-(6-bromo-9H-pyrido[2*** *,**3-b]indol-4-yl)-3-nitrobenzene-1**,**4-diamine*** **(6g)**

Yield 56%; dark red solid; mp 287–291 °C; ^1^H NMR (DMSO-*d*_6_) *δ* 11.77 (s, 1H, *N*H-9), 8.54 (d, ^4^*J*_5/7_ = 1.9 Hz, 1H, H-5), 8.42 (s, 1H, *NH*), 8.05 (d, ^3^*J*_2/3_ = 5.6 Hz, 1H, H-2), 7.91 (d, ^4^*J*_2‘/6‘_ = 2.8 Hz, 1H, H-2‘), 7.49 (dd, ^3^*J*_7/8_ = 8.5, ^4^*J*_7/5_ = 1.9 Hz, 1H, H-7), 7.49 (dd, ^3^*J*_6‘/5‘_ = 8.4, ^4^*J*_6‘/2‘_ = 2.8 Hz, 1H, H-6‘), 7.47 (s, 2H, *N*H_2_), 7.40 (d, ^3^*J*_8/7_ = 8.5 Hz, 1H, H-8), 7.11 (d, ^3^*J*_5‘/6‘_ = 8.4 Hz, 1H, H-5‘), 6.48 (d, ^3^*J*_3/2_ = 5.6 Hz, 1H, H-3); ^13^C NMR (DMSO-*d*_6_) *δ* 153.82, 147.69, 147.60, 143.84, 136.16, 133.66, 129.68, 128.57, 127.00, 124.23, 121.91, 120.07, 119.23, 112.23, 111.18, 101.12, 100.12; HRMS (ESI) *m*/*z* (%) = calculated for C_17_H_13_BrN_5_O_2_ [M+H]^+^: 398.0247; found: 398.0254.

***Data for 4-((6-bromo-9H-pyrido[2*** *,**3-b]indol-4-yl)amino)-2-nitrophenol*** **(6h)**

Yield 34%; dark red solid; mp 268–271 °C; ^1^H NMR (DMSO-*d*_6_) *δ* 11.81 (s, 1H, *N*H-9), 8.54 (s, 1H, *N*H), 8.50 (d, ^4^*J*_5/7_ = 1.9 Hz, 1H, H-5), 8.08 (d, ^3^*J*_2/3_ = 5.6 Hz, 1H, H-2), 7.84 (d, ^4^*J*_3‘/6‘_ = 2.7 Hz, 1H, H-3‘), 7.57 (dd, ^3^*J*_6‘/5‘_ = 8.9, ^4^*J*_6‘/3‘_ = 2.7 Hz, 1H, H-6‘), 7.50 (dd, ^3^*J*_7/8_ = 8.6, ^4^*J*_7/5_ = 1.9 Hz, 1H, H-7), 7.40 (d, ^3^*J*_8/7_ = 8.6 Hz, 1H, H-8), 7.19 (d, ^3^*J*_5‘/6‘_ = 8.9 Hz, 1H, H-5‘), 6.58 (d, ^3^*J*_3/2_ = 5.6 Hz, 1H, H-3); ^13^C NMR (DMSO-*d*_6_) *δ* 153.84, 148.81, 147.74, 147.06, 136.45, 136.25, 132.25, 131.07, 127.18, 124.33, 121.79, 119.89, 119.16, 112.33, 111.19, 101.57, 100.37; HRMS (ESI) *m*/*z* (%) = calculated for C_17_H_12_BrN_4_O_3_ [M+H]^+^: 399.0087; found: 399.0090.

***Data for 6-bromo-N-(3-fluorophenyl)-9H-pyrido[2*** *,**3-b]indol-4-amine*** **(6i)**

Yield 64%; dark greyish solid; mp 263–266 °C; ^1^H NMR (DMSO-*d*_6_) *δ* 11.88 (s, 1H, *N*H-9), 8.80 (s, 1H, *N*H), 8.29 (d, ^4^*J*_5/7_ = 1.9 Hz, 1H, H-5), 8.17 (d, ^3^*J*_2/3_ = 5.6 Hz, 1H, H-2), 7.51 (dd, ^3^*J*_7/8_ = 8.7, ^4^*J*_7/5_ = 1.9 Hz, 1H, H-7), 7.42 (d, ^3^*J*_8/7_ = 8.7 Hz, 1H, H-8), 7.39 (ddd, ^3^*J*_5‘/4‘_ = 8.2, ^3^*J*_5‘/6‘_ = 8.2, ^4^*J*_5‘/3‘(F)_ = 6.9 Hz, 1H, H-5‘), 7.15 (ddd, ^3^*J*_6‘/5‘_ = 8.2, ^4^*J*_6‘/2‘_ = 2.3, ^4^*J*_6‘/4‘_ = 0.9 Hz, 1H, H-6‘), 7.09 (ddd, ^3^*J*_2‘/3‘(F)_ = 11.2, ^4^*J*_2‘4‘_ = 2.3 Hz, ^4^*J*_2‘6‘_ = 2.3 Hz, 1H, H-2‘), 6.89 (dddd, ^3^*J*_4‘/3‘(F)_ = 8.75, ^3^*J*_4‘/5‘_ = 8.2, ^4^*J*_4‘/2‘_ = 2.3, ^4^*J*_4‘/6‘_ = 0.9 Hz, 1H, H-4‘), 6.88 (d, ^3^*J*_3/2_ = 5.6 Hz, 1H, H-3); ^13^C NMR (DMSO-*d*_6_) *δ* 163.56, 161.95, 153.98, 147.73, 145.73, 143.30 (d, *J* = 10.6 Hz), 136.48, 130.75 (d, *J* = 9.9 Hz), 127.45, 124.79, 121.63, 116.57 (d, *J* = 2.7 Hz), 112.42, 111.09, 109.03 (d, *J* = 21.2 Hz), 107.44 (d, *J* = 24.1 Hz), 103.09, 102.61; HRMS (ESI) *m*/*z* (%) = calculated for C_17_H_12_BrFN_3_ [M+H]^+^: 356.0193; found: 356.0198.

***Data for 6-bromo-N-(3-(trifluoromethyl)phenyl)-9H-pyrido[2*** *,**3-b]indol-4-amine*** **(6j)**

Yield 23%; grey brownish solid; mp 256–258 °C; ^1^H NMR (DMSO-*d*_6_) *δ* 11.90 (s, 1H, *N*H-9), 8.91 (s, 1H, *N*H), 8.30 (d, ^4^*J*_5/7_ = 2.0 Hz, 1H, H-5), 8.18 (d, ^3^*J*_2/3_ = 5.6 Hz, 1H, H-2), 7.63–7.56 (m, 3H, H-4‘, H-5‘, H-6‘), 7.52 (dd, ^3^*J*_7/8_ = 8.6, ^4^*J*_7/5_ = 2.0 Hz, 1H, H-7), 7.43 (d, ^3^*J*_8/7_ = 8.6 Hz, 1H, H-8), 7.40 (m, 1H, H-2‘), 6.86 (d, ^3^*J*_3/2_ = 5.6 Hz, 1H, H-3); HRMS (ESI) *m*/*z* (%) = calculated for C_18_H_12_BrF_3_N_3_ [M+H]^+^: 406.0161; found: 406.0161.

***Data for 6-bromo-N-(3-methoxyphenyl)-9H-pyrido[2*** *,**3-b]indol-4-amine*** **(6k)**

Yield 35%; greyish solid; mp 246–247 °C; ^1^H NMR (DMSO-*d*_6_) *δ* 11.81 (s, 1H, *N*H-9), 8.60 (s, 1H, *N*H), 8.36 (d, ^4^*J*_5/7_ = 1.9 Hz, 1H, H-5), 8.11 (d, ^3^*J*_2/3_ = 5.6 Hz, 1H, H-2), 7.50 (dd, ^3^*J*_7/8_ = 8.6, ^4^*J*_7/5_ = 1.9 Hz, 1H, H-7), 7.40 (d, ^3^*J*_8/7_ = 8.6 Hz, 1H, H-8), 7.29 (t, ^3^*J*_5‘/6‘_ = 8.0, ^3^*J*_5‘/4‘_ = 8.0 Hz, 1H, H-5‘), 6.91 (ddd, ^3^*J*_6‘/5‘_ = 8.0, ^4^*J*_6‘/2‘_ = 2.4, ^4^*J*_6‘/4‘_ = 1.0 Hz, 1H, H-6‘), 6.89 (t, ^4^*J*_2‘/6‘_ = 2.2, ^4^*J*_2‘/4‘_ = 2.2 Hz, 1H, H-2‘), 6.81 (d, ^3^*J*_3/2_ = 5.6 Hz, 1H, H-3), 6.69 (ddd, ^3^*J*_4‘/5‘_ = 8.0, ^4^*J*_4‘/2‘_ = 2.4, ^4^*J*_4‘/6‘_ = 1.0 Hz, 1H, H-4‘), 3.76 (s, 3H, OCH_3_-3‘); HRMS (ESI) *m*/*z* (%) = calculated for C_18_H_15_BrN_3_O [M+H]^+^: 368.0393; found: 368.0395.

***Data for 3-((6-bromo-9H-pyrido[2*** *,**3-b]indol-4-yl)amino)phenol*** **(6l)**

Yield 33%; greyish solid; mp 248–252 °C; ^1^H NMR (DMSO-*d*_6_) *δ* 12.02 (s, 1H, *N*H-9), 9.51 (s, 1H, OH-1‘), 8.78 (s, 1H, *N*H), 8.44 (d, ^4^*J*_5/7_ = 1.9 Hz, 1H, H-5), 8.10 (d, ^3^*J*_2/3_ = 5.9 Hz, 1H, H-2), 7.53 (dd, ^3^*J*_7/8_ = 8.6, ^4^*J*_7/5_ = 1.9 Hz, 1H, H-7), 7.45 (d, ^3^*J*_8/7_ = 8.6 Hz, 1H, H-8), 7.20 (t, ^3^*J*_5‘/6‘_ = 7.9 Hz, ^3^*J*_5‘/4‘_ = 7.9 Hz, 1H, H-5‘), 6.80–6.73 (m, 2H, H-2‘, H-4‘), 6.76 (d, ^3^*J*_3/2_ = 5.9 Hz, 1H, H-3), 6.59 (dd, ^3^*J*_6‘/5‘_ = 7.9, ^4^*J*_6‘/2‘_ = 2.1 Hz, 1H, H-6‘); HRMS (ESI) *m*/*z* (%) = calculated for C_17_H_13_BrN_3_O [M+H]^+^: 354.0237; found: 354.0237.

***Data for 5-((6-bromo-9H-pyrido[2*** *,**3-b]indol-4-yl)amino)-2-methoxyphenol*** **(6m)**

Yield 42%; dark brownish solid; mp 269–273 °C; ^1^H NMR (DMSO-*d*_6_) *δ* 12.00 (br, 1H, *N*H-9), 9.65 (br, 1H, OH-1‘), 8.91 (br, 1H, *N*H), 8.23 (d, ^4^*J*_5/7_ = 1.8 Hz, 1H, H-5-5), 7.95 (d, ^3^*J*_2/3_ = 6.0 Hz, 1H, H-2), 7.47 (dd, ^3^*J*_7/8_ = 8.6, ^4^*J*_7/5_ = 1.8 Hz, 1H, H-7), 7.40 (d, ^3^*J*_8/7_ = 8.6 Hz, 1H, H-8), 6.97 (d, ^3^*J*_3‘/4‘_ = 8.5 Hz, 1H, H-3‘), 6.84 (d, ^4^*J*_6‘/4‘_ = 2.6 Hz, 1H, H-6‘), 6.80 (dd, ^3^*J*_4‘/3‘_ = 8.5, ^4^*J*_4‘/6‘_ = 2.6 Hz, 1H, H-4‘), 6.62 (d, ^3^*J*_3/2_ = 6.0 Hz, 1H, H-3), 3.89 (s, 3H, OCH_3_-2‘); ^13^C NMR (DMSO-*d*_6_) *δ* 153.86, 148.06, 147.47, 147.06, 144.84, 136.09, 133.64, 126.82, 122.07, 114.61, 112.90, 111.98, 111.11, 101.04, 100.49, 55.97, 30.68; HRMS (ESI) *m/z* (%) = calculated for C_18_H_15_BrN_3_O_2_ [M+H]^+^: 384.0342; found: 384.0345.

**Formation of the 4-chloro-6-nitro-9*H*-pyrido[2**,**3-*b*]indole (7)**

To water-free nitric acid (1.2 mL), one equivalent of compound **3** was added at −5 °C and stirred vigorously. The mixture was then stirred at room temperature for 20 min, poured on ice, diluted with water (20 mL) and then alkalized to pH 10 with a saturated potassium carbonate solution. The yellow solid was filtered off, washed with water, dried and purified over silica gel with an eluent mixture of dichloromethane and ethyl acetate (80:20).

***Data for 4-chloro-6-nitro-9H-pyrido[2****,**3-b]indole*** **(7)**

Yield 49%; yellow solid; mp > 320 °C; ^1^H NMR (DMSO-*d*_6_) 12.87 (s, 1H, *N*H-9), 9.00 (d, *J* = 2.3 Hz, 1H, H-5), 8.46 (d, *J* = 5.3 Hz, 1H, H-2), 8.35 (dd, *J* = 9.0, 2.3 Hz, 1H, H-7), 7.65 (d, *J* = 9.0 Hz, 1H, H-8), 7.41 (d, *J* = 5.3 Hz, 1H, H-3); MS (ESI) *m*/*z* (%) = found: 248.02 (100, [M+H]^+^).

**Formation of the 4-aniline-substituted 6-nitro-9*H*-pyrido[2**,**3-*b*]indoles (8a–e)**

The procedure was similar to compounds **2a**–**d** except the volume of ethyl acetate used for dilution after the reactions was 50 mL. The remaining ethyl acetate was removed under reduced pressure, and the remaining crude product **8** was purified over silica gel using cyclohexane, acetonitril and ethyl acetate as eluent mixture (2:1:1).

***Data for N-(3-chlorophenyl)-6-nitro-9H-pyrido[2*** *,**3-b]indol-4-amine*** **(8a)**

Yield 19%; yellow brownish solid; mp 308–312 °C; ^1^H NMR (CD_3_OD) *δ* 12.35 (br, 1H, *N*H-9), 9.15 (br, 1H, *N*H), 9.00 (d, ^4^*J*_5/7_ = 2.2 Hz, 1H, H-5), 8.37 (dd, ^3^*J*_7/8_ = 8.9, ^4^*J*_7/5_ = 2.2 Hz, 1H, H-7), 8.17 (d, ^3^*J*_2/3_ = 6.1 Hz, 1H, H-2), 7.65 (d, ^3^*J*_8/7_ = 8.9 Hz, 1H, H-8), 7.41 (t, ^3^*J*_5‘/6‘_ = 8.1, ^3^*J*_5‘/4‘_ = 8.1 Hz, 1H, H-5‘), 7.38 (t, ^4^*J*_2‘/6‘_ = 2.1, ^4^*J*_2‘/4‘_ = 2.1 Hz, 1H, H-2‘), 7.30 (ddd, ^3^*J*_6‘/5‘_ = 8.1, ^4^*J*_6‘/2‘_ = 2.1, ^4^*J*_6‘/4‘_ = 1.0 Hz, 1H, H-6‘), 7.21 (ddd, ^3^*J*_4‘/5‘_ = 8.1, ^4^*J*_4‘/2‘_ = 2.1, ^4^*J*_4‘/6‘_ = 1.0 Hz, 1H, H-4‘), 6.95 (d, ^3^*J*_3/2_ = 6.1 Hz, 1H, H-3); HRMS (ESI) *m*/*z* (%) = calculated for C_17_H_12_ClN_4_O_2_ [M+H]^+^: 339.0643; found: 339.0647.

***Data for N-(3-chloro-4-methylphenyl)-6-nitro-9H-pyrido[2*** *,**3-b]indol-4-amine*** **(8b)**

Yield 42%; yellow brownish solid; mp 323–327 °C; ^1^H NMR (DMSO-*d*_6_) *δ* 12.46 (s, 1H, *N*H-9), 9.10 (d, ^4^*J*_5/7_ = 2.3 Hz, 1H, H-5), 8.99 (s, 1H, *N*H), 8.29 (dd, ^3^*J*_7/8_ = 9.0, ^4^*J*_7/5_ = 2.3 Hz, 1H, H-7), 8.20 (d, ^3^*J*_2/3_ = 5.7 Hz, 1H, H-2), 7.60 (d, ^3^*J*_8/7_ = 9.0 Hz, 1H, H-8), 7.39 (d, ^4^*J*_2‘/6‘_ = 2.3 Hz, 1H, H-2‘), 7.38 (d, ^3^*J*_5‘/6‘_ = 8.6 Hz, 1H, H-5‘), 7.23 (dd, ^3^*J*_6‘/5‘_ = 8.6, ^4^*J*_6‘/2‘_ = 2.3 Hz, 1H, H-6‘), 6.81 (d, ^3^*J*_3/2_ = 5.7 Hz, 1H, H-3), 2.34 (s, 3H, CH_3_-4‘); HRMS (ESI) *m*/*z* (%) = calculated for C_18_H_14_ClN_4_O_2_ [M+H]^+^: 353.0800; found: 353.0802.

***Data for N-(3-ethoxyphenyl)-6-nitro-9H-pyrido[2*** *,**3-b]indol-4-amine*** **(8c)**

Yield 41%; yellow brownish solid; mp 263–264 °C; ^1^H NMR (DMSO-*d*_6_) *δ* 12.43 (s, 1H, *N*H-9), 9.07 (d, ^4^*J*_5/7_ = 2.3 Hz, 1H, H-5), 8.95 (s, 1H, *N*H), 8.29 (dd, ^3^*J*_7/8_ = 8.9, ^4^*J*_7/5_ = 2.3 Hz, 1H, H-7), 8.19 (d, ^3^*J*_2/3_ = 5.5 Hz, 1H, H-2), 7.59 (d, ^3^*J*_8/7_ = 8.9 Hz, 1H, H-8), 7.29 (t, ^3^*J*_5‘/6‘_ = 8.1, ^3^*J*_5‘/4‘_ = 8.1 Hz, 1H, H-5‘), 6.90 (dd, ^3^*J*_6‘/5‘_ = 8.1, ^4^*J*_6‘/2‘_ = 2.2 Hz, 1H, H-6‘), 6.88 (t, ^4^*J*_2‘/6‘_ = 2.2, ^4^*J*_2‘/4‘_ = 2.2 Hz, 1H, H-2‘), 6.87 (d, ^3^*J*_3/2_ = 5.5 Hz, 1H, H-3), 6.71 (dd, ^3^*J*_4‘/5‘_ = 8.1, ^4^*J*_4‘/2‘_ = 2.2 Hz, 1H, H-4‘), 4.03 (q, *J* = 7.0 Hz, 2H, CH_2_), 1.32 (t, *J* = 7.0 Hz, 3H, CH_3_); ^13^C NMR (DMSO-*d*_6_) *δ* 159.39, 154.98, 148.35, 147.15, 141.96, 141.69, 140.13, 130.04, 120.68, 119.71, 119.08, 114.07, 110.56, 109.75, 108.07, 103.25, 103.01, 63.01, 14.63; HRMS (ESI) *m*/*z* (%) = calculated for C_19_H_17_N_4_O_3_ [M+H]^+^: 349.1295; found: 349.1297.

***Data for 3-((6-nitro-9H-pyrido[2*** *,**3-b]indol-4-yl)amino)phenol*** **(8d)**

Yield 35%; yellow solid; mp 322–328 °C; ^1^H NMR (DMSO-*d*_6_) *δ* 12.32 (br s, 1H, *N*H-9), 9.34 (br s, 1H, OH-1‘) 9.07 (d, ^4^*J*_5/7_ = 2.3 Hz, 1H, H-5), 8.97 (br s, 1H, *N*H), 8.94 (d, ^4^*J*_5/7_ = 2.2 Hz, 1H, H-5), 8.33 (dd, ^3^*J*_7/8_ = 9.0, ^4^*J*_7/5_ = 2.2 Hz, 1H, H-7), 8.11 (d, ^3^*J*_2/3_ = 5.9 Hz, 1H, H-2), 7.59 (d, ^4^*J*_8/7_ = 9.0 Hz, 1H, H-8), 7.22 (t, ^3^*J*_5‘/4‘_ = 8.0, ^3^*J*_5‘/6‘_ = 8.0 Hz, 1H, H-5‘), 6.91 (d, ^3^*J*_3/2_ = 5.9 Hz, 1H, H-3), 6.82 (ddd, ^3^*J*_4‘/5‘_ = 8.0, ^4^*J*_4‘/2‘_ = 2.2, ^4^*J*_4‘/6‘_ = 0.9 Hz, 1H, H-4‘), 6.79 (t, ^4^*J*_2‘/4‘_ = 2.2, ^4^*J*_2‘/6‘_ = 2.2 Hz, 1H, H-2‘), 6.64 (ddd, ^3^*J*_6‘/5‘_ = 8.0, ^4^*J*_6‘/2‘_ = 2.2, ^4^*J*_6‘/4‘_ = 0.9 Hz, 1H, H-6‘); ^13^C NMR (DMSO-*d*_6_) *δ* 158.21, 154.98, 148.30, 147.30, 141.81, 141.68, 140.14, 129.96, 120.63, 119.78, 119.14, 112.77, 110.81, 110.50, 108.97, 103.18, 103.07; HRMS (ESI) *m*/*z* (%) = calculated for C_17_H_13_N_4_O_3_ [M+H]^+^: 321.0982; found: 321.0985.

***Data for 6-nitro-N-(3-(trifluoromethyl)phenyl)-9H-pyrido[2*** *,**3-b]indol-4-amine*** **(8e)**

Yield 29%; yellow solid; mp 310–314 °C; ^1^H NMR (DMSO-*d*_6_) *δ* 12.52 (s, 1H, *N*H-9), 9.26 (s, 1H, *N*H), 9.00 (d, ^4^*J*_5/7_ = 2.3 Hz, 1H, H-5), 8.31 (dd, ^3^*J*_7/8_ = 8.9, ^4^*J*_7/5_ = 2.3 Hz, 1H, H-7), 8.27 (d, ^3^*J*_2/3_ = 5.7 Hz, 1H, H-2), 7.69–7.59 (m, 3H, H-6‘, H-5‘, H-2‘), 7.61 (d, ^3^*J*_8/7_ = 8.9 Hz, 1H, H-8), 7.44 (dd, ^3^*J*_4‘/5‘_ = 7.0, ^4^*J*_4‘/2‘_ = 3.9 Hz, 1H, H-4‘), 6.93 (d, ^3^*J*_3/2_ = 5.7 Hz, 1H, H-3); HRMS (ESI) *m*/*z* (%) = calculated for C_18_H_12_F_3_N_4_O_2_ [M+H]^+^: 373.0907; found: 373.0900.

**Formation of the 4-aniline-substituted 6-amino-9*H*-pyrido[2**,**3-*b*]indoles (9a–d)**

One equivalent of compound **8** was suspended in hydrochloric acid (10%) under argon atmosphere. Six equivalents of tin(II) chloride bishydrate were added and subsequently stirred under reflux for 75 min. After cooling to room temperature, water (20 mL) was added and then alkalized to pH 12 with a 10 M sodium hydroxide solution. The solution was extracted with ethyl acetate (20 mL) twice, and the unified organic layers were washed with water (20 mL). The solution was dried over sodium sulfate and filtered, and the solvent was removed under reduced pressure. The remaining solid of compound **9** was purified via column chromatography using silica gel and either dichloromethane and methanol (19:1) or cyclohexane, acetonitrile and ethyl acetate (2:1:1).

***Data for N*** **^4^** ***-(3-chlorophenyl)-9H-pyrido[2*** *,**3-b]indole-4**,**6-diamine*** **(9a)**

Yield 32%; greyish solid; mp 216–221 °C; ^1^H NMR (DMSO-*d*_6_) *δ* 11.20 (s, 1H, *N*H-9), 8.60 (s, 1H, *N*H), 8.07 (d, ^3^*J*_2/3_ = 5.5 Hz, 1H, H-2), 7.34 (d, ^4^*J*_5/7_ = 2.0 Hz, 1H, H-5), 7.32 (t, ^3^*J*_5‘/6‘_ = 8.0, ^3^*J*_5‘/4‘_ = 8.0 Hz, 1H, H-5‘), 7.31 (t, ^4^*J*_2‘/6‘_ = 2.2, ^4^*J*_2‘/4‘_ = 2.2 Hz, 1H, H-2‘), 7.23 (ddd, ^3^*J*_6‘/5‘_ = 8.0, ^4^*J*_6‘/2‘_ = 2.2, ^4^*J*_6‘/4‘_ = 1.0 Hz, 1H, H-6‘), 7.17 (d, ^3^*J*_8/7_ = 8.4 Hz, 1H, H-8), 7.01 (dd, ^3^*J*_7/8_ = 8.4, ^4^*J*_7/5_ = 2.0 Hz, 1H, H-7), 6.81 (d, ^3^*J*_3/2_ = 5.5 Hz, 1H, H-3), 6.77 (ddd, ^3^*J*_4‘/5‘_ = 8.0, ^4^*J*_4‘/2‘_ = 2.2, ^4^*J*_4‘/6‘_ = 1.0 Hz, 1H, H-4‘), 4.78 (s, 2H, *N*H_2_-6); ^13^C NMR (DMSO-*d*_6_) *δ* 153.95, 146.19, 144.80, 141.30, 133.53, 130.59, 120.93, 120.59, 118.56, 117.30, 114.53, 110.78, 107.27, 104.88, 102.24; HRMS (ESI) *m*/*z* (%) = calculated for C_17_H_14_ClN_4_ [M+H]^+^: 309.0902; found: 309.0904.

***Data for N*** **^4^** ***-(3-chloro-4-methylphenyl)-9H-pyrido[2*** *,**3-b]indole-4**,**6-diamine*** **(9b)**

Yield 34%; beige solid; mp 236–238 °C; ^1^H NMR (DMSO-*d*_6_) *δ* 11.15 (s, 1H, *N*H-9), 8.40 (s, 1H, *N*H), 8.03 (d, ^3^*J*_2/3_ = 5.6 Hz, 1H, H-2), 7.38 (d, ^4^*J*_5/7_ = 2.1 Hz, 1H, H-5), 7.34 (d, ^4^*J*_2‘/6‘_ = 2.3 Hz, 1H, H-2‘), 7.29 (d, ^3^*J*_8/7_ = 8.5 Hz, 1H, H-8), 7.18 (dd, ^3^*J*_6‘/5‘_ = 8.4, ^4^*J*_6‘/2‘_ = 2.3 Hz, 1H, H-6‘), 7.16 (d, ^3^*J*_5‘/6‘_ = 8.4 Hz, 1H, H-5‘), 6.76 (dd, ^3^*J*_7/8_ = 8.5, ^4^*J*_7/5_ = 2.1 Hz, 1H, H-7), 6.72 (d, ^3^*J*_3/2_ = 5.6 Hz, 1H, H-3), 4.74 (s, 2H, *N*H_2_-6), 2.30 (s, 3H, CH_3_-4‘); ^13^C NMR (DMSO-*d*_6_) *δ* 153.88, 146.19, 145.52, 141.19, 133.43, 131.50, 130.46, 128.27, 120.68, 120.07, 118.61, 114.31, 110.70, 107.15, 104.12, 101.24, 18.86; HRMS (ESI) *m*/*z* (%) = calculated for C_18_H_16_ClN_4_ [M+H]^+^: 332.1058; found: 332.1059.

***Data for N*** **^4^** ***-(3-ethoxyphenyl)-9H-pyrido[2*** *,**3-b]indole-4**,**6-diamine*** **(9c)**

Yield 8%; greyish solid; mp 139–144 °C; ^1^H NMR (DMSO-*d*_6_) *δ* 11.11 (s, 1H, *N*H-9), 8.28 (s, 1H, *N*H), 8.01 (d, ^3^*J*_2/3_ = 5.6 Hz, 1H, H-2), 7.39 (d, ^4^*J*_5/7_ = 2.1 Hz, 1H, H-5), 7.22 (dd, ^3^*J*_5‘/6‘_ = 8.4, ^3^*J*_5‘/4‘_ = 7.8 Hz, 1H, H-5‘), 7.16 (d, ^3^*J*_8/7_ = 8.5 Hz, 1H, H-8), 6.89 (ddd, ^3^*J*_6‘/5‘_ = 8.4, ^4^*J*_6‘/2‘_ = 2.0, ^4^*J*_6‘/4‘_ = 0.9 Hz, 1H, H-6‘), 6.90–6.84 (m, 1H, H-2‘), 6.79 (d, ^3^*J*_3/2_ = 5.6 Hz, 1H, H-3), 6.74 (dd, ^3^*J*_7/8_ = 8.5, ^4^*J*_7/5_ = 2.1 Hz, 1H, H-7), 6.58 (ddd, ^3^*J*_4‘/5‘_ = 7.8, ^4^*J*_4‘/2‘_ = 2.3, ^4^*J*_4‘/6‘_ = 0.9 Hz, 1H, H-4‘), 4.67 (s, 2H, *N*H_2_-6), 4.01 (q, *J* = 7.0 Hz, 2H, CH_2_), 1.32 (t, *J* = 6.9 Hz, 3H, CH_3_); ^13^C NMR (DMSO-*d*_6_) *δ* 159.33, 153.87, 145.87, 143.02, 130.44, 129.83, 120.79, 114.19, 112.25, 110.64, 108.13, 107.21, 106.18, 104.00, 101.39, 62.87, 14.68; HRMS (ESI) *m*/*z* (%) = calculated for C_19_H_19_N_4_O [M+H]^+^: 319.1553; found: 319.1556.

***Data for 3-((6-amino-9H-pyrido[2*** *,**3-b]indol-4-yl)amino)phenol*** **(9d)**

Yield 12%; brownish solid; mp 164–169 °C; ^1^H NMR (DMSO-*d*_6_) *δ* 11.12 (s, 1H, *N*H-9), 9.35 (s, 1H, OH-1‘), 8.20 (s, 1H, *N*H), 8.00 (d, ^3^*J*_2/3_ = 5.6 Hz, 1H, H-2), 7.40 (d, ^4^*J*_5/7_ = 2.2 Hz, 1H, H-5), 7.16 (d, ^3^*J*_8/7_ = 8.6 Hz, 1H, H-8), 7.12 (t [dd], ^3^*J*_5‘/4‘_ = 8.4, ^3^*J*_5‘/6‘_ = 8.4 Hz, 1H, H-5‘), 6.76 (dd, ^3^*J*_7/8_ = 8.6, ^4^*J*_7/5_ = 2.2 Hz, 1H, H-7), 6.75 (d, ^3^*J*_3/2_ = 5.6 Hz, 1H, H-3), 6.76–6.67 (m, 2H, H-4‘, H-2‘), 6.44 (ddd, ^3^*J*_6‘/5‘_ = 8.4, ^4^*J*_6‘/2‘_ = 2.2, ^4^*J*_6‘/4‘_ = 1.0 Hz, 1H, H-6‘), 4.80 (s, 2H, *N*H_2_-6); ^13^C NMR (DMSO-*d*_6_) *δ* 158.09, 146.56, 146.24, 142.74, 139.11, 130.52, 129.78, 124.18, 122.05, 120.83, 114.26, 111.24, 110.65, 109.48, 107.25, 103.86, 101.41; HRMS (ESI) *m*/*z* (%) = calculated for C_17_H_15_N_4_O [M+H]^+^: 291.1240; found: 291.1240.

**Protein Kinase Activity Determination**

The protein kinases Brk as human Brk (NBCI/Protein entry NP_005966.1) and HER2 as human HER2 (GenBank entry X03363) were all expressed as human recombinant GST fusion proteins in Sf9 insect cells. Purification was performed using affinity chromatography over GSH-agarose. The achieved kinase purity was checked using SDS-PAGE/Coomassie staining techniques.

**Assay Conditions for Affinity Determinations**

The assay buffer consisted of 70 mM of HEPES-NAOH, 3 mM of magnesium chloride and 3 mM manganese(II) chloride, 3 µM of sodium orthovanadate, 1.2 mM of DTT, 50 µg/mL of PEG_20000_ and finally [γ-^33^P]-ATP that made about 4 × 10^5^ cpm in each well. Further, 17.1 nM for Brk and 14.9 nM for HER2 were the final kinase concentrations. Poly(Glu,Tyr) 4:1 was used as substrate for both Brk and HER2 in an amount of 125 ng/50 µL. Incubation of the mixtures was performed for 60 min at 30 °C and washed subsequently. Scintillation counting of the incorporated ^33^Pi was conducted using a microplate scintillation counter. The IC_50_ values were determined from the measured reduced enzyme activities at the given concentrations. Concentration-dependent activity (inhibition) curves of selected compounds from all the compound classes with dual activities and the staurosporine control are shown in the Supplementary Materials.

**Cancer Cell Growth Inhibition and Cell Viability Assay**

The human cancer cell lines were cultured in RPMI 1640 medium (Merck, Darmstadt, Germany) with 5% foetal bovine serum and 2 mM L-glutamine. Cell inoculation was performed in 96-well microtiter plates in 100 mL. The plate densities ranged from 5000 to 40,000 cells/well and depended on the cell line doubling time. The incubation of the microtiter plates was carried out for 24 h at 37 °C, 5% CO_2_, 95% air and 100% relative humidity before addition of the used inhibitors. Two plates of each cell line were fixed after 24 h in situ with TCA in order to represent a measurement of the cell population at the time of compound addition (Tz). The inhibitors were dissolved in dimethylsulfoxide at 400-fold the desired final maximum of the test concentration. The samples were stored and frozen before use. For drug addition, a part of the dissolved concentrate was diluted to twice the final maximum test concentration accompanied by a complete medium exchange that contained 50 µg/mL gentamicin. Additional 4-, 10-fold or ½ log serial dilutions were performed, providing a total of five compound concentrations. Further, 100 µL of the different compound dilutions was added to the appropriate microtiter wells containing 100 µL of medium, giving the required final compound concentrations. Incubation of the plates followed at 37 °C, 5% CO_2_, 95% air, and 100% relative humidity for 48 h. The assay of adherent cells was stopped by the addition of cold TCA. Fixing of the cells were carried out in situ by the addition of 50 µL of cold 50% (*w*/*v*) TCA making 10% as the final TCA concentration. Incubation followed at 4 °C for 60 min. After removal of the supernatant, the plates were washed five times with water and finally air dried. Sulforhodamine B (SRB) solution (100 µL) at 0.4% (*w*/*v*) in 1% acetic acid was then added to each well, followed by plate incubation at room temperature for 10 min. The unbound dye was removed after staining by washing five times with 1% acetic acid. Solubilization of the bound stain was done in 10 mM trizma base. The absorbance was read using an automated plate reader at a wavelength of 515 nm. For suspension cells, the assay followed the same method and was terminated by fixing the settled cells at the bottom of the wells by addition of 50 mL of 80% TCA making a final concentration of 16% TCA. Using seven absorbance measurements [time zero, (Tz), control growth, (C), and test growth in the presence of compound at the five concentration levels (Ti)], the percentage growth was calculated for each compound concentration. Percentage growth inhibition was calculated as follows:

[(Ti − Tz)/(C − Tz)] × 100 for concentrations for which Ti ≥ Tz

[(Ti − Tz)/Tz] × 100 for concentrations for which Ti < Tz

The calculation of the growth inhibition of 50% (GI_50_) was performed with [(Ti − Tz)/(C − Tz)] × 100 = 50, which is the drug concentration resulting in a 50% reduction in the net protein increase measured by SRB staining in control cells during the drug incubation. The dose-dependent growth inhibition curves of the NCI are shown in the Supplementary Materials.

The LC_50_ (concentration of drug resulting in a 50% reduction in the measured protein at the end of the drug treatment as compared to that at the beginning), indicating a net loss of cells following treatment, is calculated from:

[(Ti − Tz)/Tz] × 100 = −50.

**Protein Preparation**

The structures for the human Brk (PDB ID: 5DA3) and HER2 (PDB ID: 7JXH) were retrieved from the Protein Data Bank (PDB; https://www.rcsb.org/). Proteins were prepared using the Protein Preparation Wizard in Schrödinger (version 2025-2) [38,39]. This workflow included the assignment of bond orders, the addition of hydrogen atoms and the reconstruction of missing side chains. Protonation states were assigned using PROPKA at pH 7.0. Finally, restrained minimization was performed using the OPLS4 force field with an RMSD cutoff of 0.3 Å for heavy atoms [40,41,42,43].

**Ligand Preparation**

The co-crystalized ligands and synthesized inhibitors were prepared using the LigPrep tool in Schrödinger (version 2025-2) [44]. Protonation states were assigned at pH 7.0 ± 1.0 with Epik, followed by energy minimization using the OPLS4 force field [39,45]. Subsequently, a maximum of 64 conformers were generated and minimized for each ligand using ConfGen [46].

**Docking**

Molecular docking was performed with Glide in Standard Precision (SP) mode [47,48,49,50,51]. The synthesized inhibitors (**6j**, **6l**, **8d** and **8e**) were docked in the ATP-binding site of Brk (PDB ID 5DA3) and HER2 (PDB 7JXH). Receptor grids with dimensions of 10 × 10 × 10 Å were generated around the co-crystalized ligands utilizing the Receptor Grid Generation panel. During docking, up to 100 poses per ligand were generated and refined by post-docking minimization. All other parameters were kept at their default settings. The resulting docking poses were ranked according to their docking scores.

The docking protocol was validated by redocking co-crystalized ligands into their respective protein structure. Glide successfully reproduced the experimental binding modes, yielding RMSD values of 0.9 Å for Brk (PDB ID: 5DA3) and 1.3 Å for HER2 (PDB ID: 7JXH) for the top-scored docking poses.

**Co-folding**

To provide independent support for the docking poses, co-folding was performed using Boltz-2 software following the official instructions on the GitHub platform [52]. Protein sequences for the human Brk and HER2 were retrieved from the UniProtKB (entry numbers Q13882 and P04626, respectively), while the co-crystalized ligands and inhibitors were provided as SMILES strings. Predictions were generated using multiple sequence alignment, 10 recycling steps, 200 sampling steps, and 5 diffusion samples. The co-folding protocol was validated by predicting complexes of the co-crystalized ligands with their respective proteins. Boltz-2 reproduced the experimental structures with Cα RMSD values of 0.9 Å for Brk and 1.4 Å for HER2. Subsequently, the same protocol was applied to the synthesized inhibitors.

**ADMET Prediction**

The pharmacokinetic and physiochemical properties of the synthesized inhibitors (**6j**, **6l**, **8d** and **8e**) were predicted using QikProp in Schrödinger (version 2025-2) [53].

## 4. Conclusions

With increasing resistance to established protein kinase inhibitors used in HER2-positive cancer, such as lapatinib or neratinib, and the limited options to treat triple-negative breast cancer, there is an urgent need for novel inhibitors of prospective targets for cancer therapies. Brk overexpression in these types of breast cancer makes Brk an attractive target for drug development. Thus, we developed novel pyrrolopyridines with varying substituents both at the molecular scaffold and the 4-position of the pyridine core and observed partly nanomolar inhibitory activities against Brk, with a 3-hydroxyaniline substitution in the 4-position that was strengthened by additional bromo and nitro functions in the 6-position of the benzo-annelated pyrrolopyridines. The best of the compounds identified demonstrated additional activity against HER2, making them promising candidates for breast cancer treatment. Lastly, we proved that the different Brk and HER2 inhibitory activities resulted in different breast cancer cell growth inhibition activities, which reflects these properties as a proof of concept. Thus, our novel compound class exhibits strong potential as a prospective agent for anticancer drugs in the treatment of Brk-relevant cancers.

## Figures and Tables

**Table 1:** Enzyme inhibition data as IC_50_ values for target compound series **2a**–**d**, **6a**–**m**, **8a**–**e** and **9a**–**d** with varied substitution patterns against Brk and HER2, respectively, with standard deviations in brackets.

			IC_50_ Value [µM] ^a^	
Compound	R^1^	R^2^	Brk	HER2
**2a**	Cl	H	92.9 (0.93)	>100
**2b**	NO_2_	H	1.45 (0.39)	66.5 (7.81)
**2c**	Cl	Me	9.97 (1.81)	23.1 (2.69)
**2d**	OH	M	0.83 (0.13)	>100
**6a**	Cl	H	2.76 (0.14)	>100
**6b**	Cl	Me	1.26 (0.50)	10.43 (0.65)
**6c**	Cl	Cl	2.04 (0.57)	22.15 (1.45)
**6d**	NO_2_	H	8.42 (0.95)	47.3 (4.12)
**6e**	NO_2_	Me	4.69 (0.12)	34.95 (0.88)
**6f**	NO_2_	F	2.69 (0.11)	21.9 (1.3)
**6g**	NO_2_	NH_2_	0.43 (0.03)	>100
**6h**	NO_2_	OH	0.31(0.11)	17.8 (2.54)
**6i**	F	H	0.11 (0.02)	17.6 (0.61)
**6j**	CF_3_	H	27.1 (0.26)	>100
**6k**	OMe	H	0.04 (0.005)	17.3 (3.53)
**6l**	OH	H	<0.003	3.35 (1.00)
**6m**	OH	OMe	0.02 (0.007)	6.11 (2.4)
**8a**	Cl	H	2.96 (0.12)	81.1 (7.9)
**8b**	Cl	Me	1.01 (0.17)	28.5 (2.83)
**8c**	OEt	H	5.51 (0.85)	82.5 (9.5)
**8d**	OH	H	<0.003	2.47 (0.68)
**8e**	H	CF_3_	7.85 (0.65)	>100
**9a**	Cl	H	0.37 (0.065)	3.55 (0.22)
**9b**	Cl	Me	1.61 (0.22)	6.67 (0.73)
**9c**	OEt	H	3.41 (0.65)	<100
**9d**	OH	H	0.01 (0.001)	4.34 (0.79)
staurosporine			0.89 (0.13)	0.19 (0.01)

^a^ mean of two determinations.

**Table 2:** Breast cancer cell growth inhibition data as GI_50_ values for selected compounds **6a**, **6k** and **6l** in NCI cell lines MCF7, MDA-MB-231 and HS 578T, respectively.

		GI_50_ Value [µM]	
Compound	MCF7	MDA-MB-231	HS 578T
**6a**	3.73	11.5	35.9
**6k**	0.05	1.94	2.98
**6l**	0.44	1.03	0.54
lapatinib	6.30	10.3	n.a. ^a^

^a^ not available.

## Data Availability

The original contributions presented in this study are included in the article and Supplementary Materials. Further inquiries can be directed to the corresponding author.

## Supplementary Materials

The following supporting information can be downloaded at: https://www.mdpi.com/article/10.3390/molecules31152730/s1, Figure S1: Enzyme activity curves for compound **2c** for BRK 1st (top) and BRK 2nd (bottom); Figure S2: Enzyme activity curves for compound **2c** for HER2 1st (top) and HER2 2nd (bottom); Figure S3: Enzyme activity curves for compound **6k** for Brk 1st (top) and Brk 2nd (bottom); Figure S4: Enzyme activity curves for compound **6k** for HER2 1st (top) and HER2 2nd (bottom); Figure S5: Enzyme activity curves for compound **6m** for Brk 1st (top) and Brk 2nd (bottom); Figure S6: Enzyme activity curves for compound **6m** for HER2 1st (top) and HER2 2nd (bottom); Figure S7: Enzyme activity curves for compound **8d** for Brk 1st (top) and Brk 2nd (bottom); Figure S8: Enzyme activity curves for compound **8d** for HER2 st (top) and HER2 2nd (bottom); Figure S9: Enzyme activity curves for compound **9a** for Brk 1st (top) and Brk 2nd (bottom); Figure S10: Enzyme activity curves for compound **9a** for HER2 1st (top) and HER2 2nd (bottom); Figure S11: Enzyme activity curves for compound **9d** for Brk 1st (top) and Brk 2nd (bottom); Figure S12: Enzyme activity curves for compound **9d** for HER2 1st (top) and HER2 2nd (bottom) Figure S13: Enzyme activity curves for staurosporine for Brk 1st (top) and Brk 2nd (bottom); Figure S14: Enzyme activity curves for staurosporine for HER2 1st (top) and HER2 2nd (bottom); Figure S15: Dose–response curves in breast cancer cells for compound **6a**; Figure S16: Dose–response curves in breast cancer cells for compound **6k**; Figure S17: Dose–response curves in breast cancer cells for compound **6l**.
