# Supplementary material for: Novel Functionalized Pyrrolopyridines to Target Brk
Source: Molecules. 2026 Aug 6;31(15):2730. doi: 10.3390/molecules31152730 (PMC13467776; doi:10.3390/molecules31152730)
Supplement: Supplementary file 1 [file molecules-31-02730-s001.zip › molecules-4422488-supplementary.pdf]

Supplementary Information

Communication

Novel functionalized Pyrrolopyridines to target Brk

Erik Schmidt [1], Jannis von Veh [1], Anne-Christin Sarnow [1], Wolfgang Sippl [1], Julian Kowalski [1], Niels Heise [2], Frank Totzke [3] and Andreas Hilgeroth [1,*]

^{1}H NMR spectrum of compound **2a** (full spectrum)

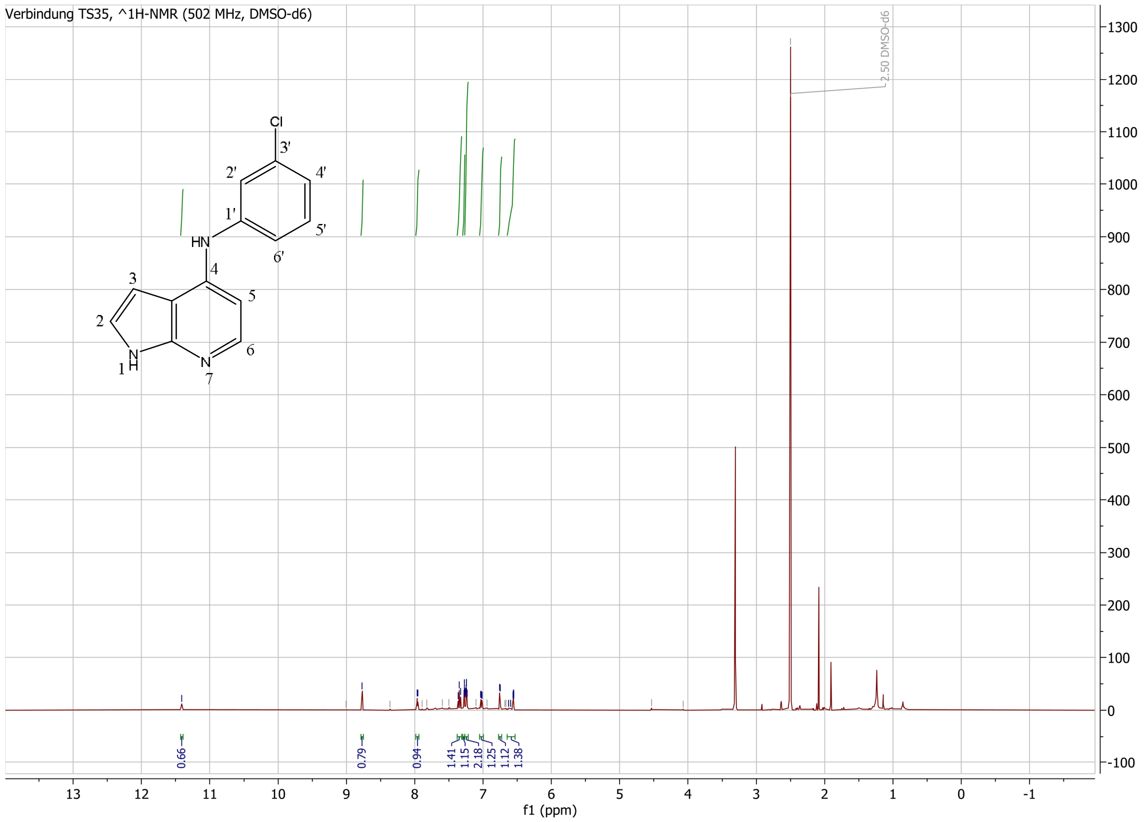

^{1}H NMR spectrum of compound **2a** (expanded region)

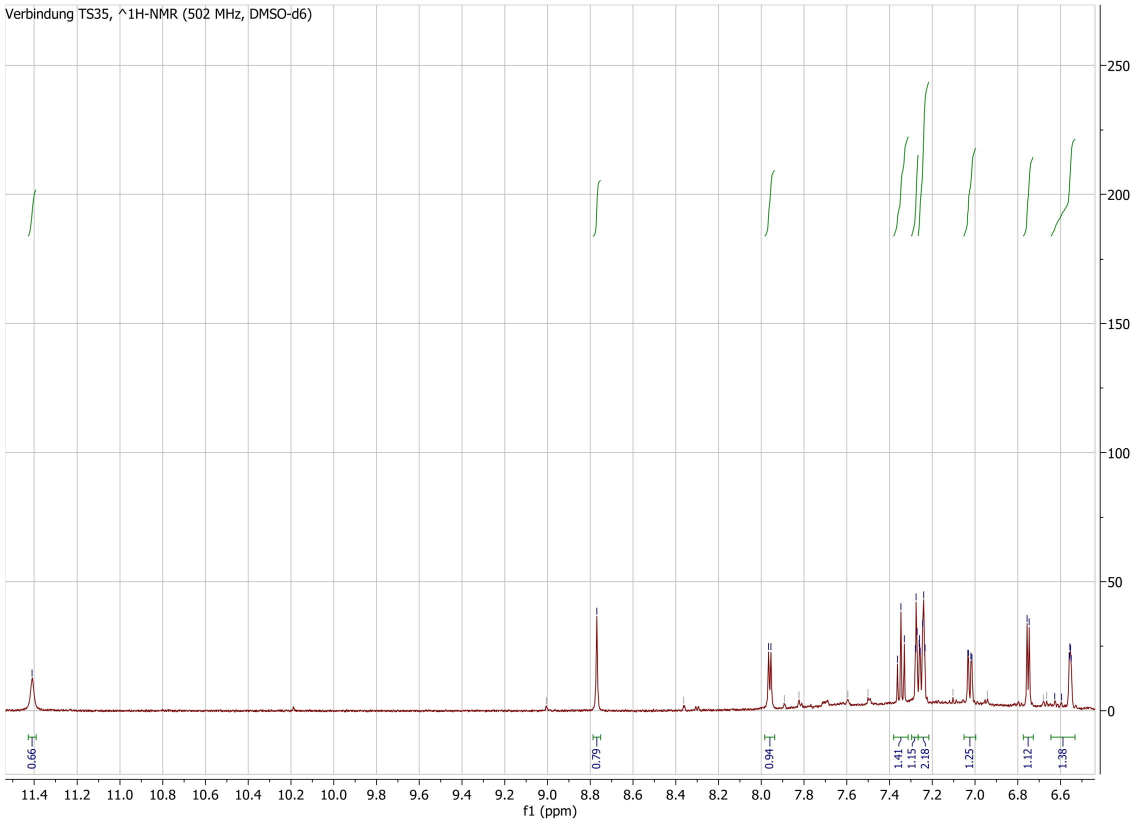

^{1}H NMR spectrum of compound **2b** (full spectrum)

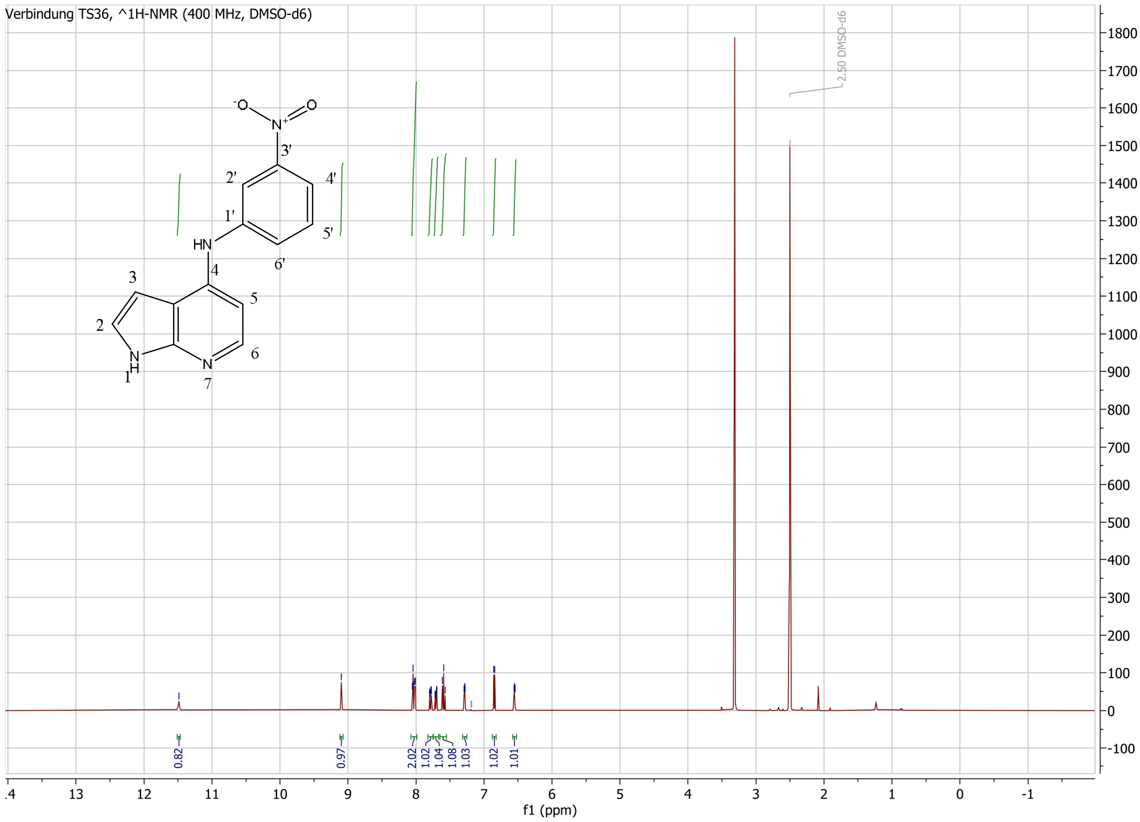

^{1}H NMR spectrum of compound **2b** (expanded region)

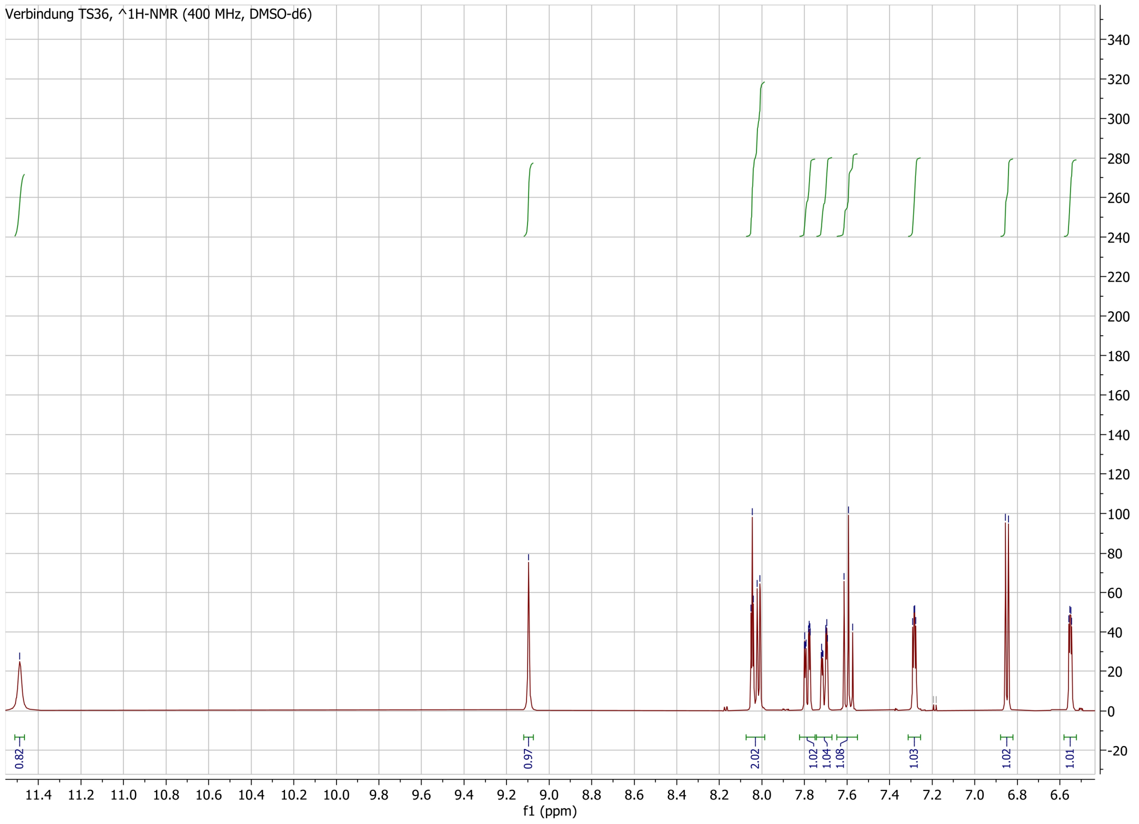

^{1}H NMR spectrum of compound **2c** (full spectrum)

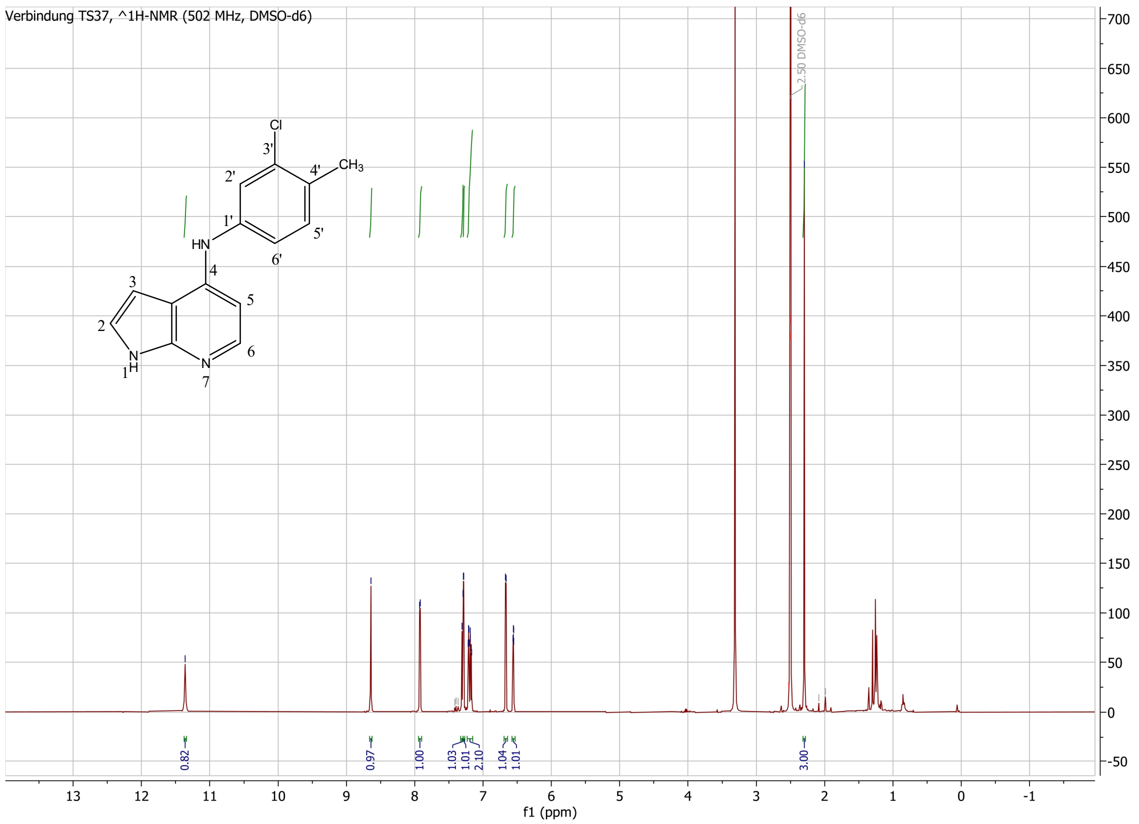

^{1}H NMR spectrum of compound **2c** (expanded region)

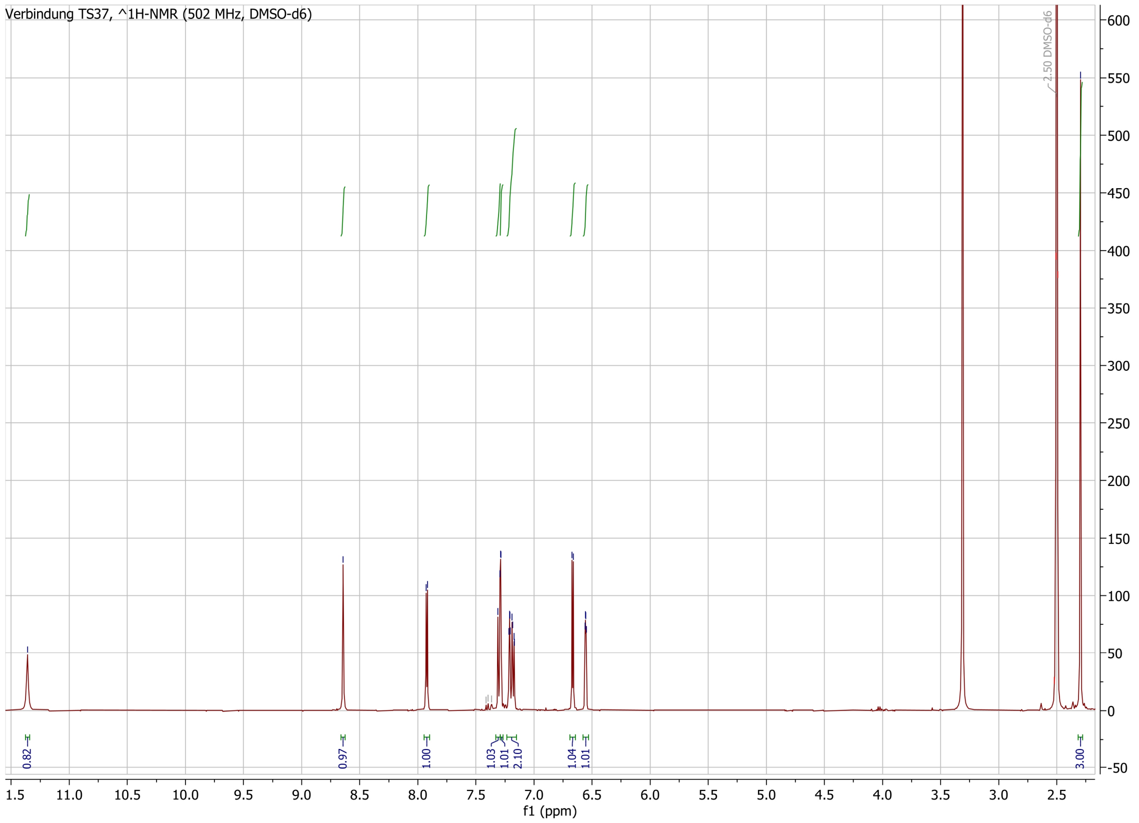

^{1}H NMR spectrum of compound **2d** (full spectrum)

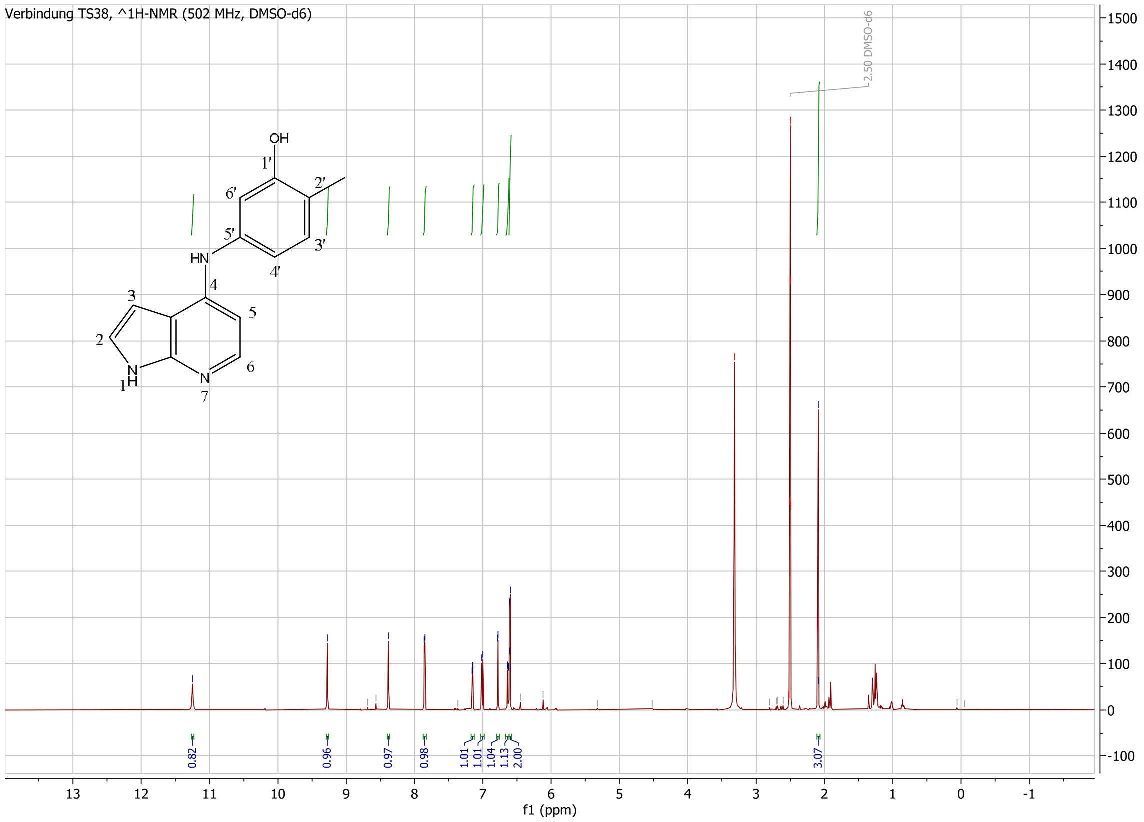

^{1}H NMR spectrum of compound **2d** (expanded region)

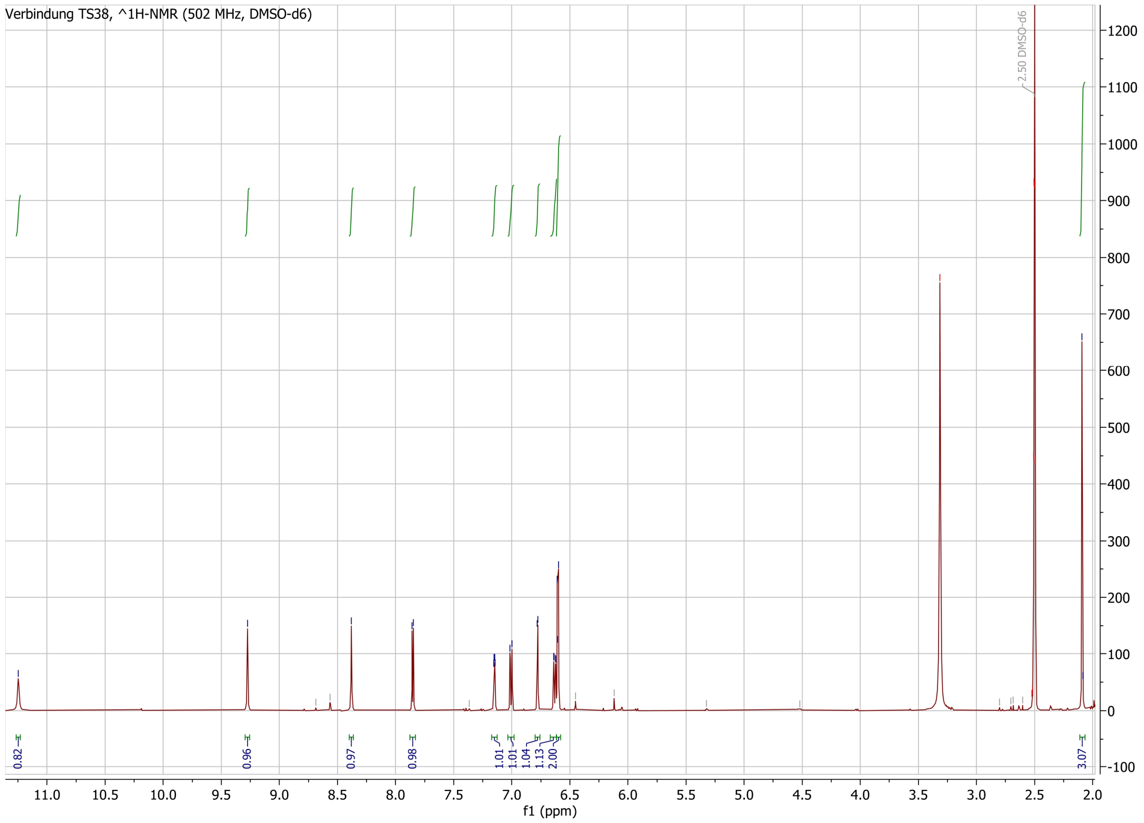

^{1}H NMR spectrum of compound **6a** (full spectrum)

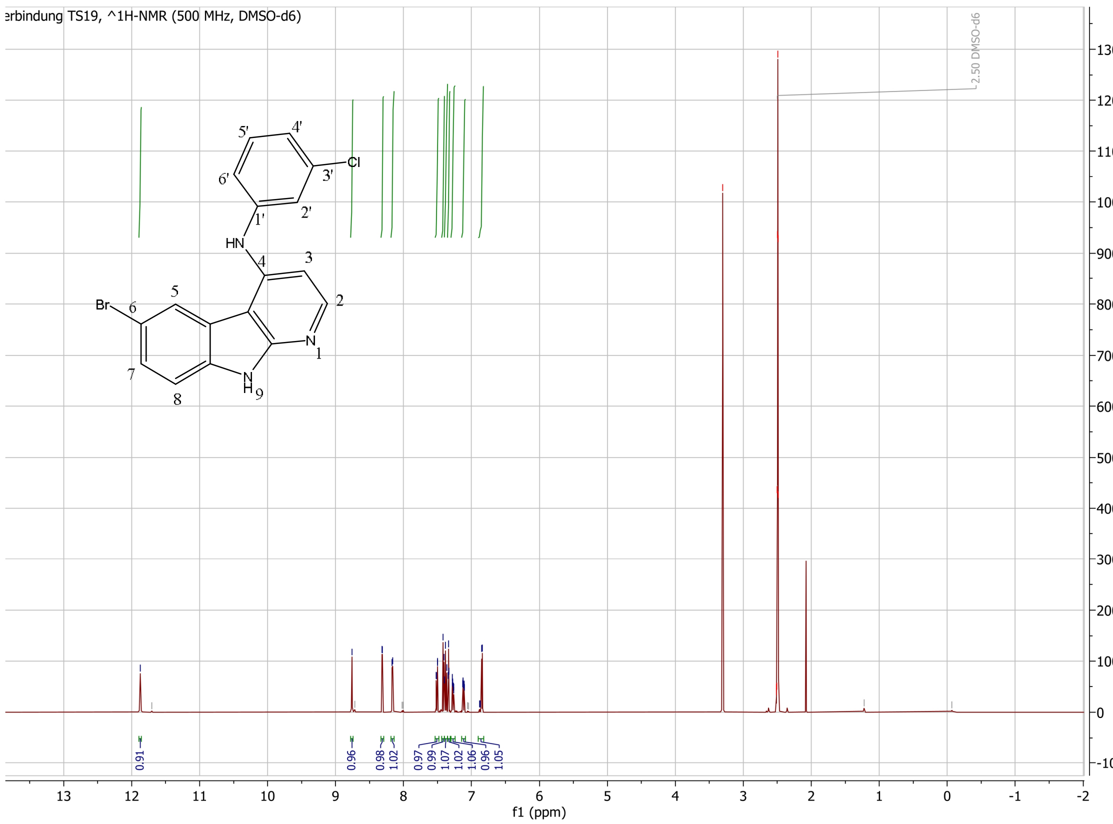

^{1}H NMR spectrum of compound **6a** (expanded region)

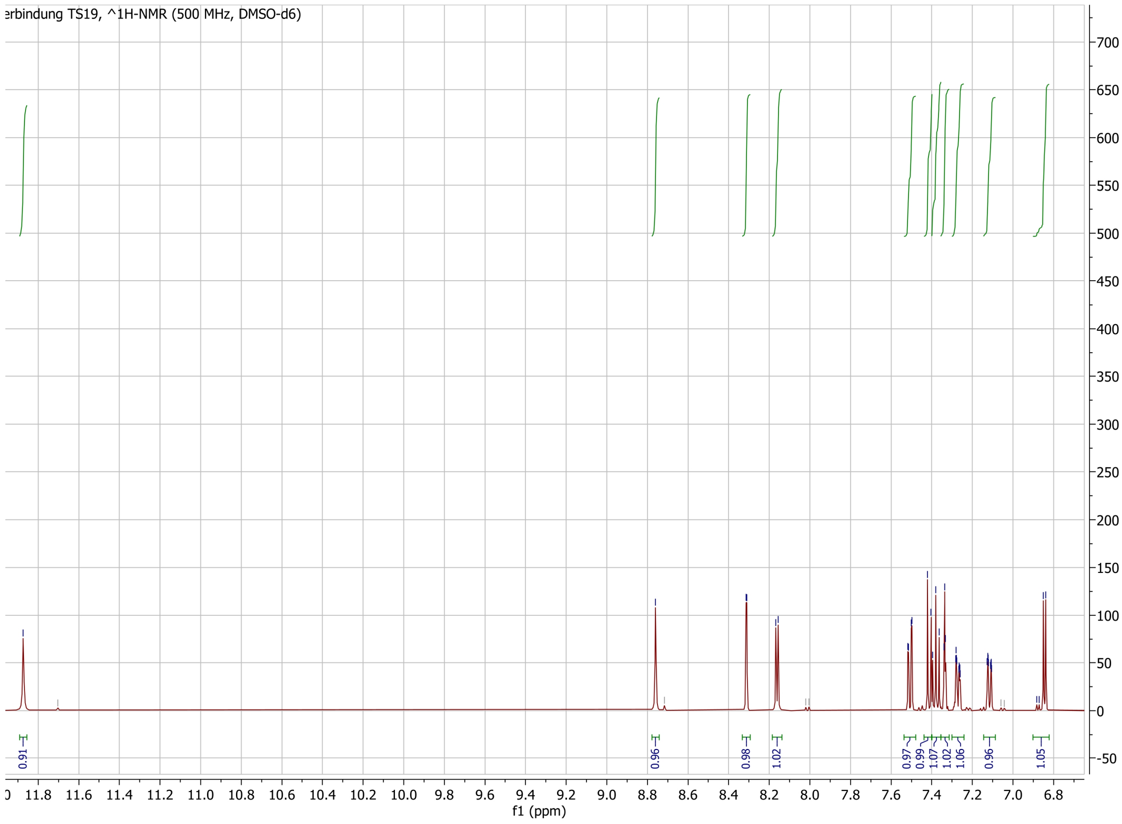

^{1}H NMR spectrum of compound **6b** (full spectrum)

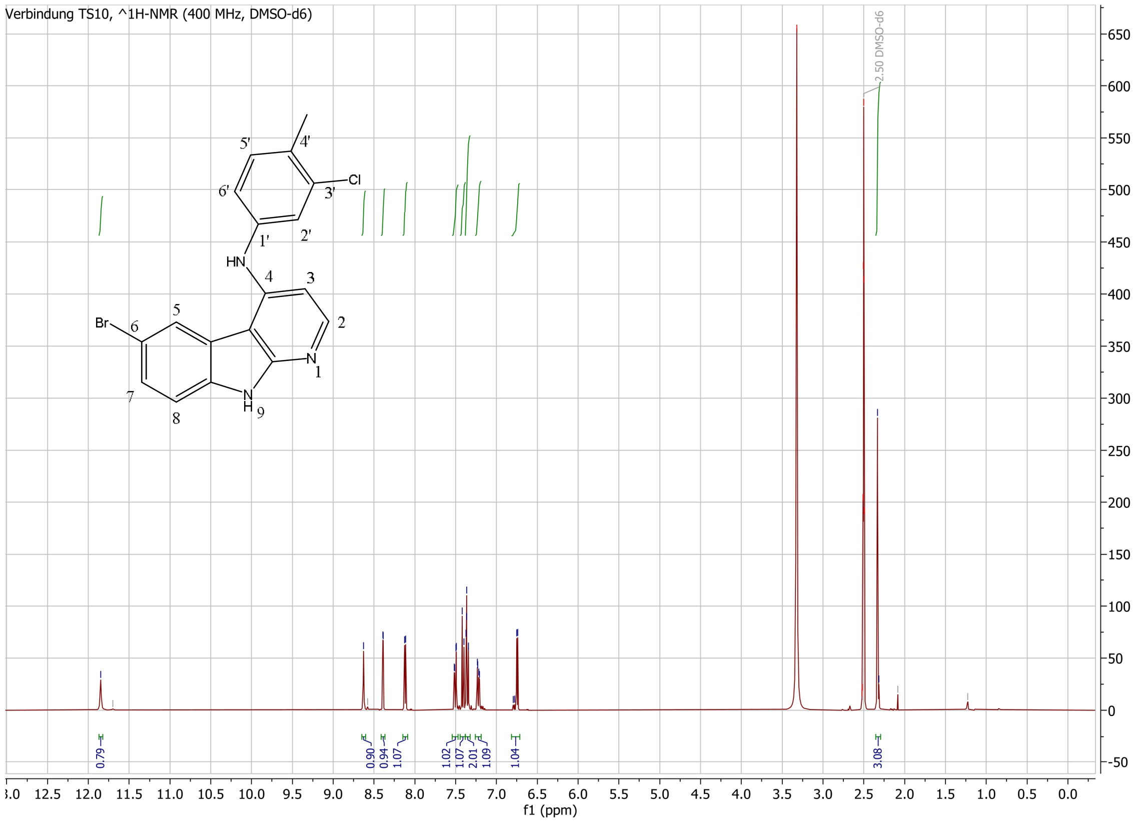

^{1}H NMR spectrum of compound **6b** (expanded region)

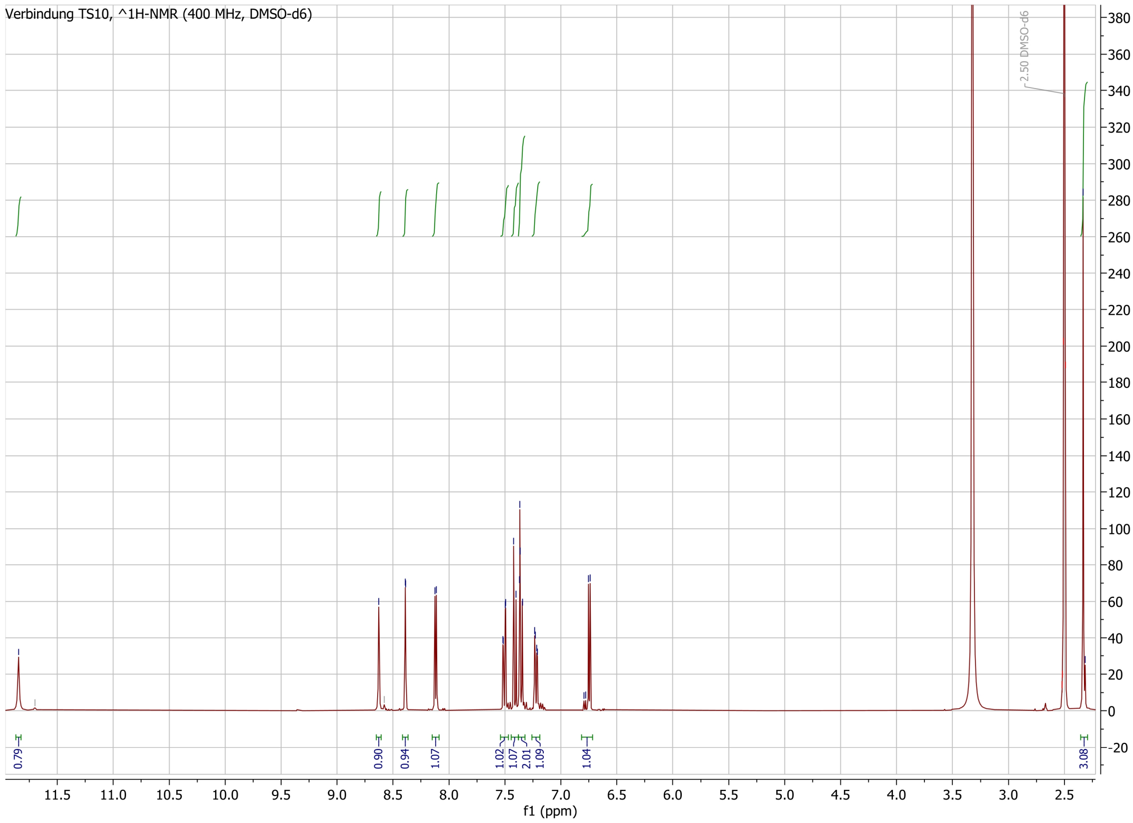

^1H NMR spectrum of compound **6c** (full spectrum)

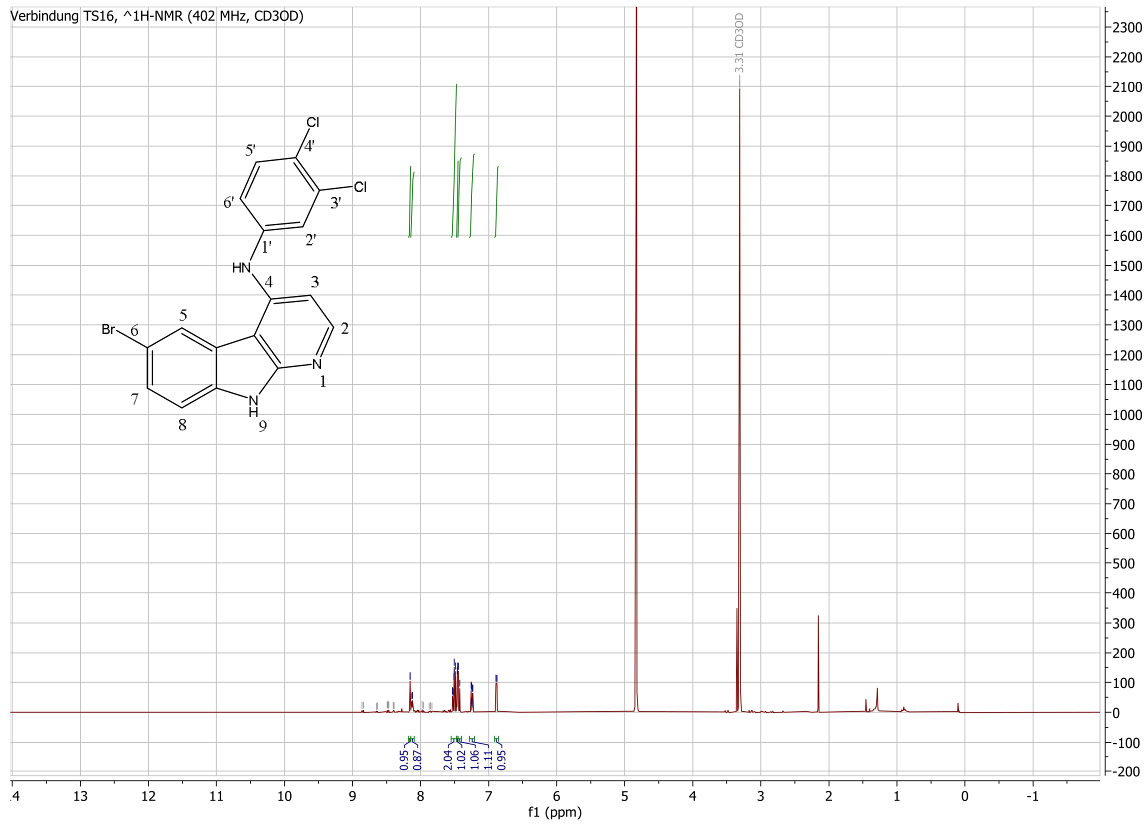

^1H NMR spectrum of compound **6c** (expanded region)

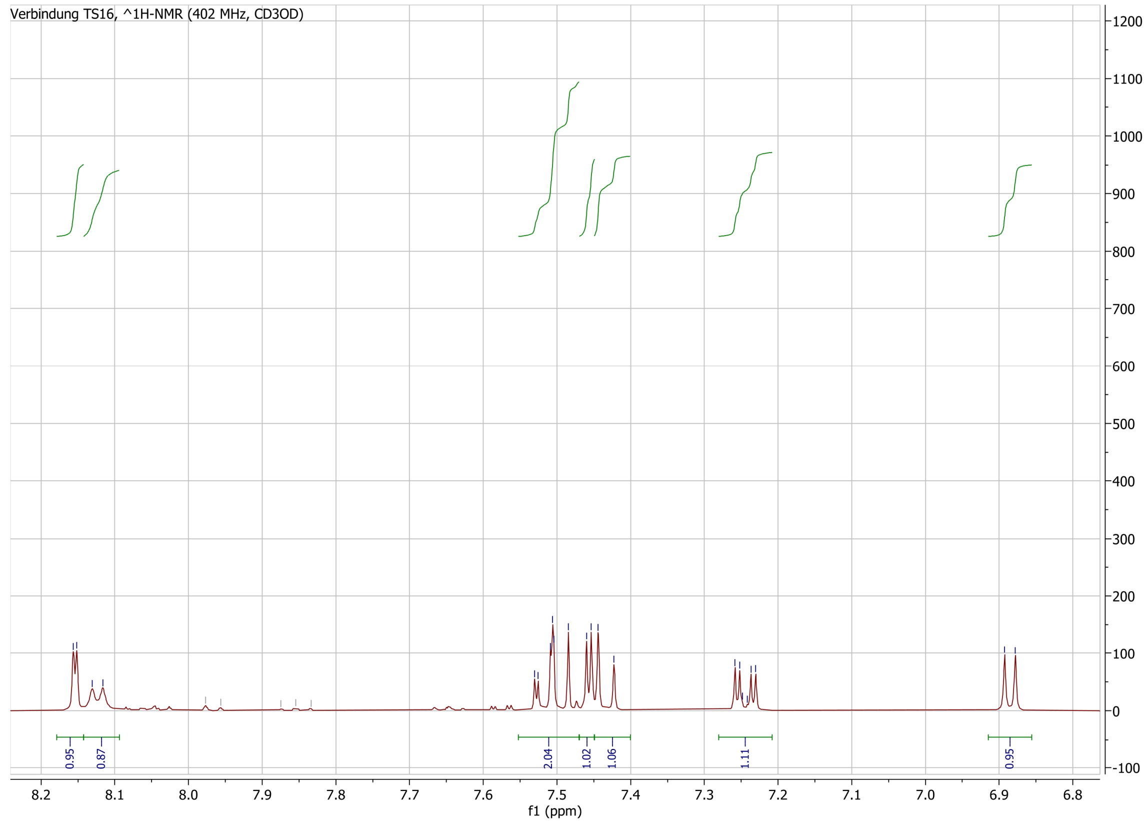

^{1}H NMR spectrum of compound **6d** (full spectrum)

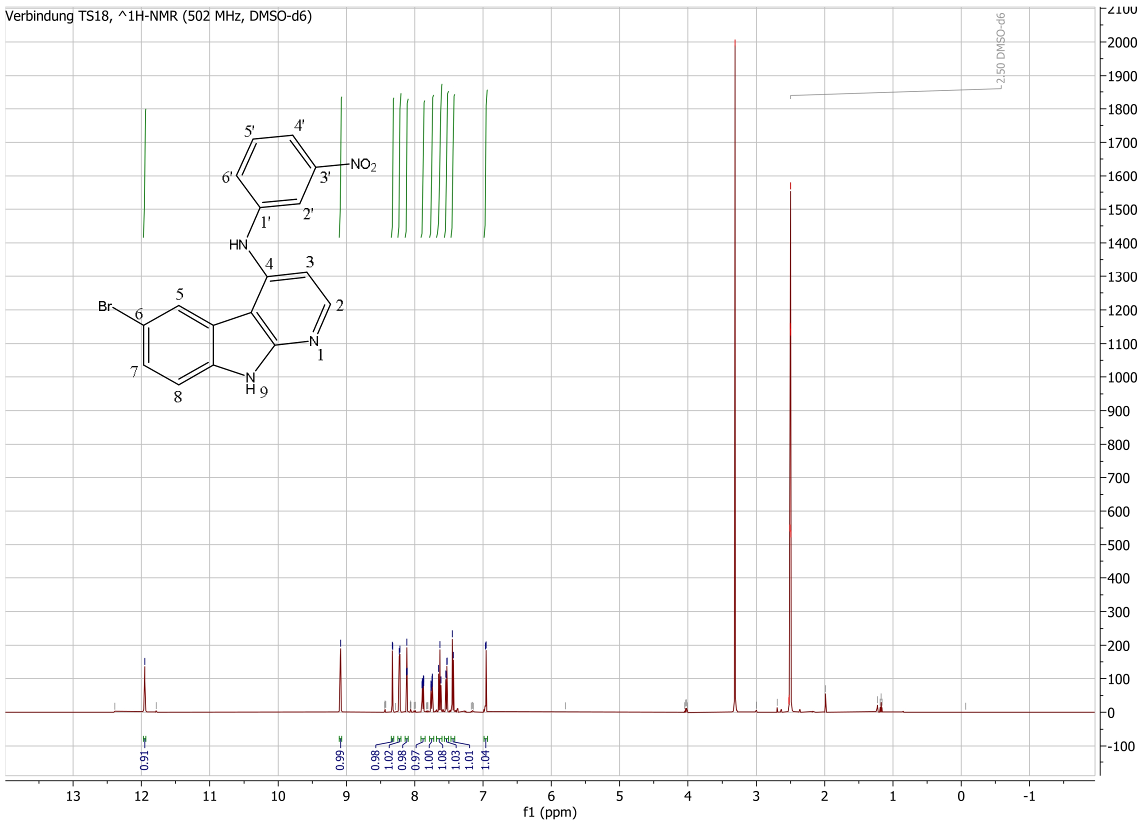

^{1}H NMR spectrum of compound **6d** (expanded region)

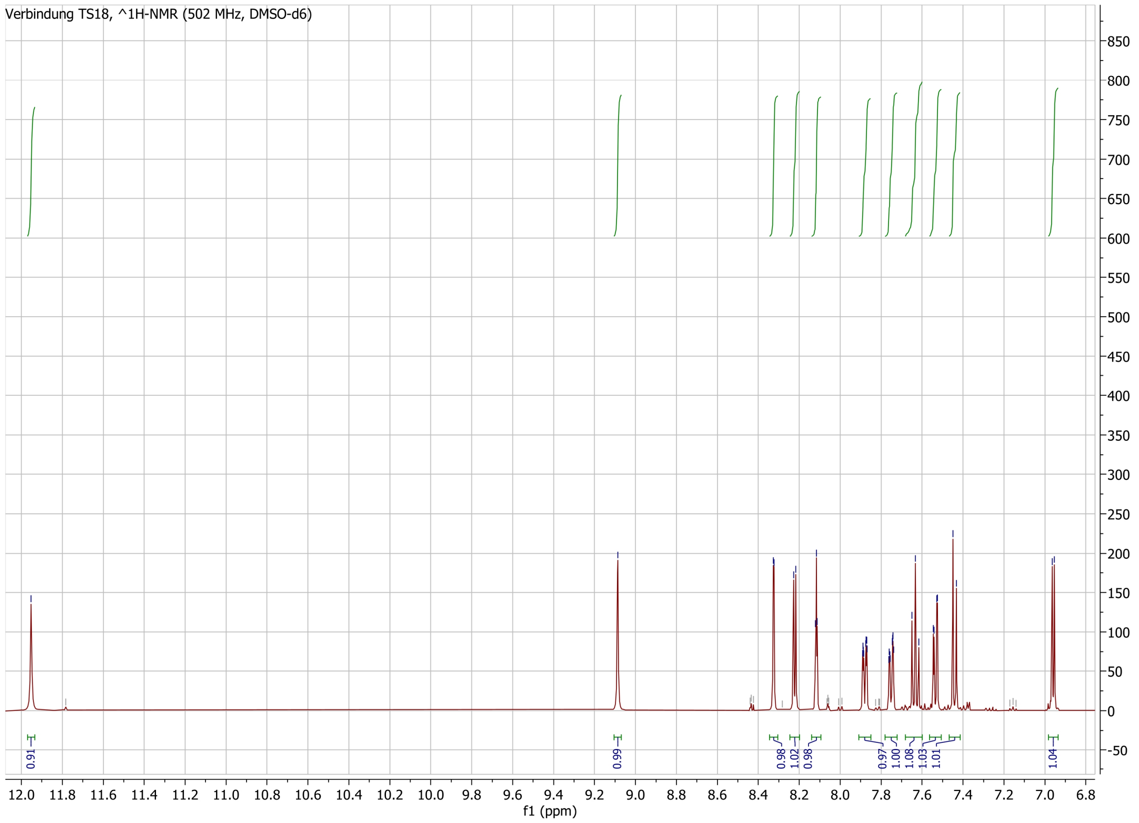

^{1}H NMR spectrum of compound **6e** (full spectrum)

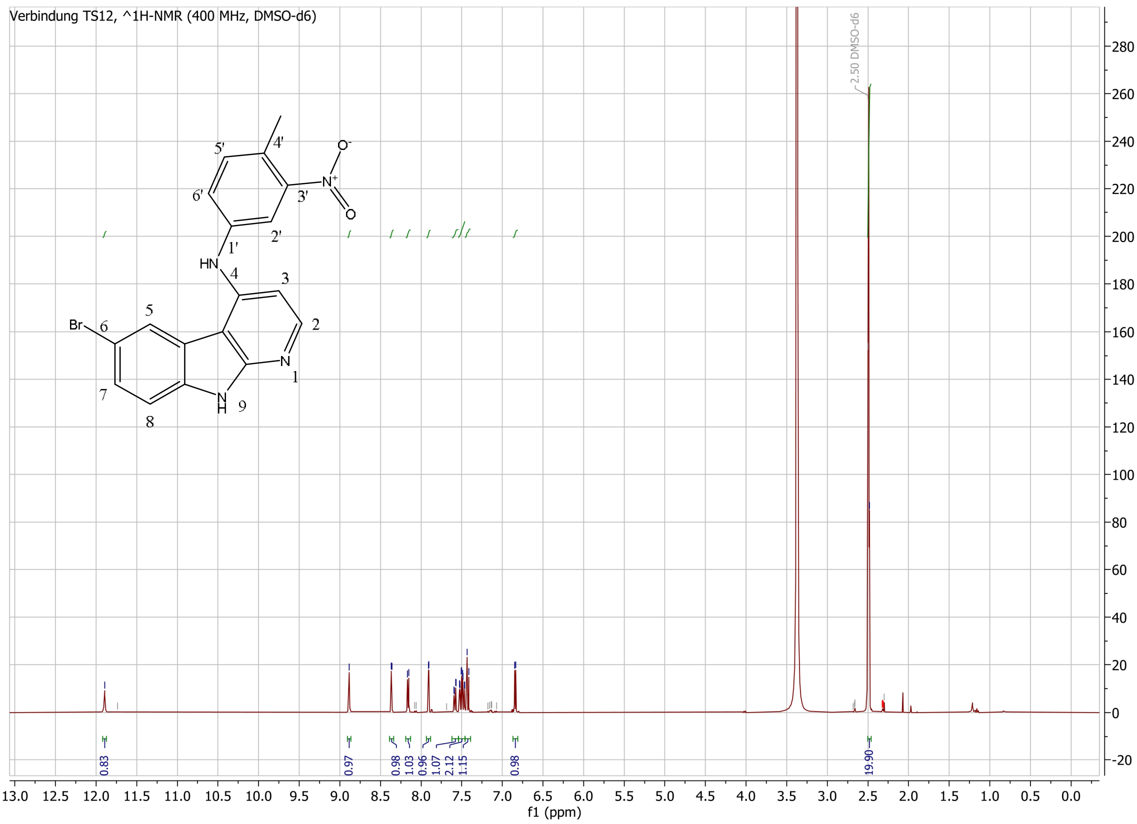

^{1}H NMR spectrum of compound **6e** (expanded region)

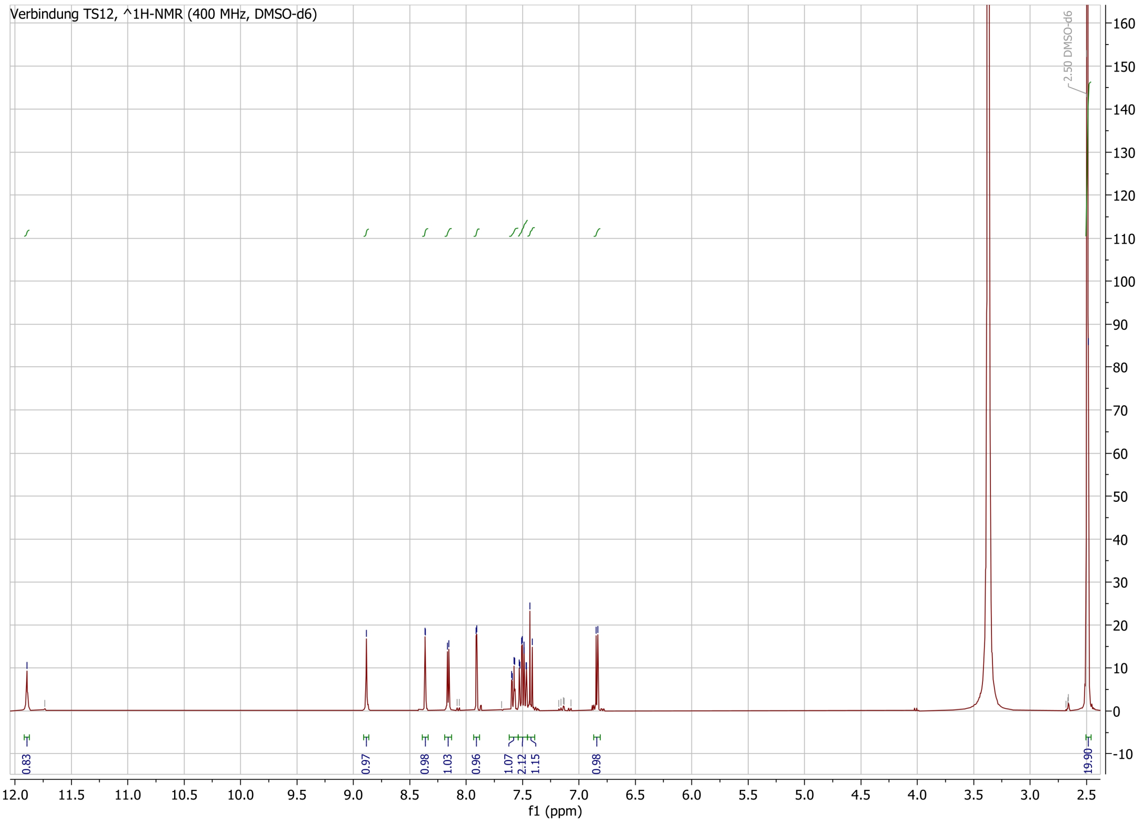

¹H NMR spectrum of compound **6f** (full spectrum)

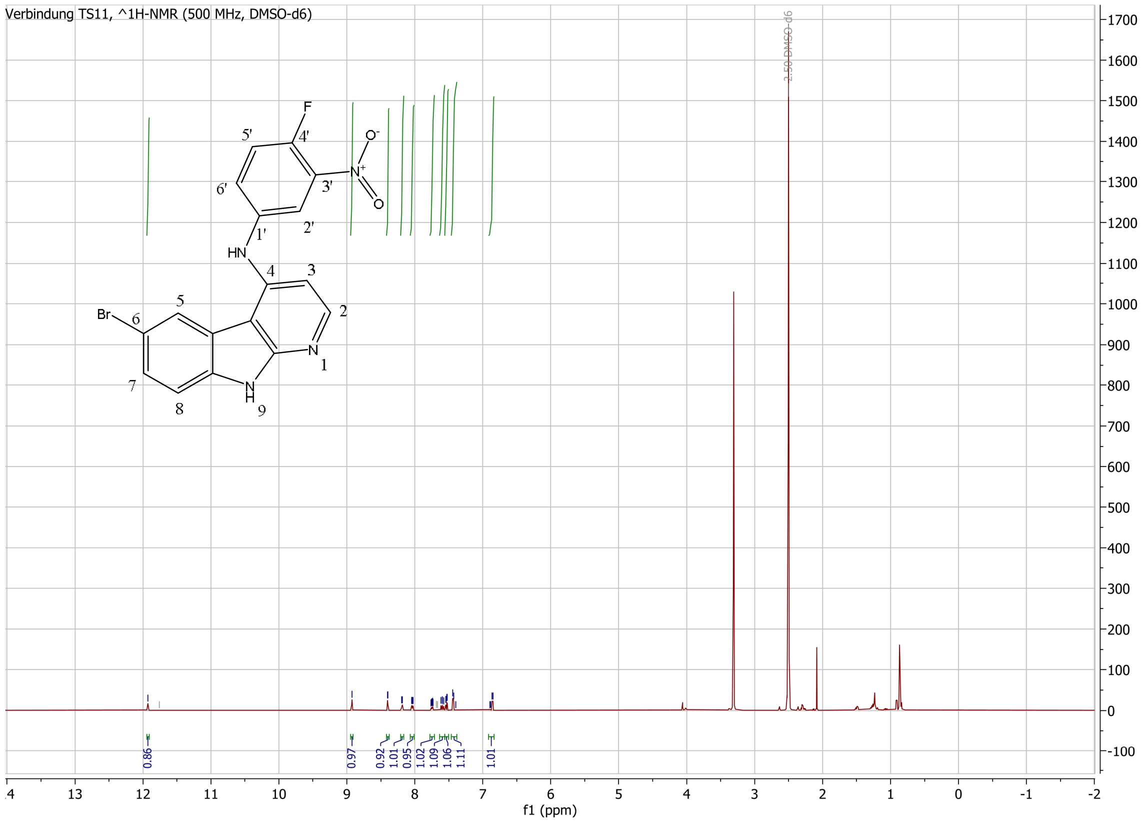

¹H NMR spectrum of compound **6f** (expanded region)

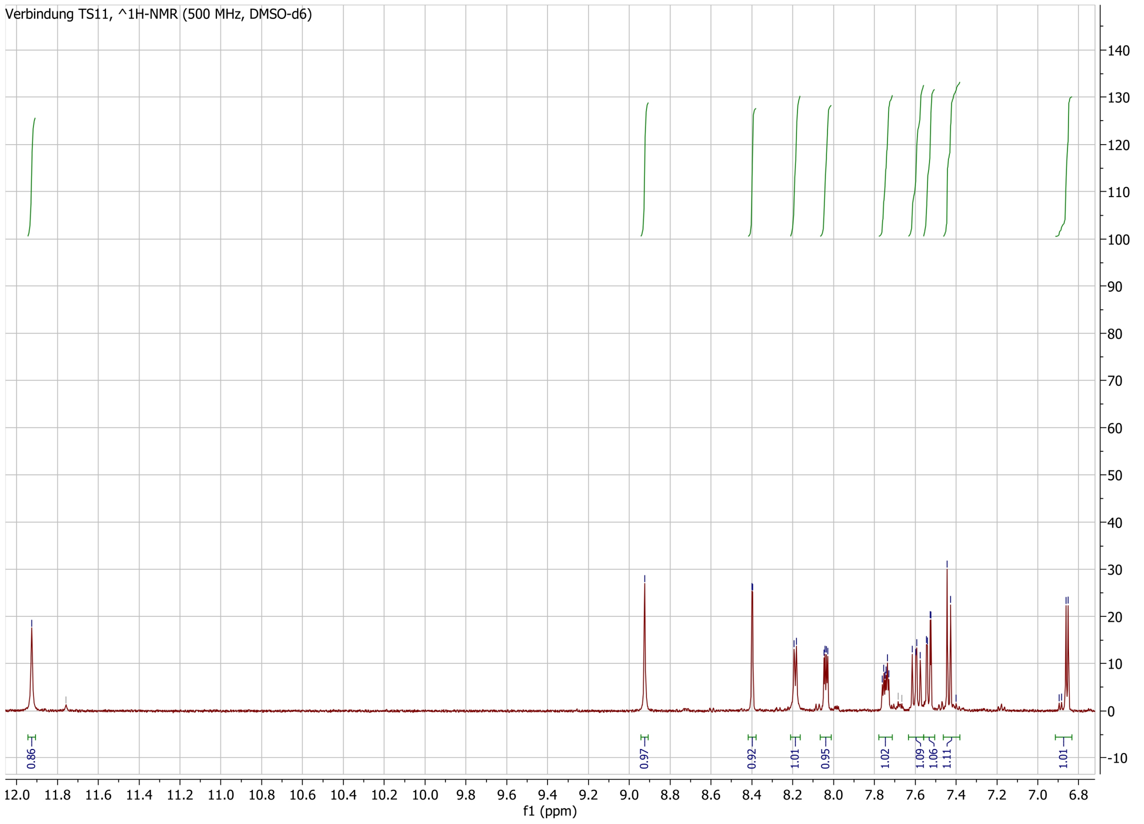

^{1}H NMR spectrum of compound **6g** (full spectrum)

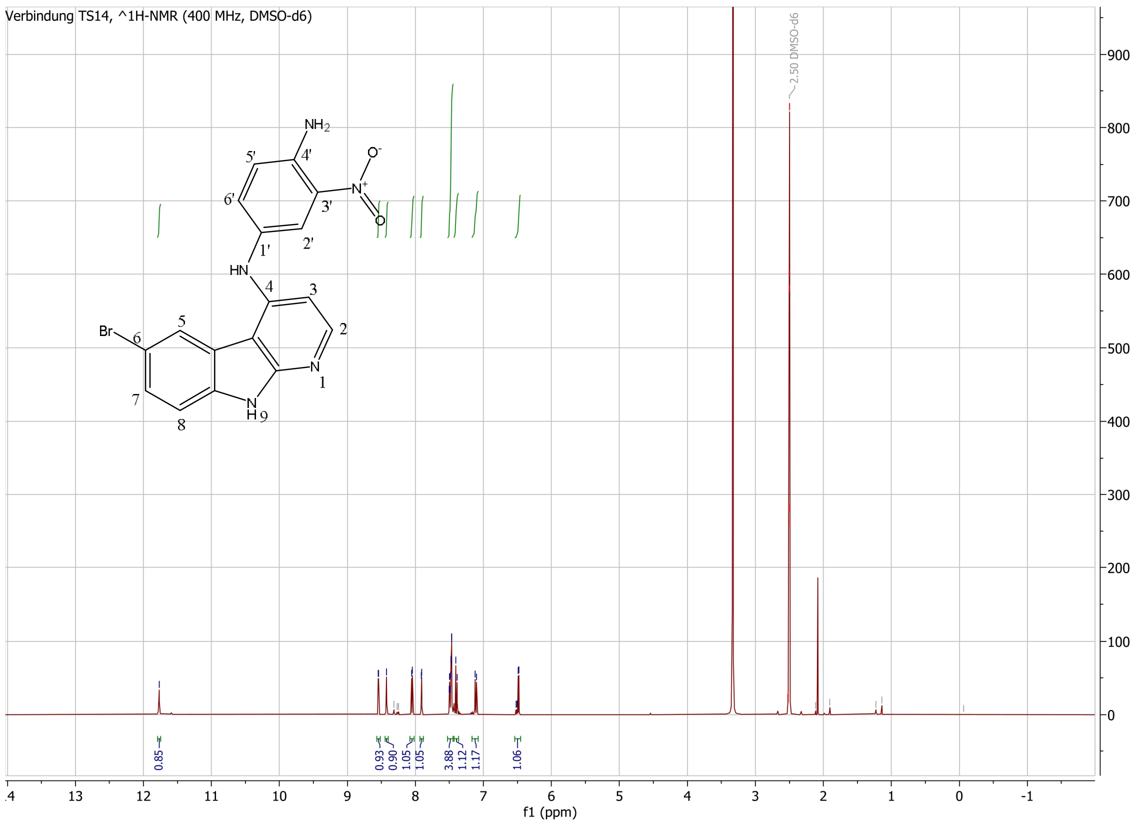

^{1}H NMR spectrum of compound **6g** (expanded region)

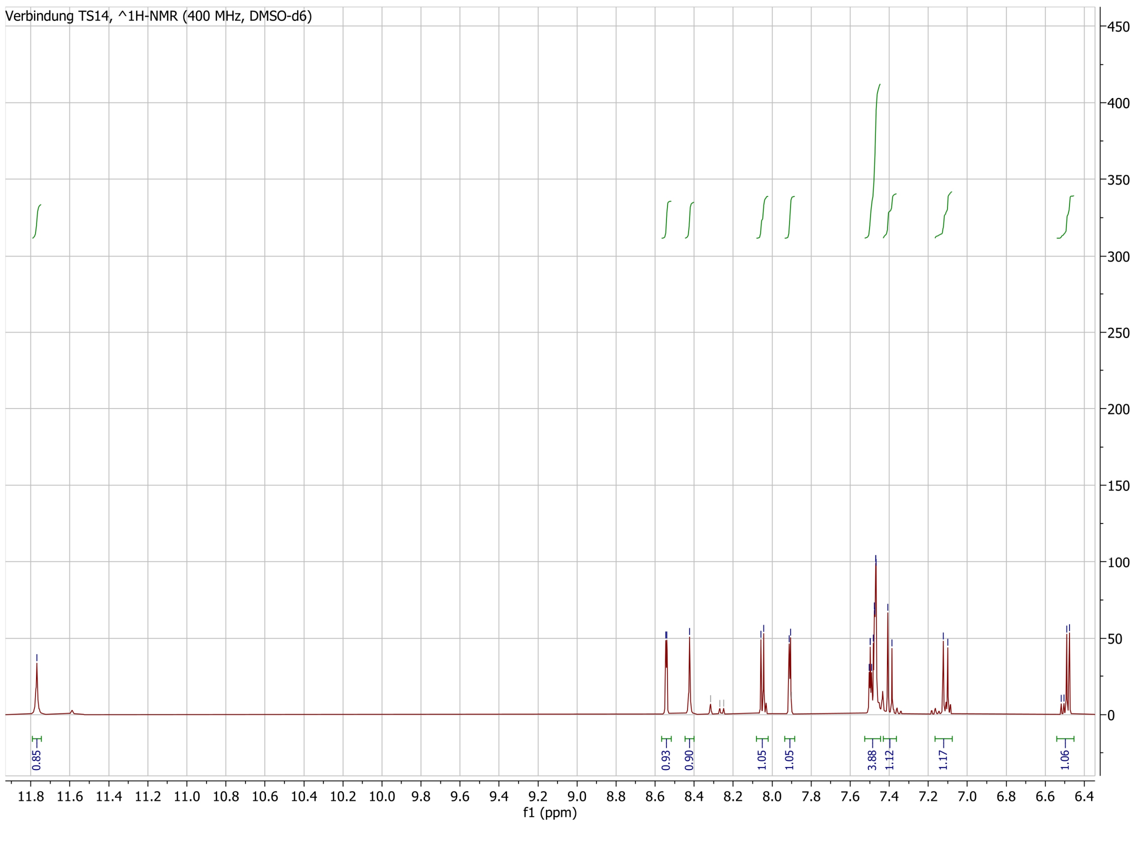

^{1}H NMR spectrum of compound **6h** (full spectrum)

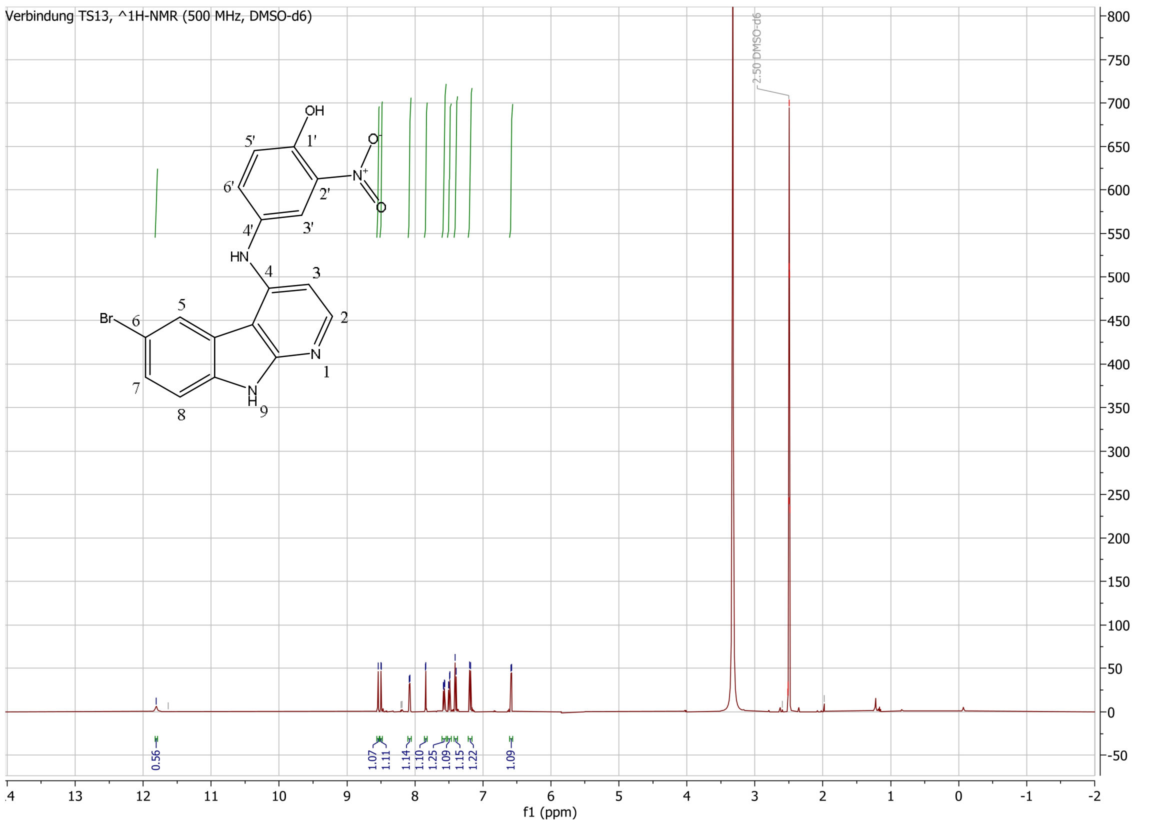

^{1}H NMR spectrum of compound **6h** (expanded region)

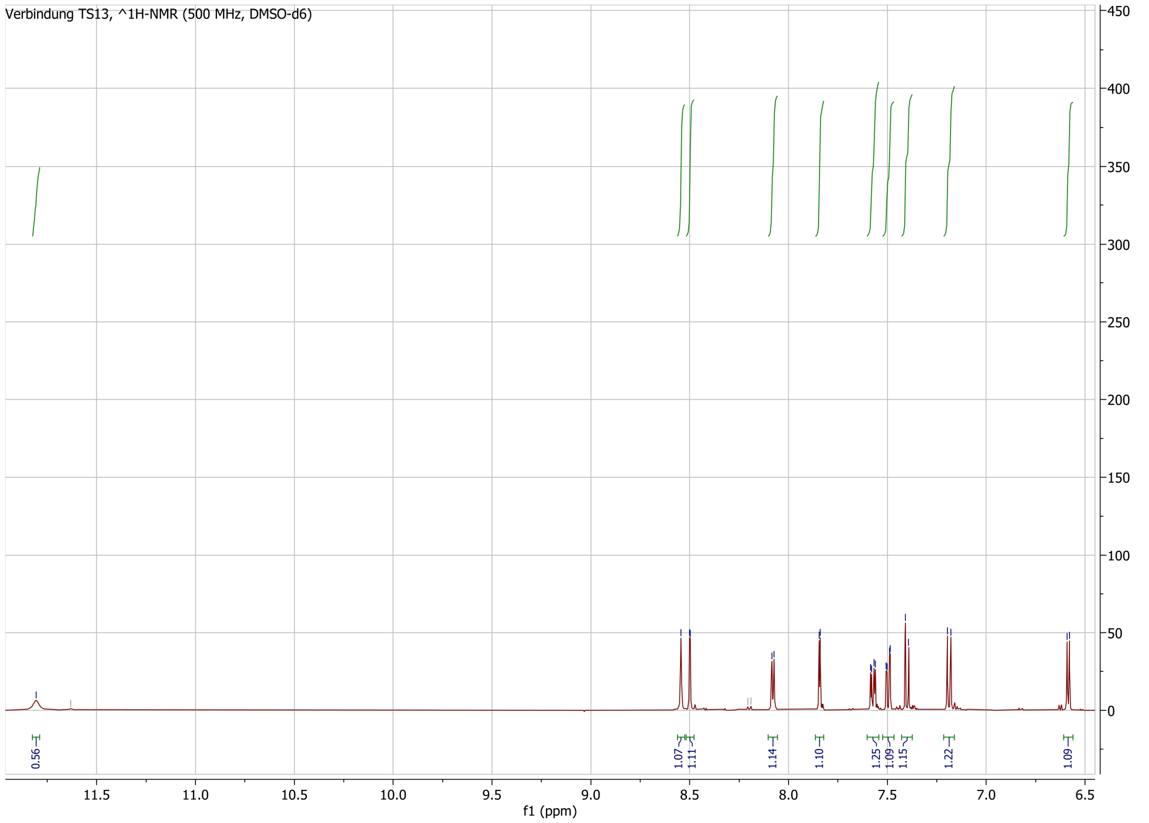

^{1}H NMR spectrum of compound **6i** (full spectrum)

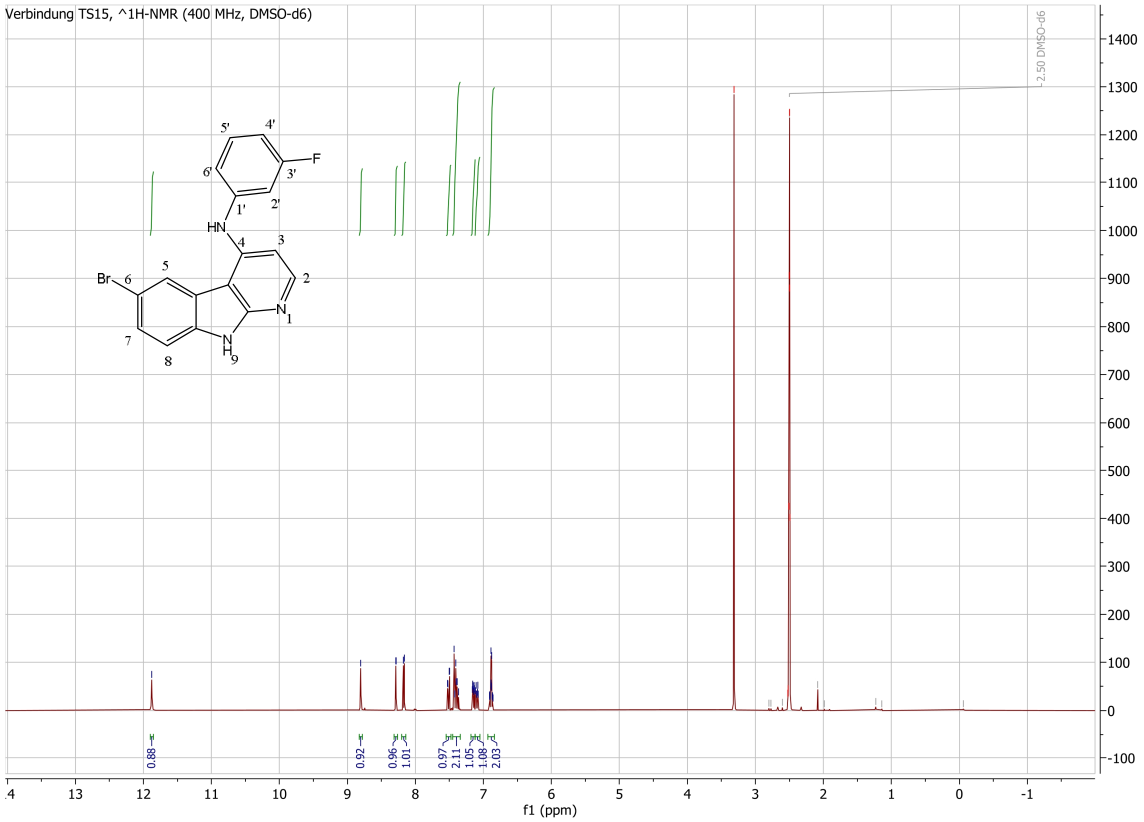

^{1}H NMR spectrum of compound **6i** (expanded region)

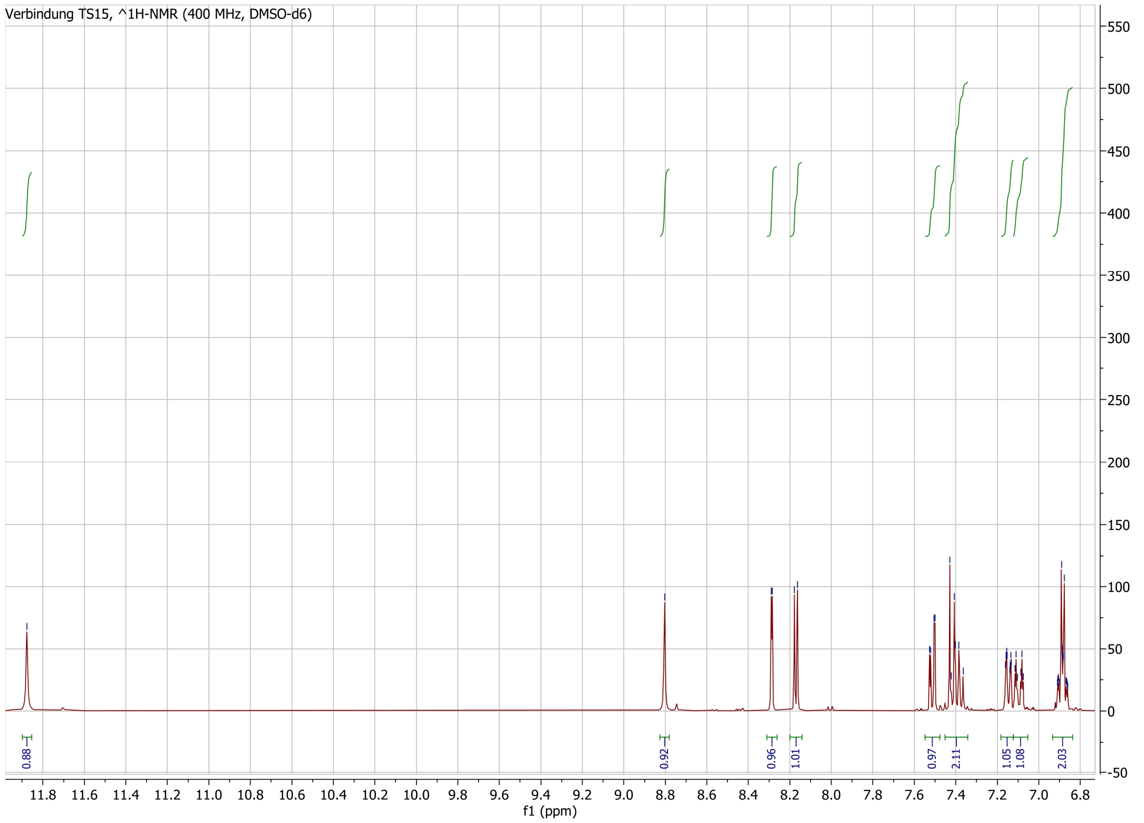

^{1}H NMR spectrum of compound **6j** (full spectrum)

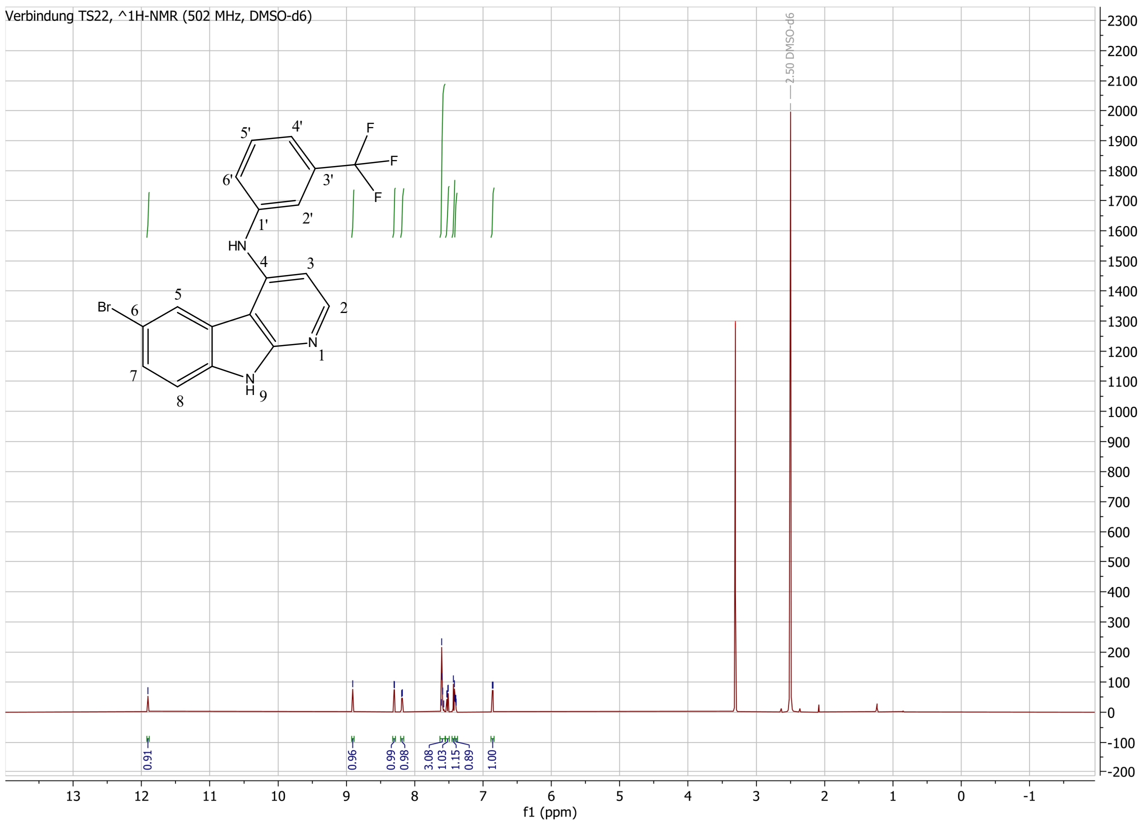

^{1}H NMR spectrum of compound **6j** (expanded region)

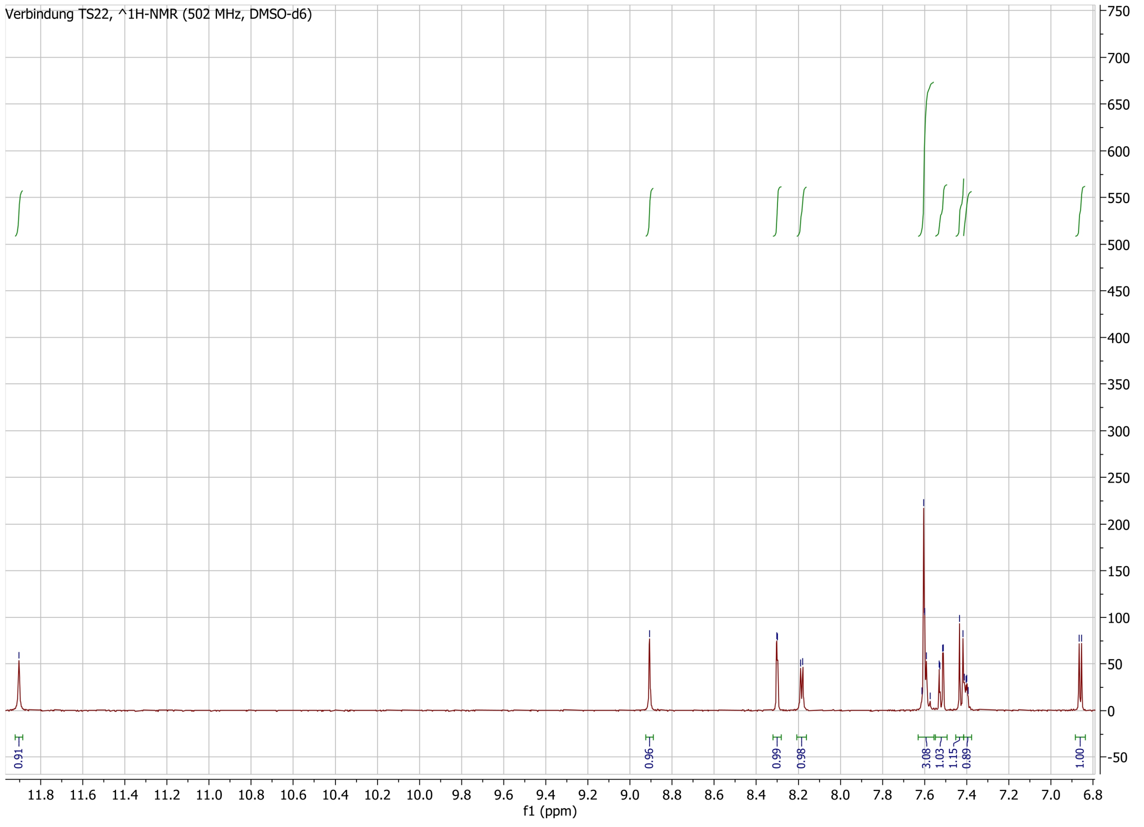

^{1}H NMR spectrum of compound **6k** (full spectrum)

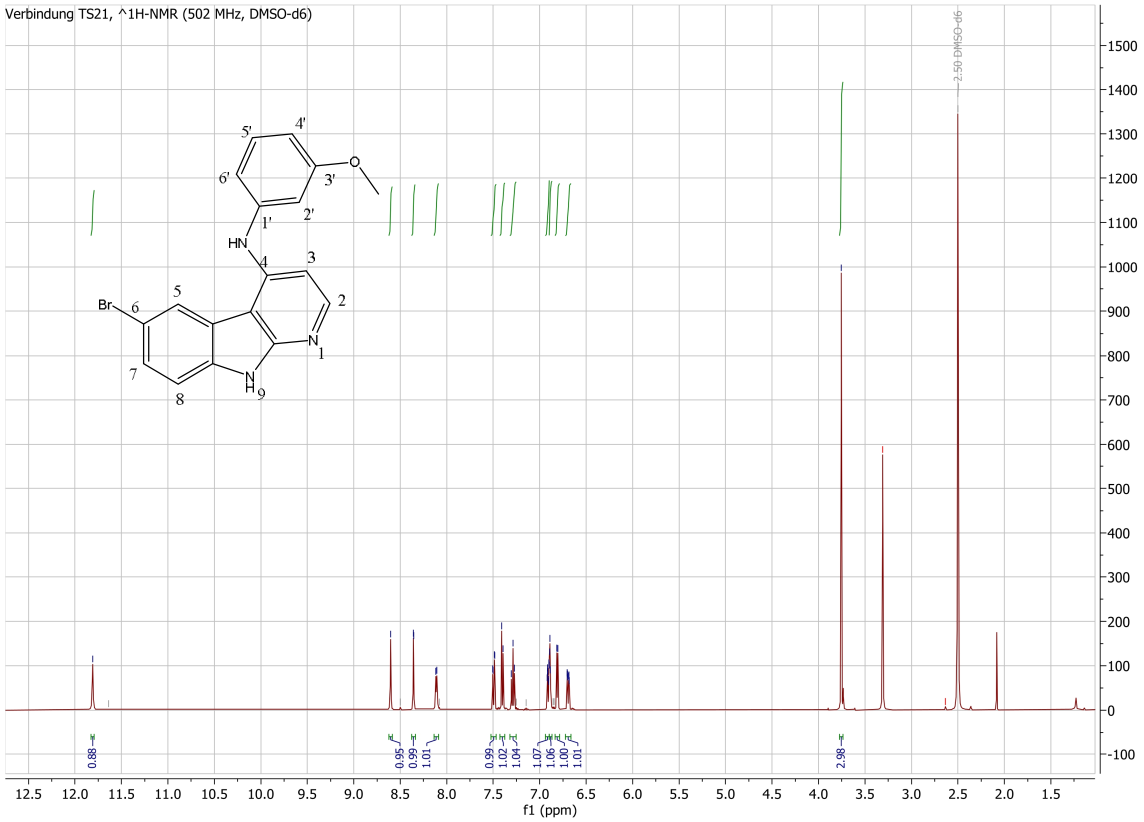

^{1}H NMR spectrum of compound **6k** (expanded region)

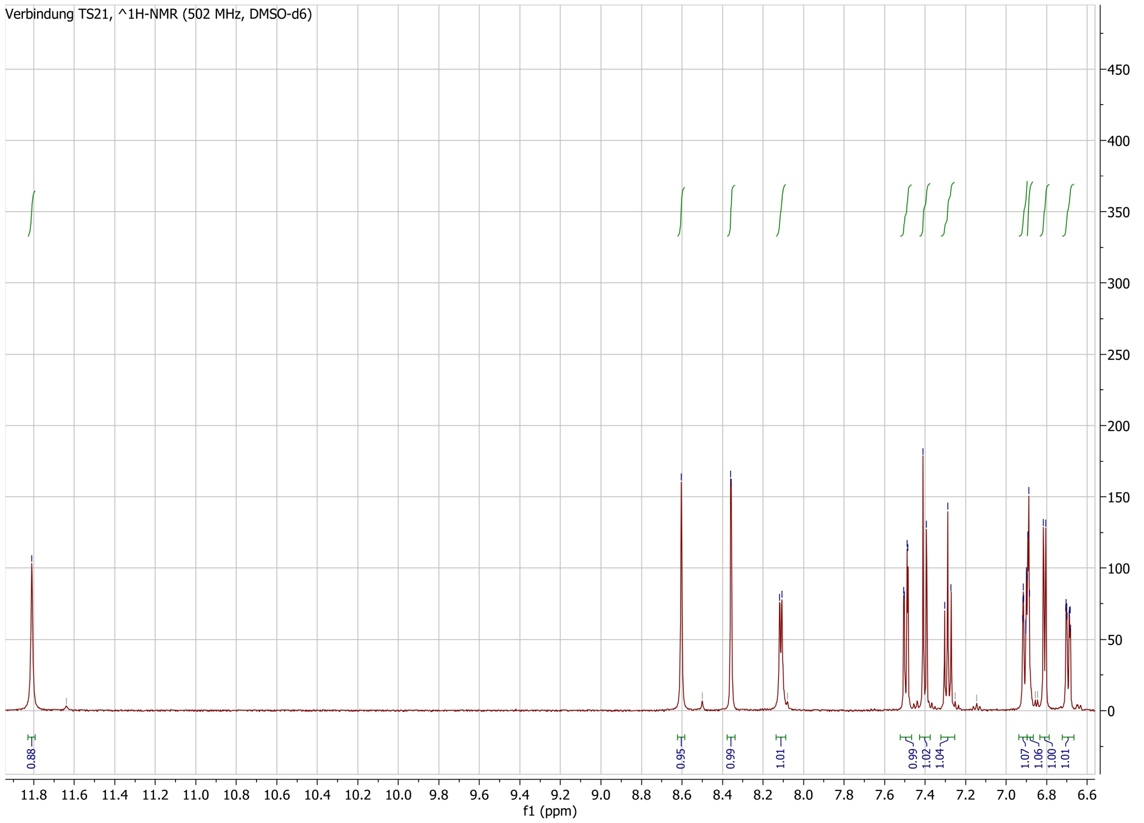

^{1}H NMR spectrum of compound **6l** (full spectrum)

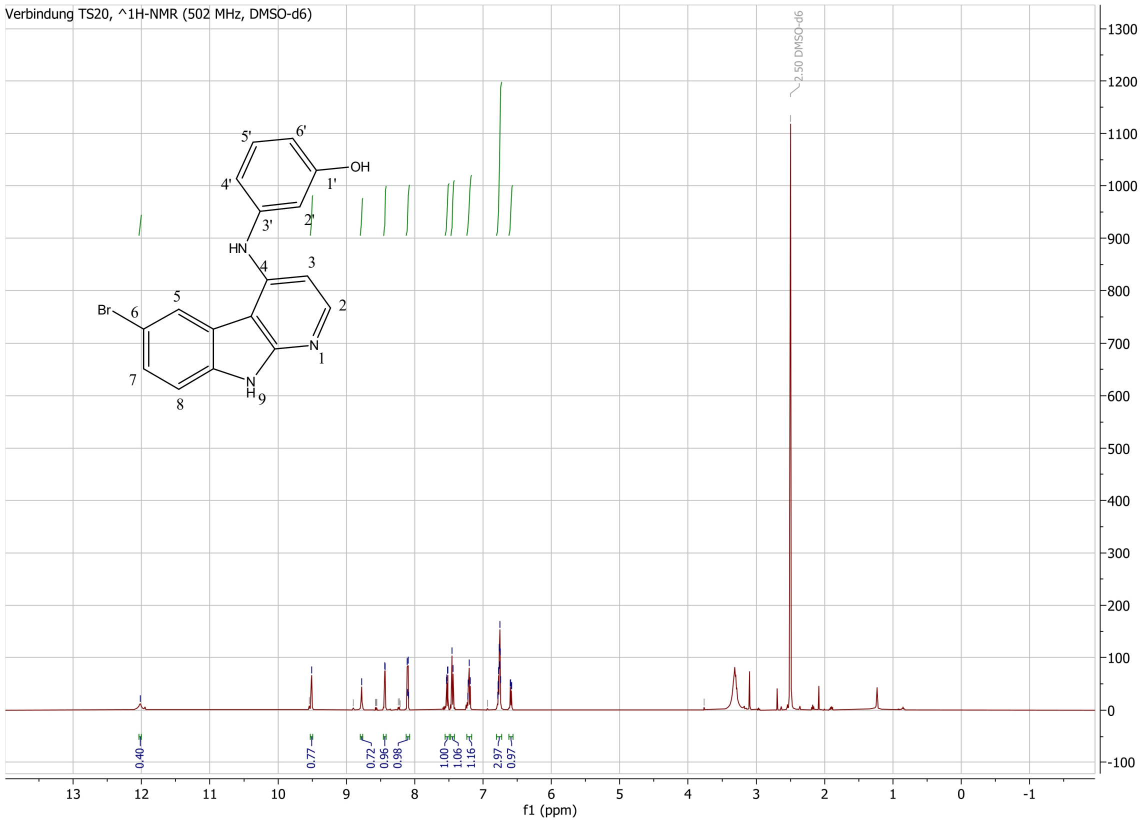

^{1}H NMR spectrum of compound **6l** (expanded region)

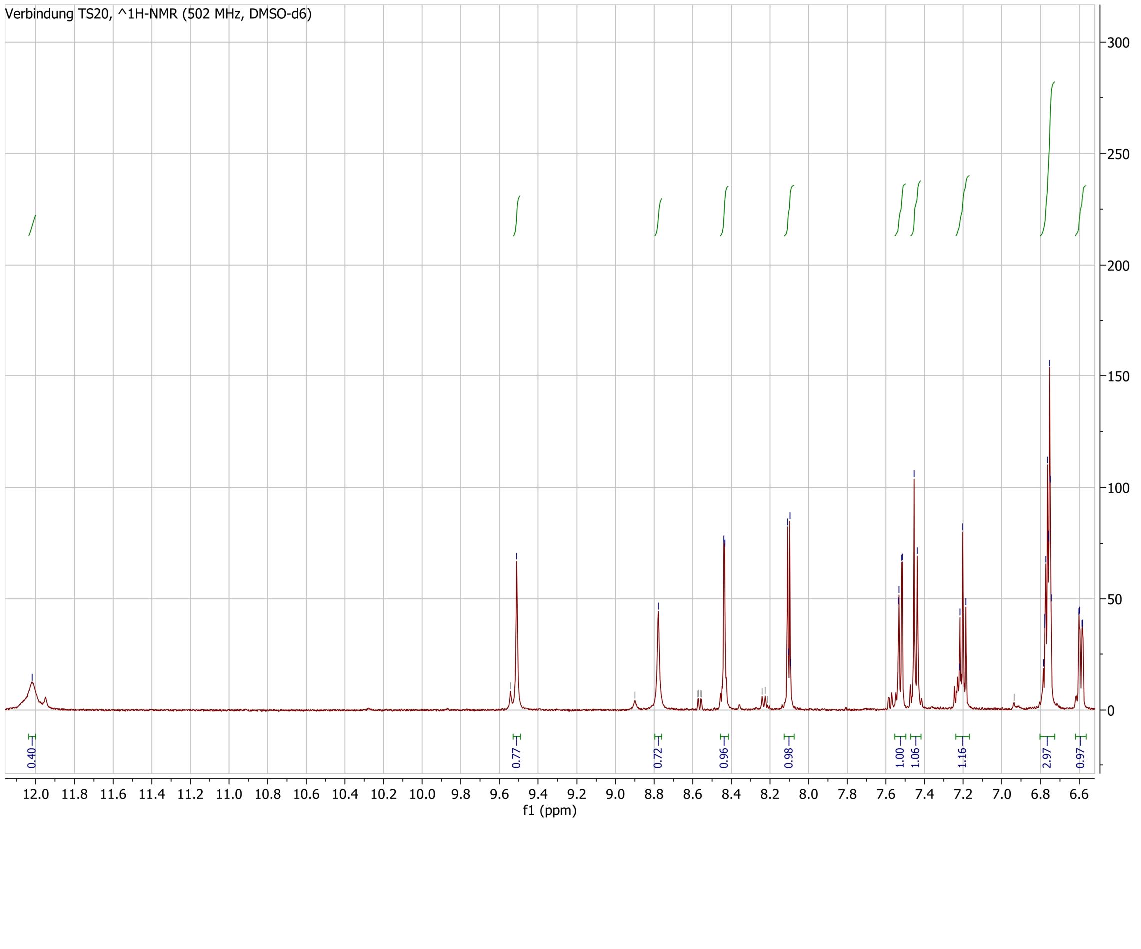

^{1}H NMR spectrum of compound **6m** (full spectrum)

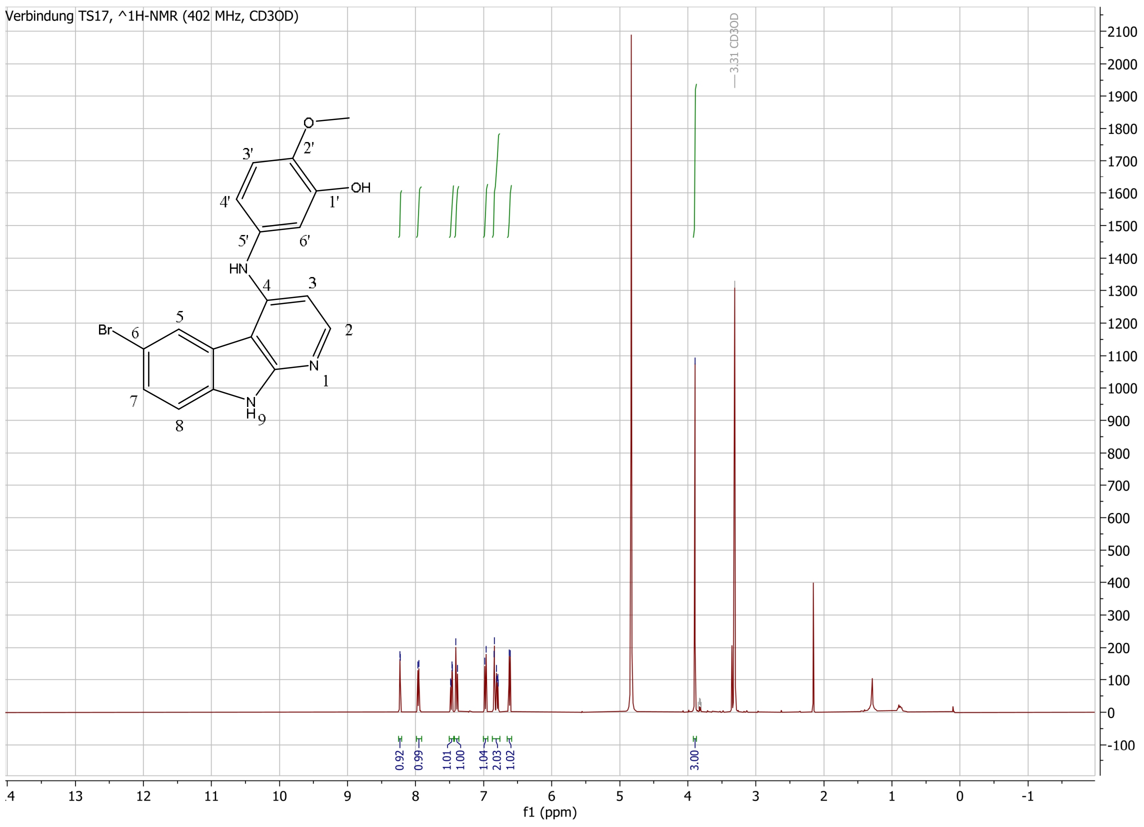

^{1}H NMR spectrum of compound **6m** (expanded region)

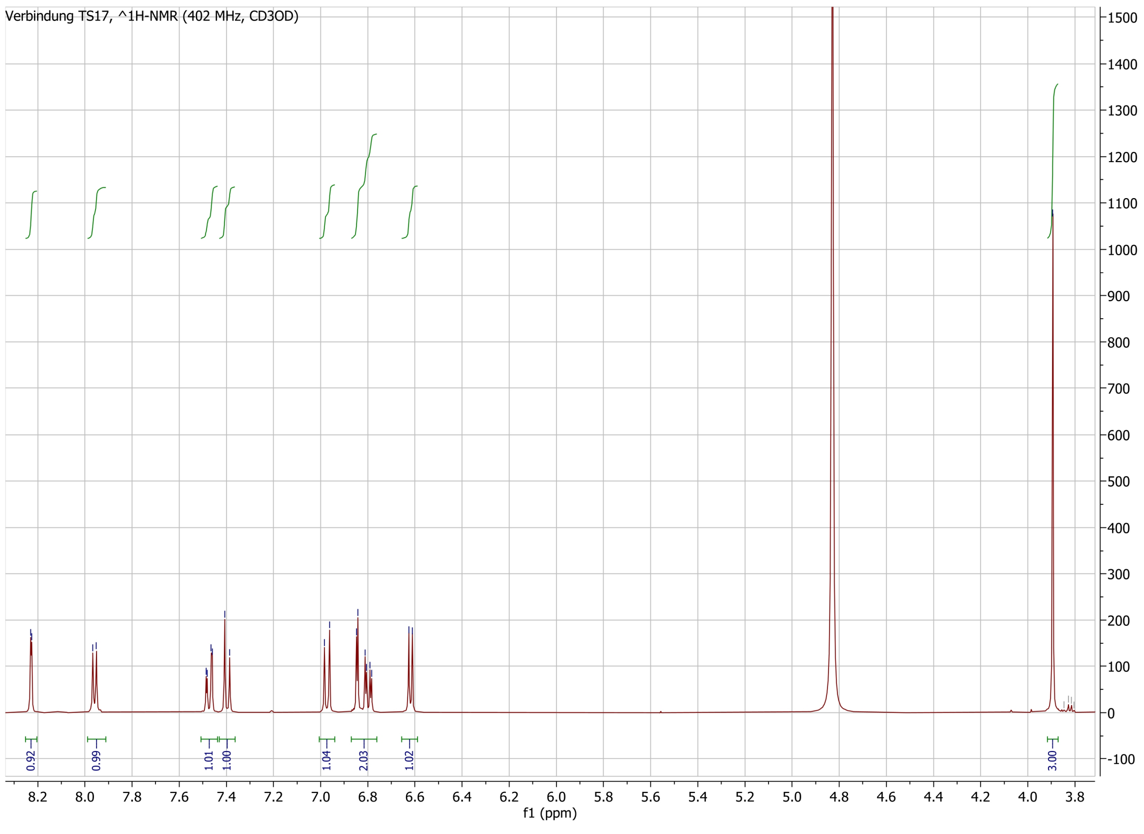

^{1}H NMR spectrum of compound **8a** (full spectrum)

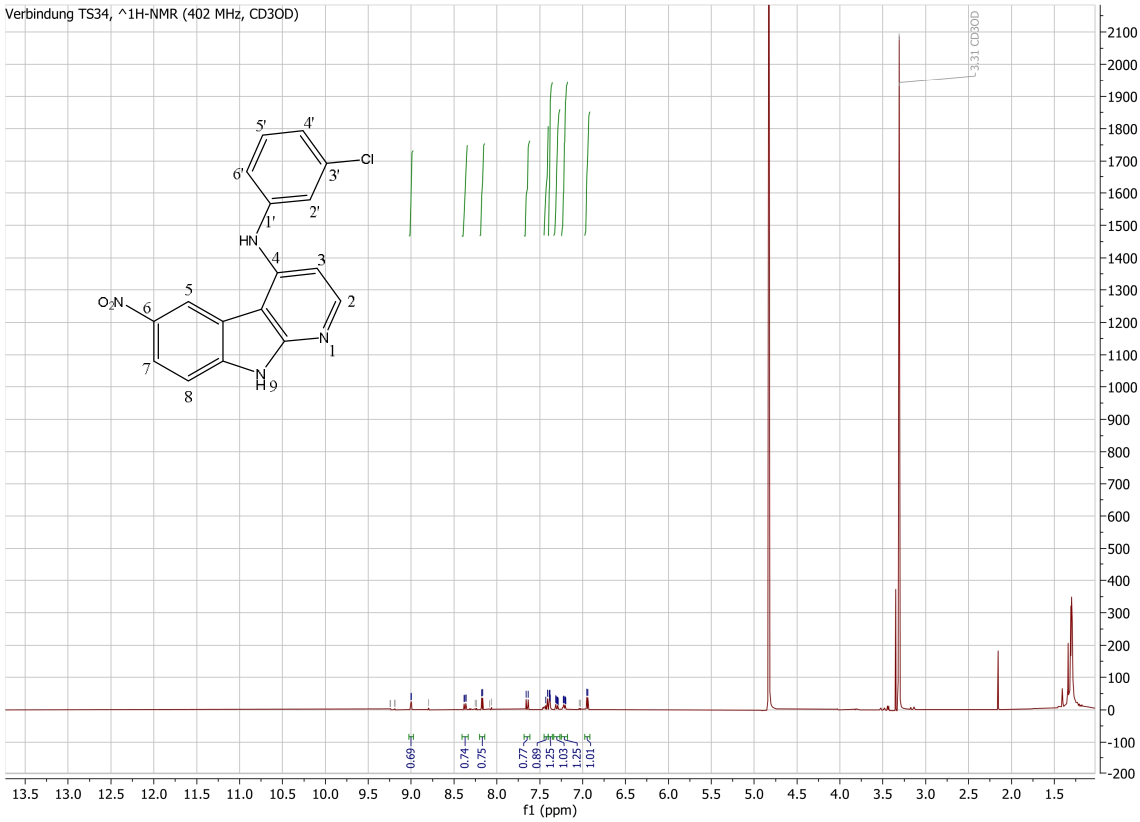

^{1}H NMR spectrum of compound **8a** (expanded region)

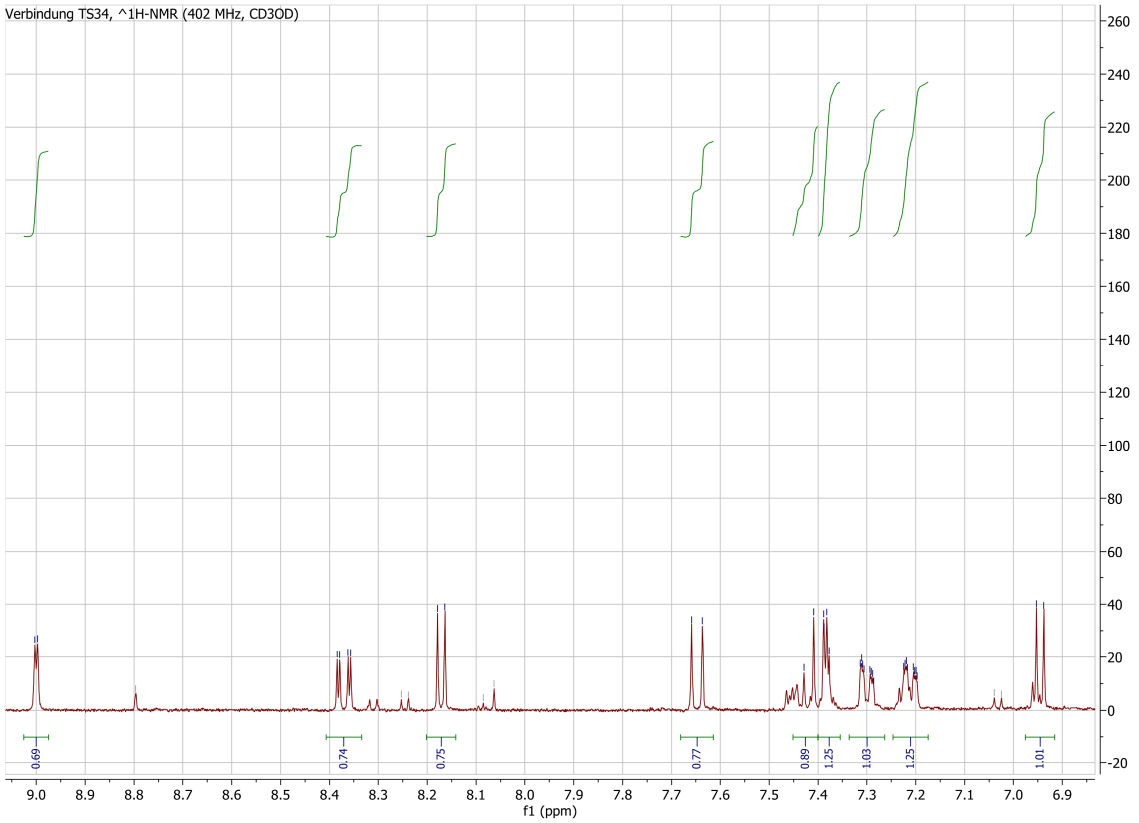

^{1}H NMR spectrum of compound **8b** (full spectrum)

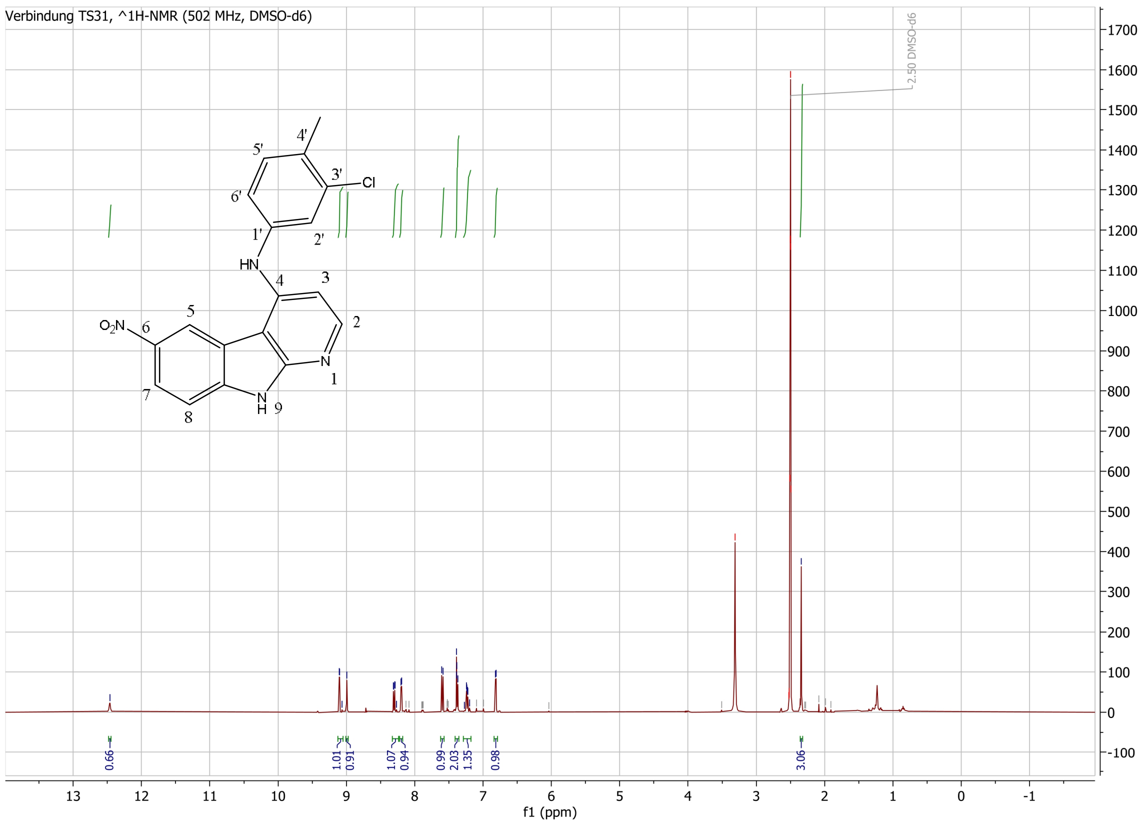

^{1}H NMR spectrum of compound **8b** (expanded region)

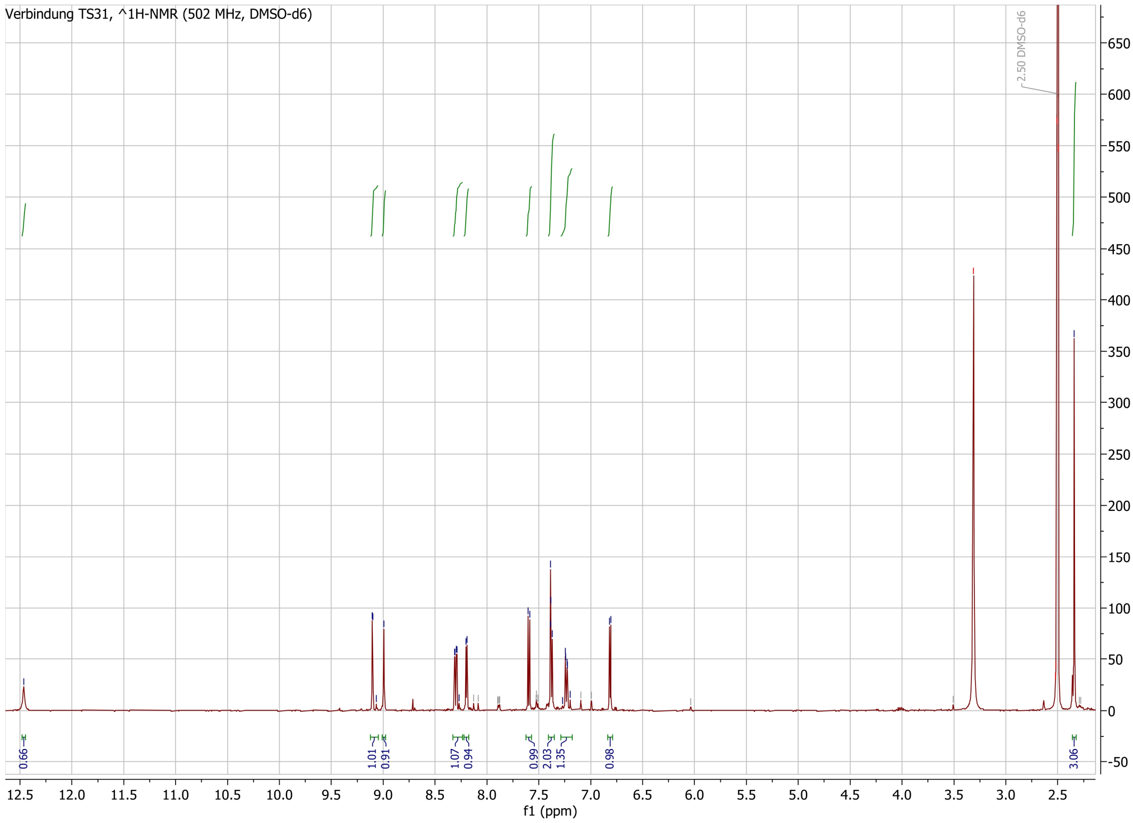

^{1}H NMR spectrum of compound **8c** (full spectrum)

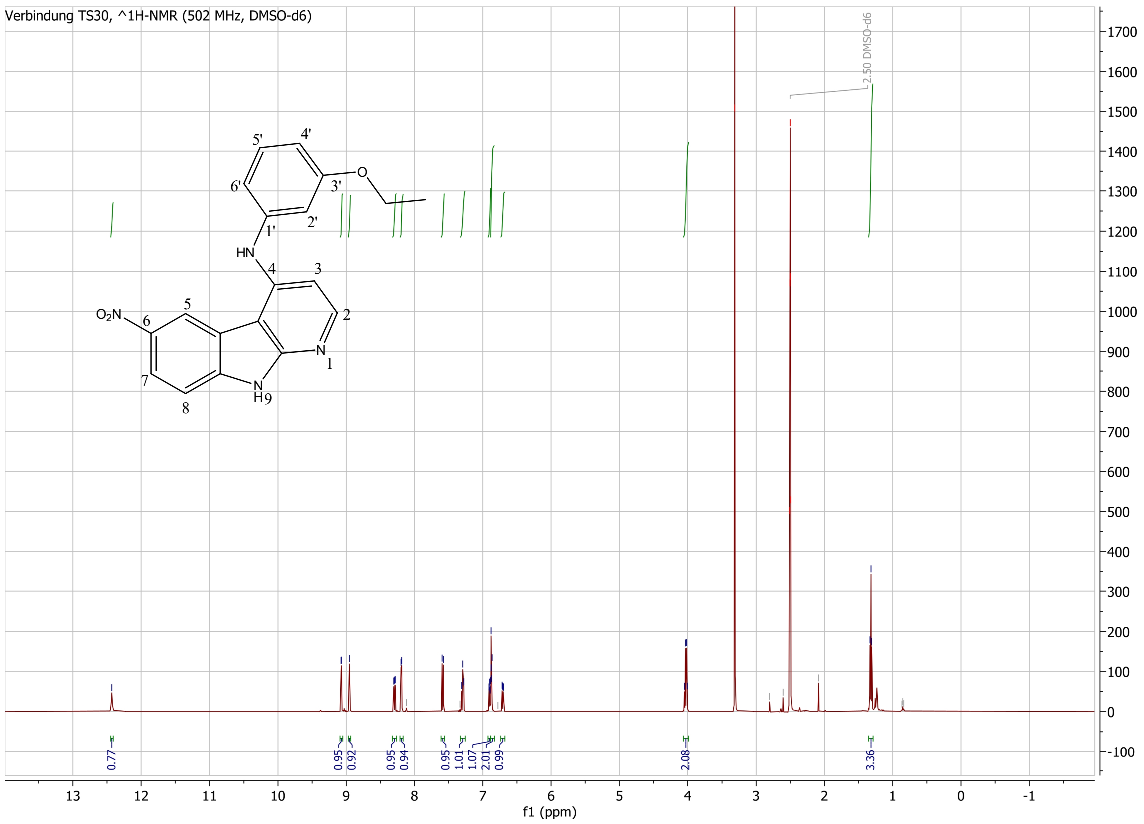

^{1}H NMR spectrum of compound **8c** (expanded region)

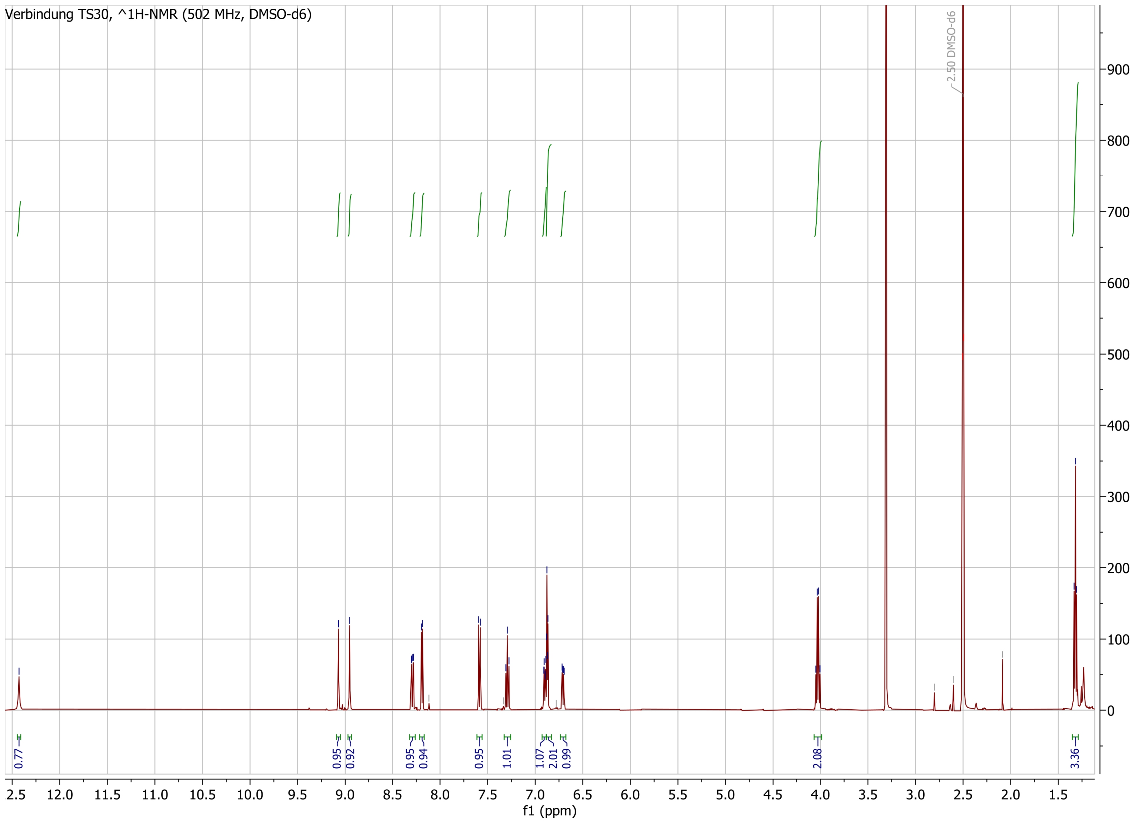

^{1}H NMR spectrum of compound **8d** (full spectrum)

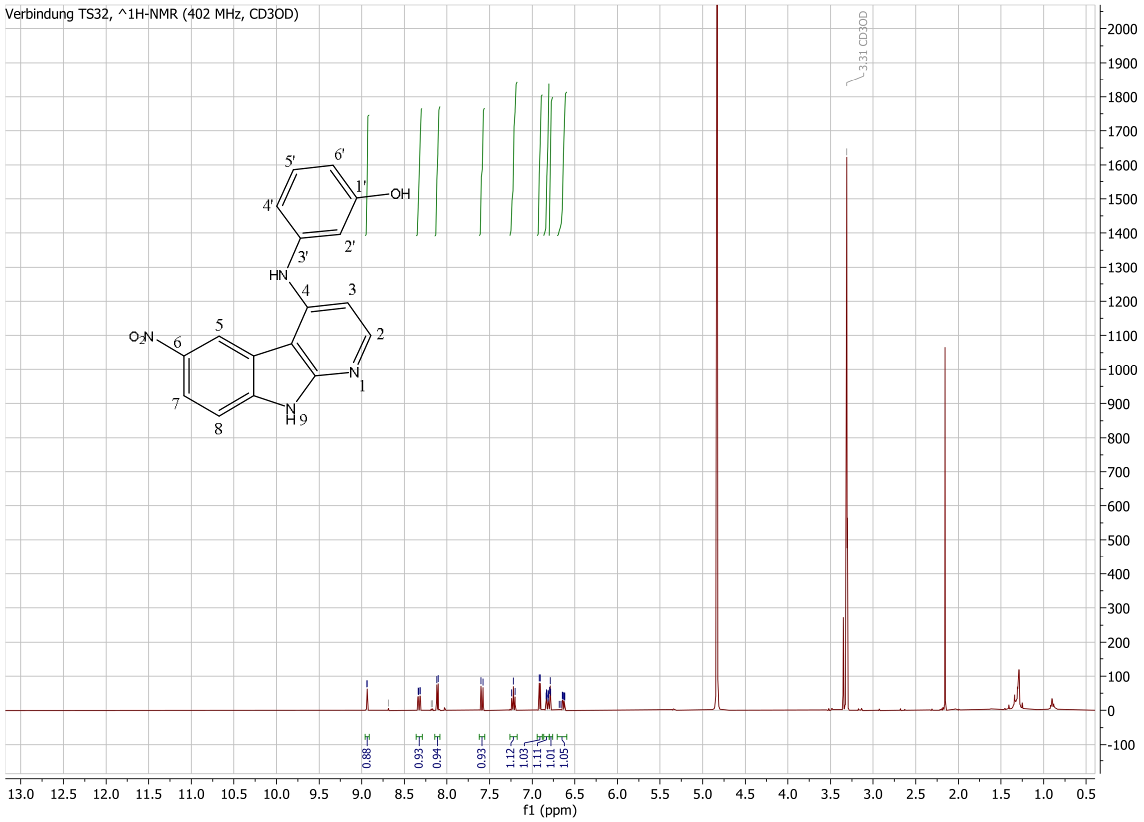

^{1}H NMR spectrum of compound **8d** (expanded region)

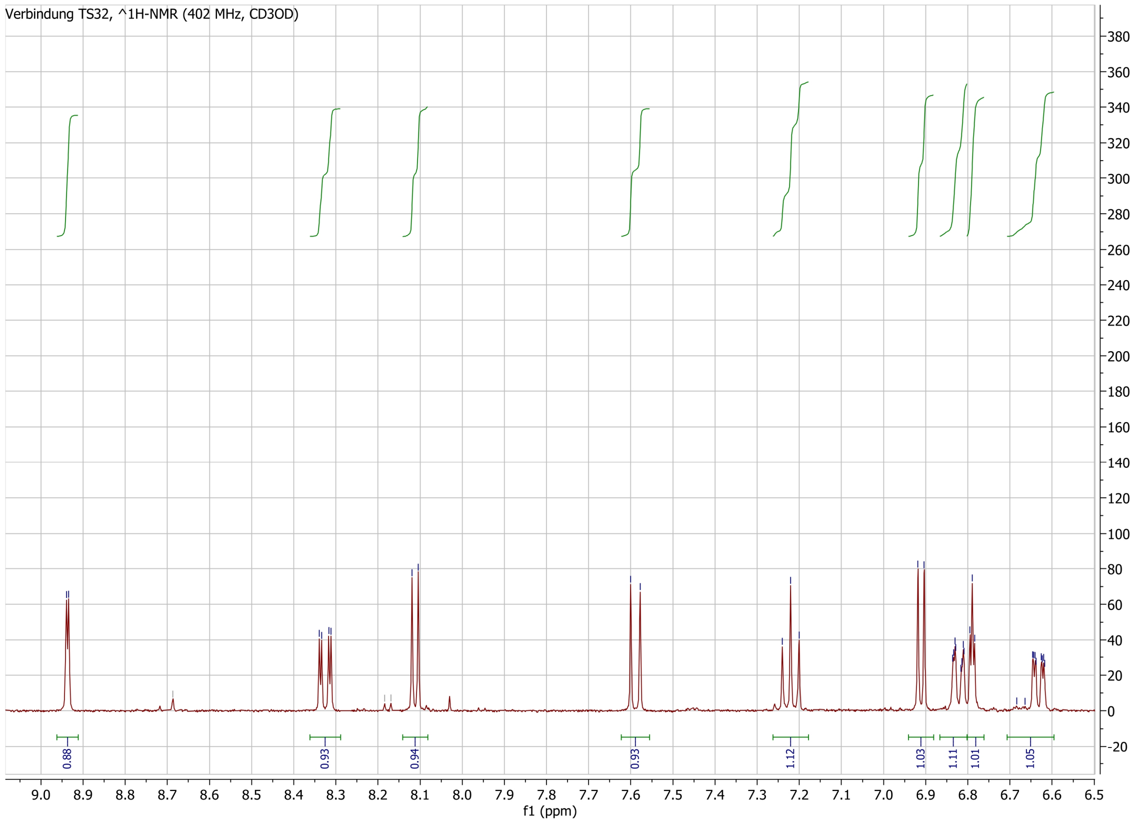

^{1}H NMR spectrum of compound **8e** (full spectrum)

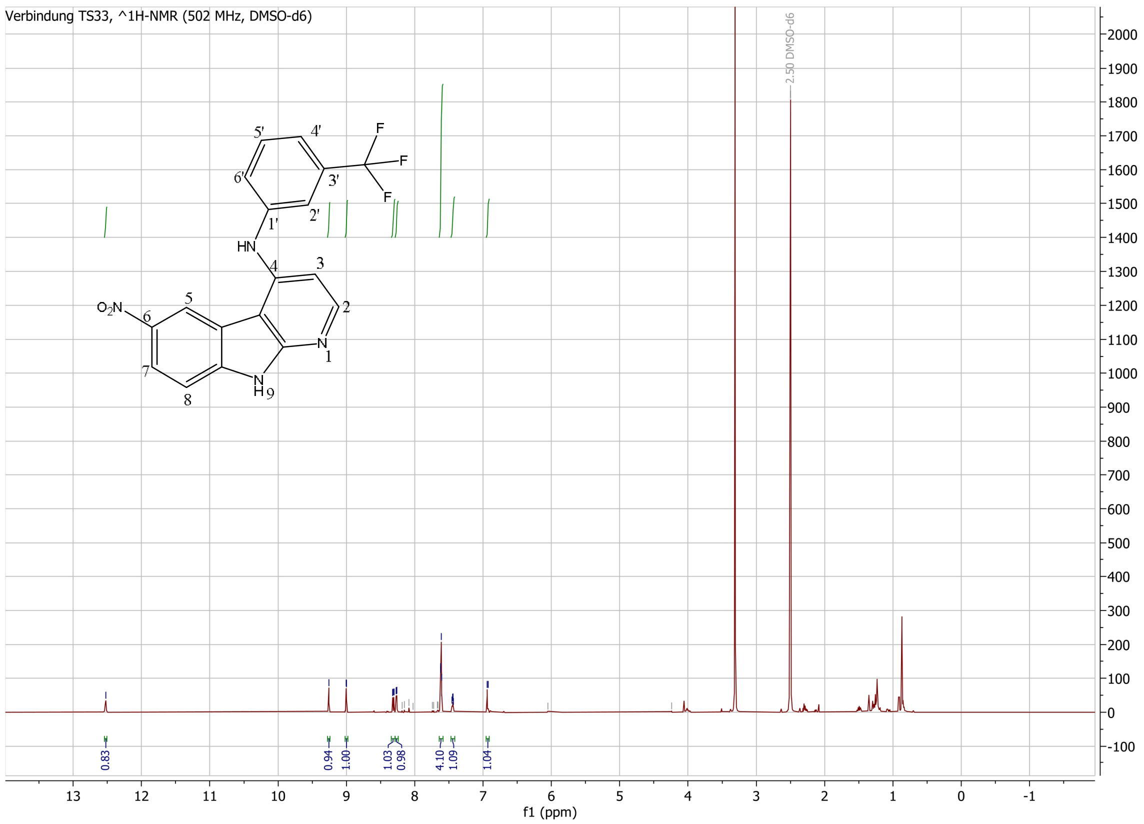

^{1}H NMR spectrum of compound **8e** (expanded region)

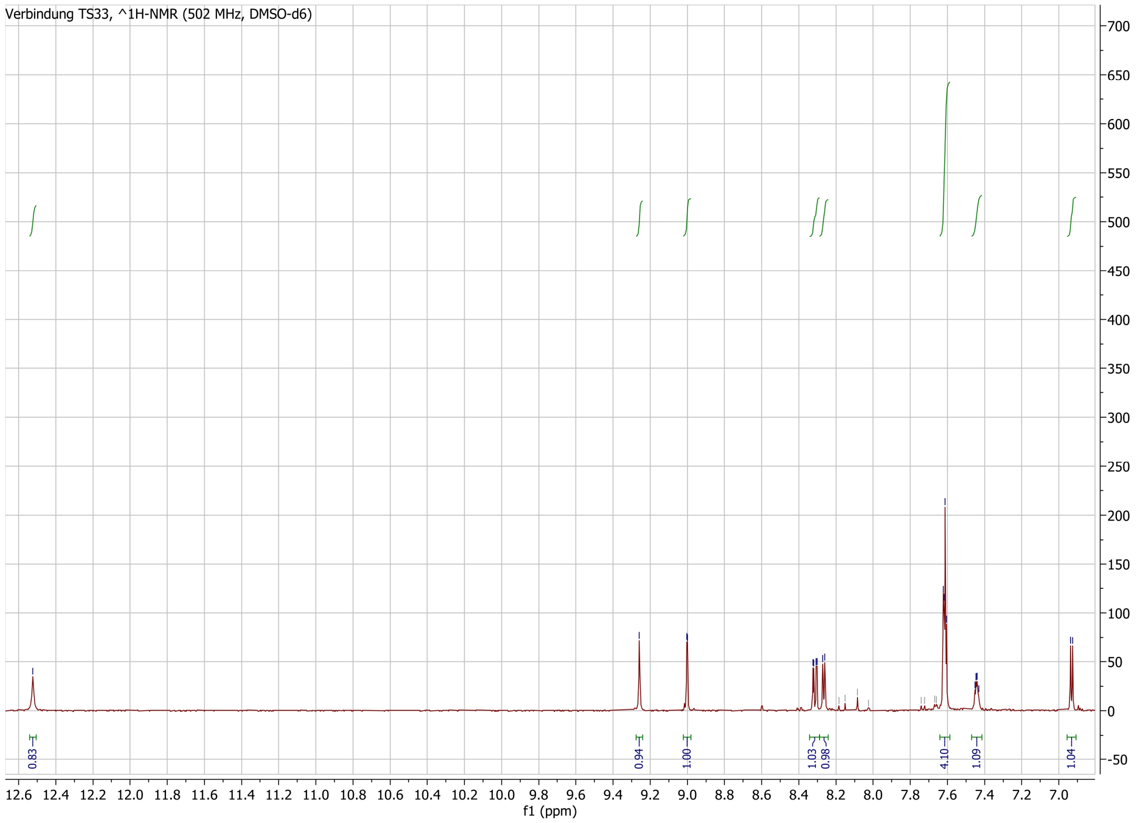

^{1}H NMR spectrum of compound **9a** (full spectrum)

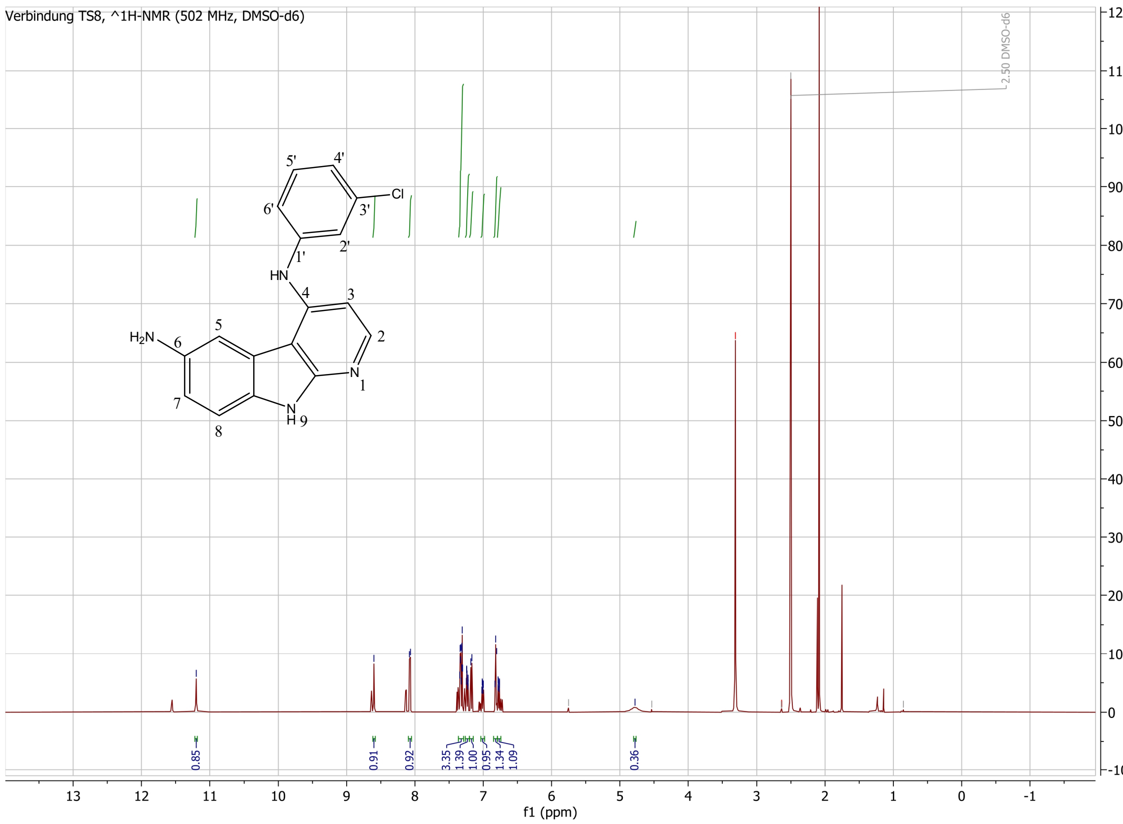

^{1}H NMR spectrum of compound **9a** (expanded region)

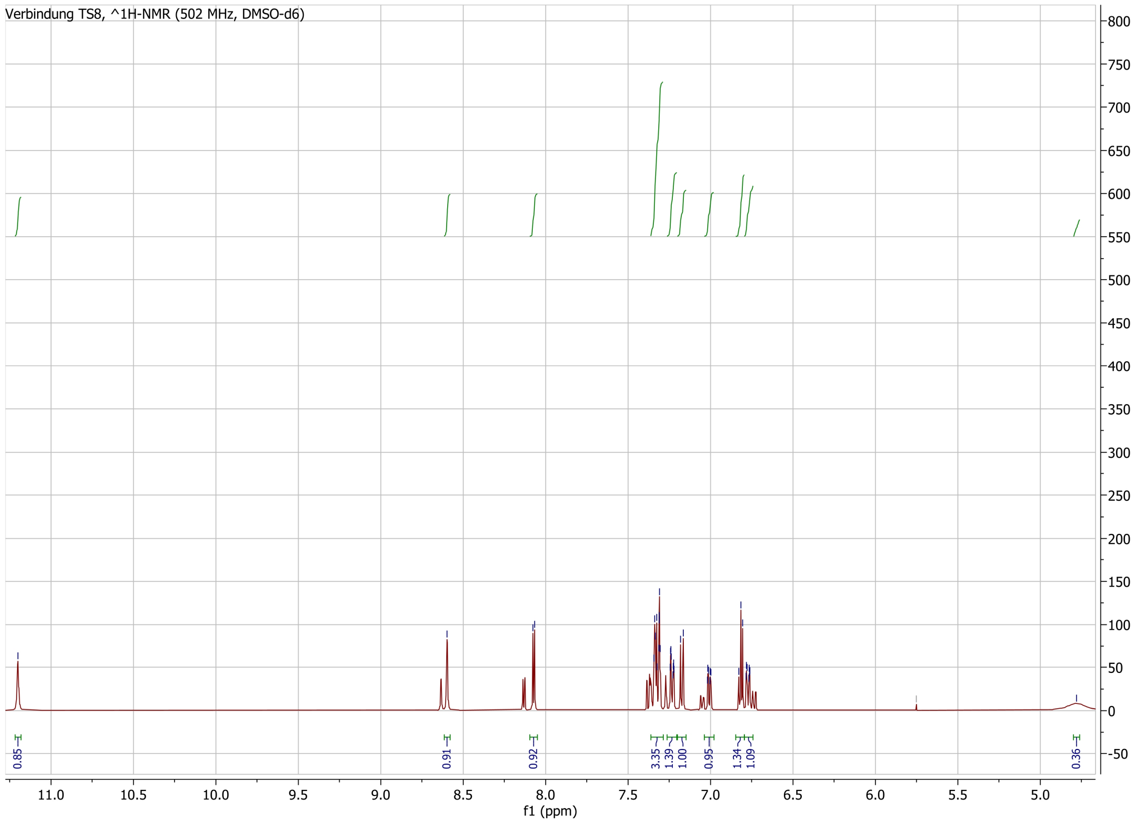

^{1}H NMR spectrum of compound **9b** (full spectrum)

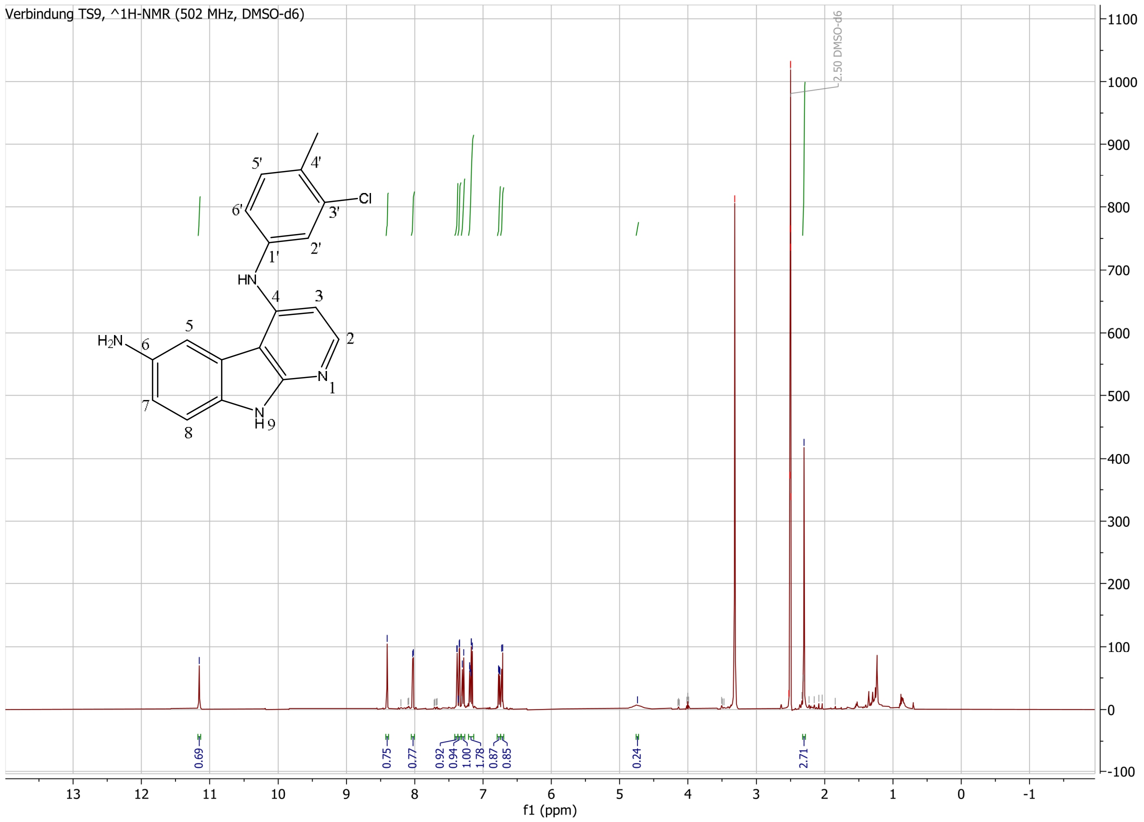

^{1}H NMR spectrum of compound **9b** (expanded region)

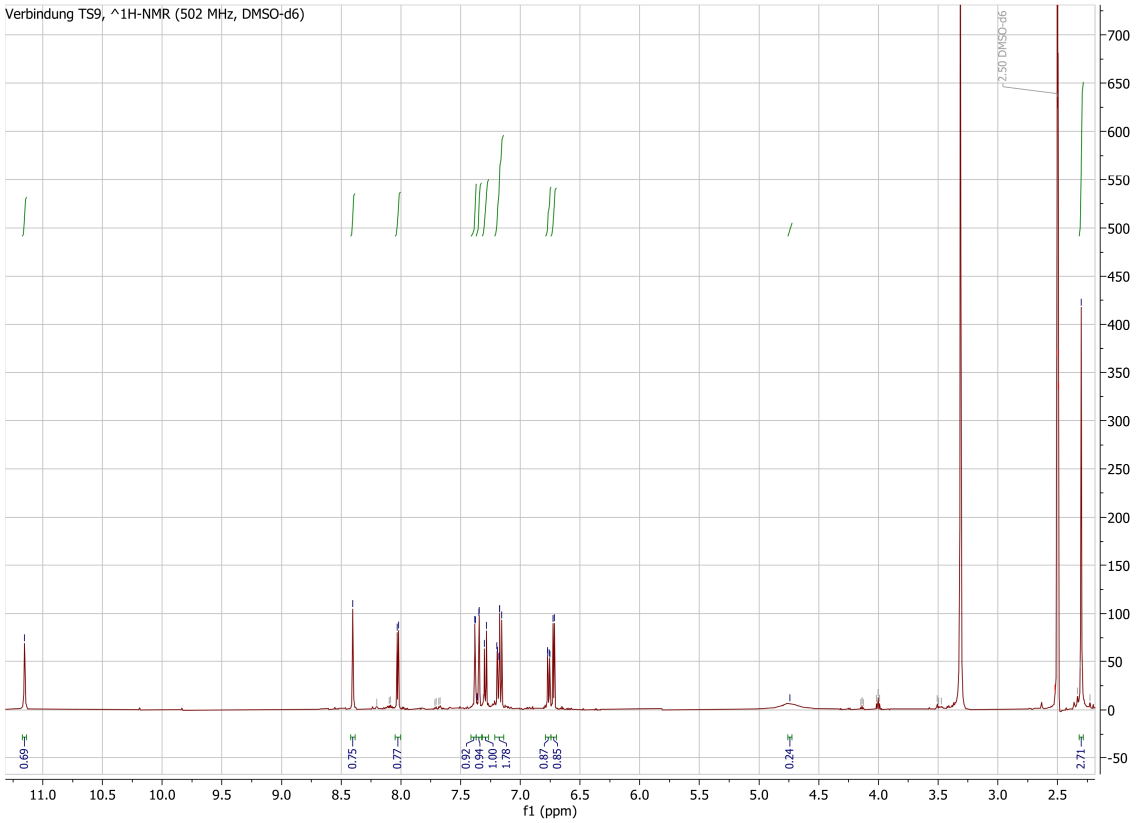

^{1}H NMR spectrum of compound **9c** (full spectrum)

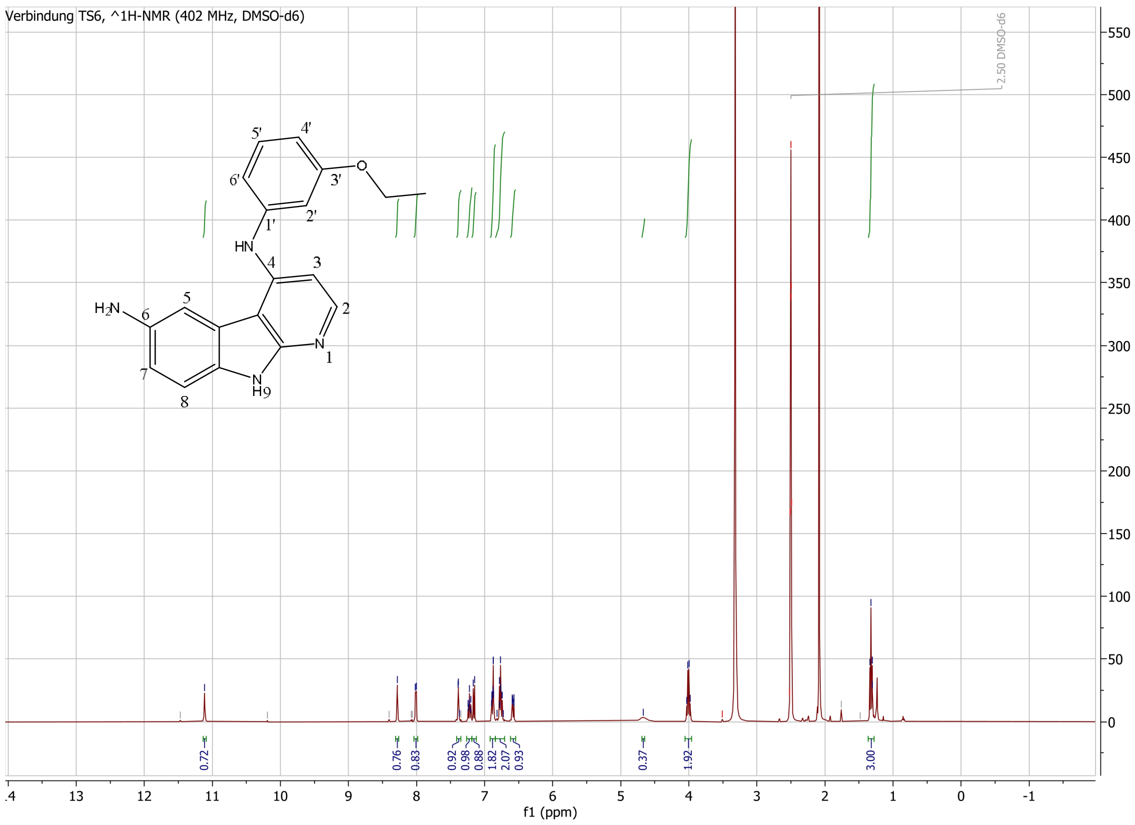

^{1}H NMR spectrum of compound **9c** (expanded region)

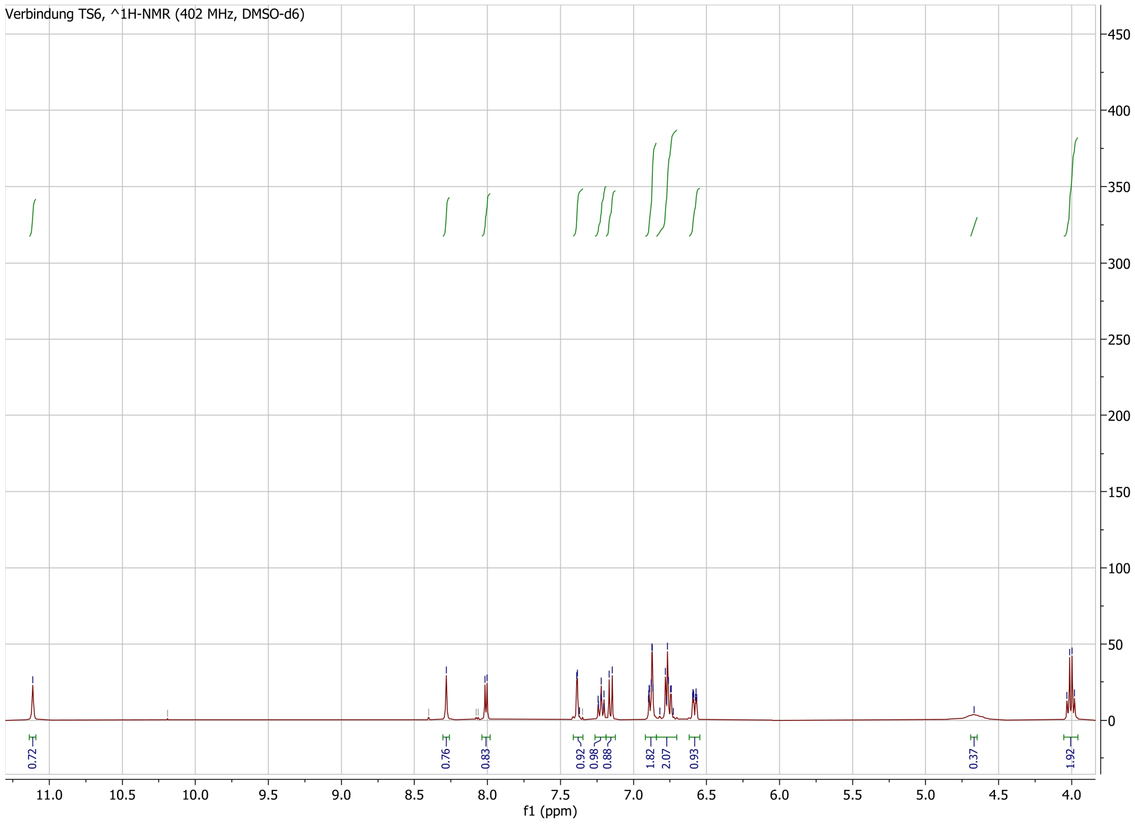

^{1}H NMR spectrum of compound **9d** (full spectrum)

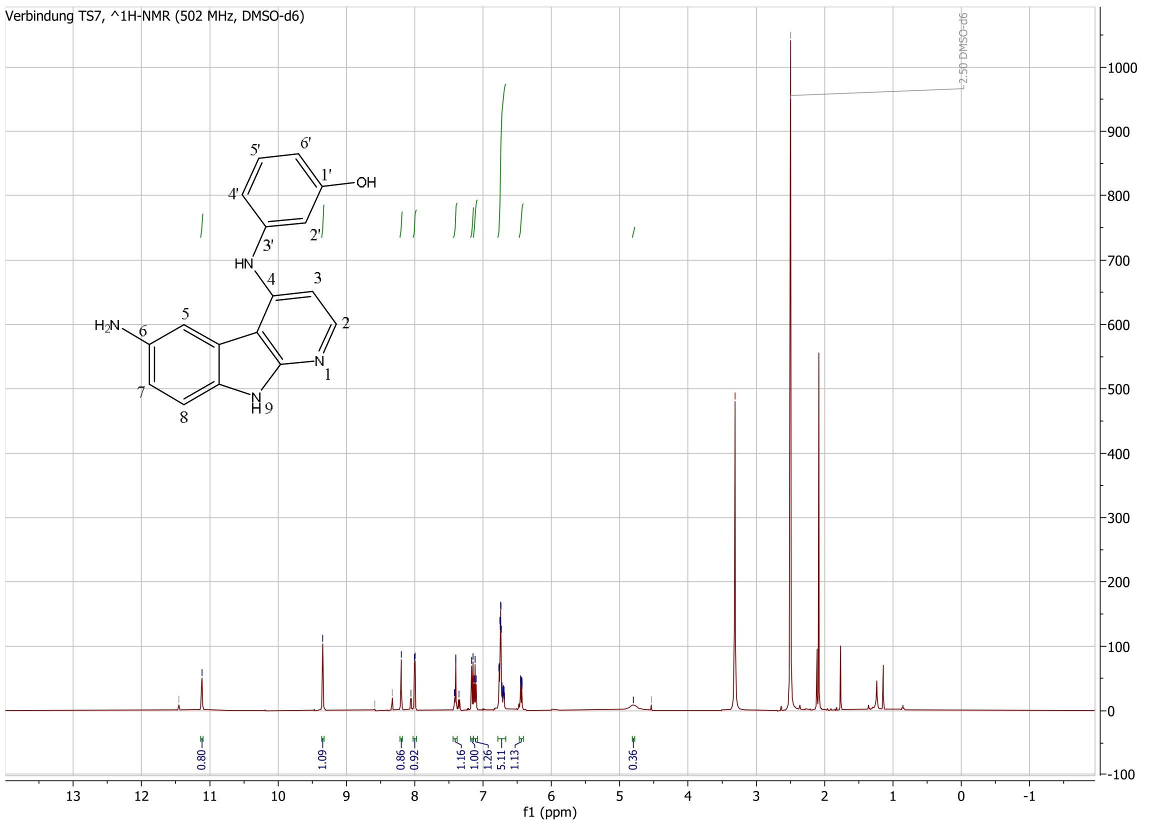

^{1}H NMR spectrum of compound **9d** (expanded region)

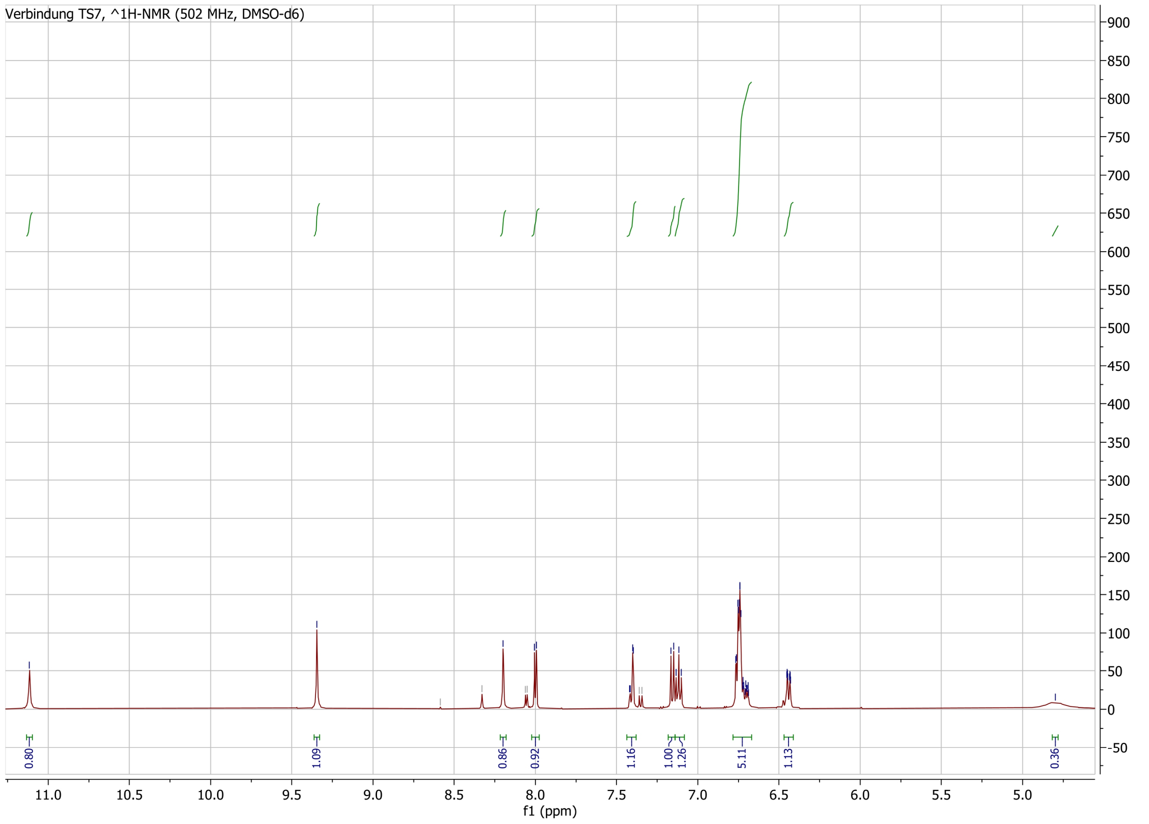

HRMS spectrum of compound **2a**

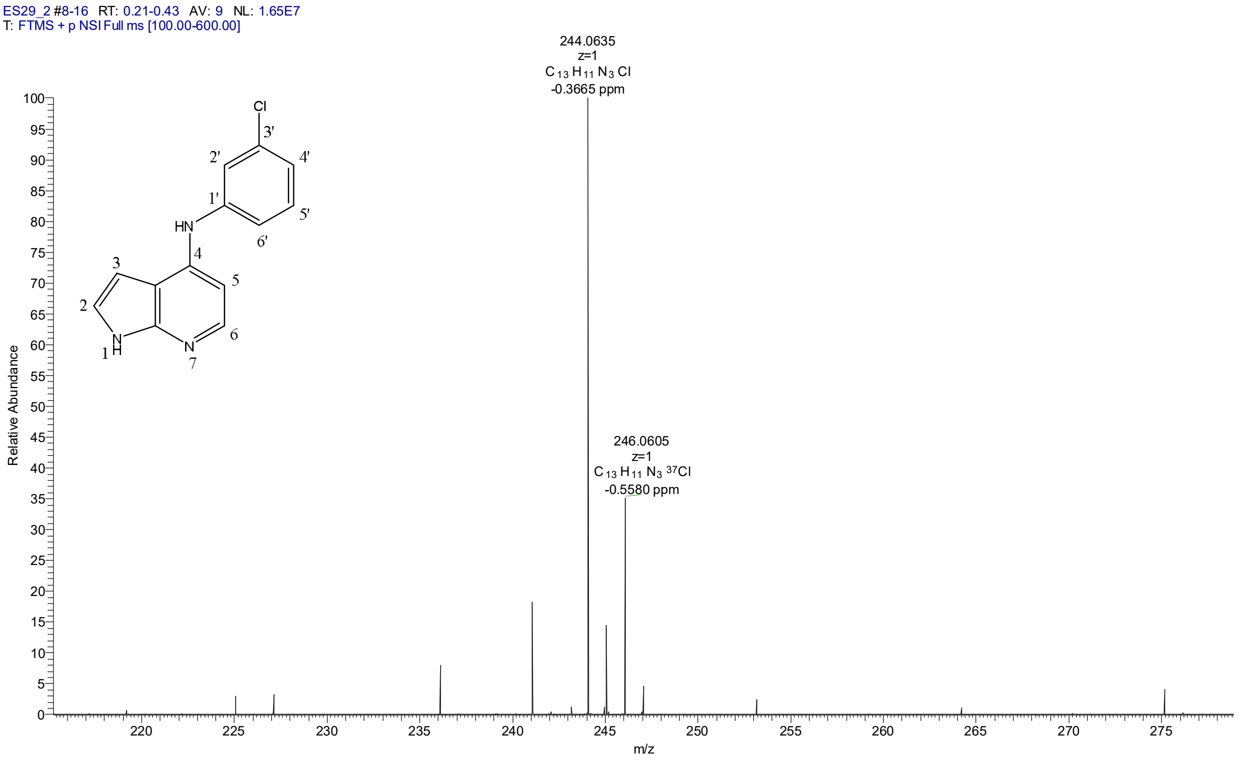

HRMS spectrum of compound **2b**

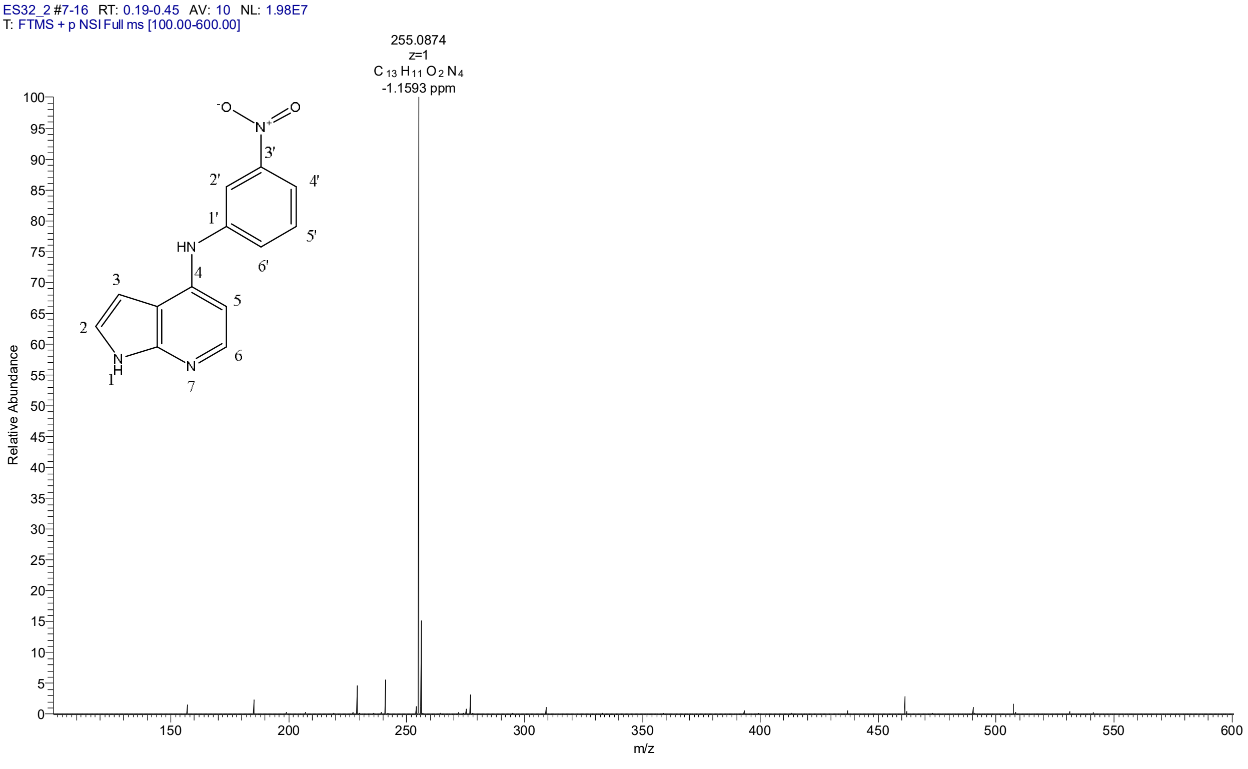

HRMS spectrum of compound **2c**

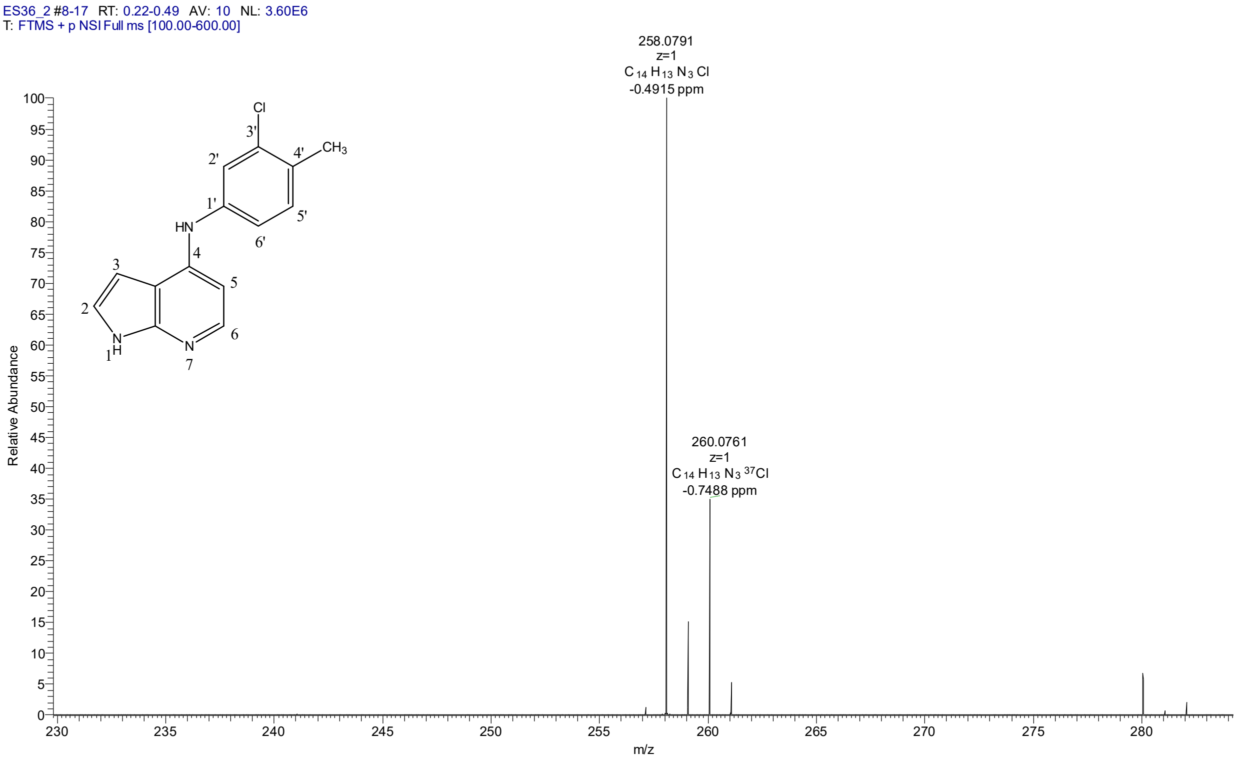

HRMS spectrum of compound **2d**

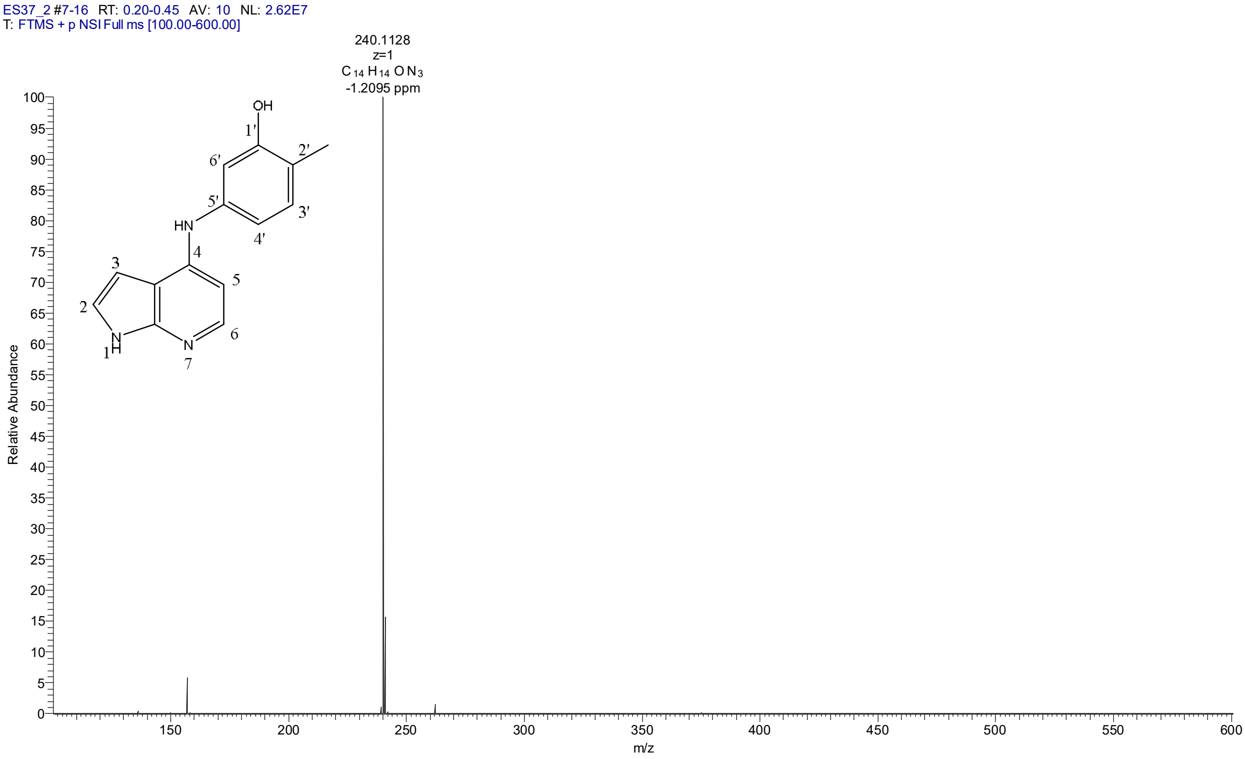

HRMS spectrum of compound **6a**

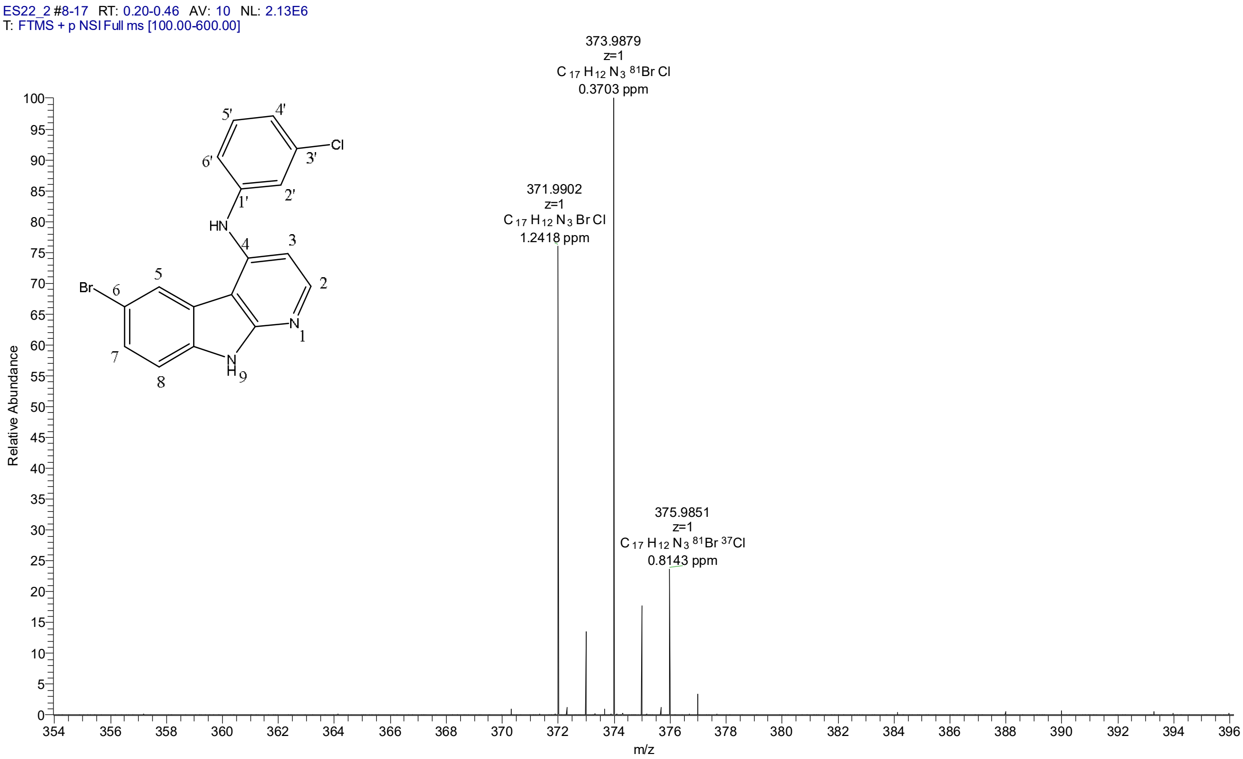

HRMS spectrum of compound **6b**

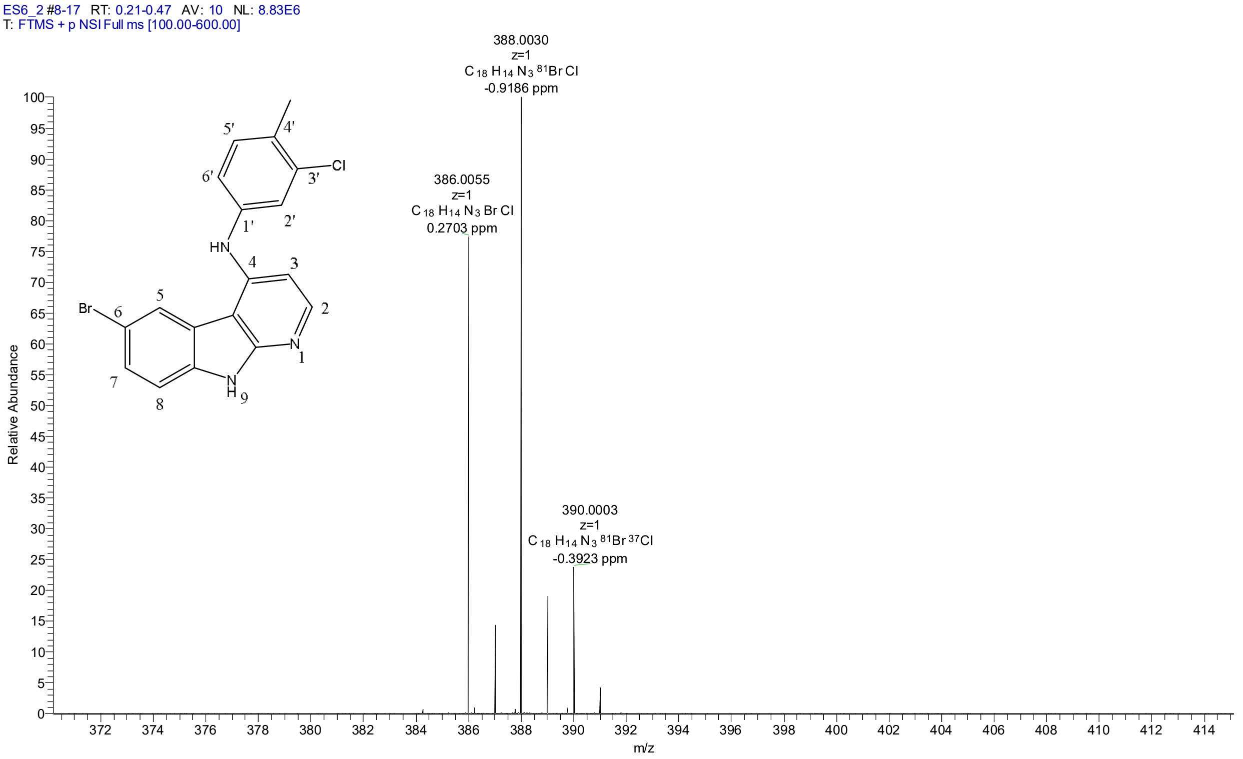

HRMS spectrum of compound **6c**

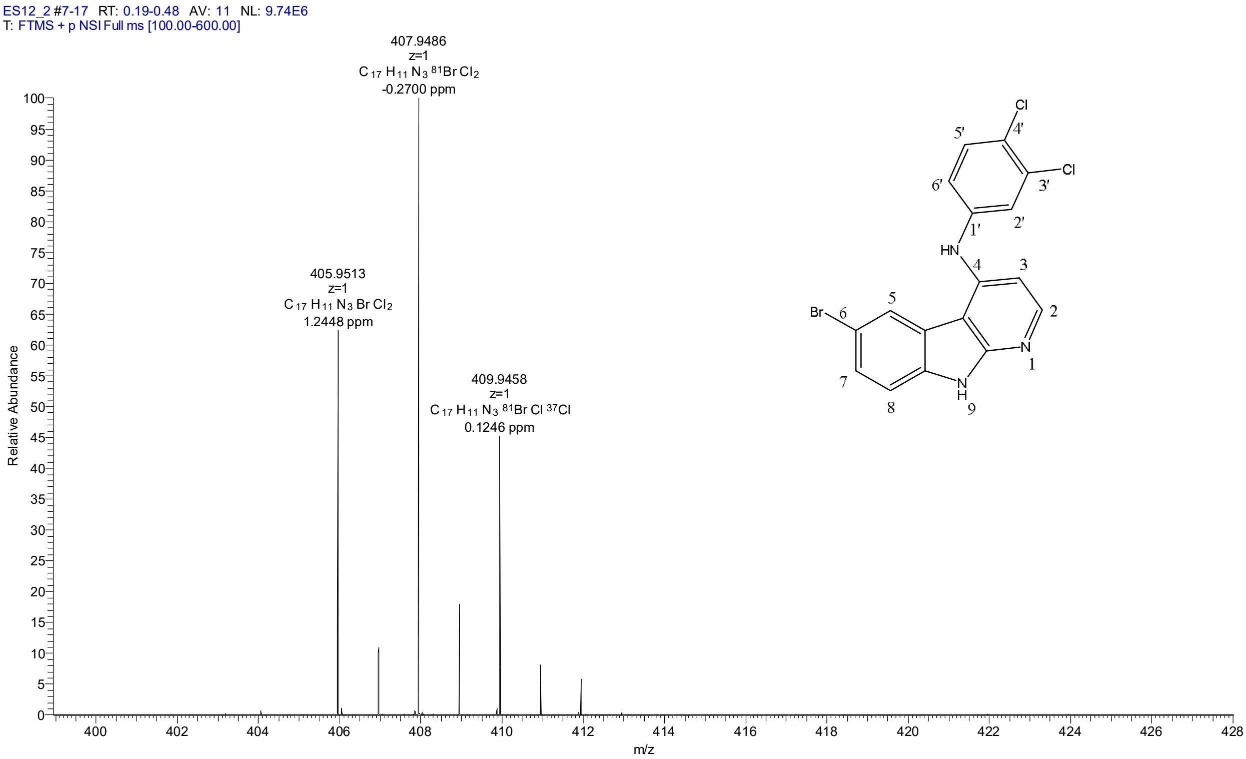

HRMS spectrum of compound **6d**

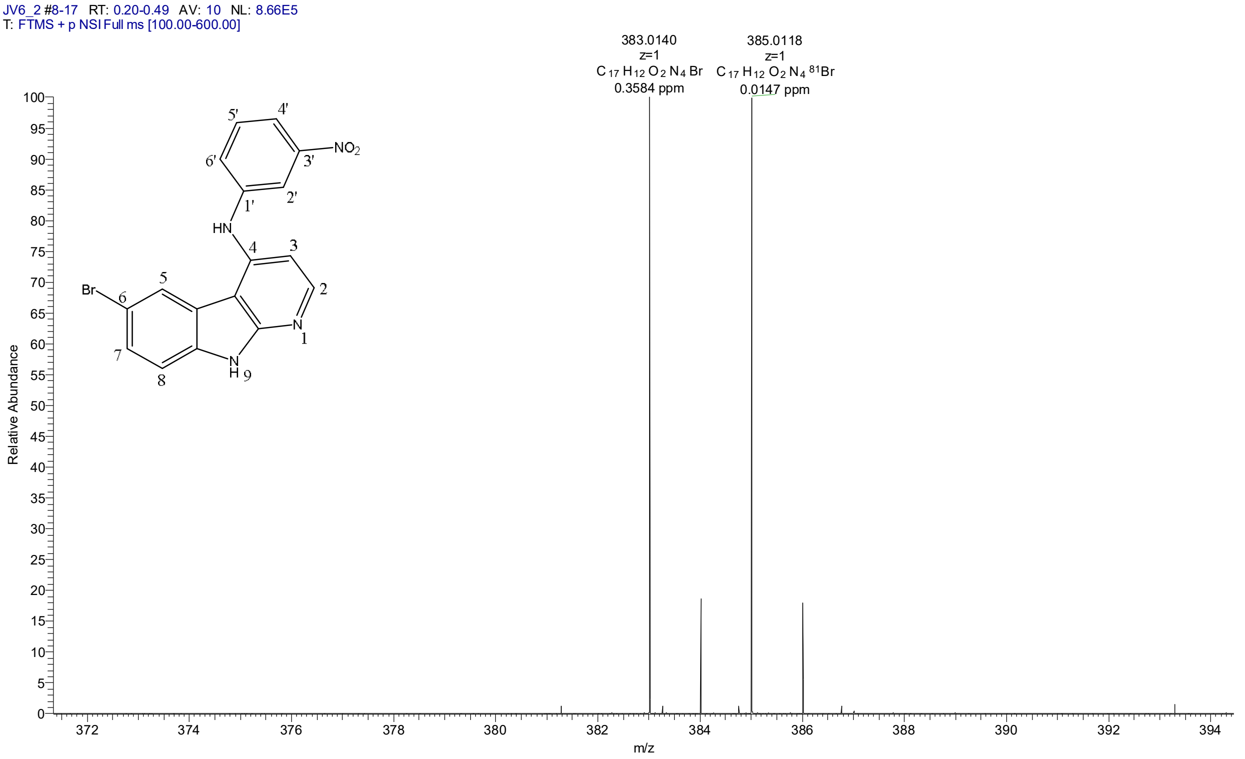

HRMS spectrum of compound **6e**

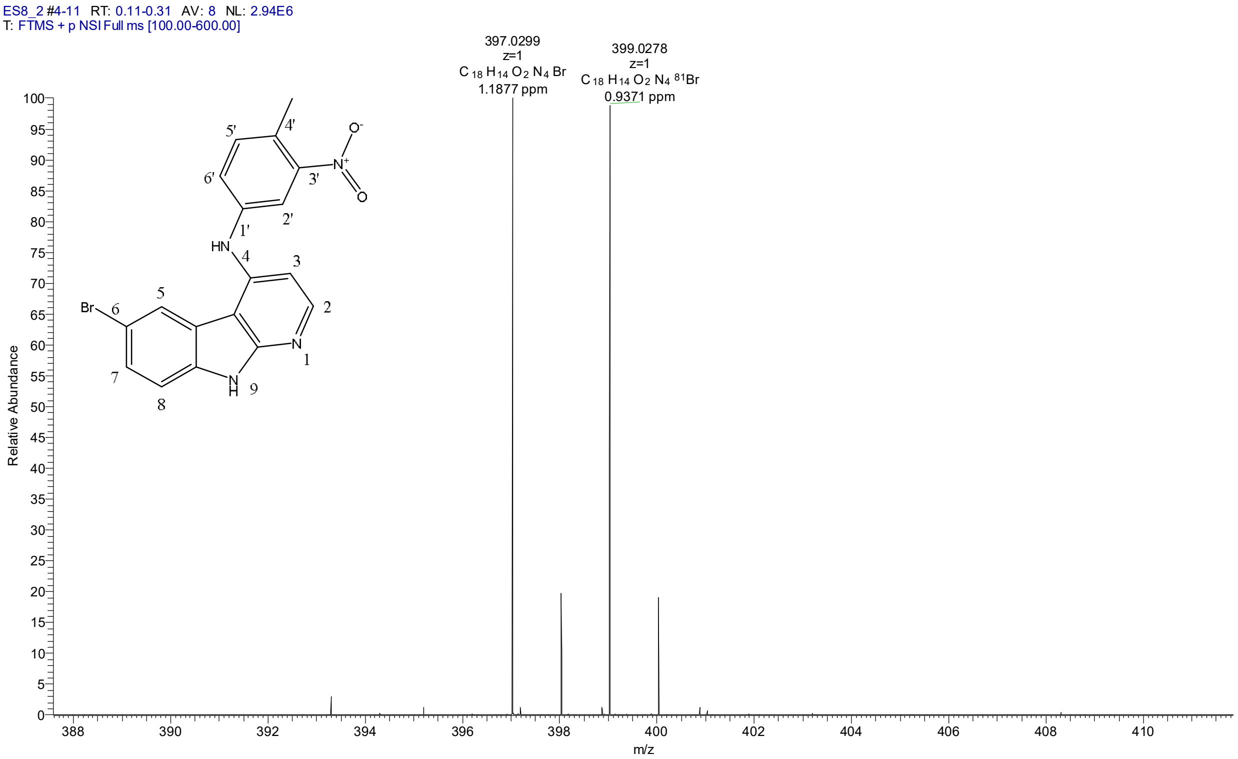

HRMS spectrum of compound **6f**

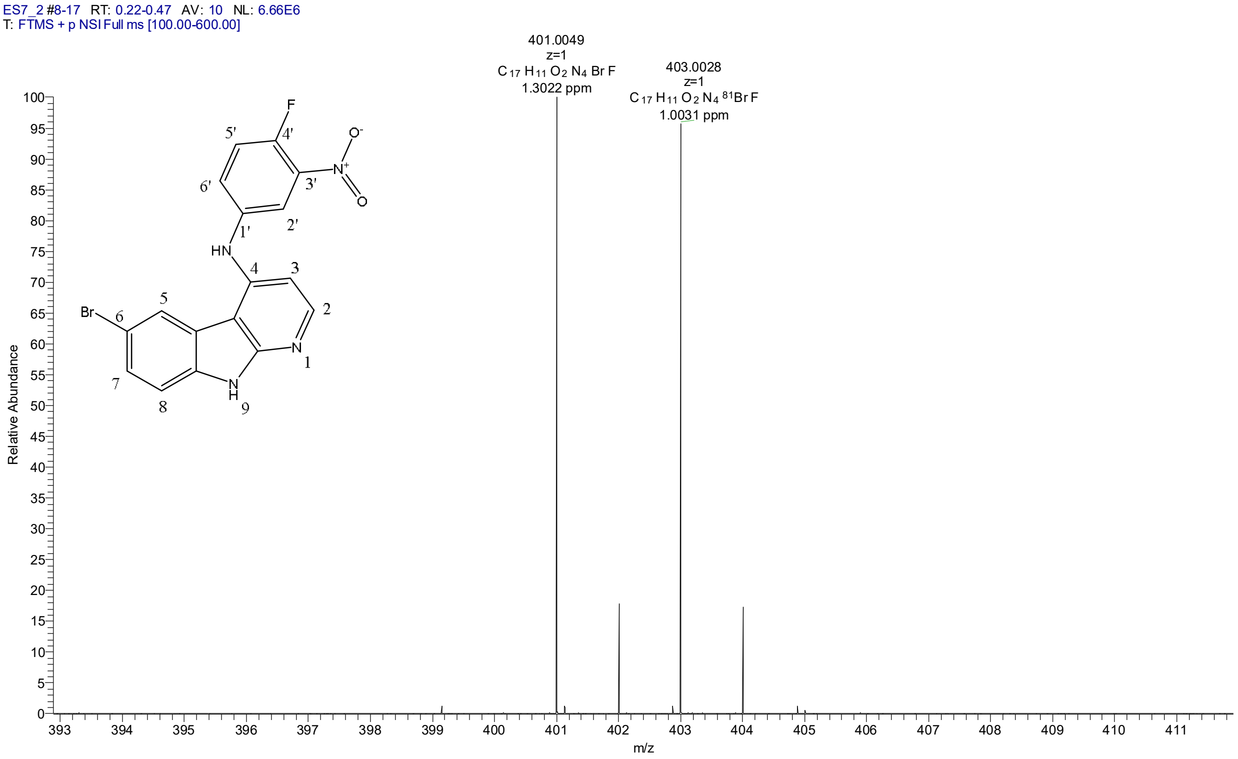

HRMS spectrum of compound **6g**

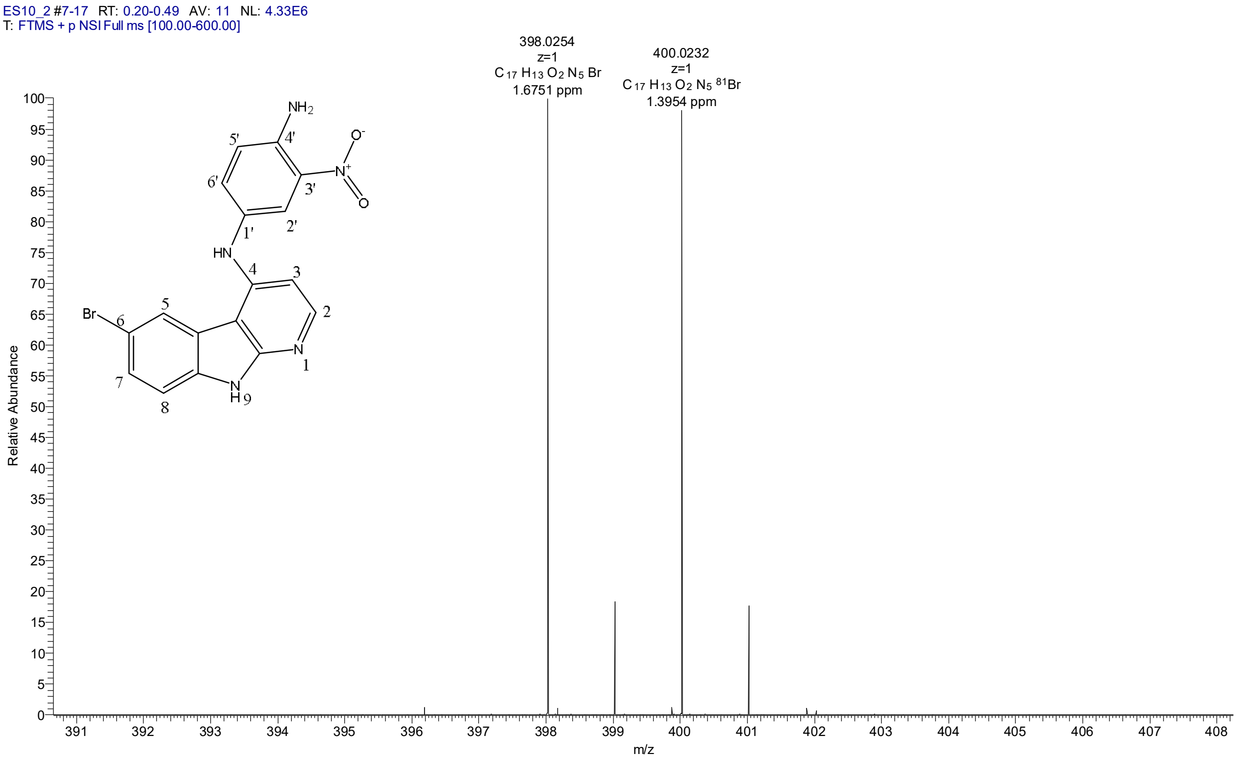

HRMS spectrum of compound **6h**

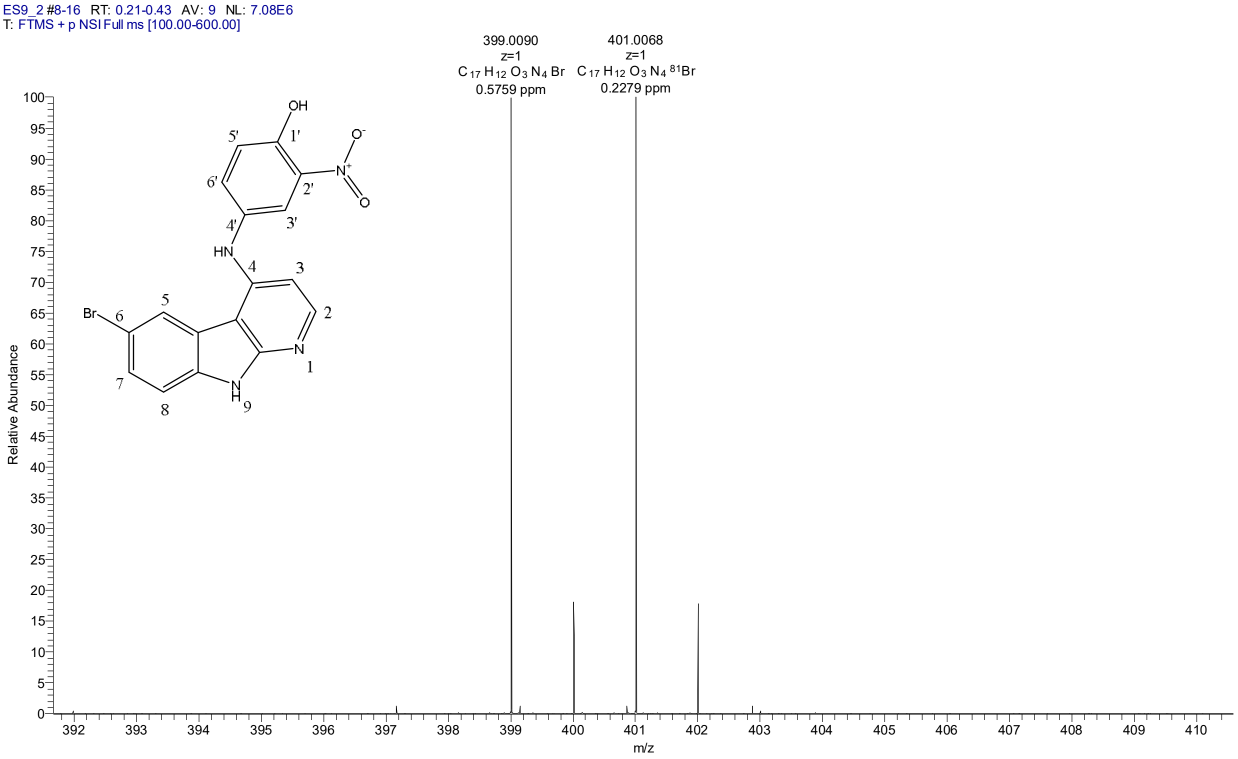

HRMS spectrum of compound **6i**

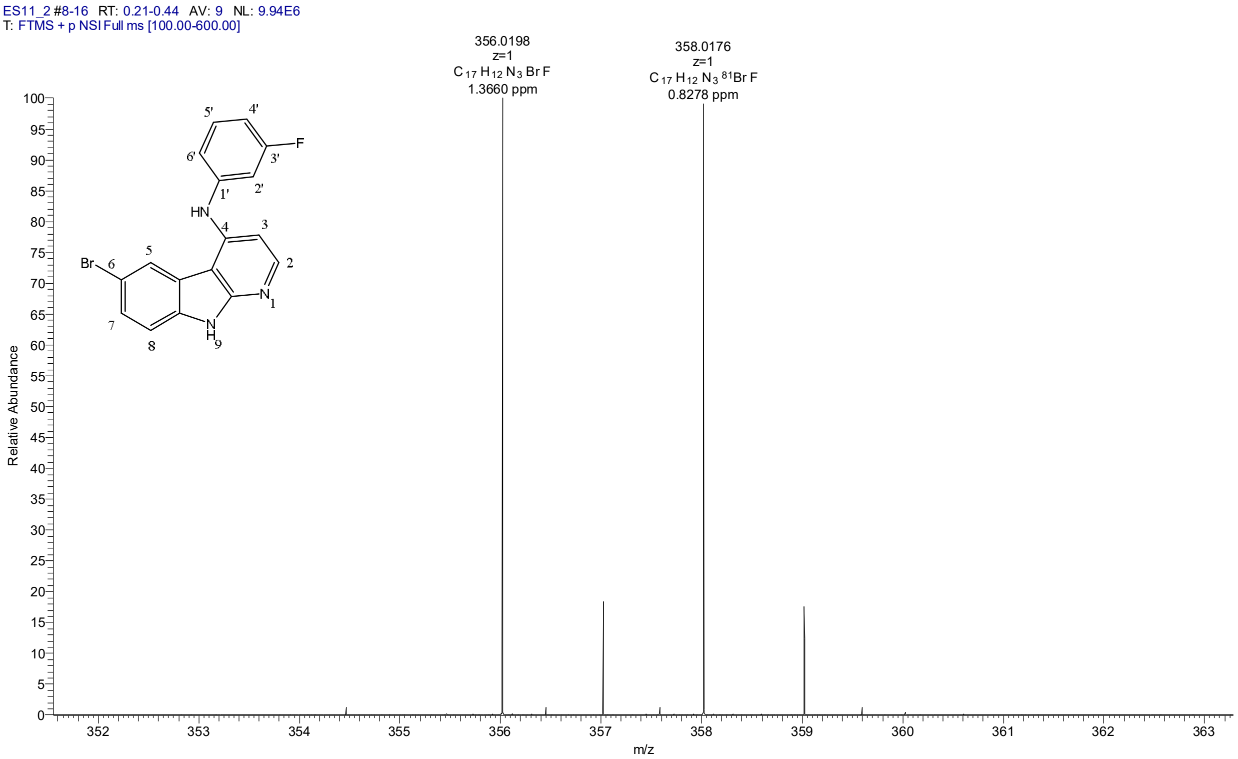

HRMS spectrum of compound **6j**

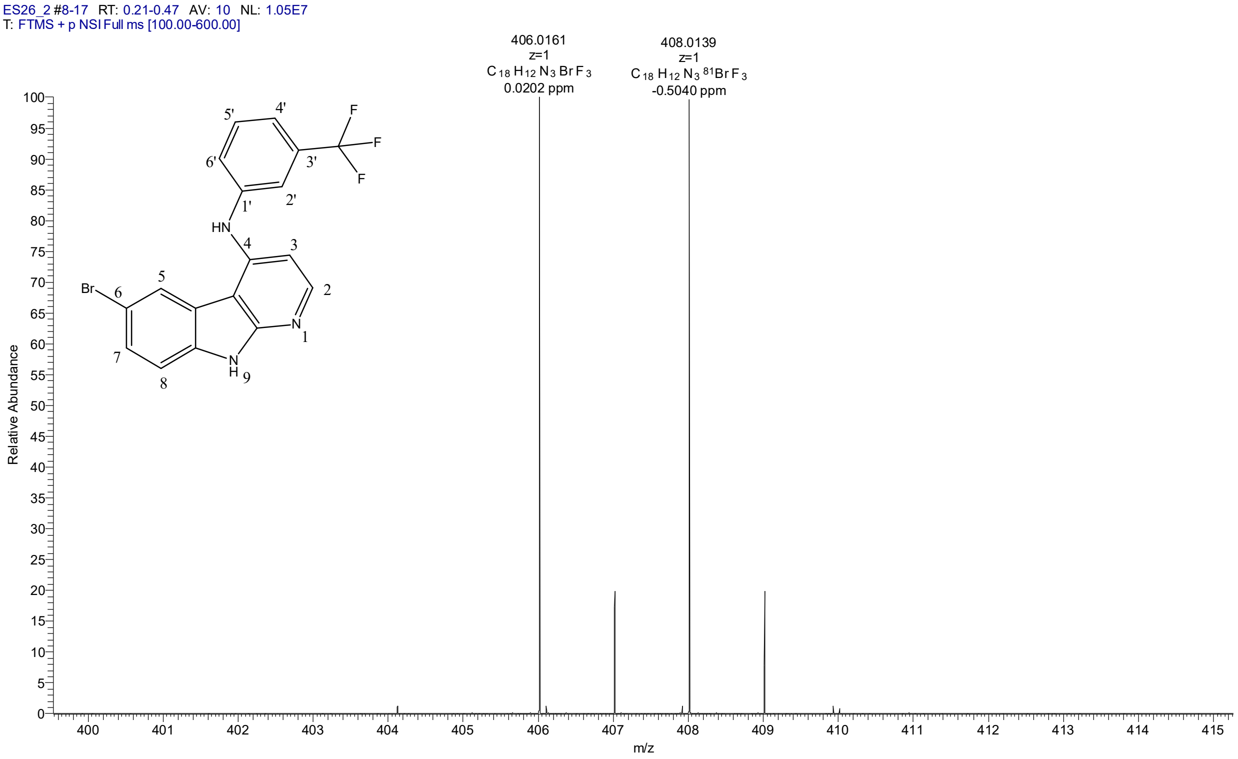

HRMS spectrum of compound **6k**

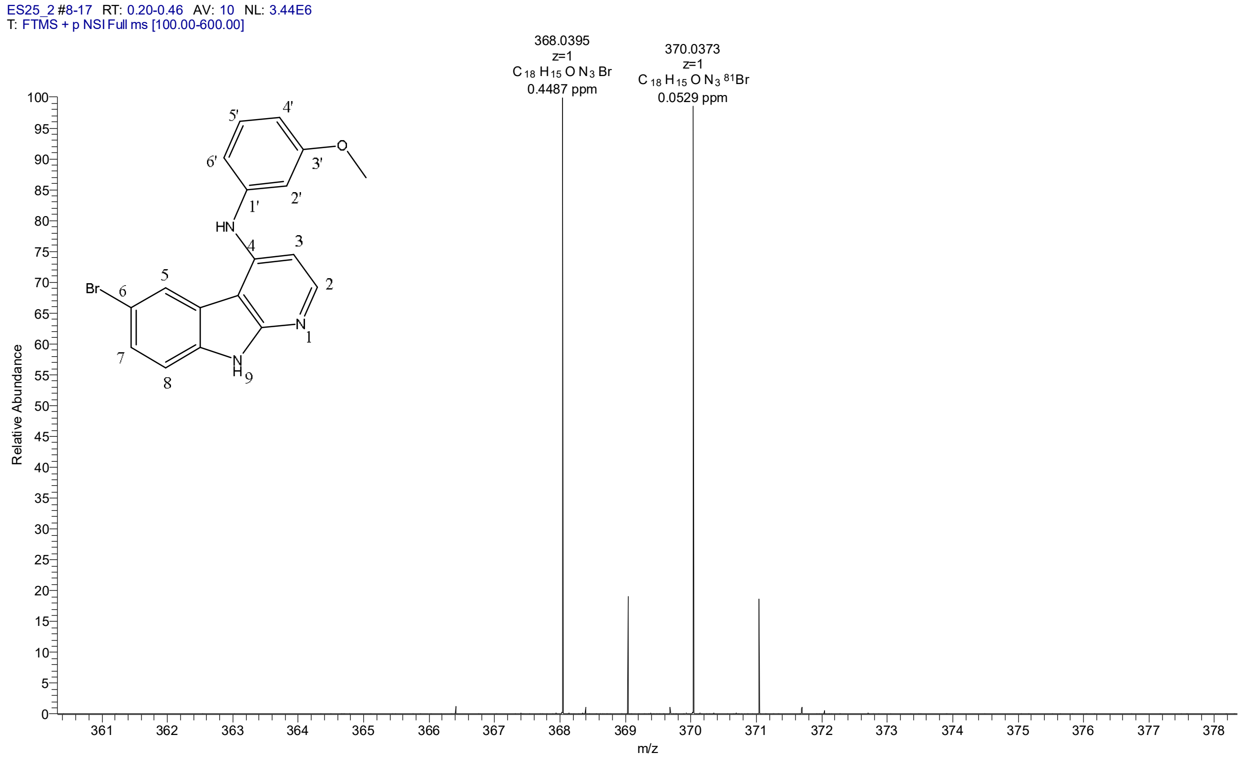

HRMS spectrum of compound **6l**

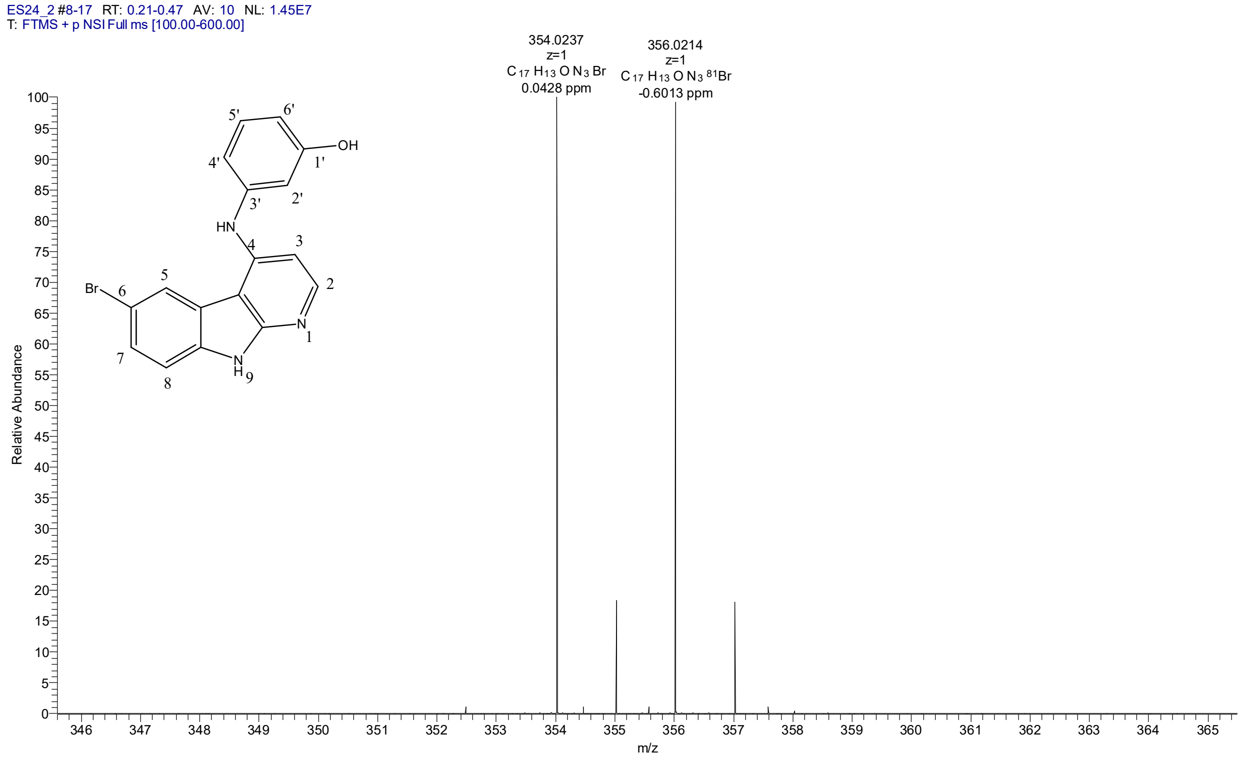

HRMS spectrum of compound **6m**

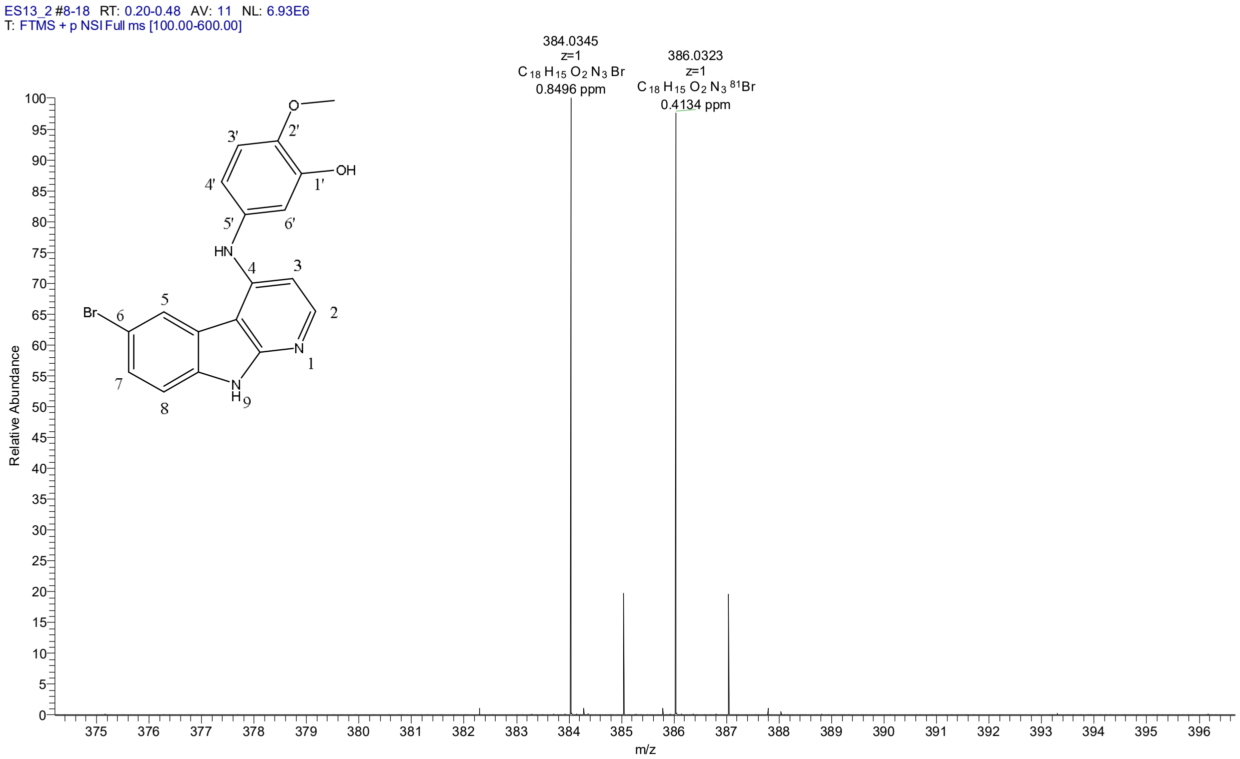

HRMS spectrum of compound **8a**

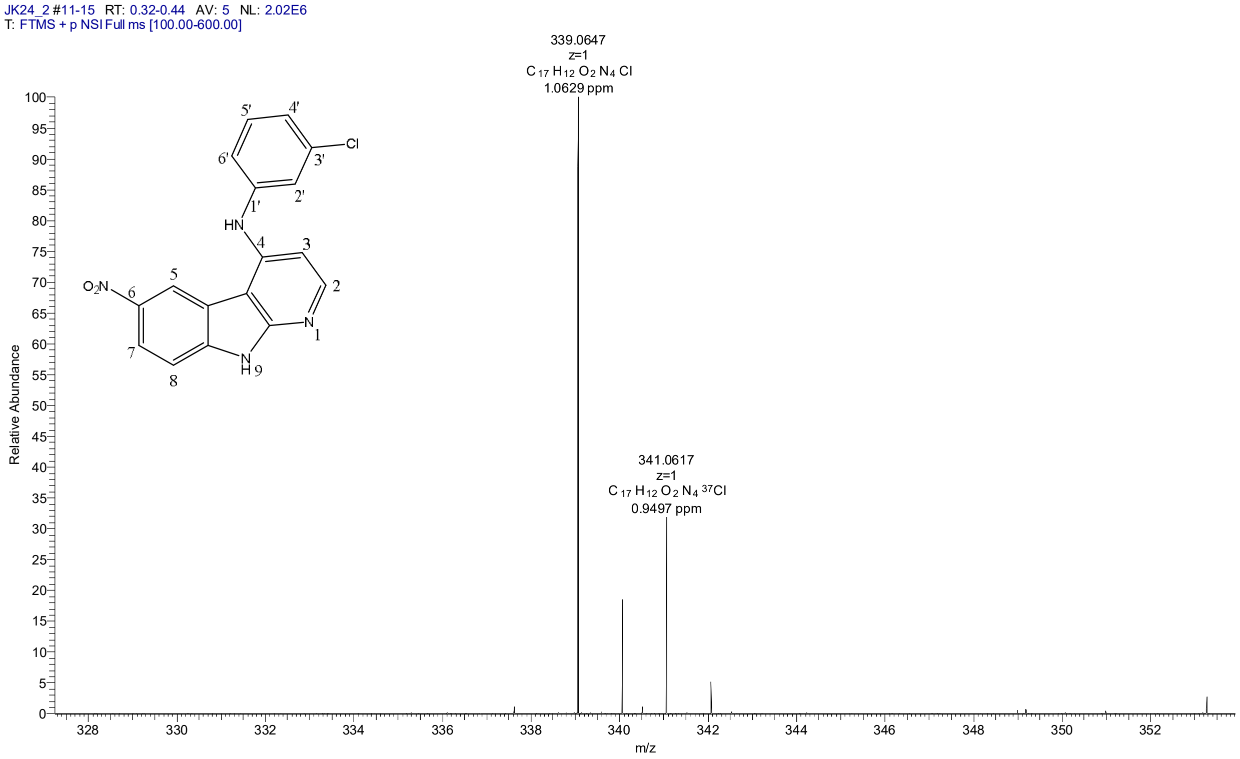

HRMS spectrum of compound **8b**

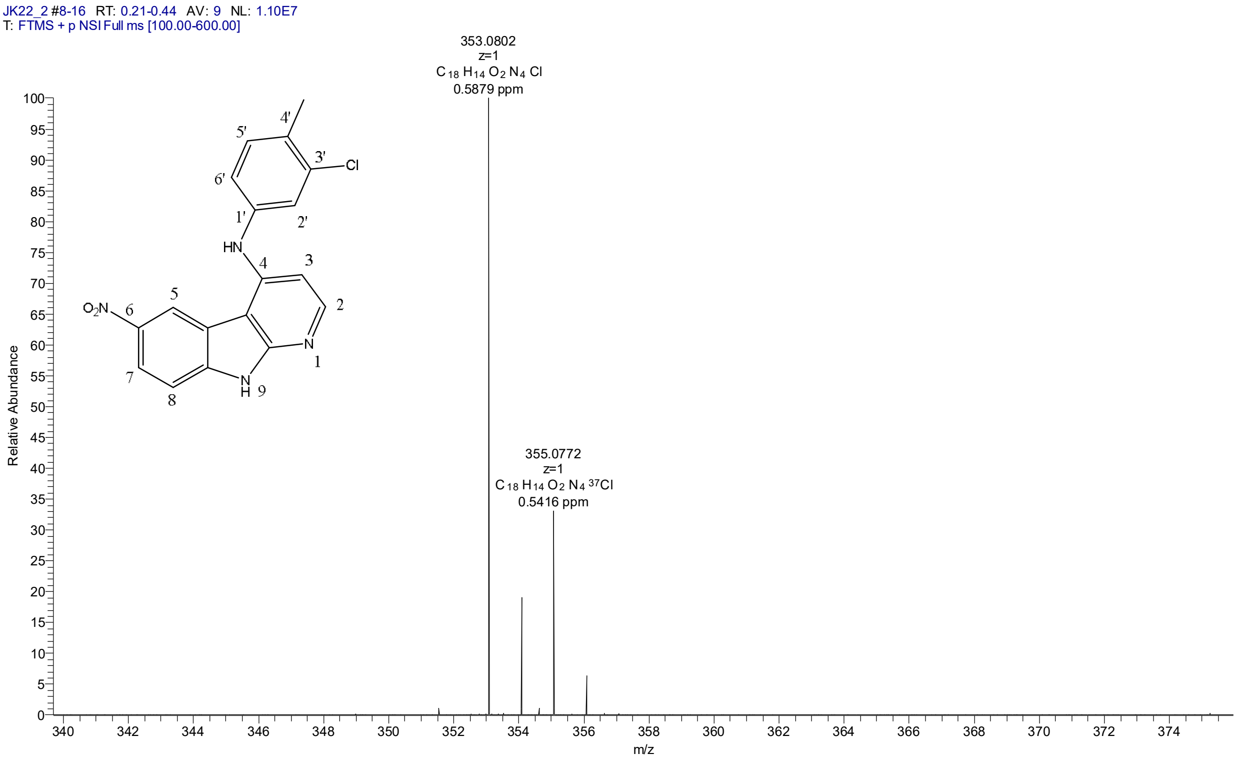

HRMS spectrum of compound **8c**

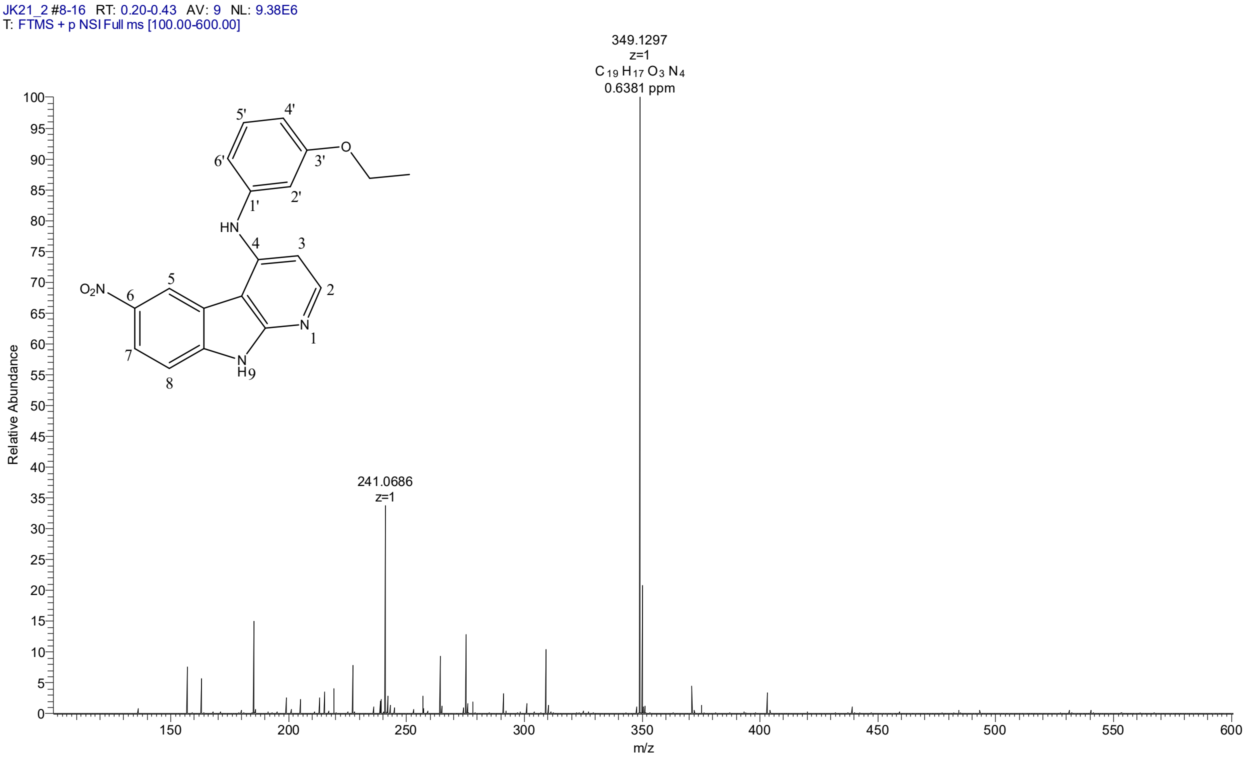

HRMS spectrum of compound **8d**

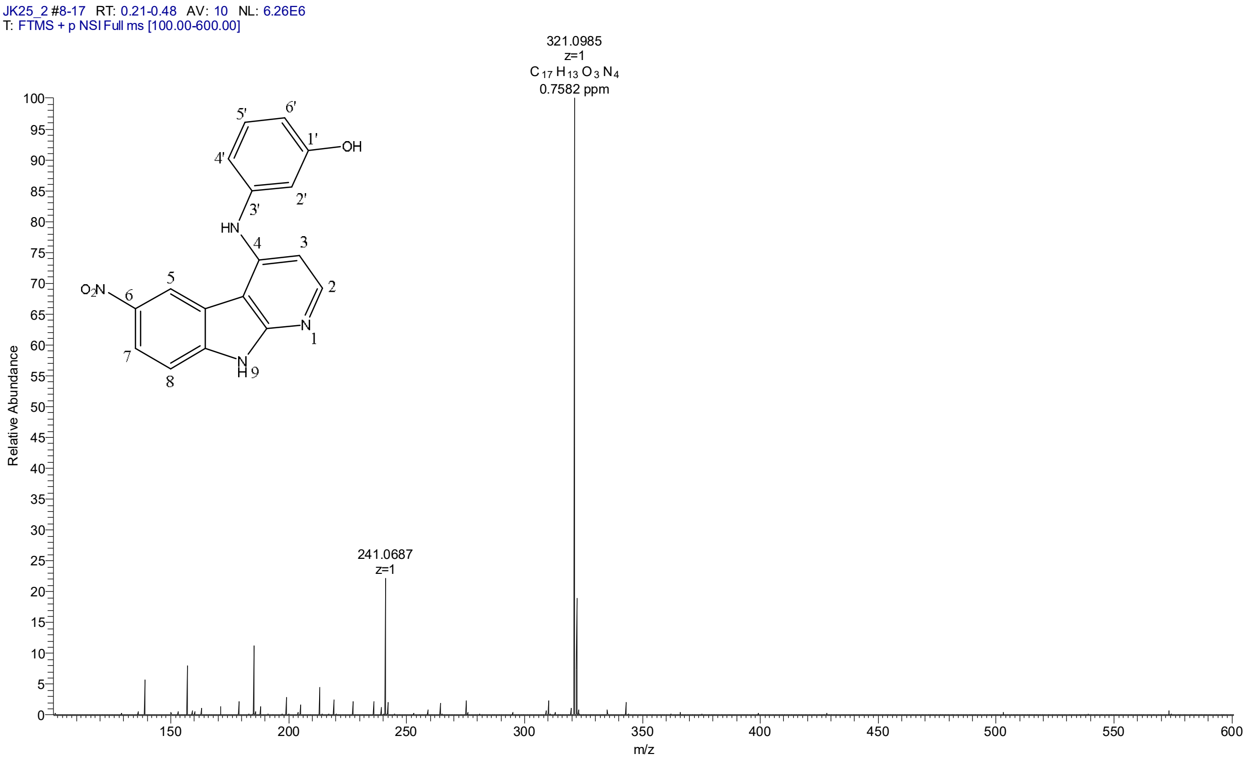

HRMS spectrum of compound **8e**

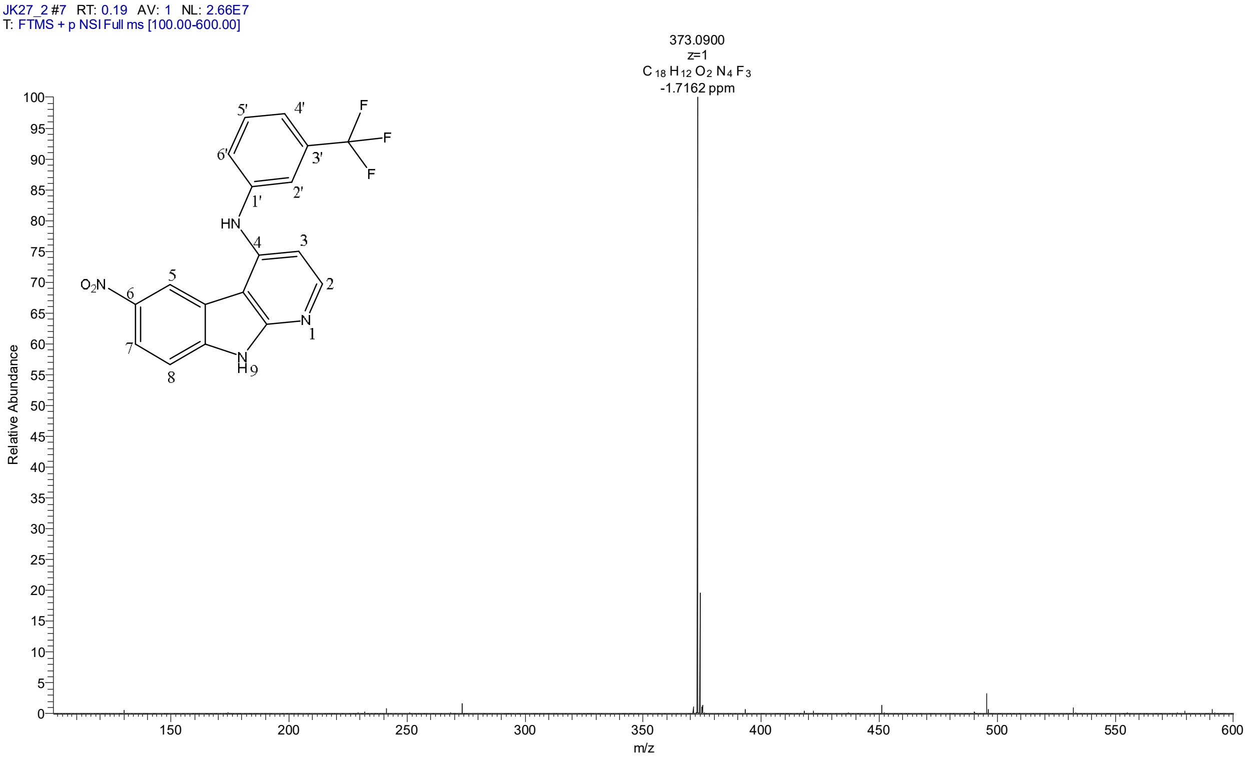

HRMS spectrum of compound **9a**

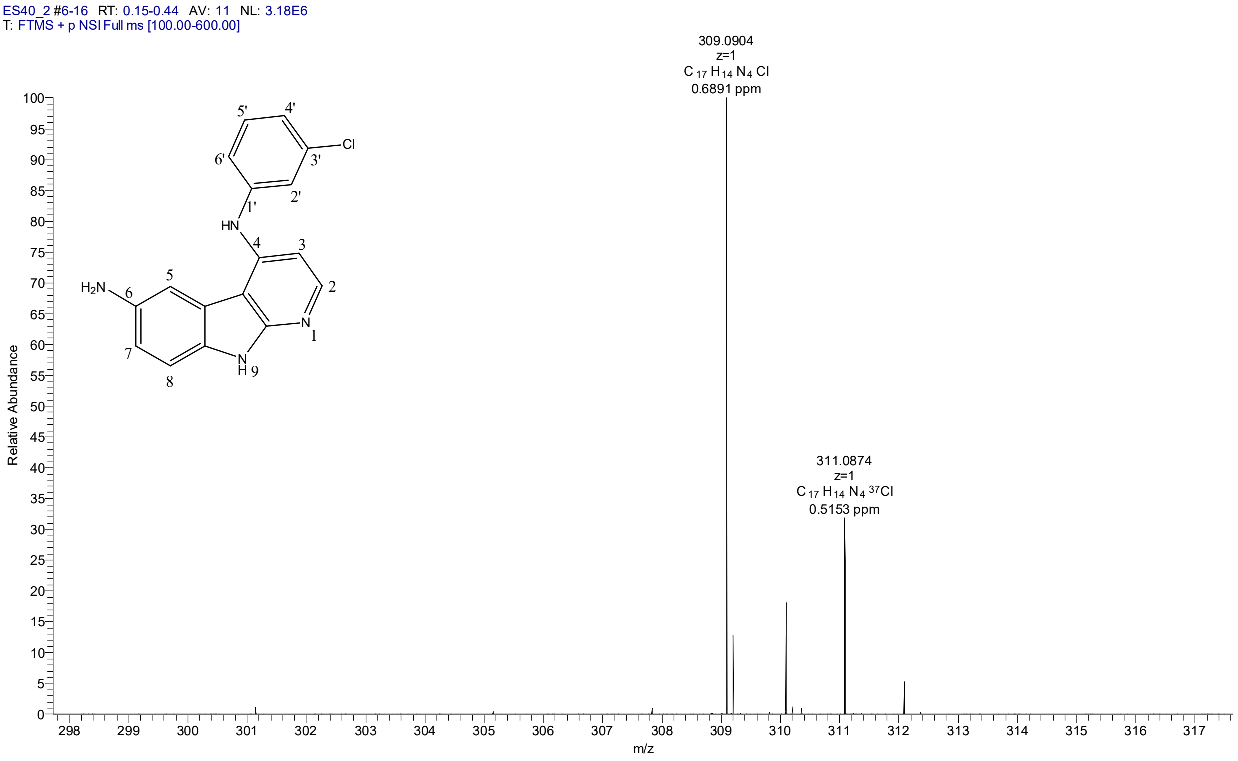

HRMS spectrum of compound **9b**

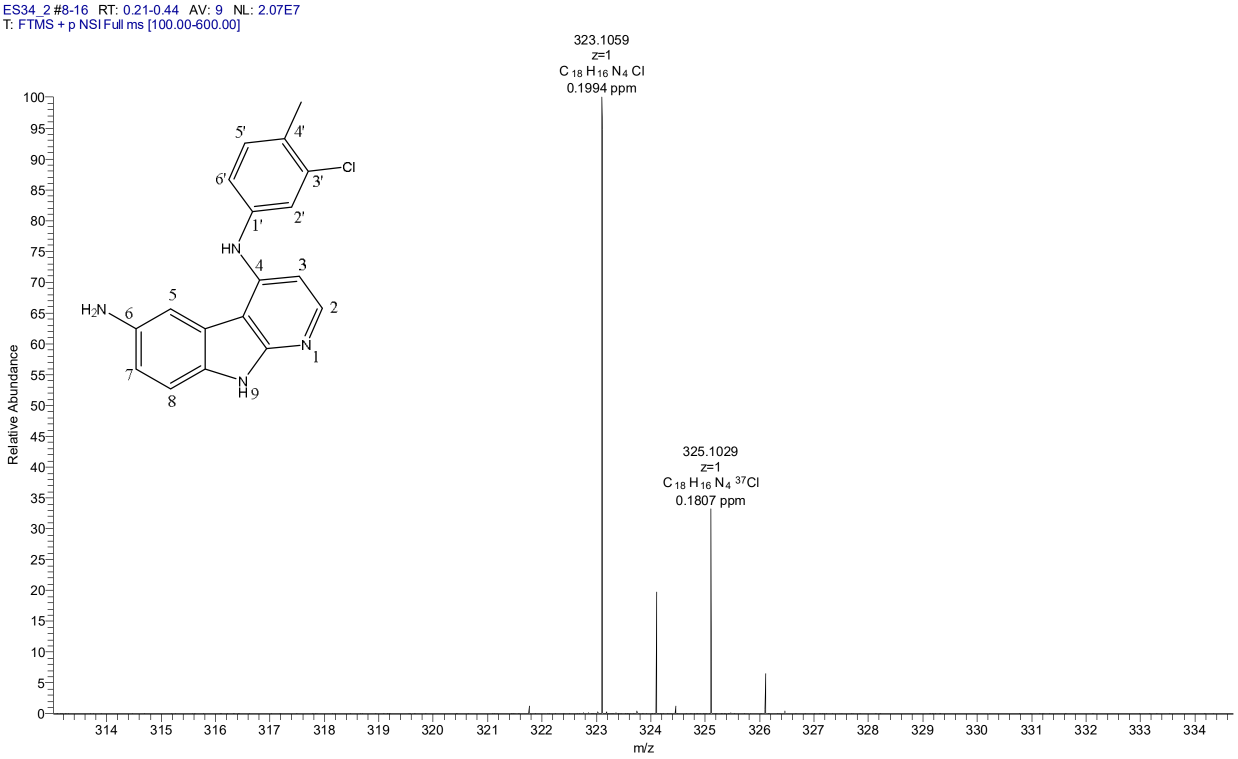

HRMS spectrum of compound **9c**

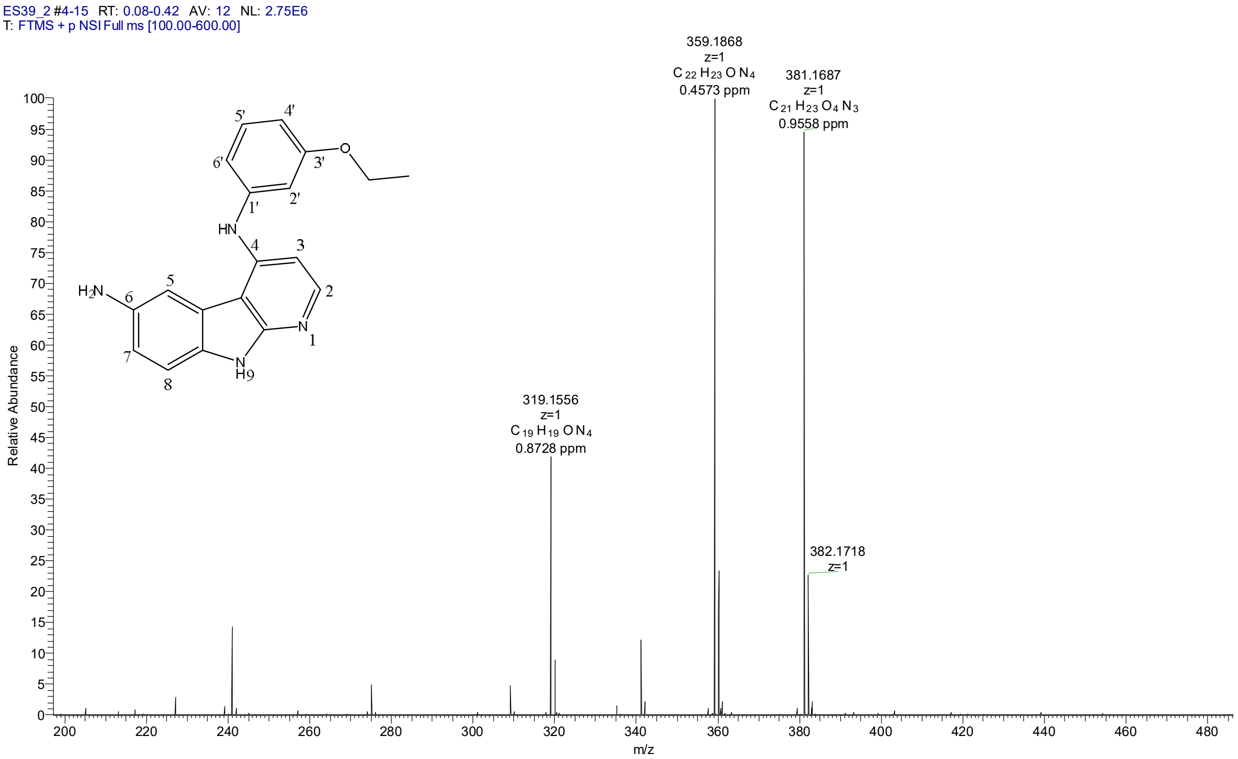

HRMS spectrum of compound **9d**

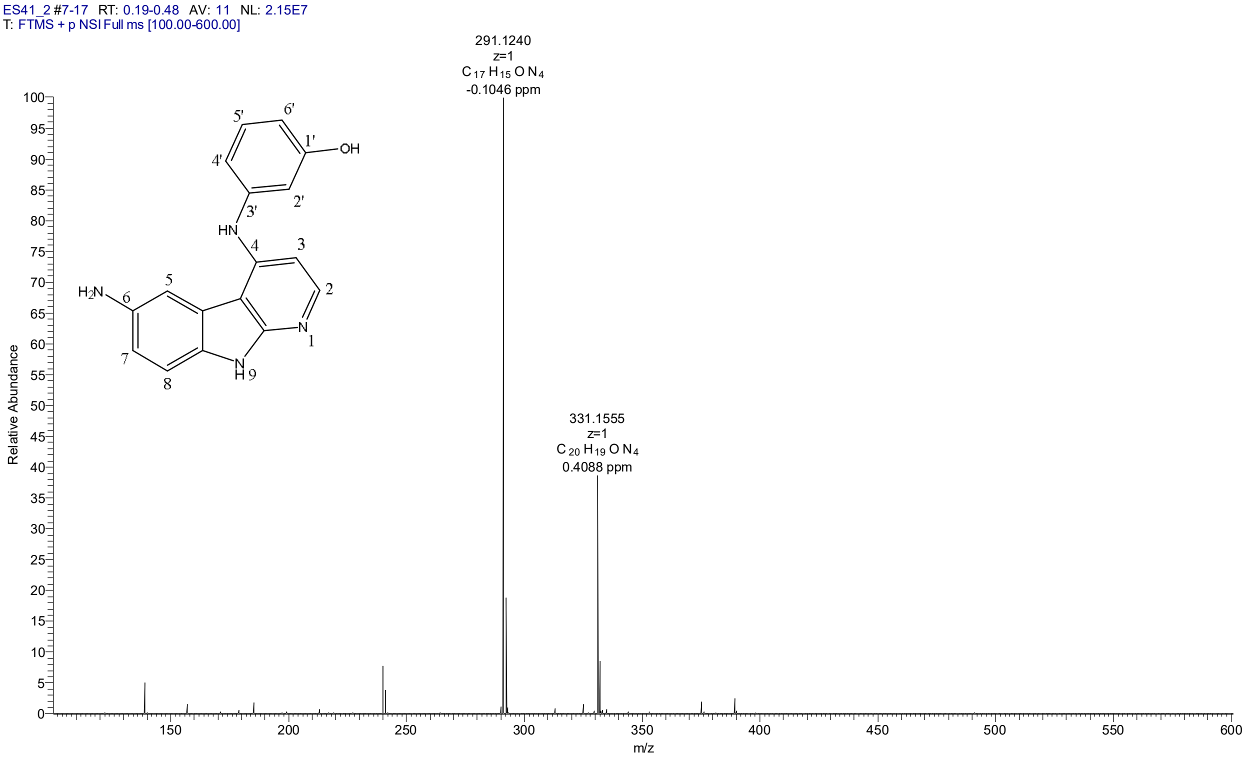

^{13}C NMR spectrum of compound **2a**

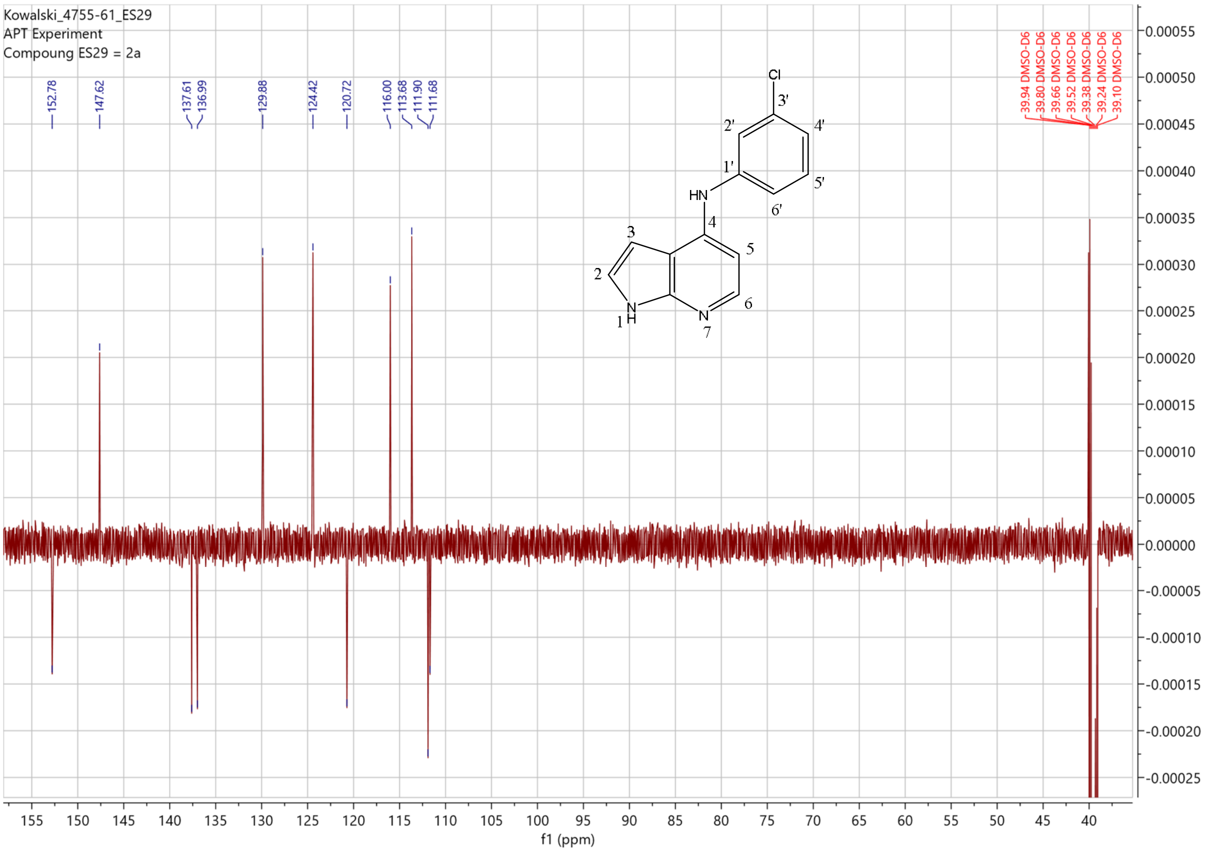

^{13}C NMR spectrum of compound **2b**

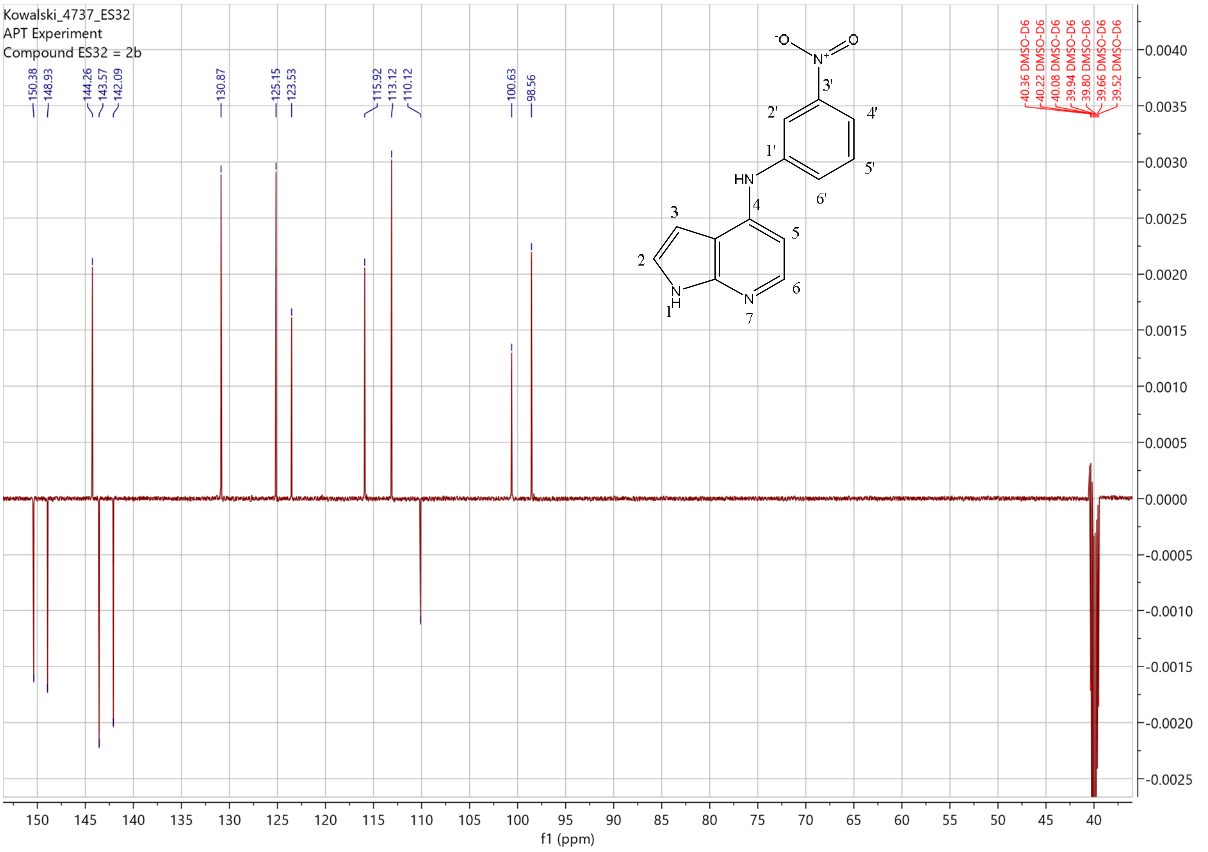

^{13}C NMR spectrum of compound **2c**

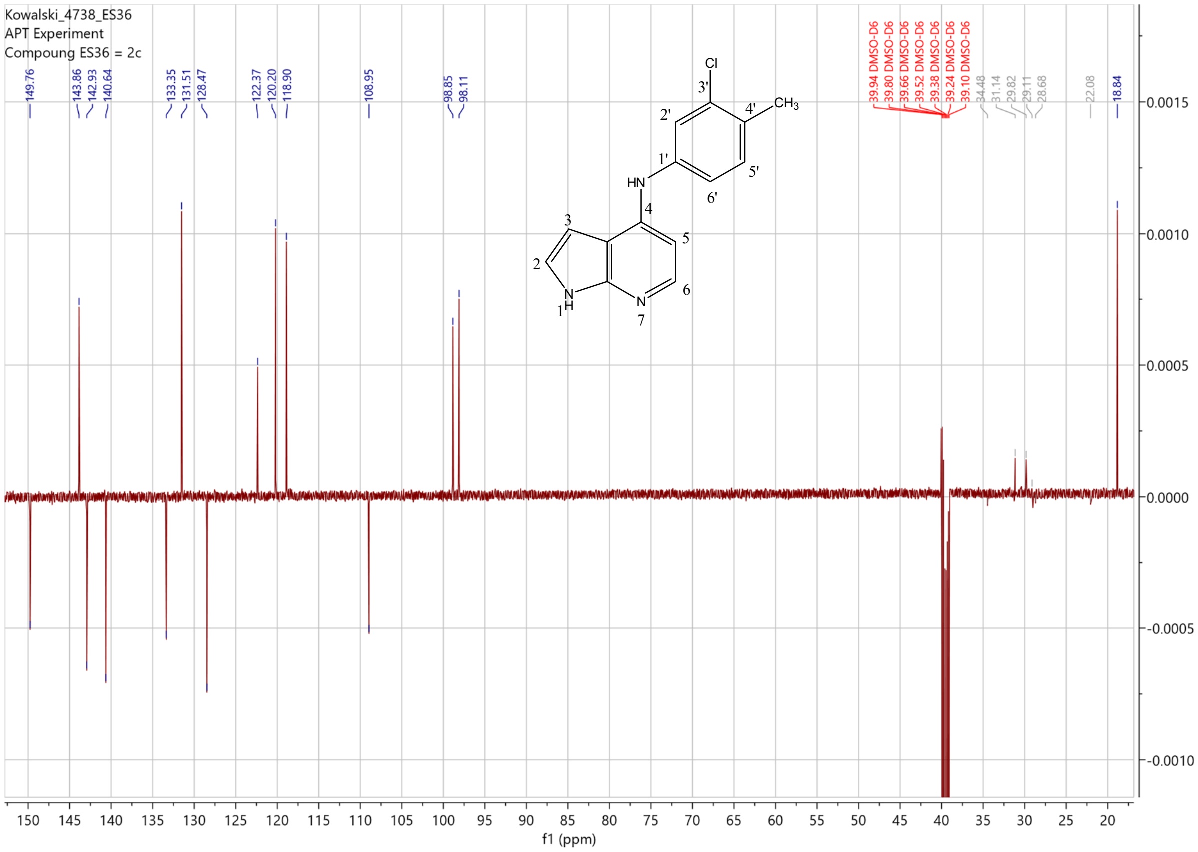

^{13}C NMR spectrum of compound **2d**

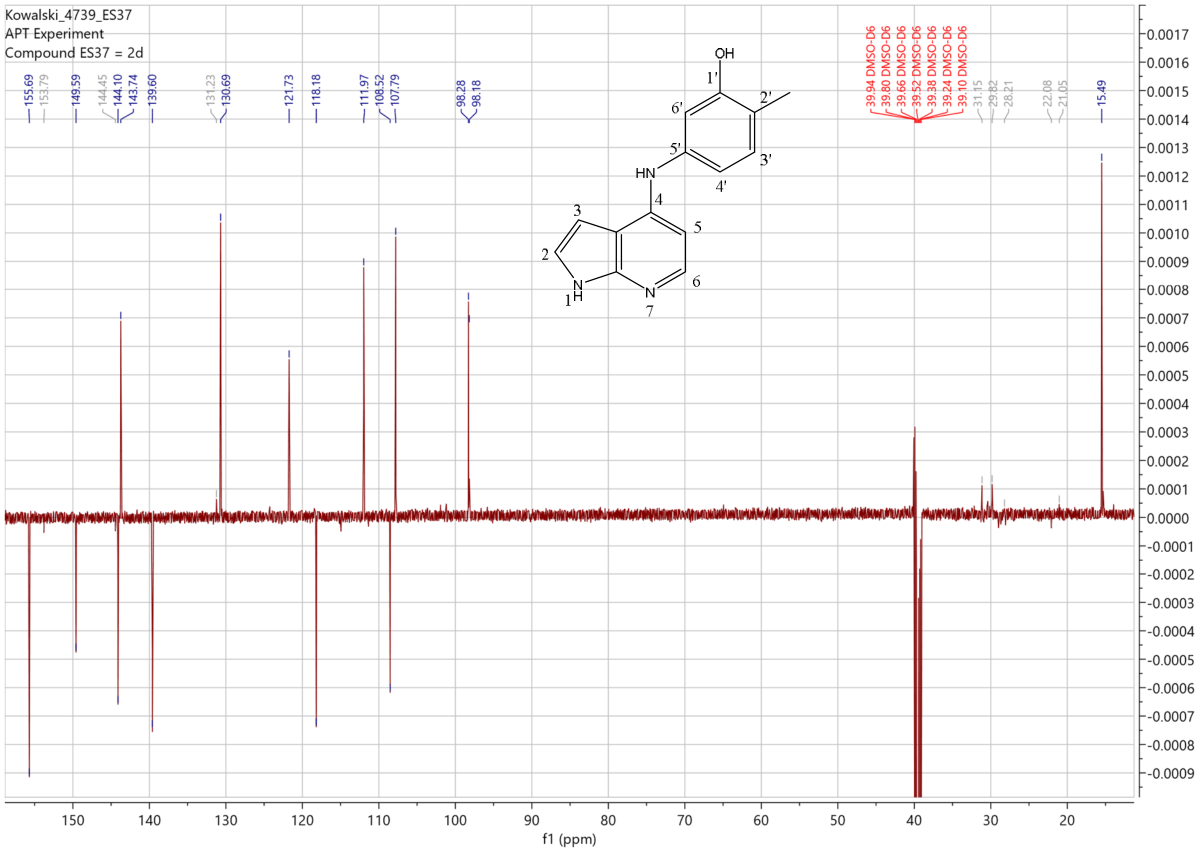

^{13}C NMR spectrum of compound **6a**

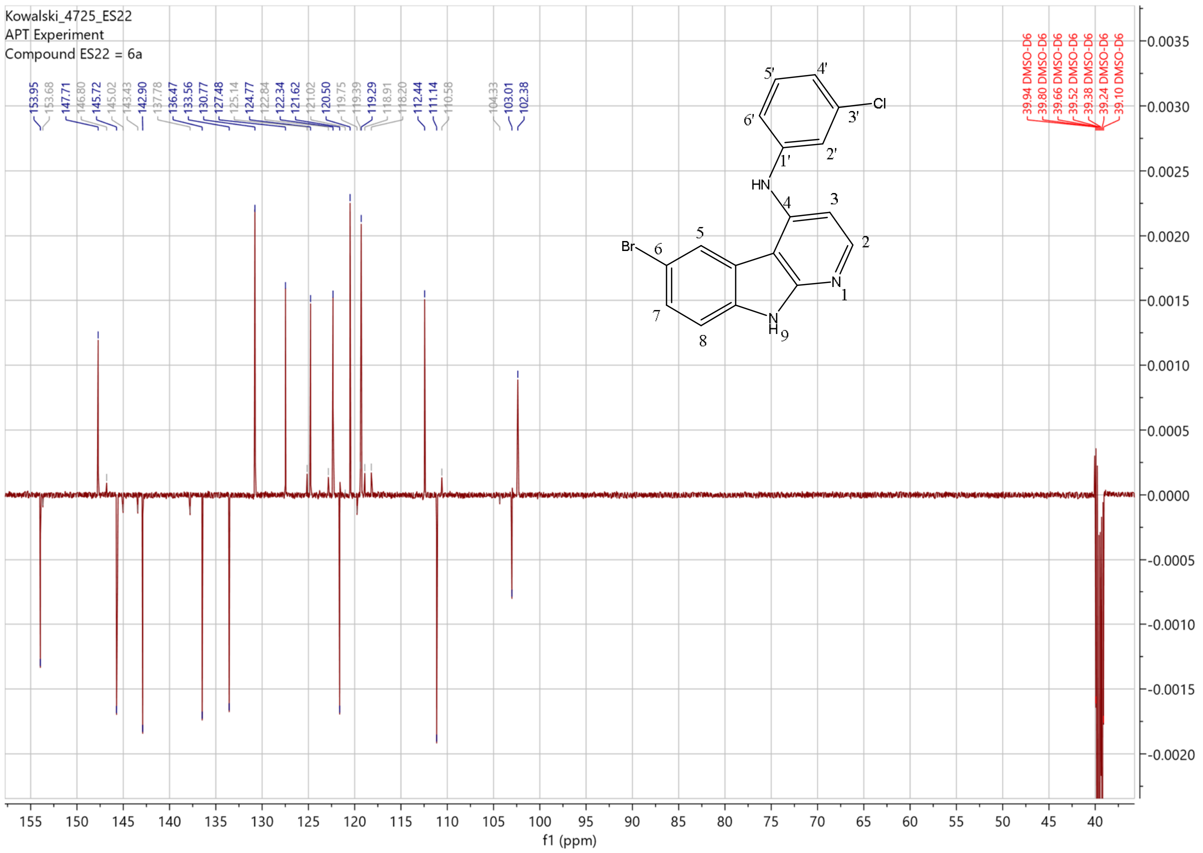

^{13}C NMR spectrum of compound **6b**

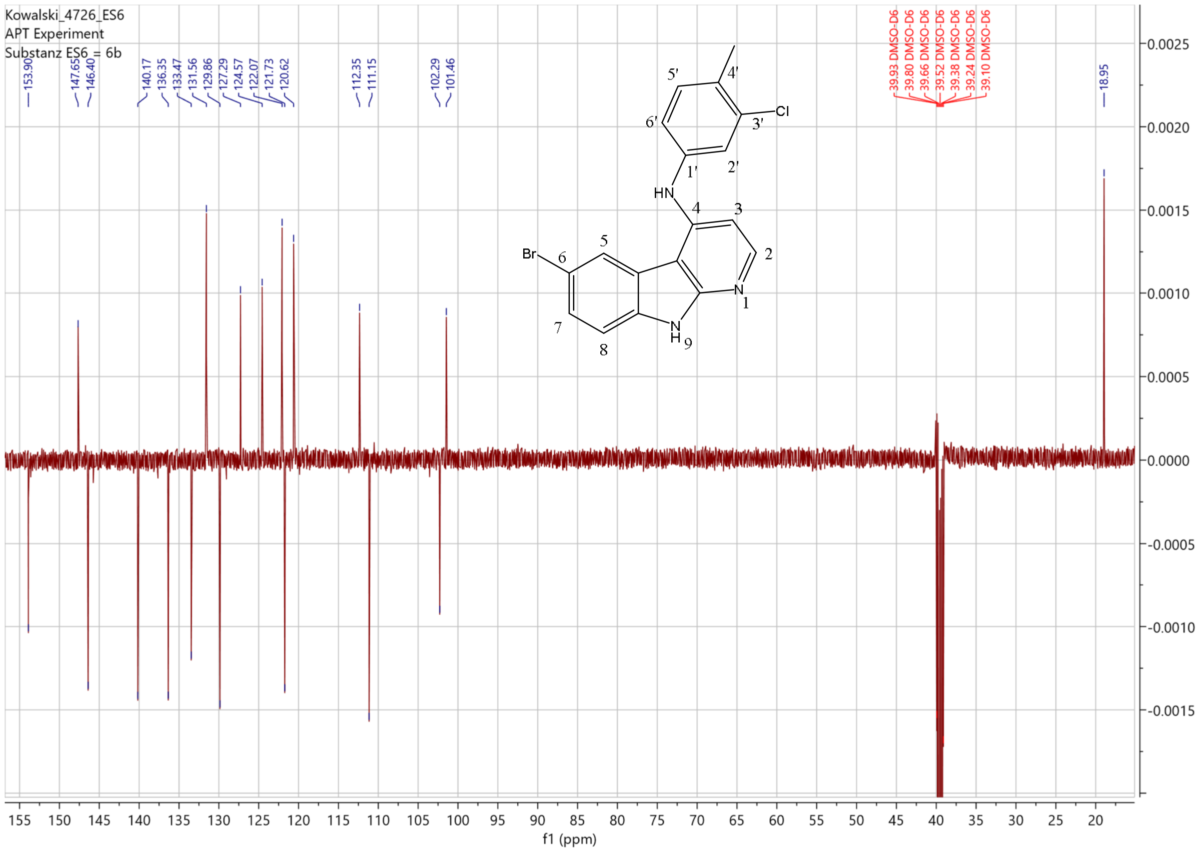

^{13}C NMR spectrum of compound **6c**

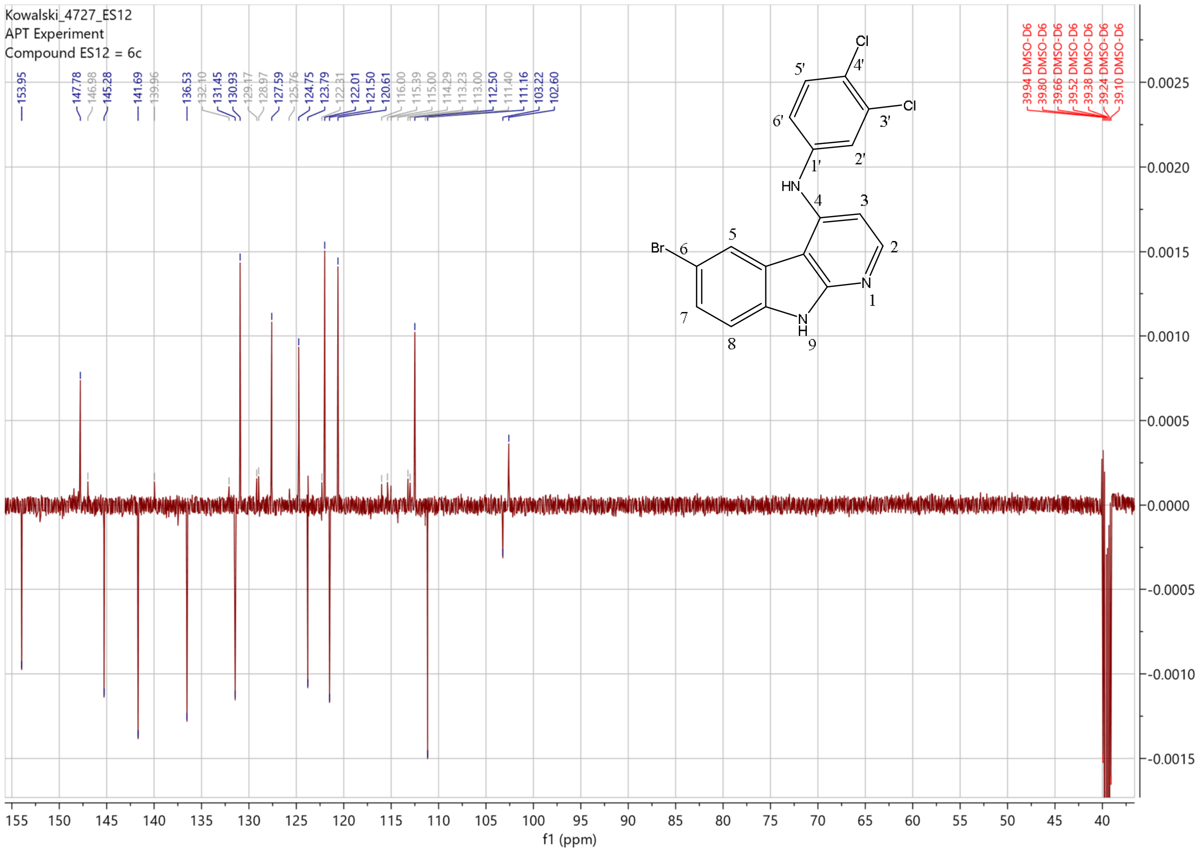

^{13}C NMR spectrum of compound **6d**

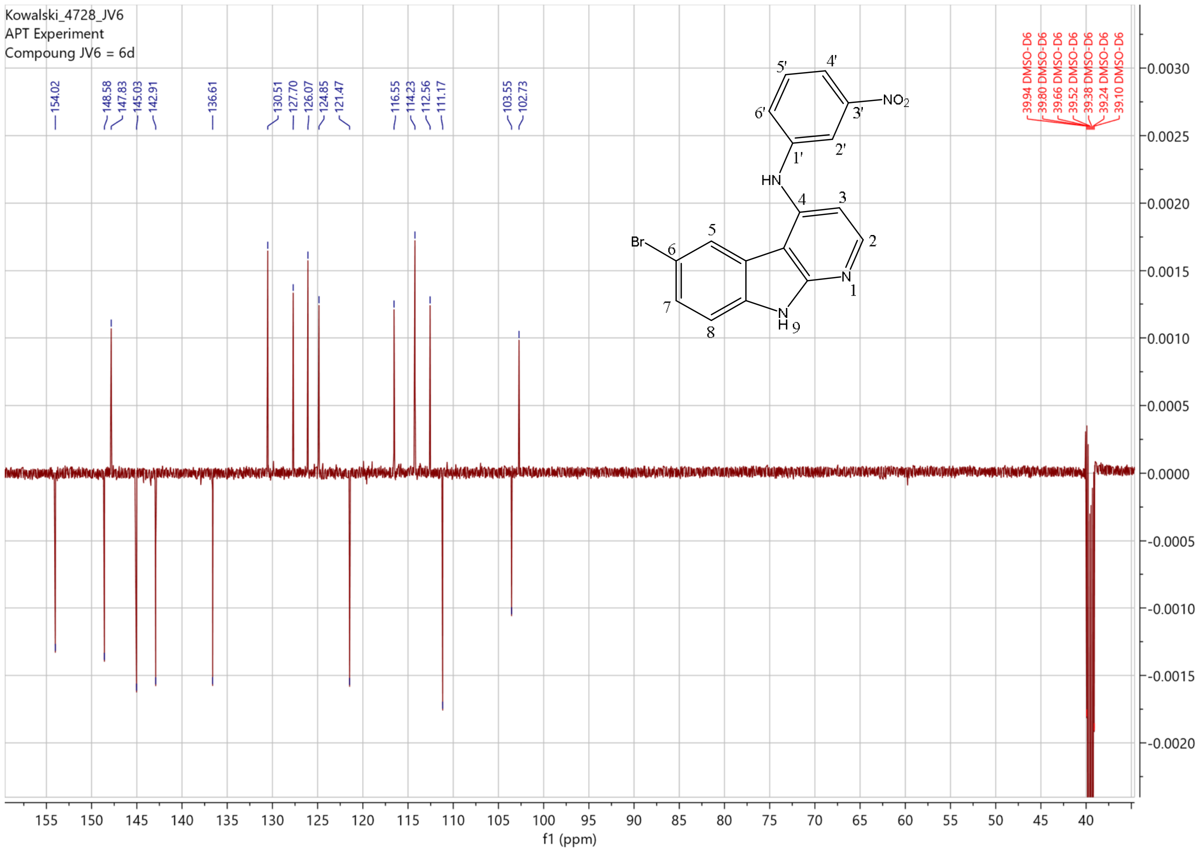

^{13}C NMR spectrum of compound **6e**

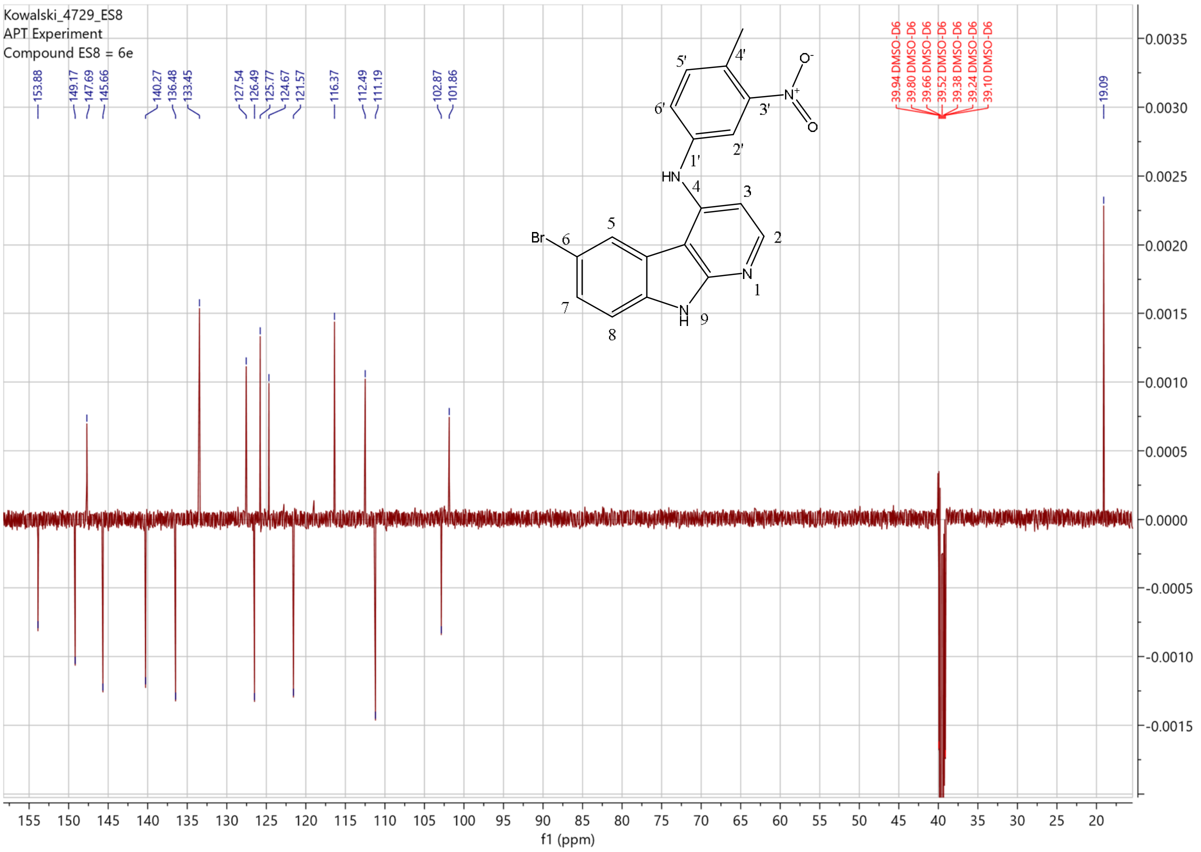

^{13}C NMR spectrum of compound **6f**

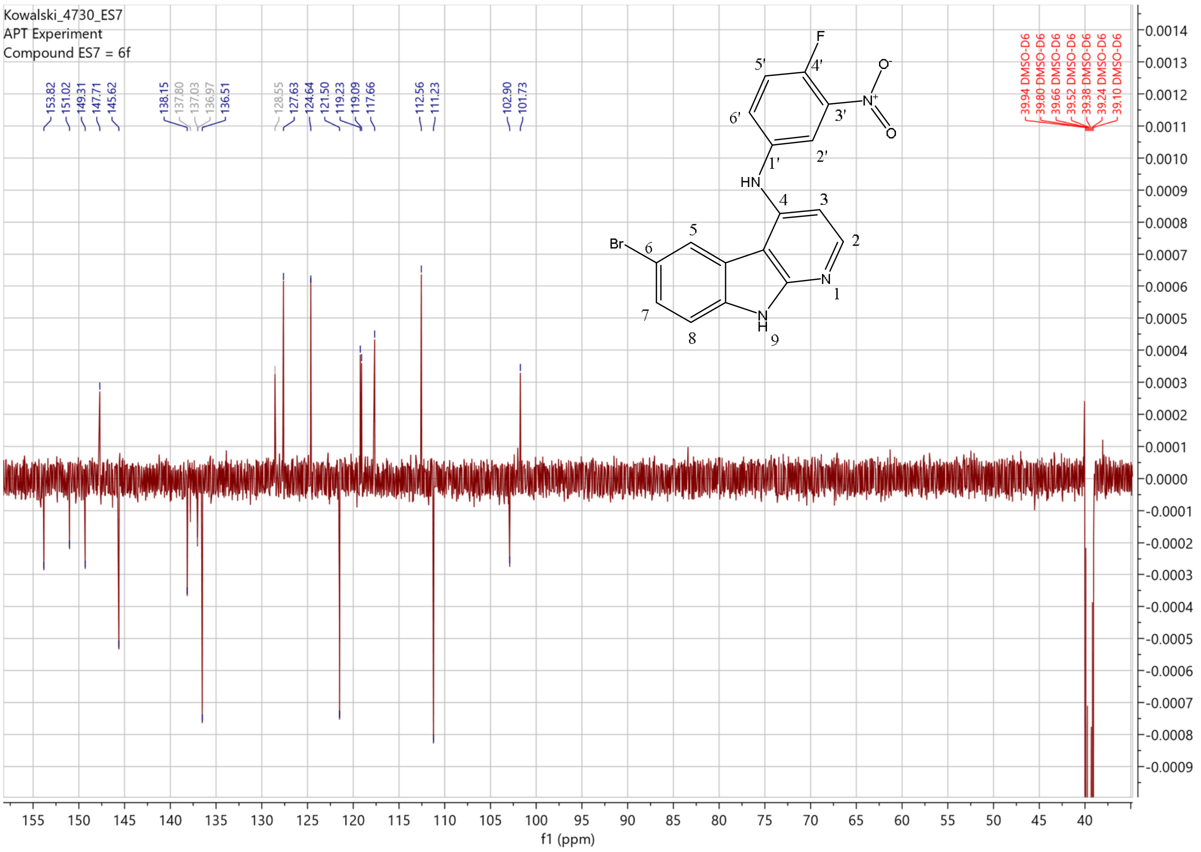

^{13}C NMR spectrum of compound **6g**

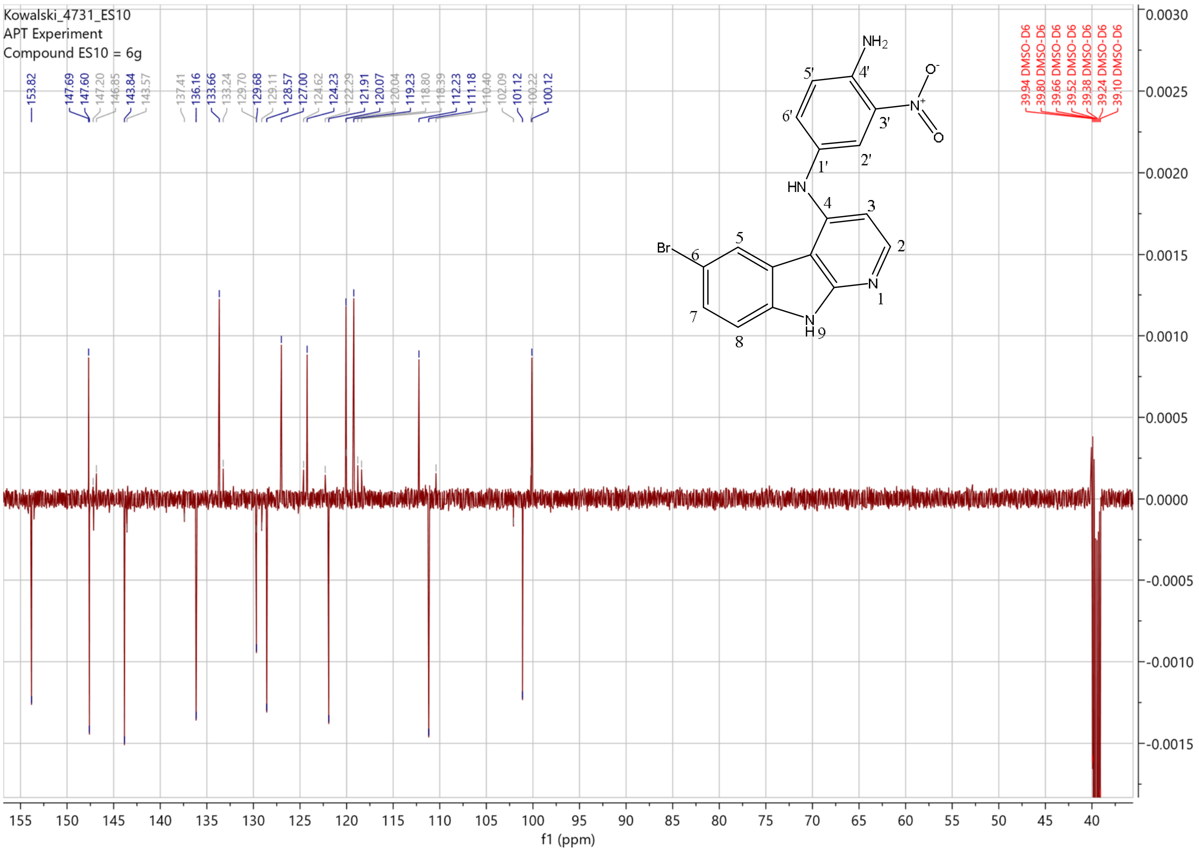

^{13}C NMR spectrum of compound **6h**

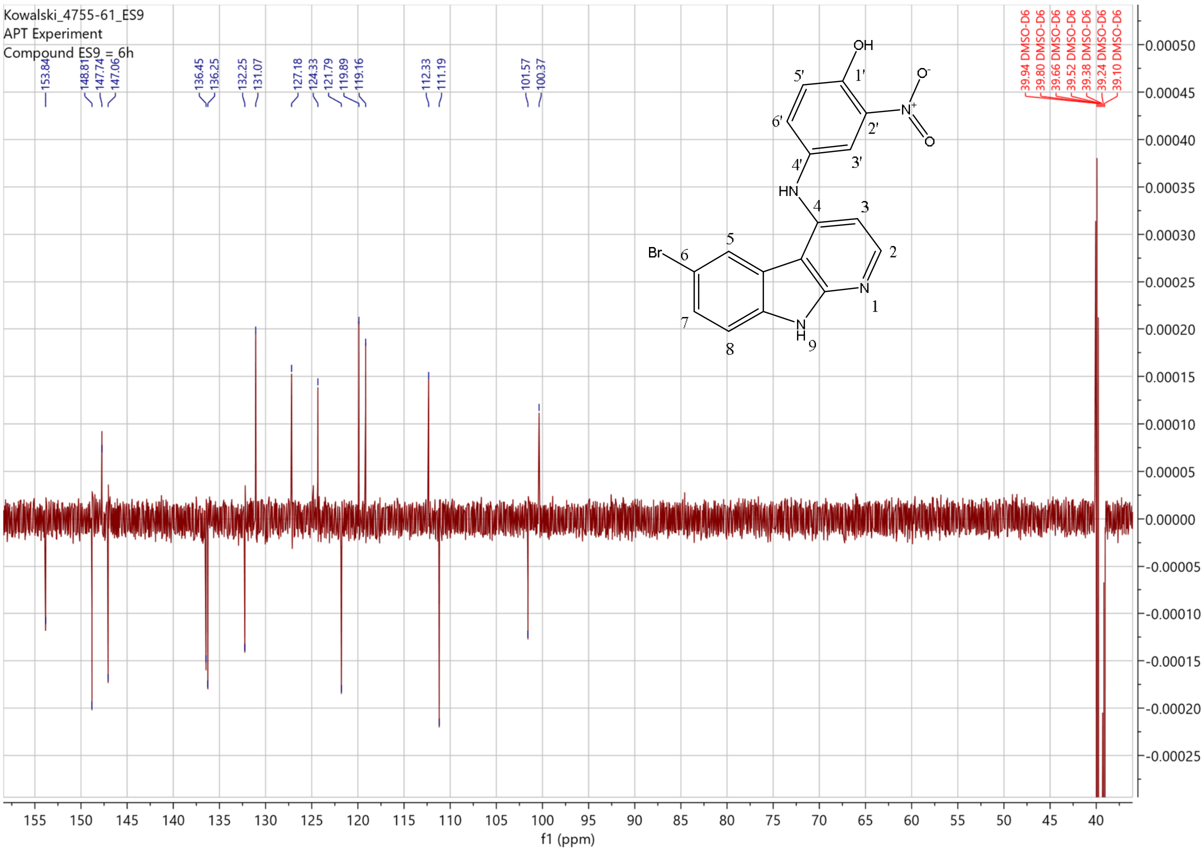

^{13}C NMR spectrum of compound **6i**

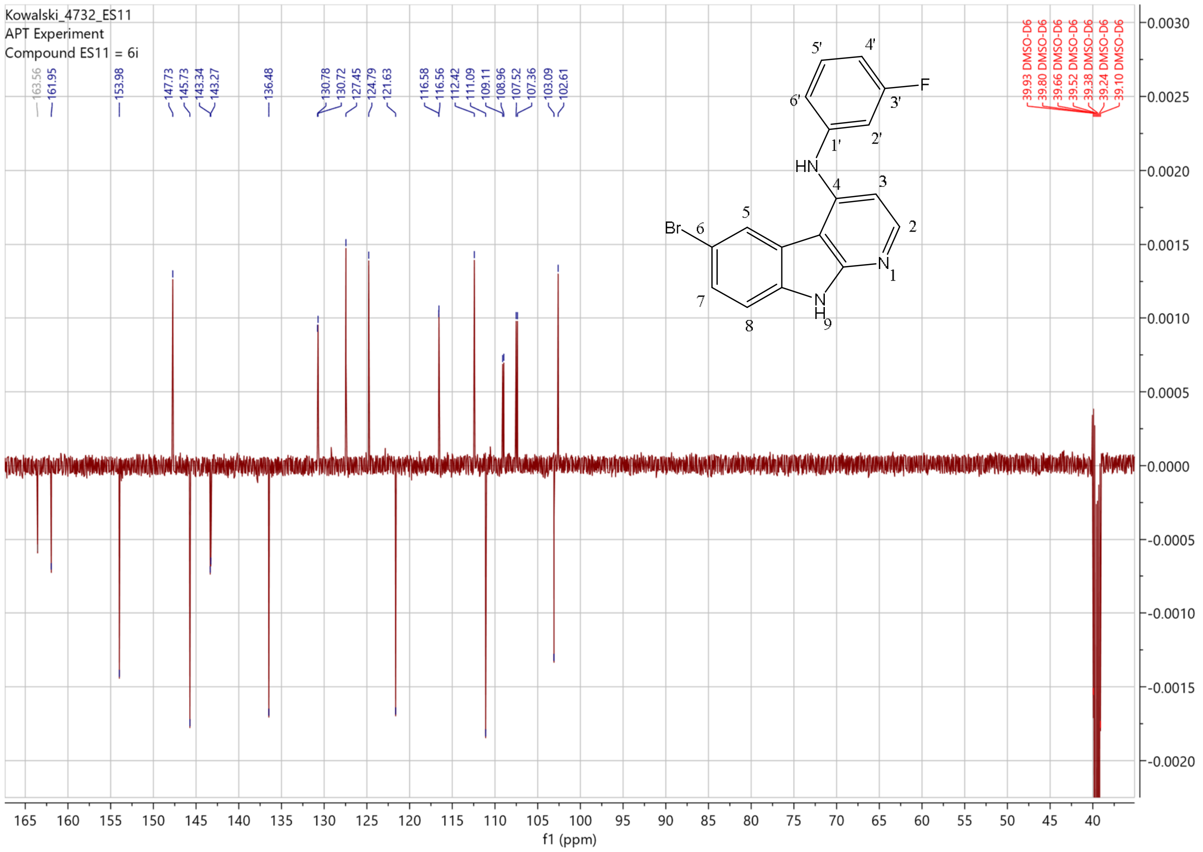

^{13}C NMR spectrum of compound **6m**

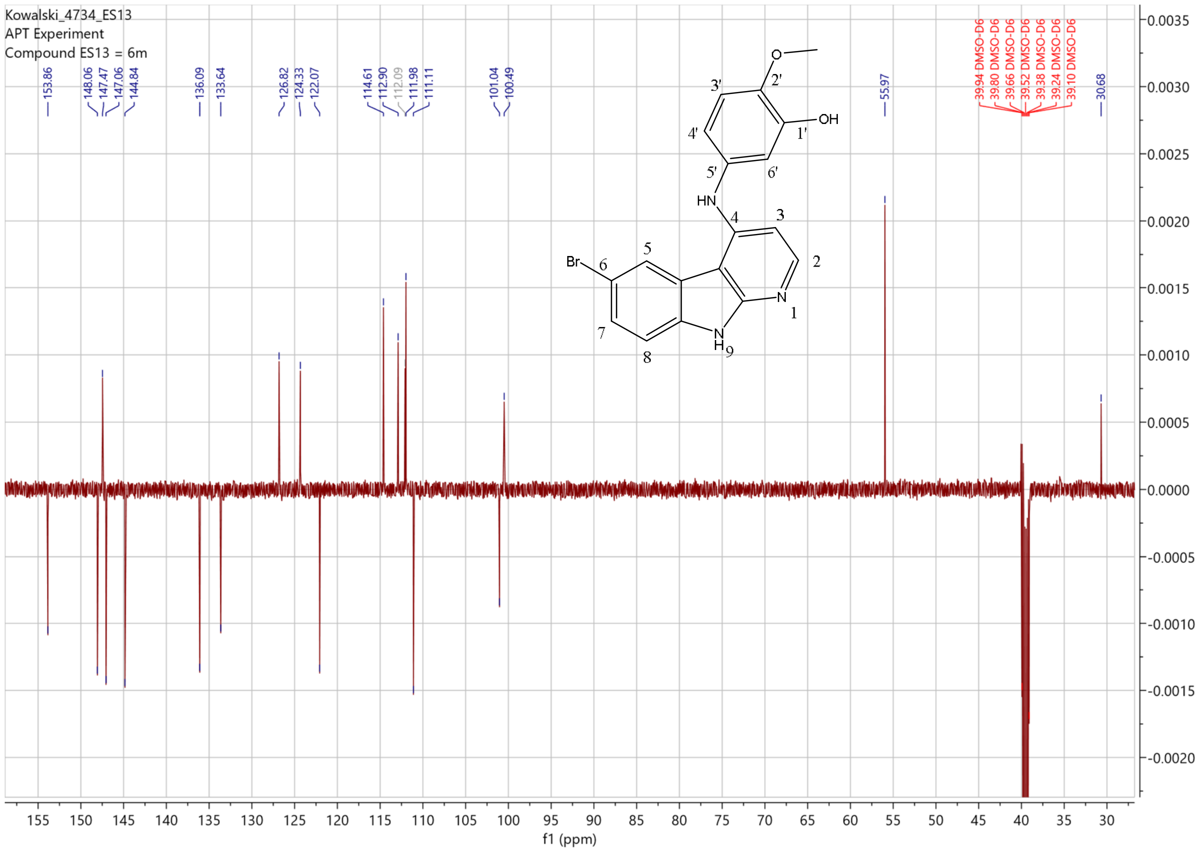

^{13}C NMR spectrum of compound **8c**

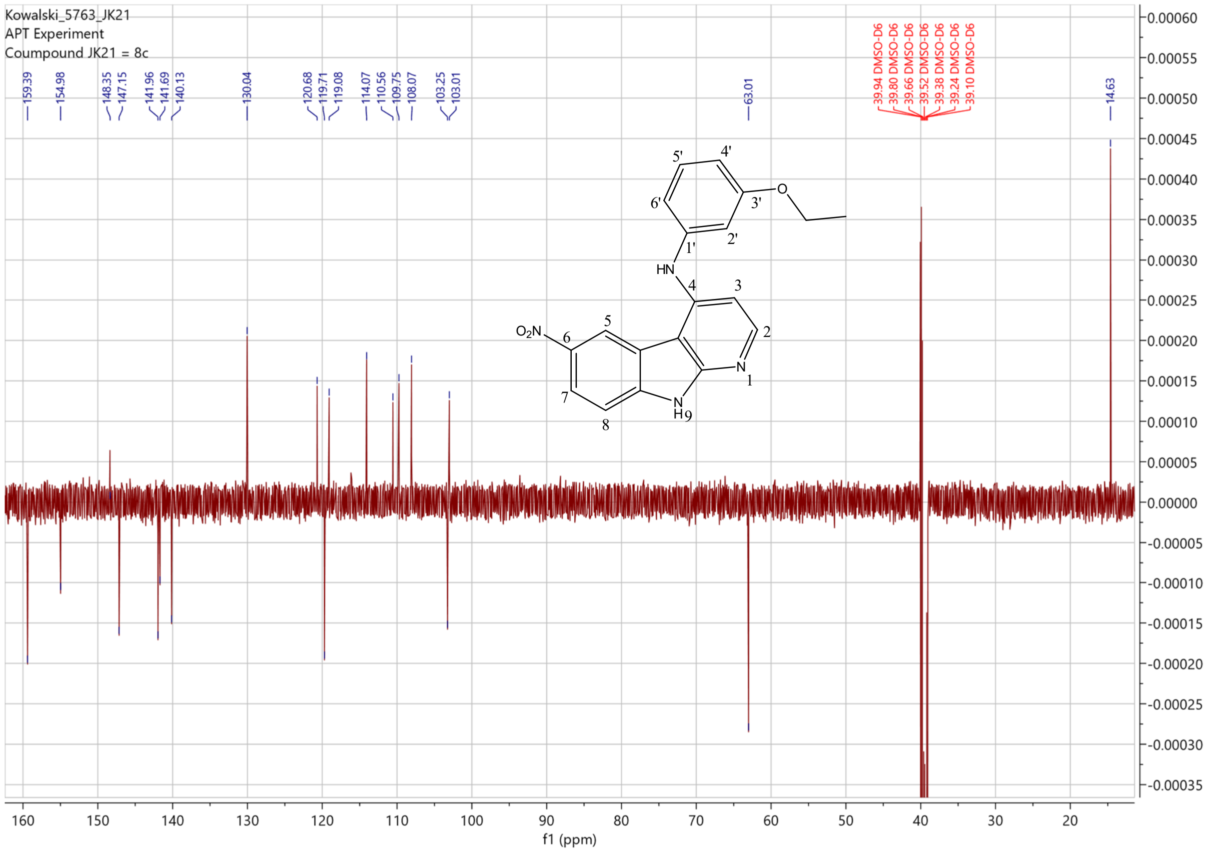

^{13}C NMR spectrum of compound **8d**

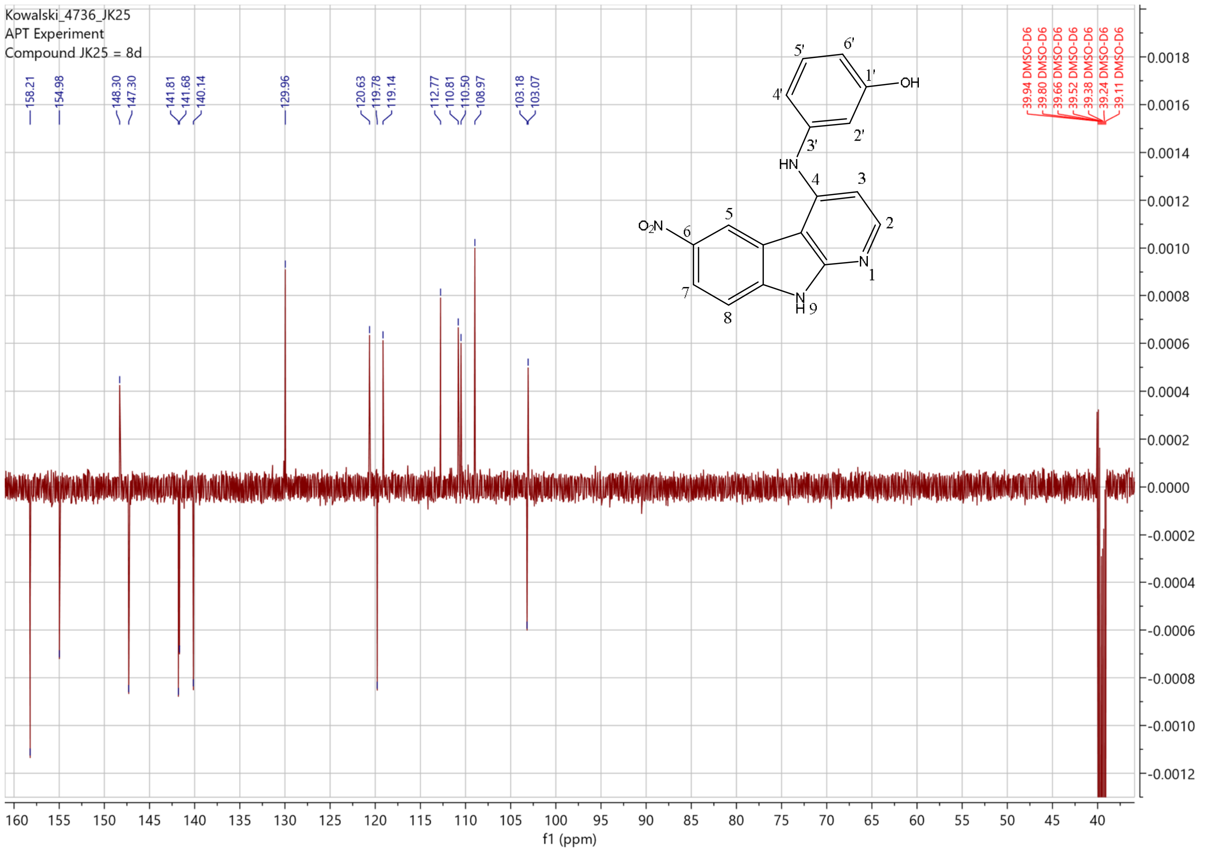

13C NMR spectrum of compound **9a**

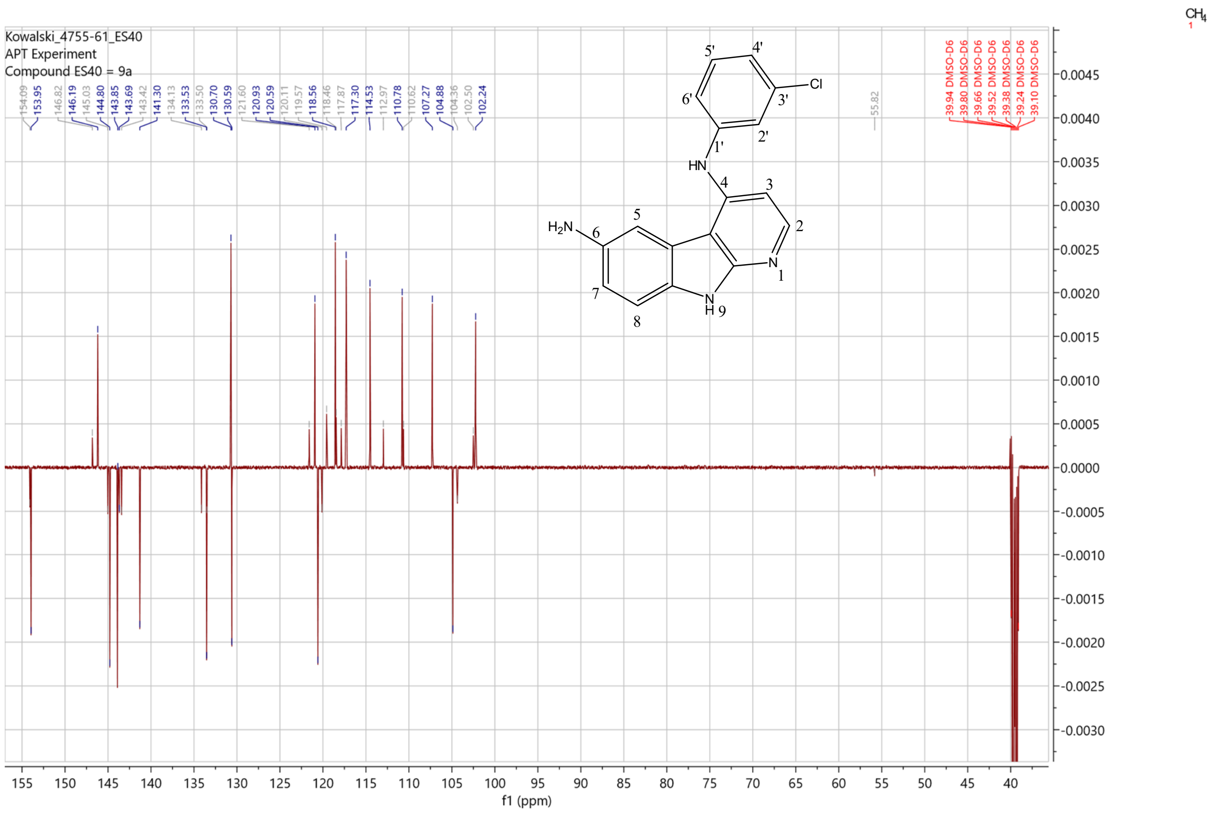

13C NMR spectrum of compound **9b**

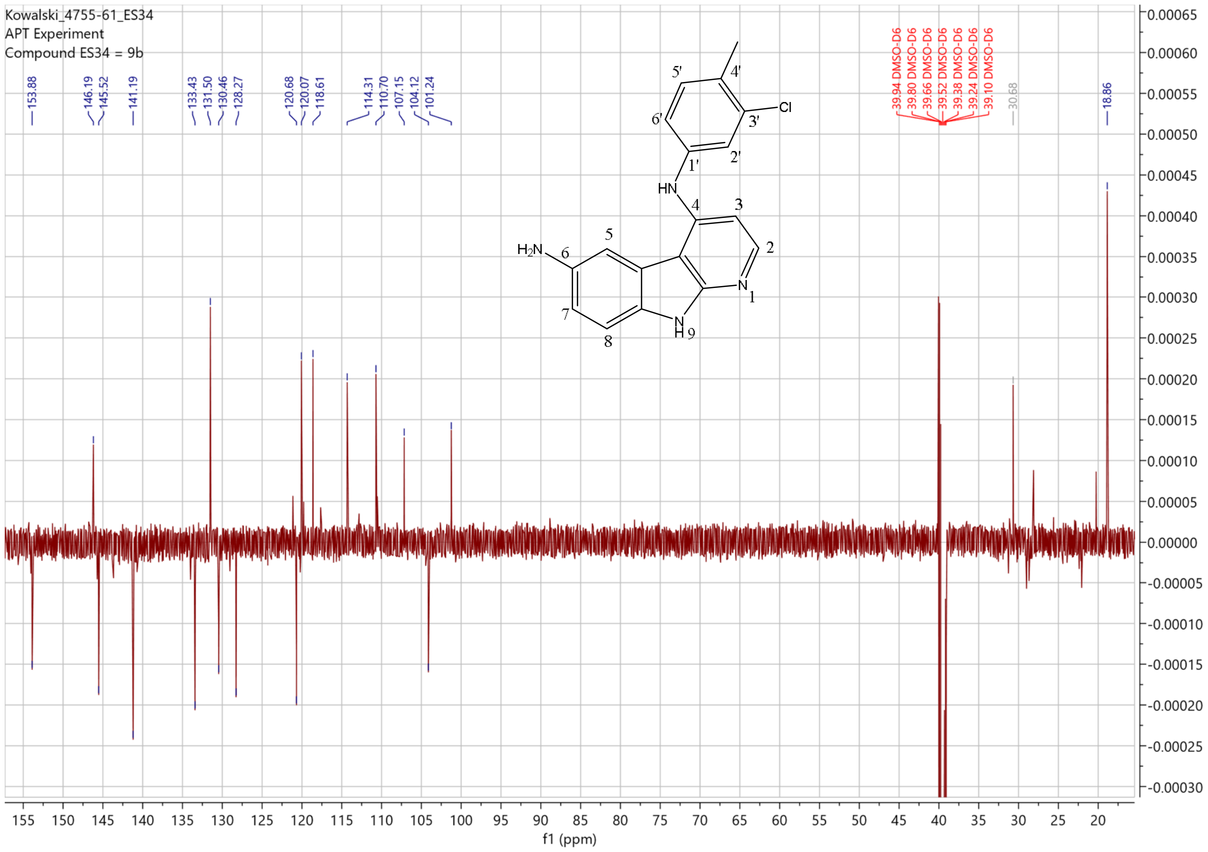

^{13}C NMR spectrum of compound **9c**

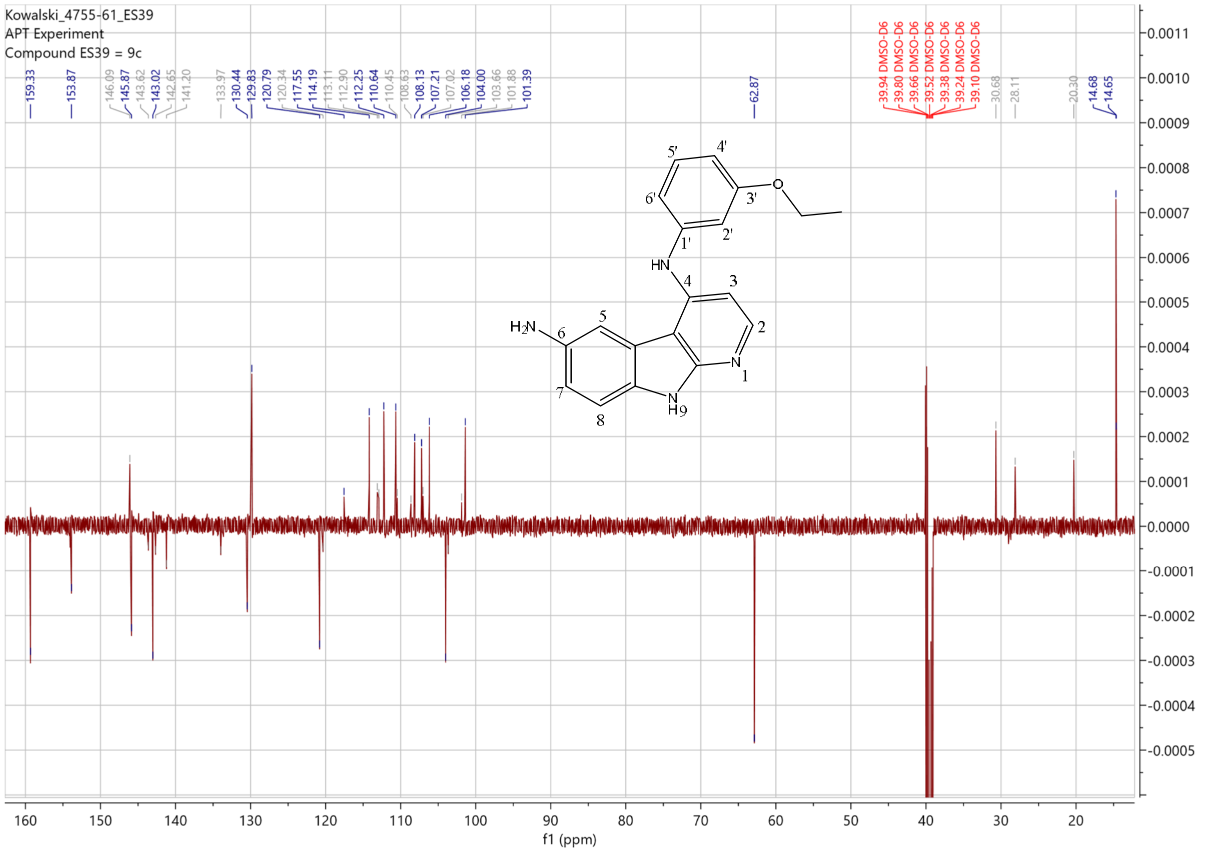

^{13}C NMR spectrum of compound **9d**

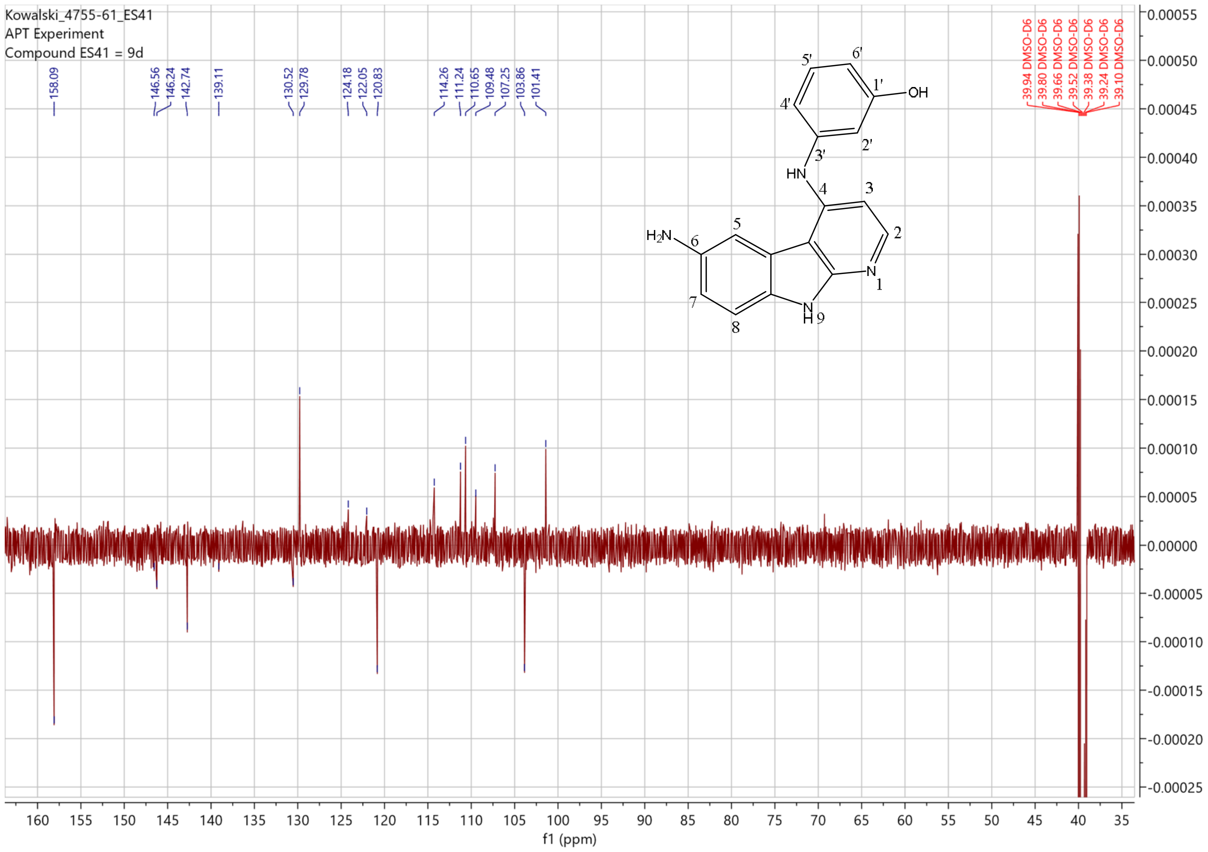

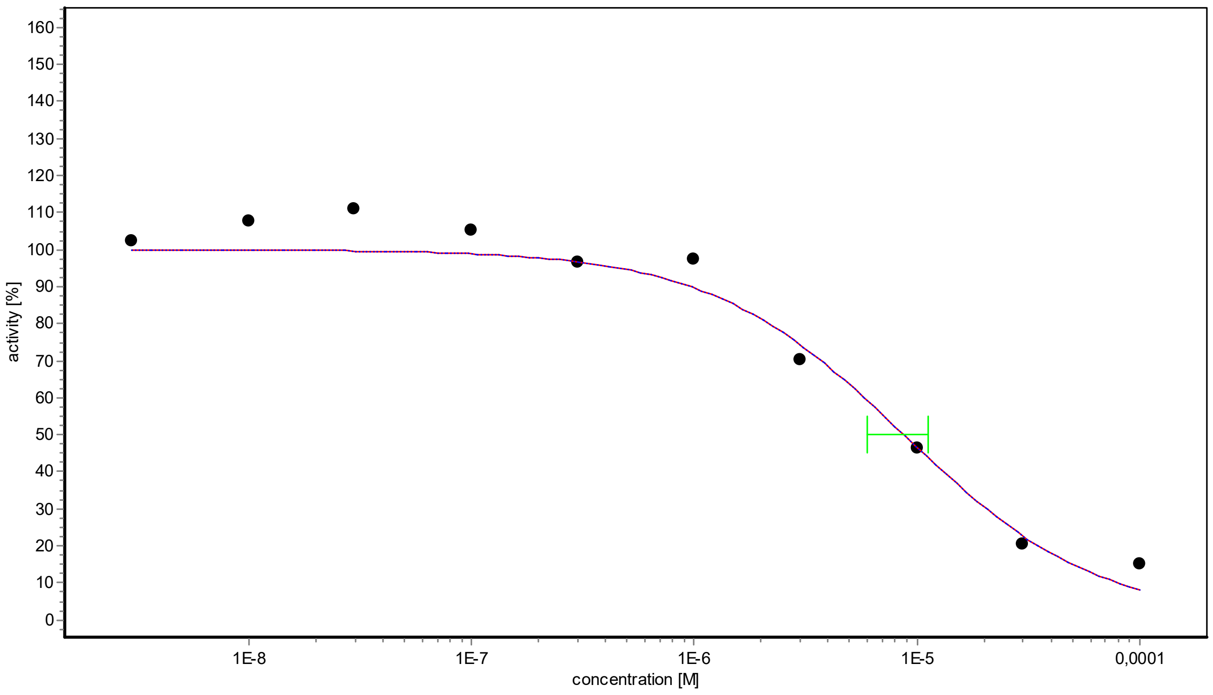

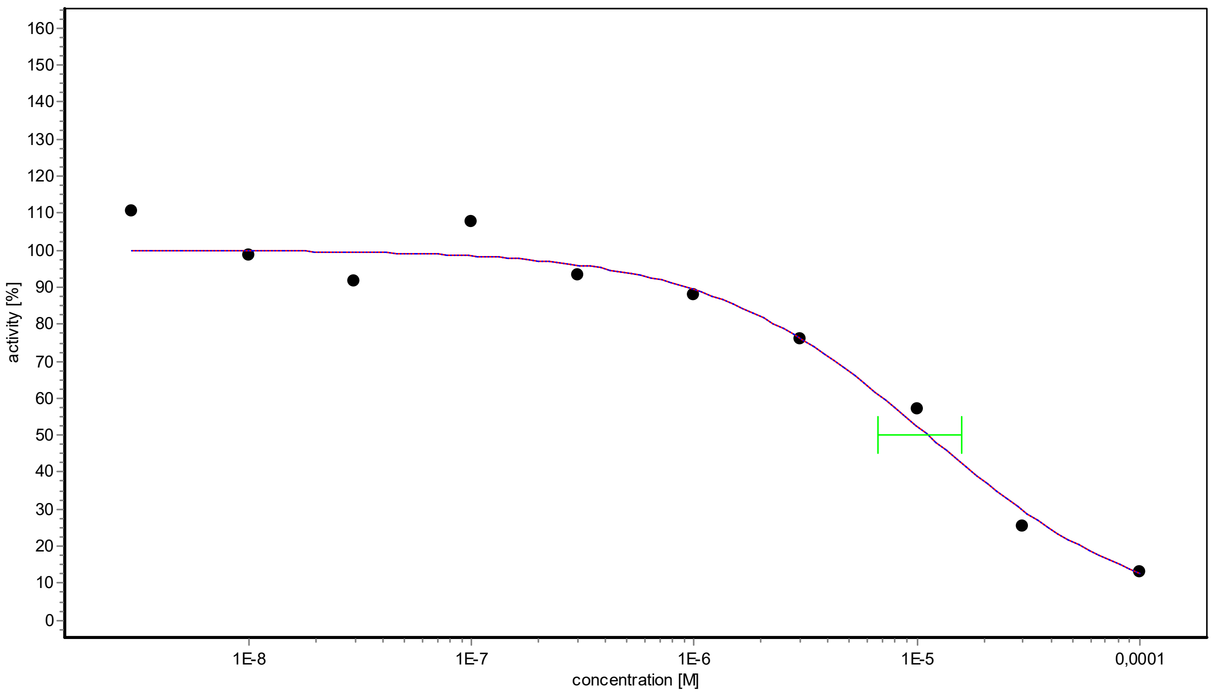

Figure S1. Enzyme activity curves for compound **2c** for Brk 1st (top) and Brk 2nd (bottom)

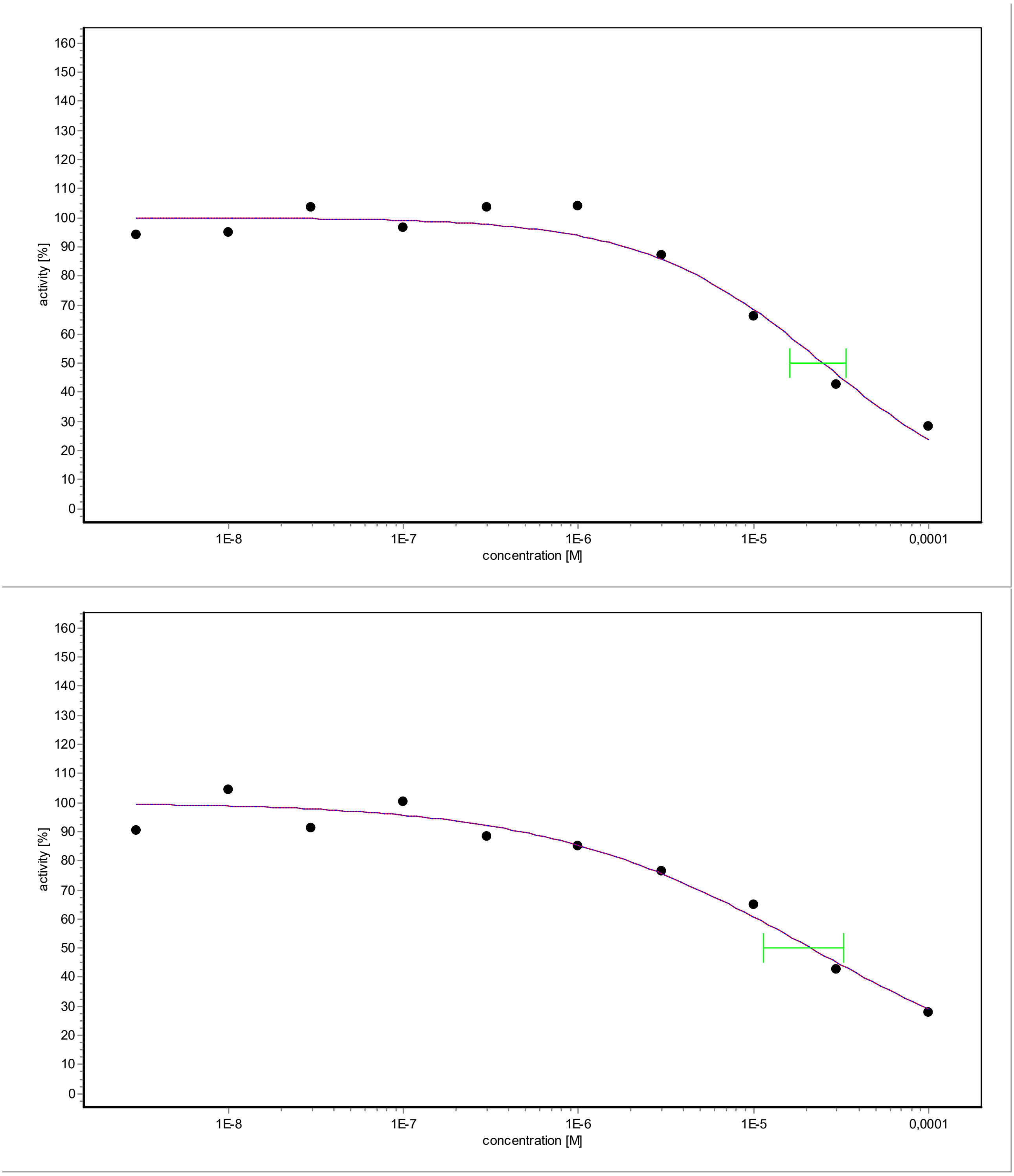

Figure S2. Enzyme activity curves for compound **2c** for HER2 1st (top) and HER2 2nd (bottom)

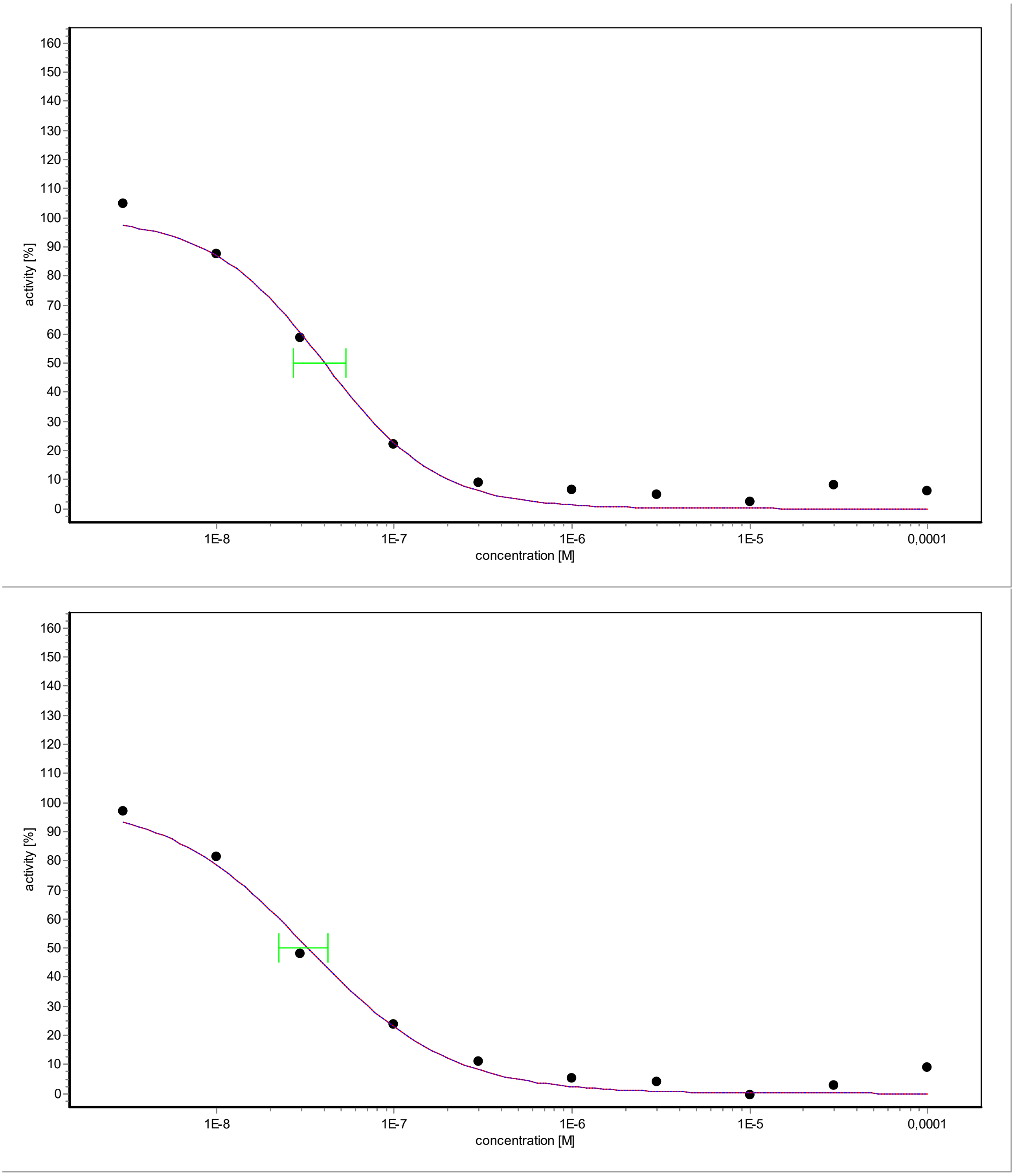

Figure S3. Enzyme activity curves for compound **6k** for Brk 1st (top) and Brk 2nd (bottom)

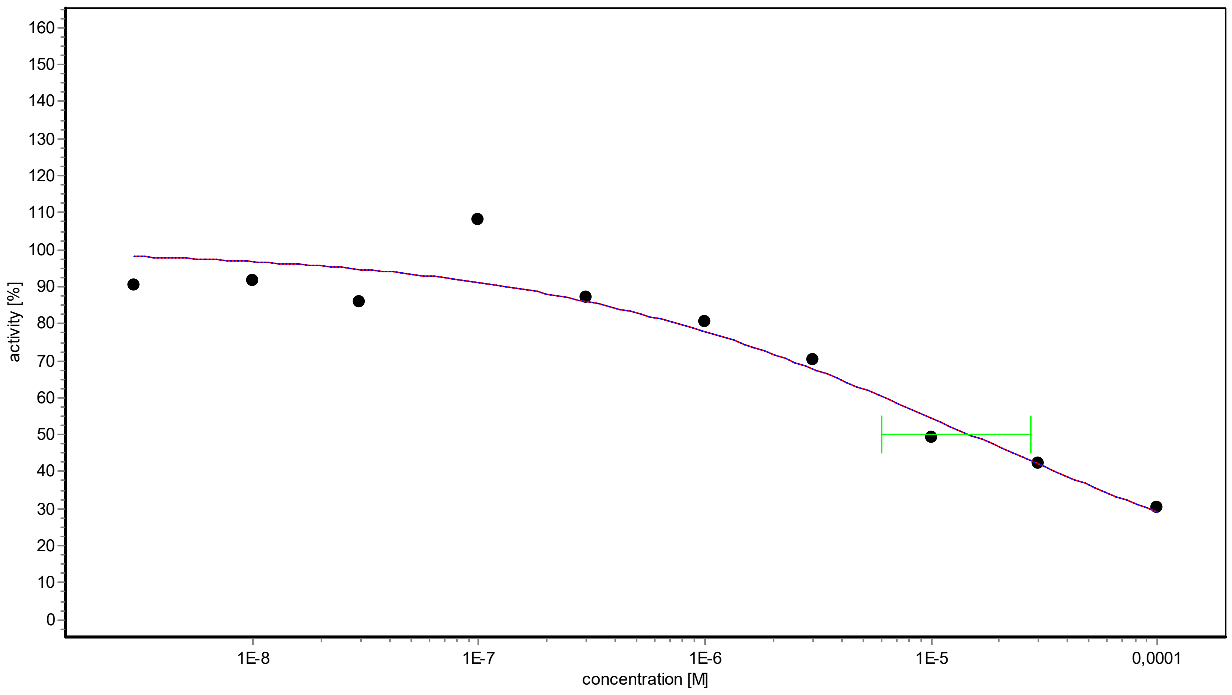

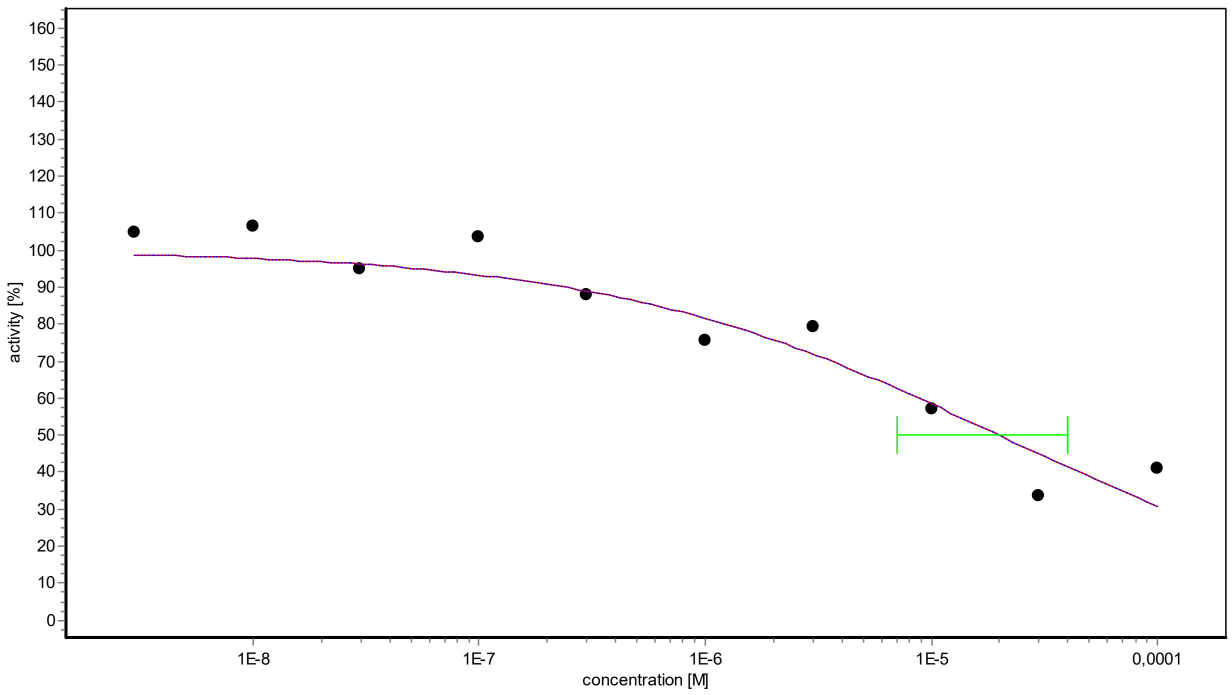

Figure S4. Enzyme activity curves for compound **6k** for HER2 1st (top) and HER2 2nd (bottom)

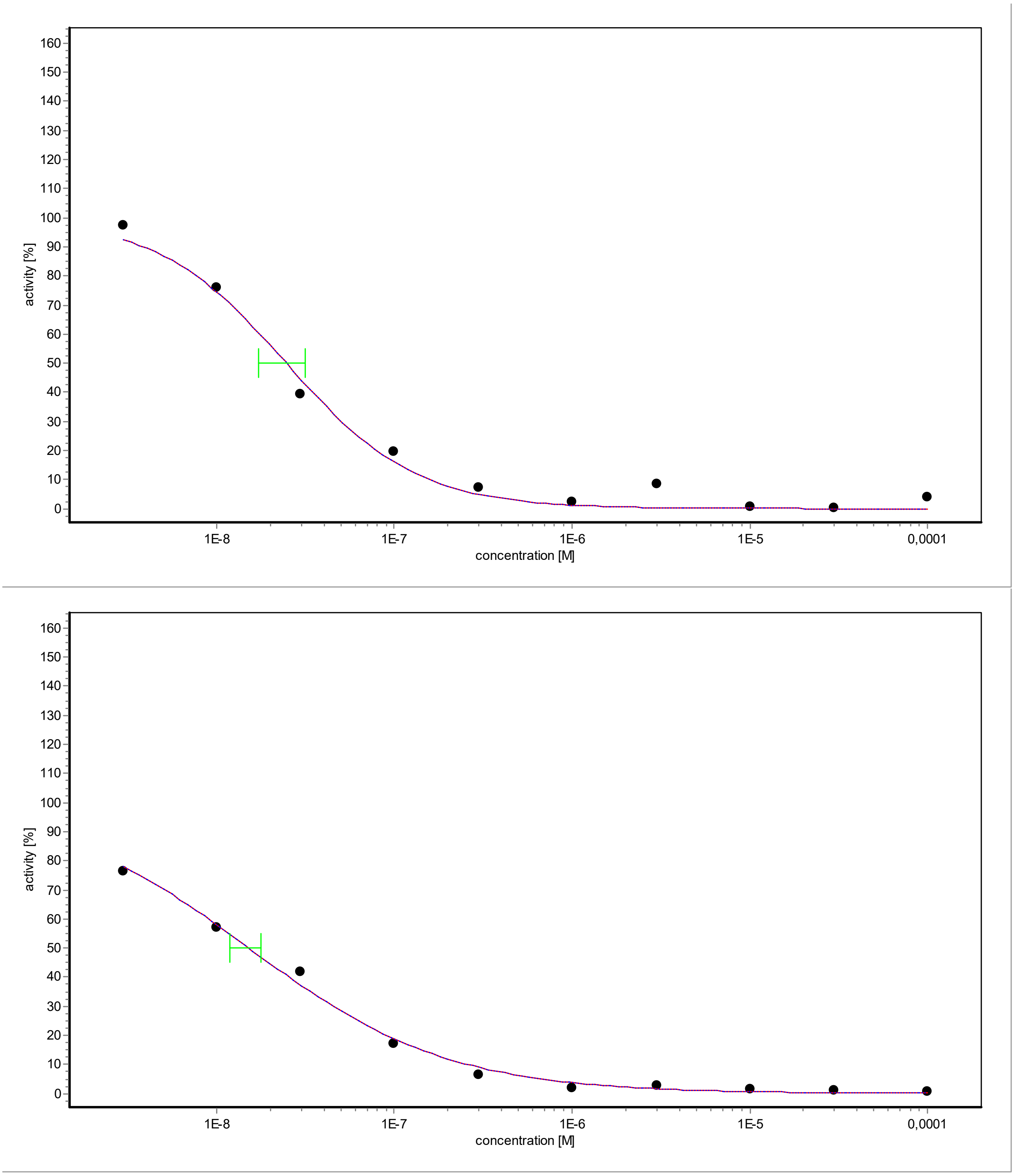

Figure S5. Enzyme activity curves for compound **6m** for Brk 1st (top) and Brk 2nd (bottom)

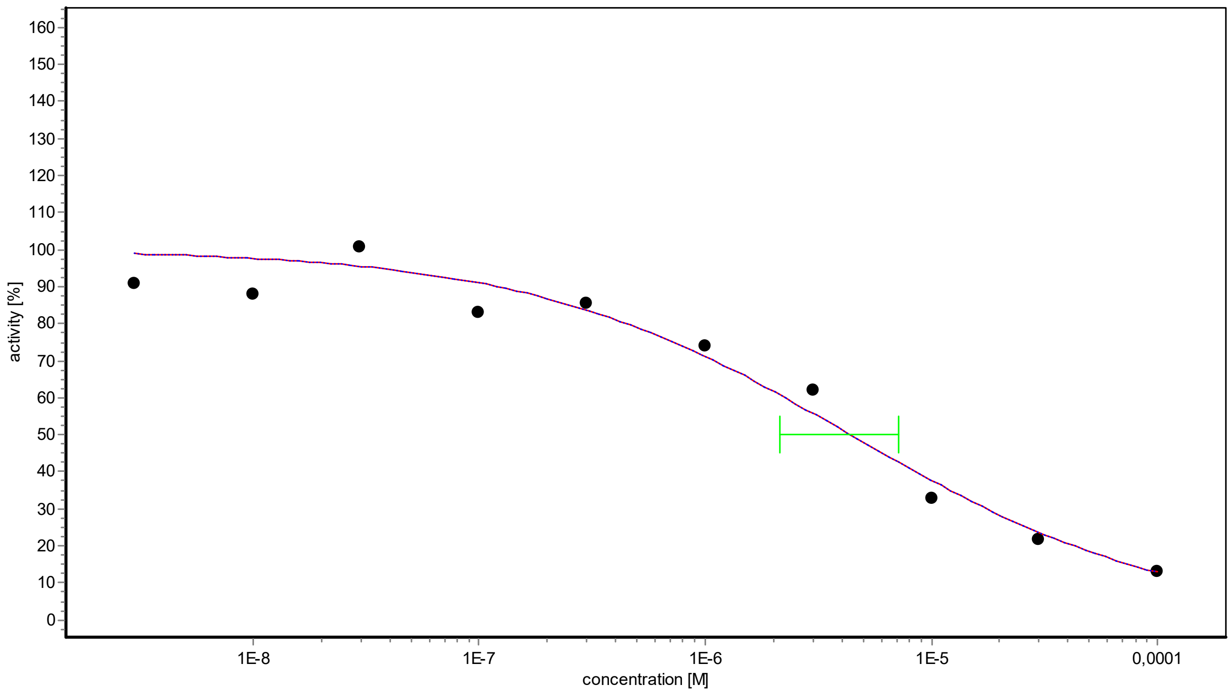

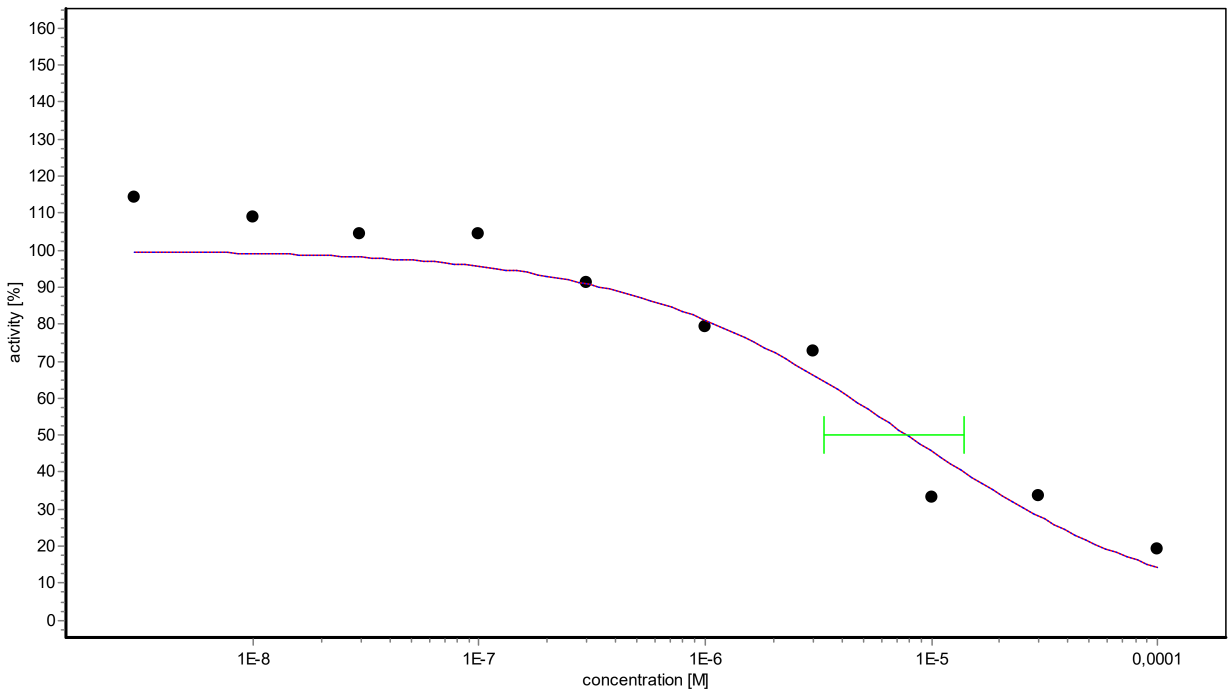

Figure S6. Enzyme activity curves for compound **6m** for HER2 1st (top) and HER2 2nd (bottom)

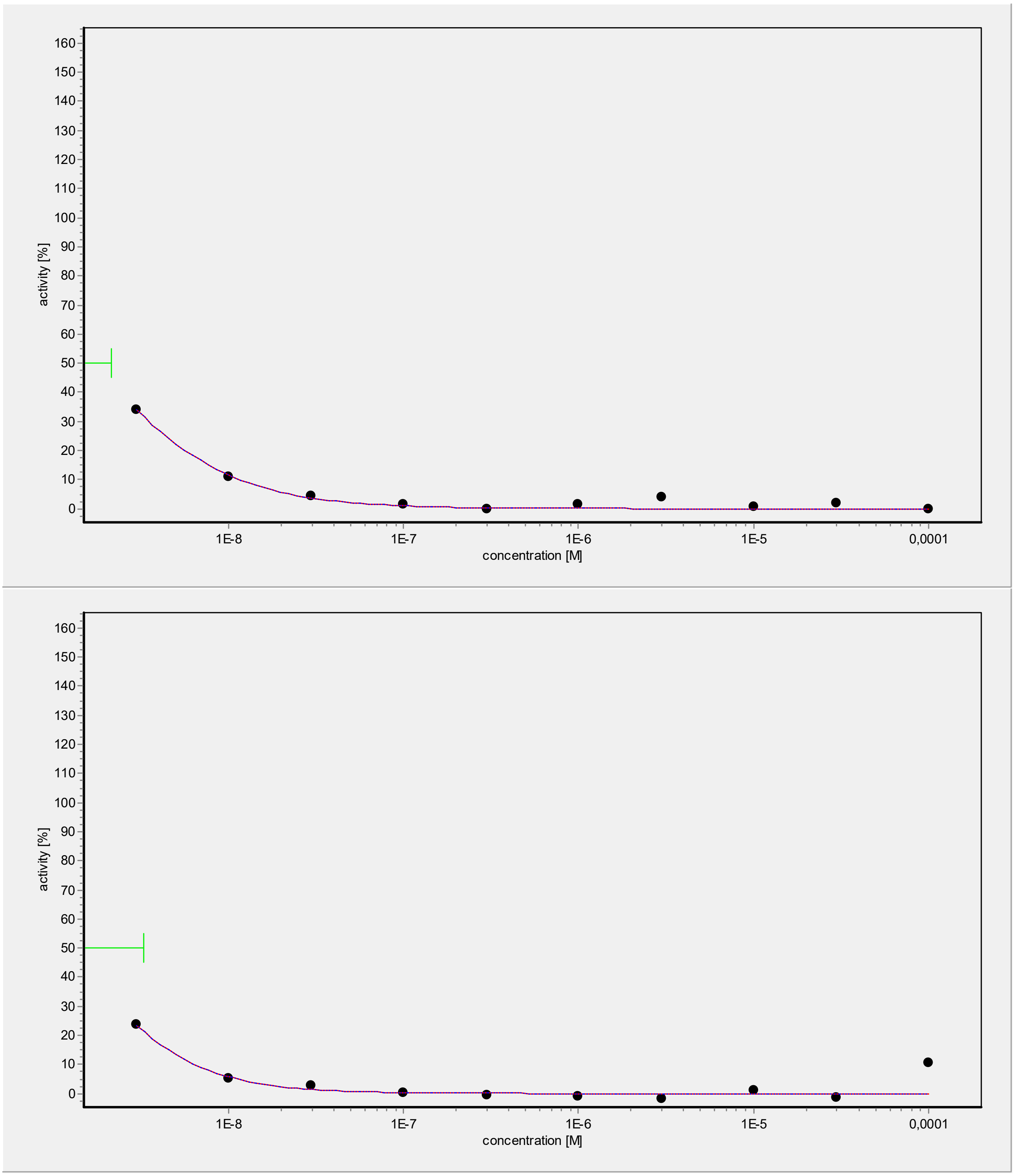

Figure S7. Enzyme activity curves for compound **8d** for Brk 1st (top) and Brk 2nd (bottom)

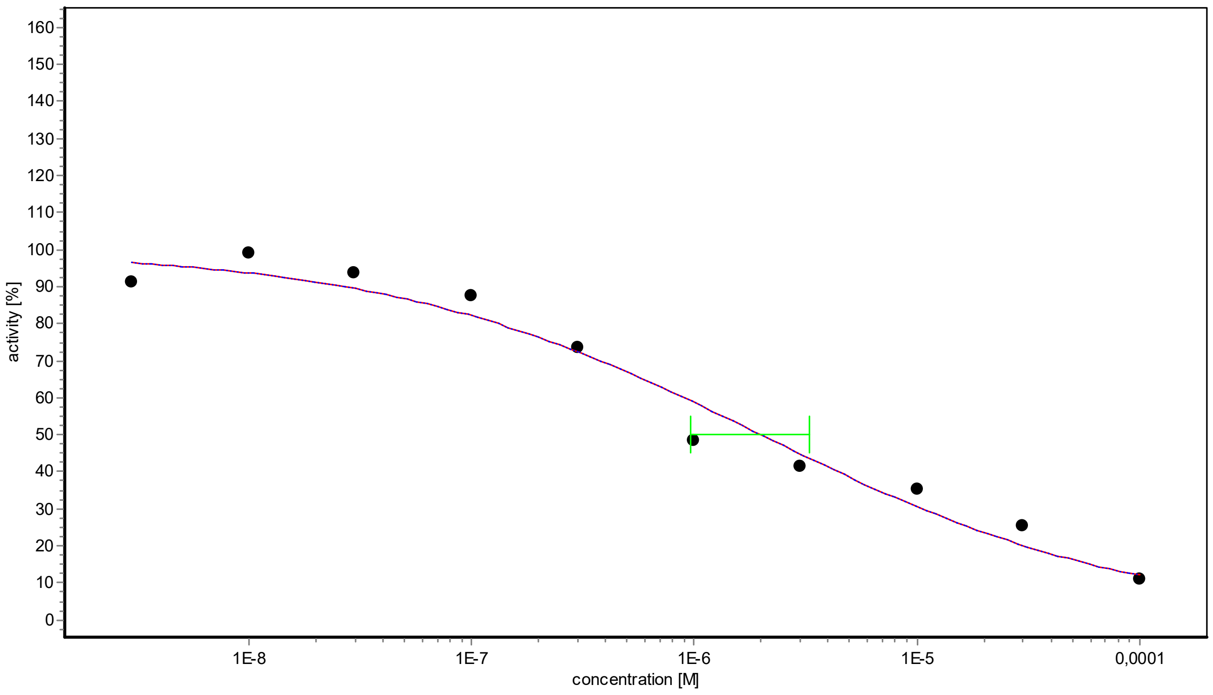

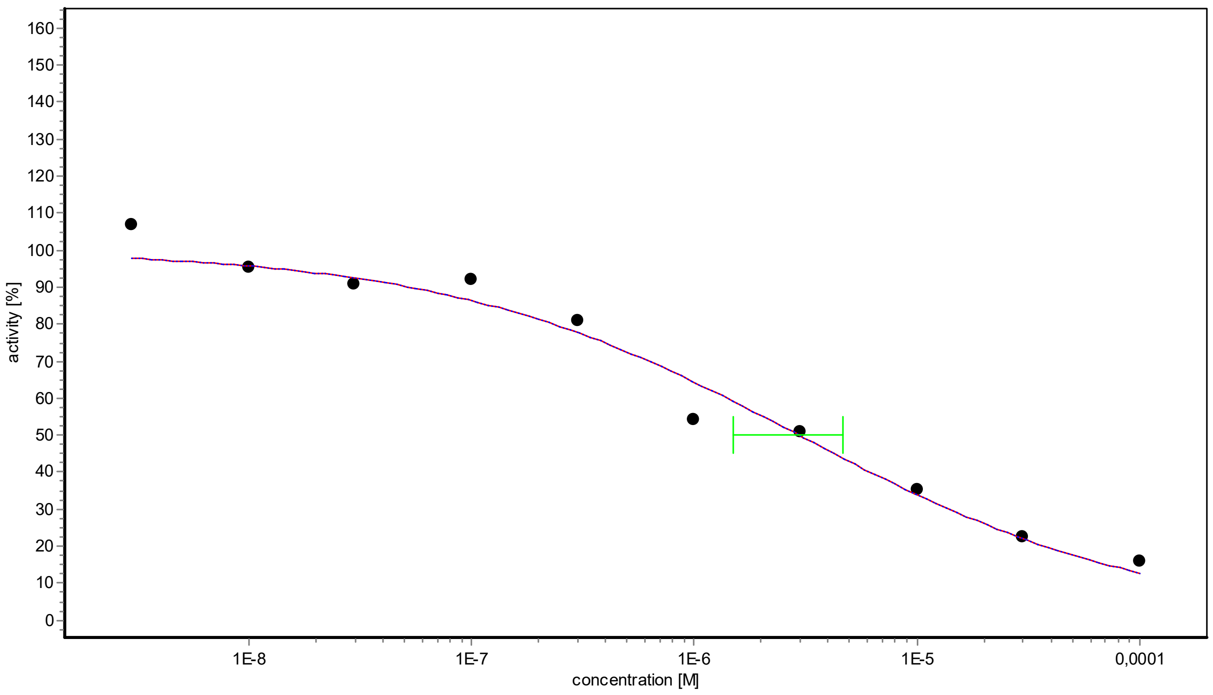

Figure S8. Enzyme activity curves for compound **8d** for HER2 1st (top) and HER2 2nd (bottom)

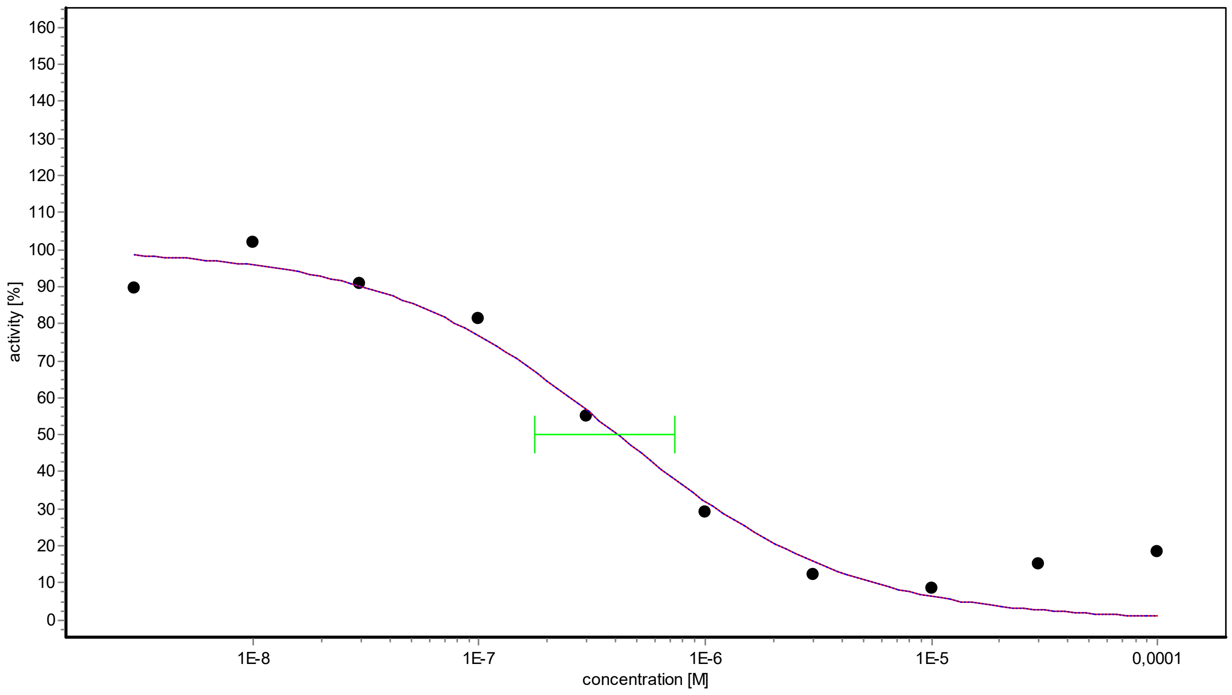

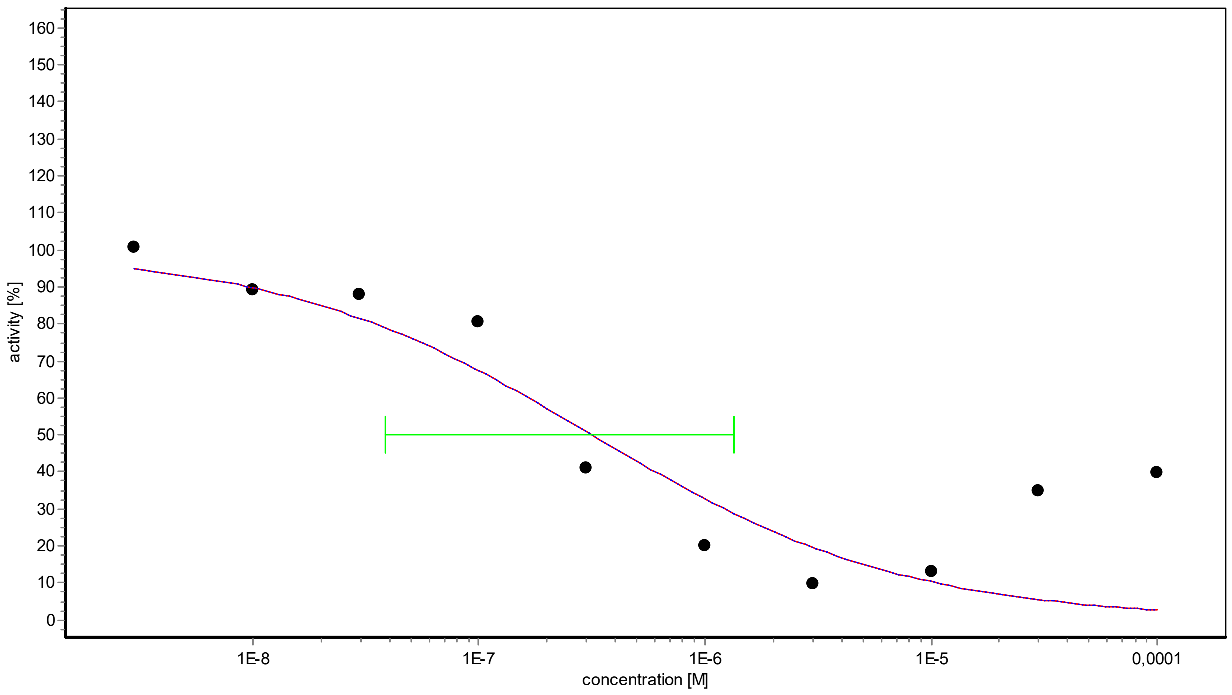

Figure S9. Enzyme activity curves for compound **9a** for Brk 1st (top) and Brk 2nd (bottom)

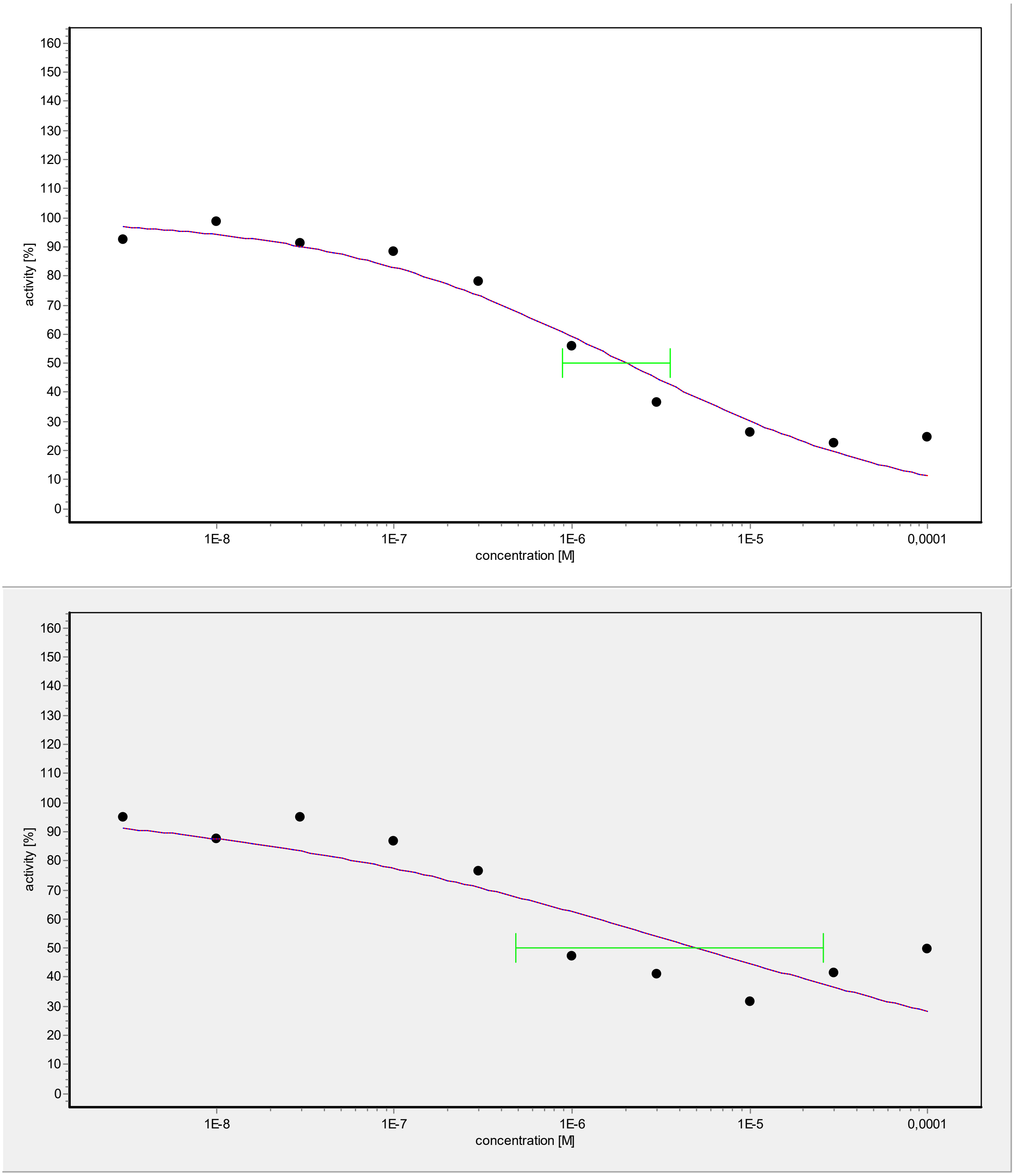

Figure S10. Enzyme activity curves for compound **9a** for HER2 1st (top) and HER2 2nd (bottom)

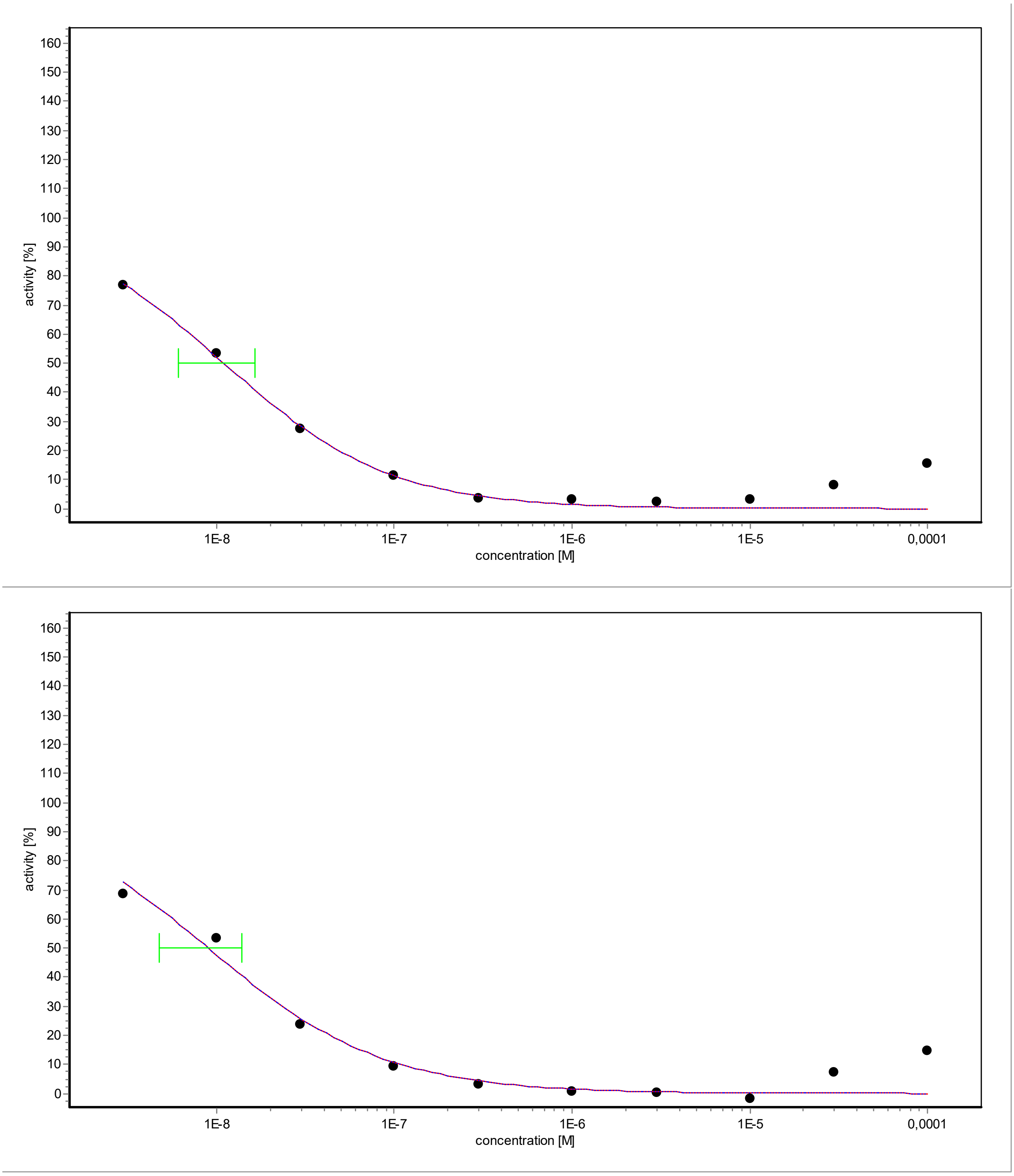

Figure S11. Enzyme activity curves for compound **9d** for Brk 1st (top) and Brk 2nd (bottom)

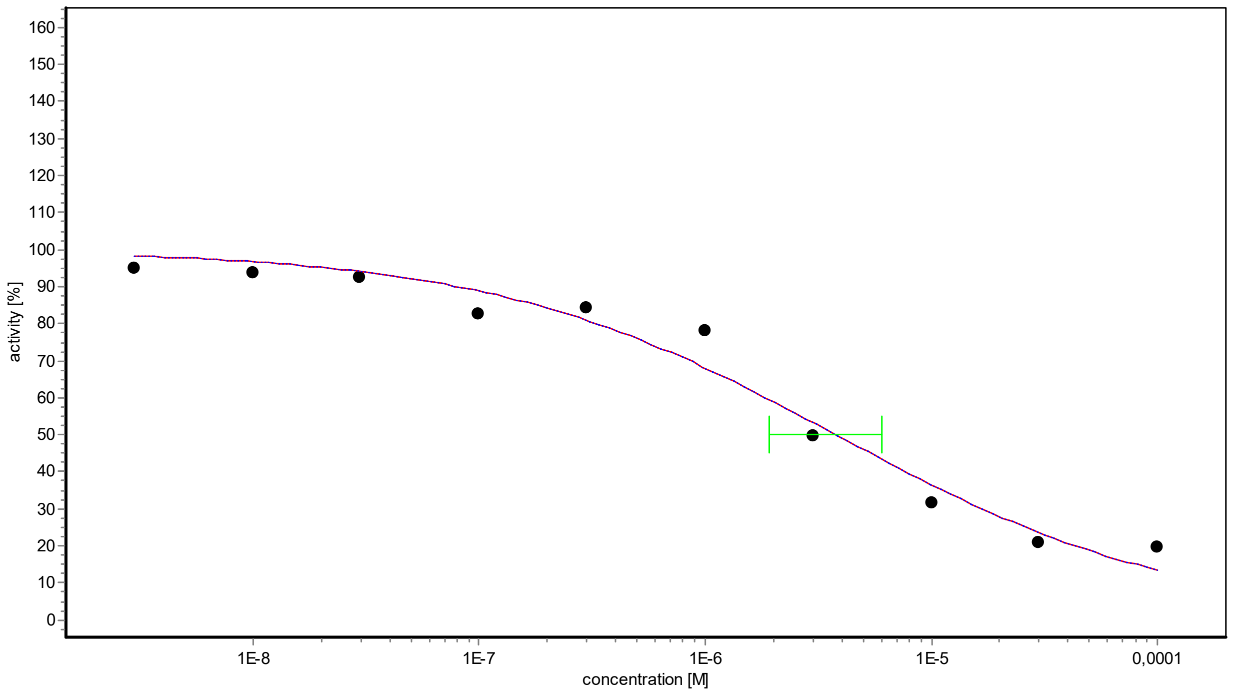

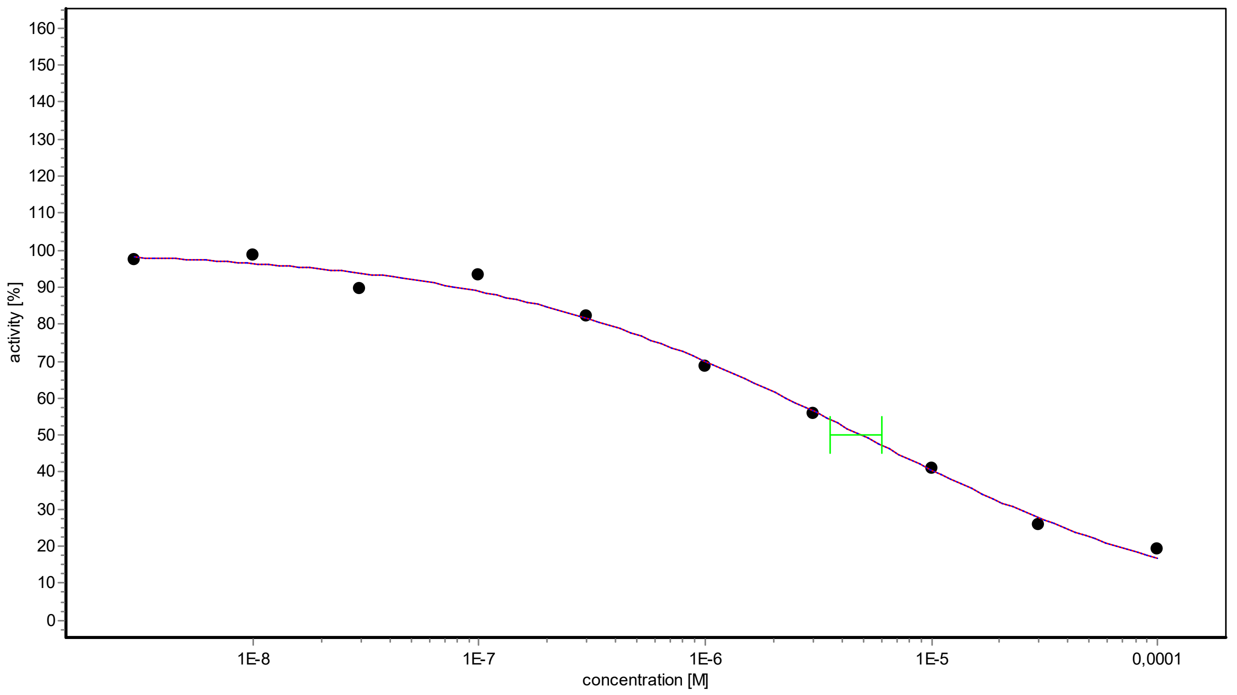

Figure S12. Enzyme activity curves for compound **9d** for HER2 1st (top) and HER2 2nd (bottom)

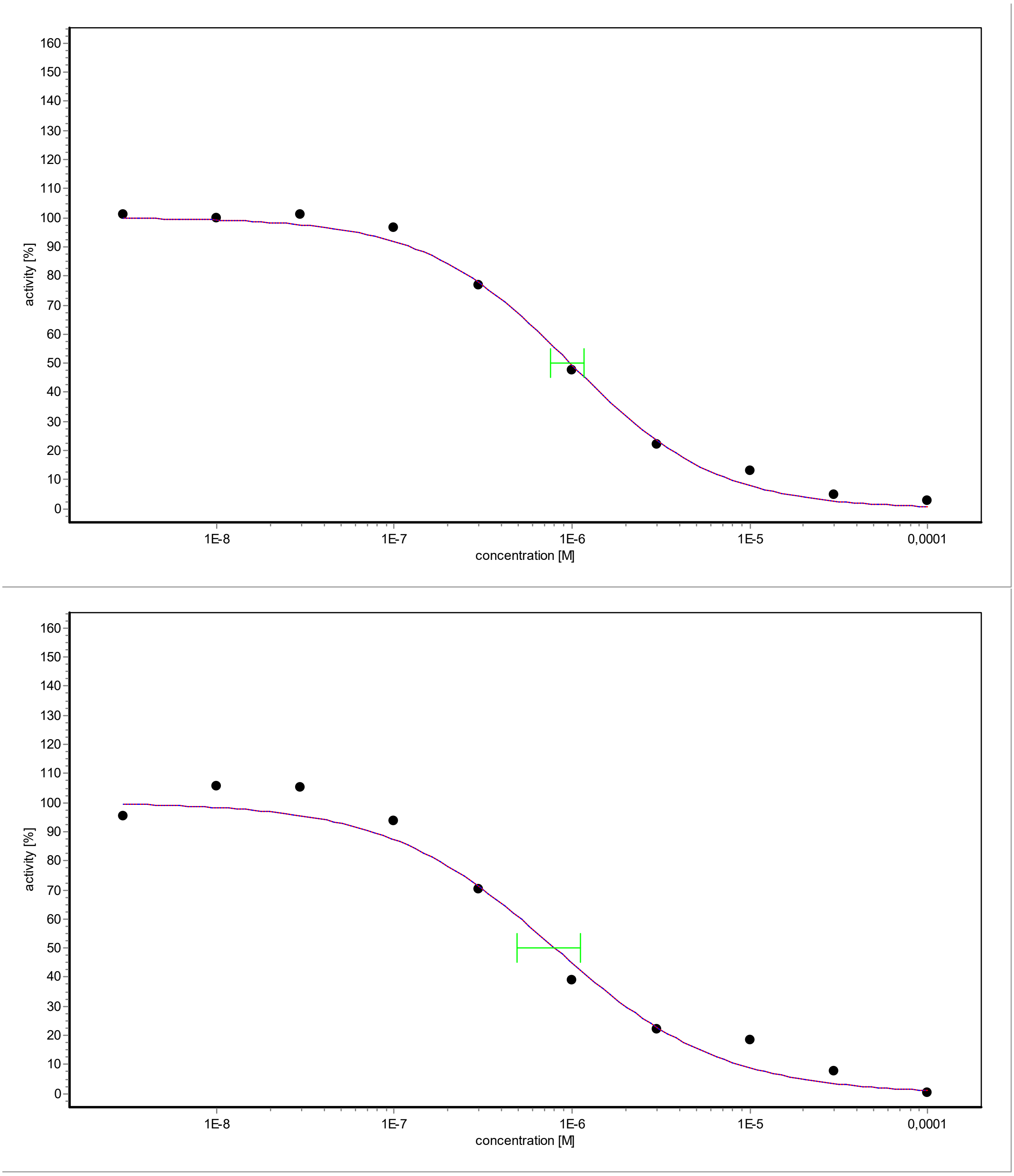

Figure S13. Enzyme activity curves for staurosporine for Brk 1st (top) and Brk 2nd (bottom)

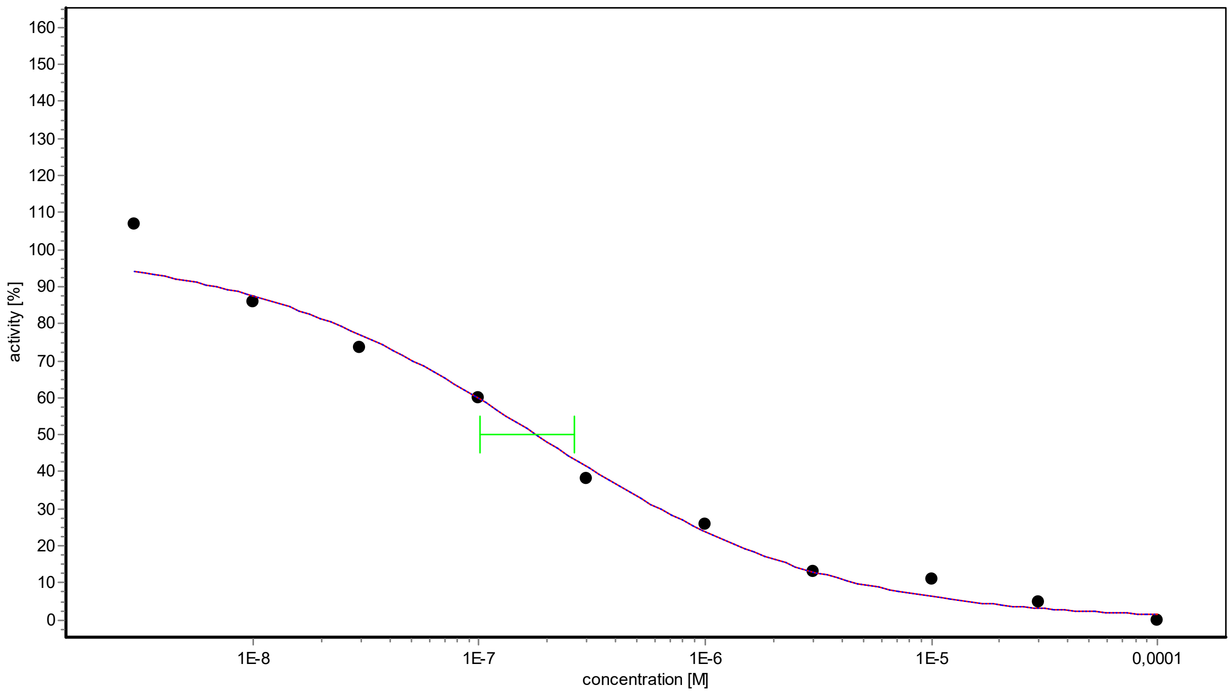

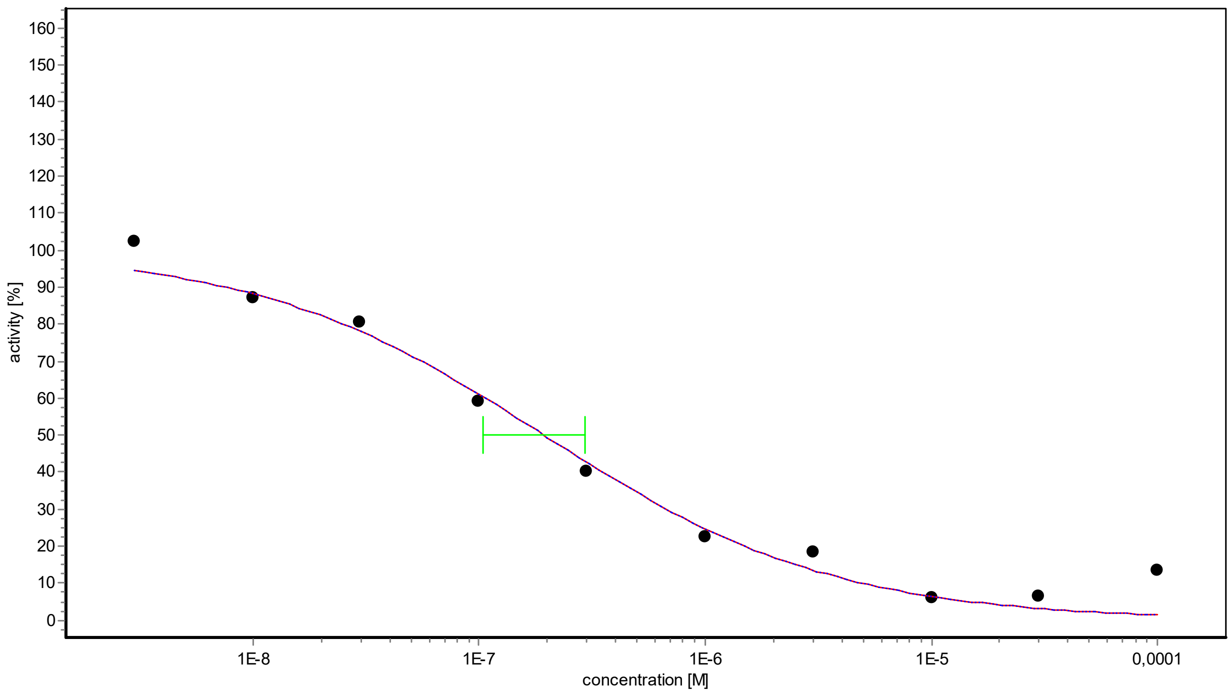

Figure S14. Enzyme activity curves for staurosporine for HER2 1st (top) and HER2 2nd (bottom)

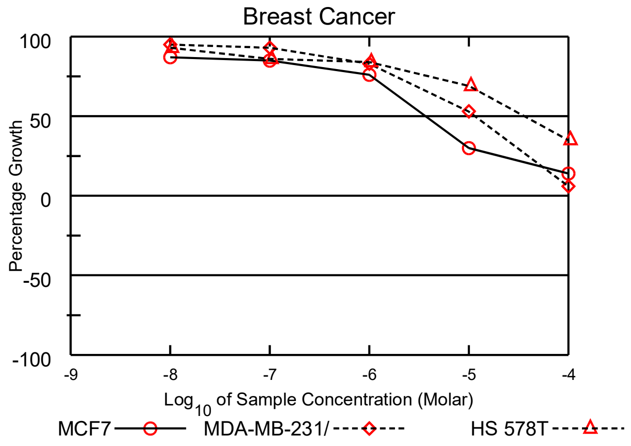

Figure S15. Dose response curves in breast cancer cells for compound **6a**

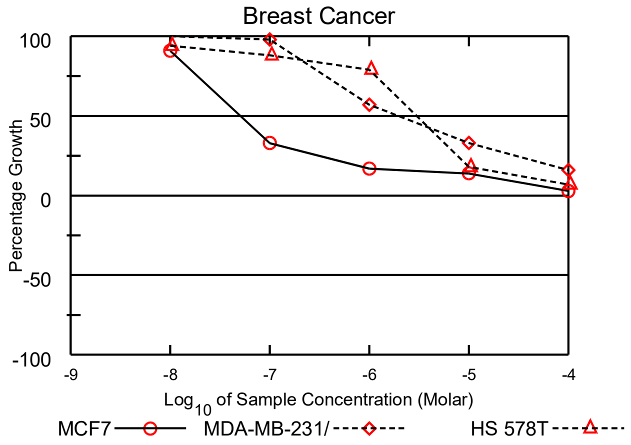

Figure S16. Dose response curves in breast cancer cells for compound **6k**

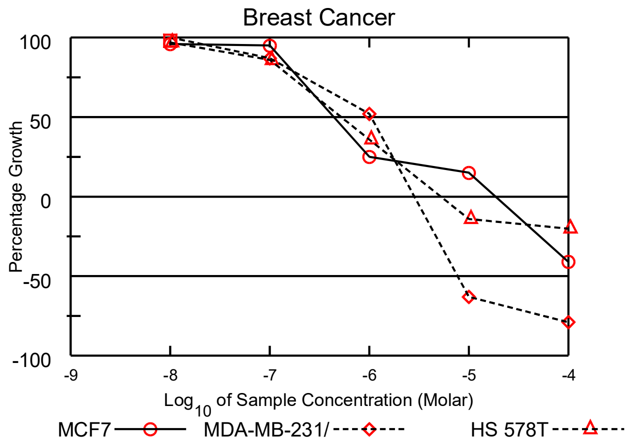

Figure S17. Dose response curves in breast cancer cells for compound **6l**
